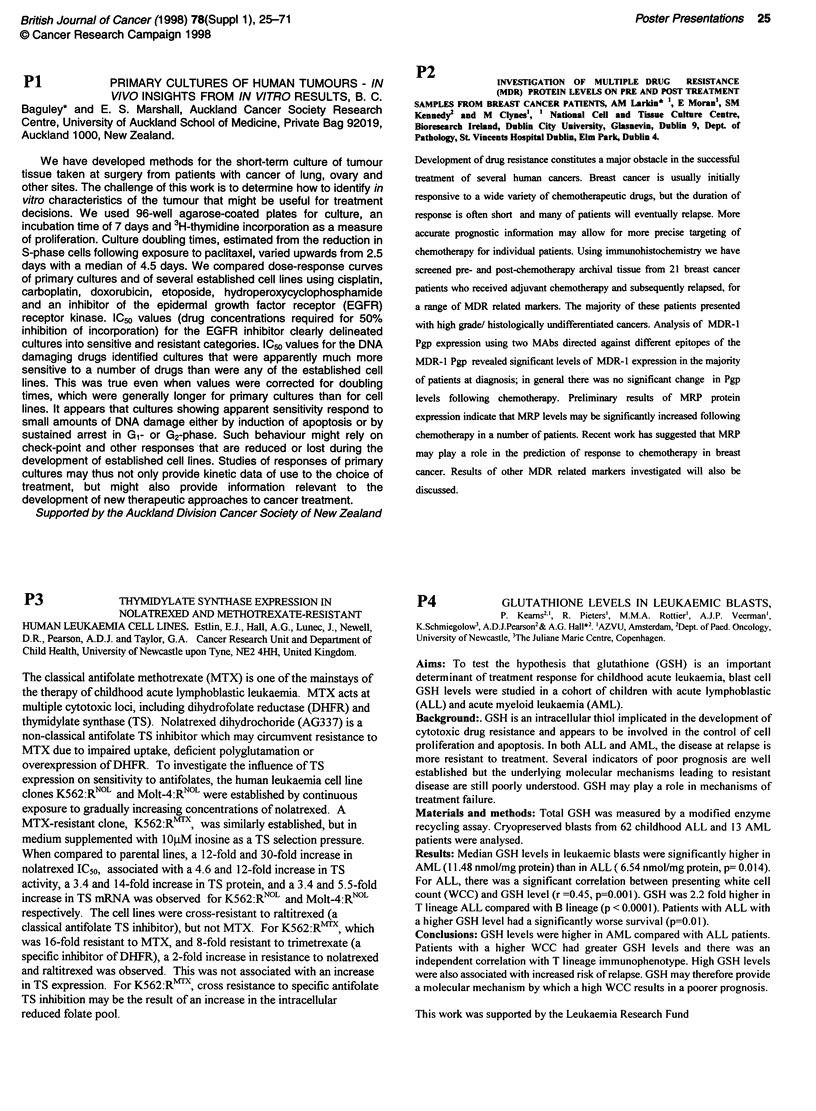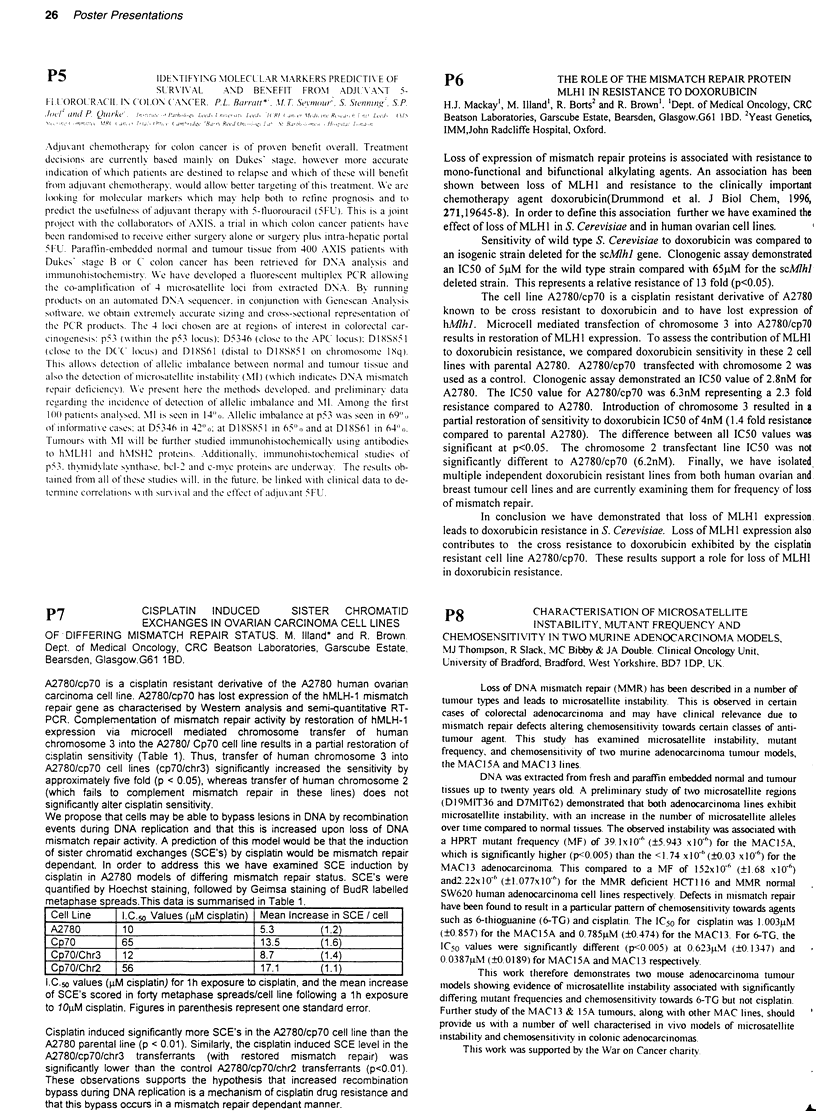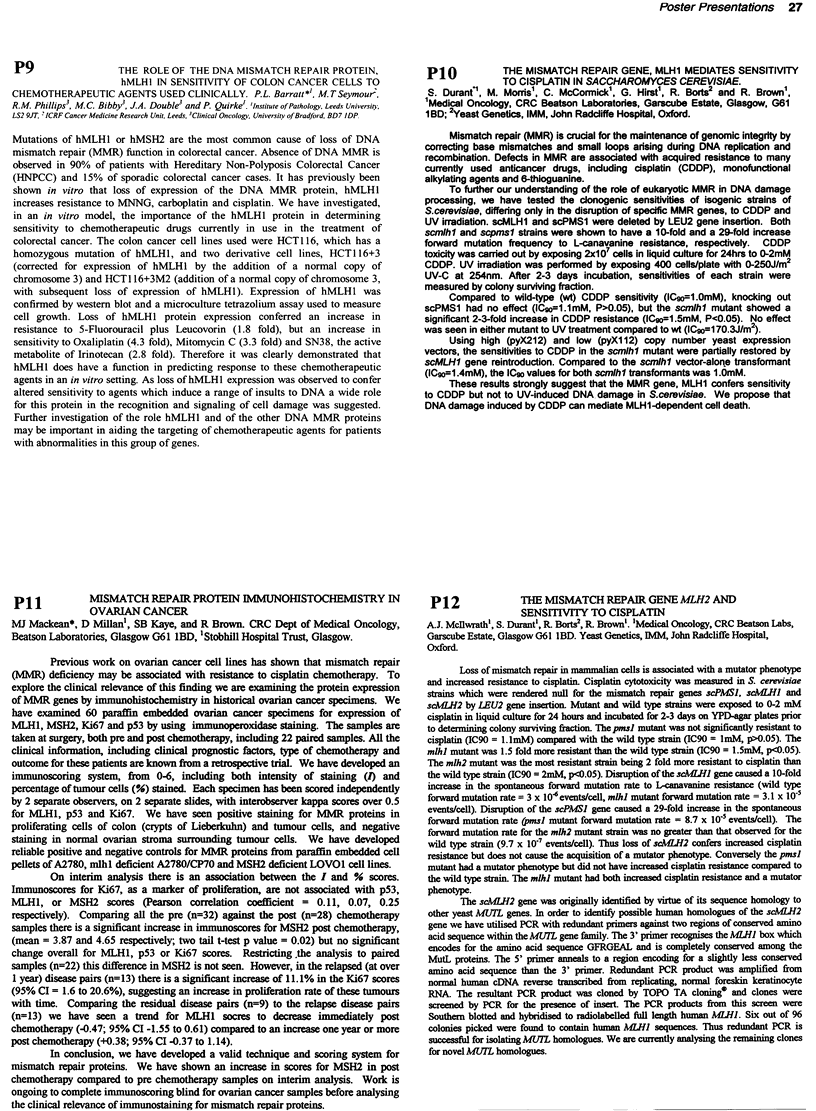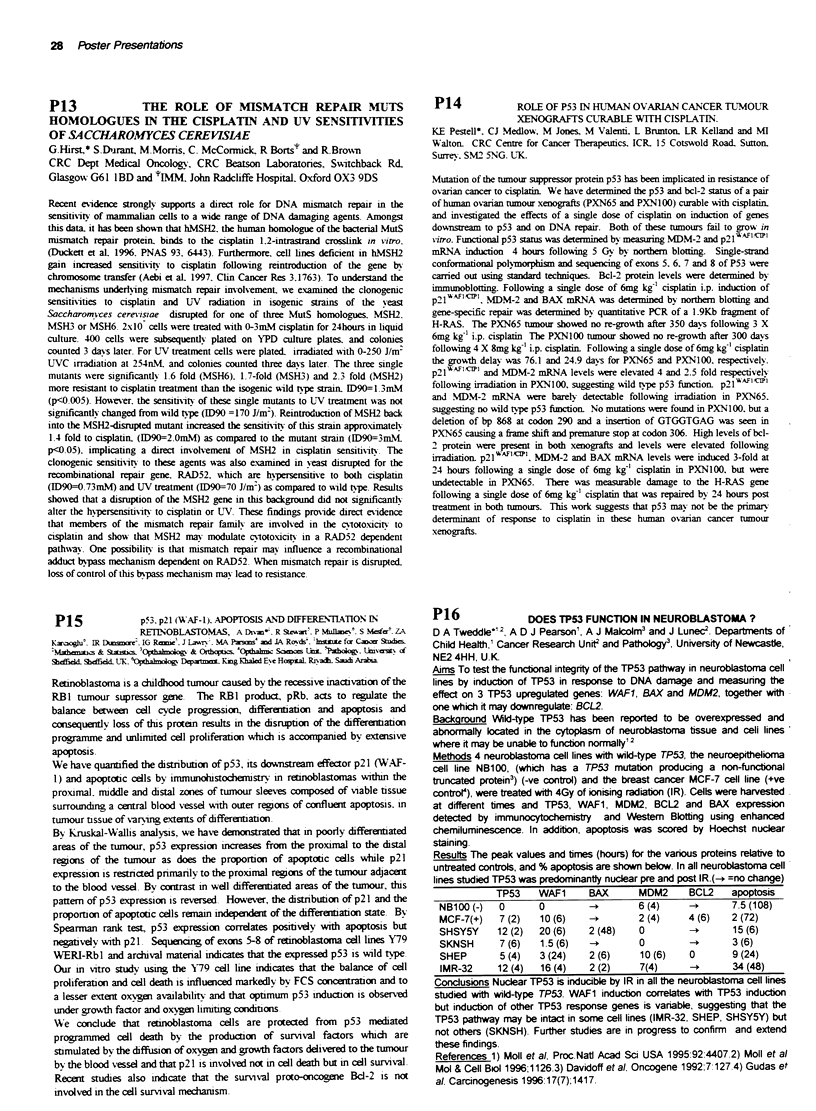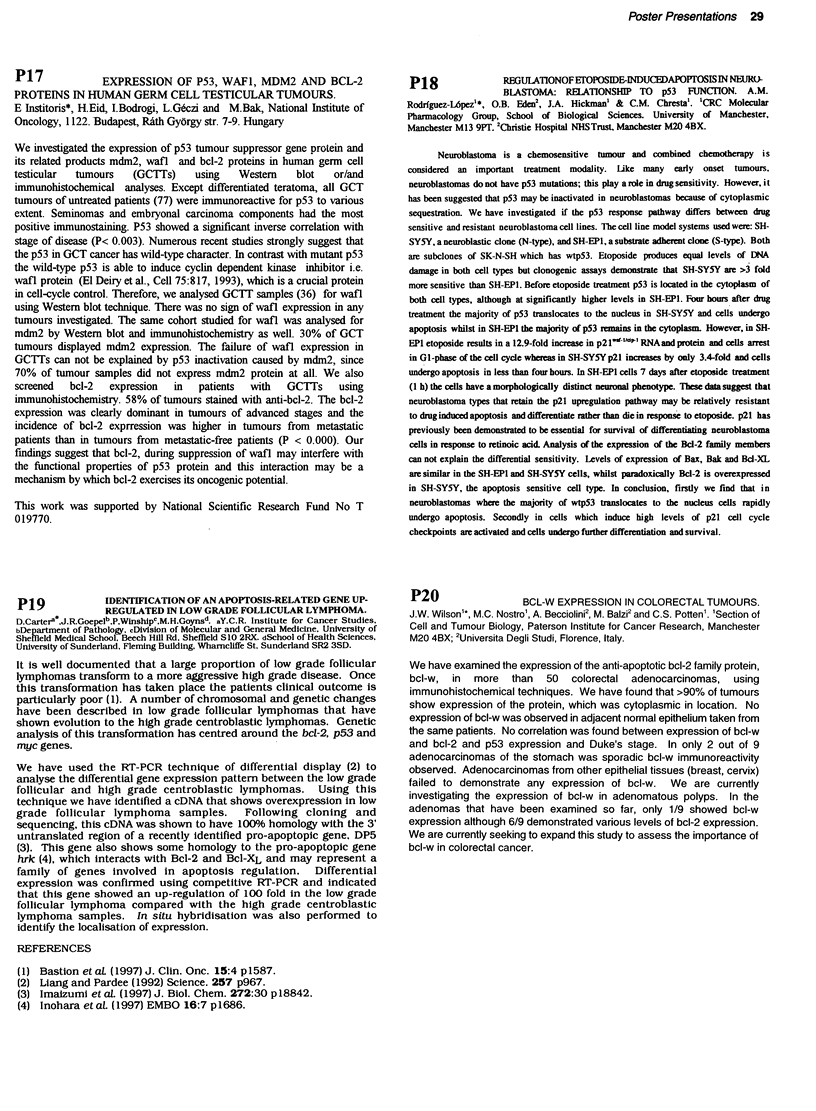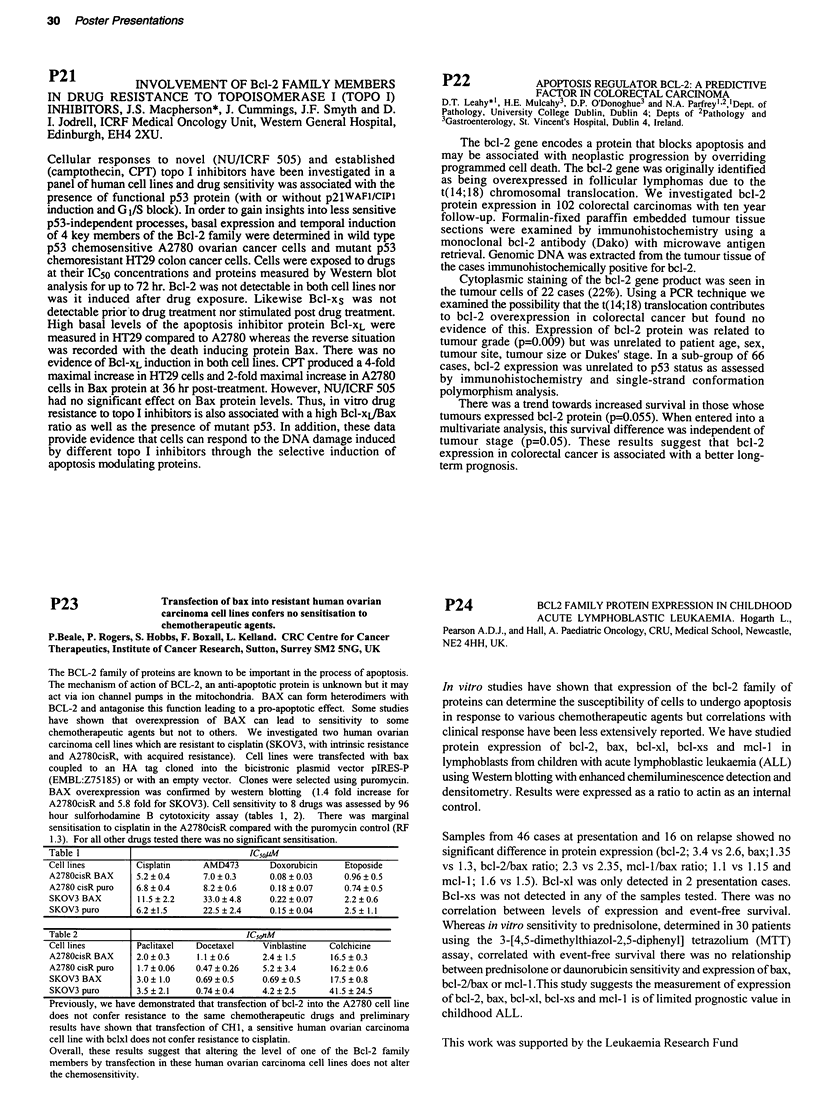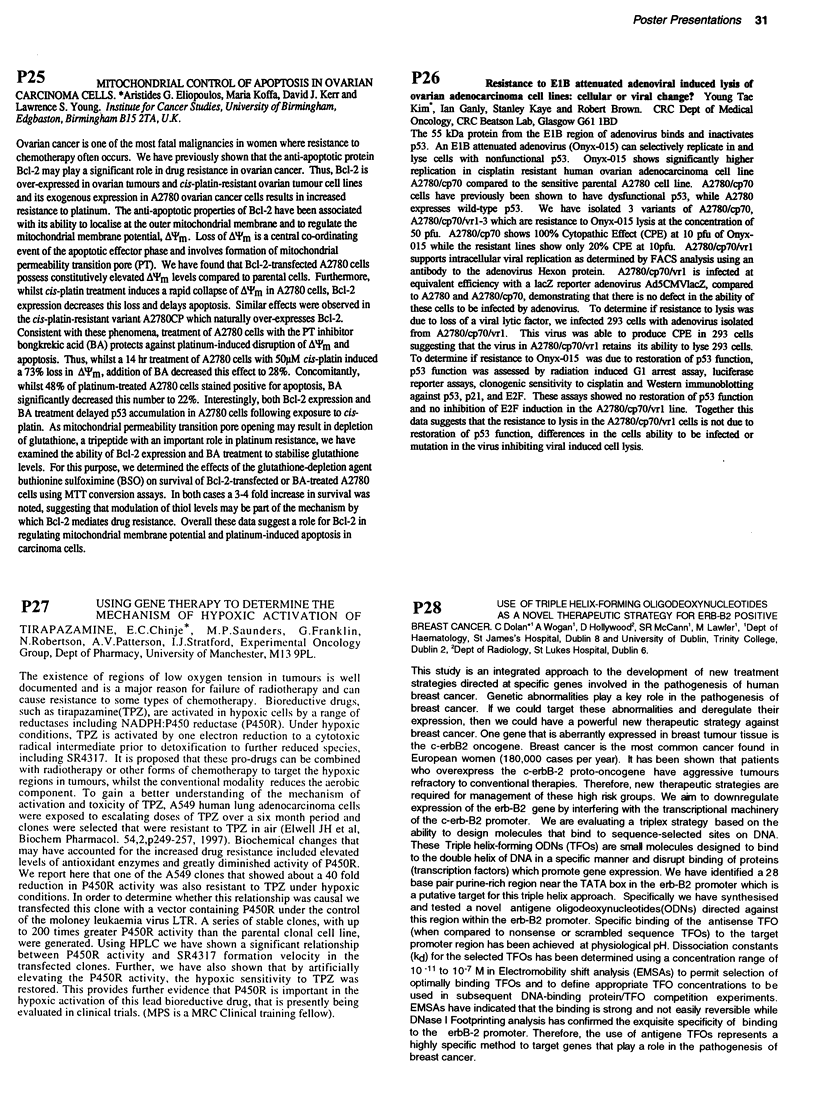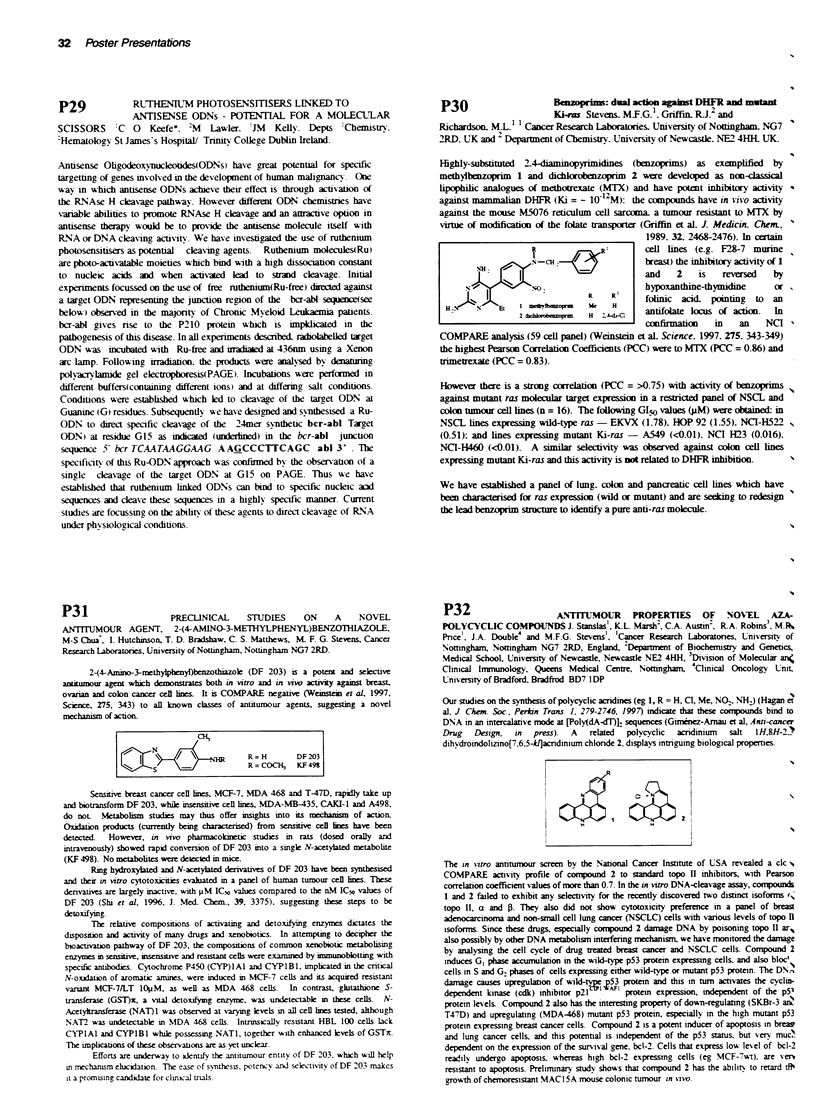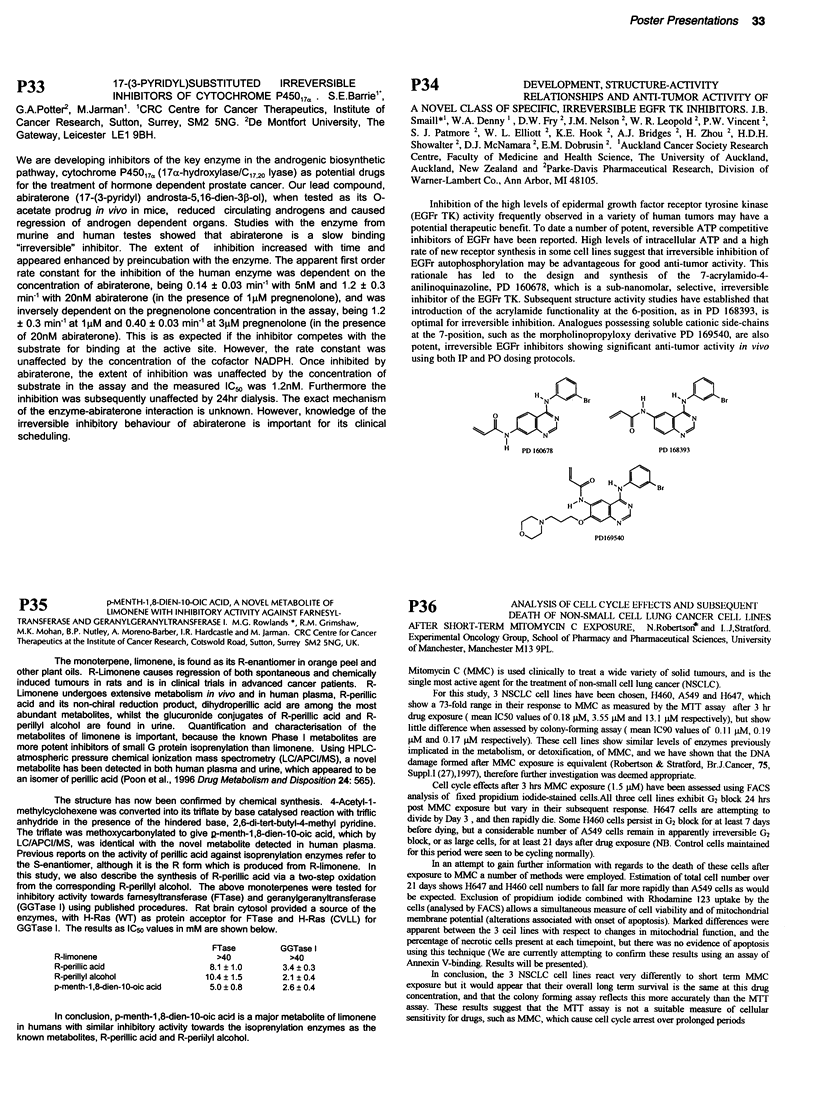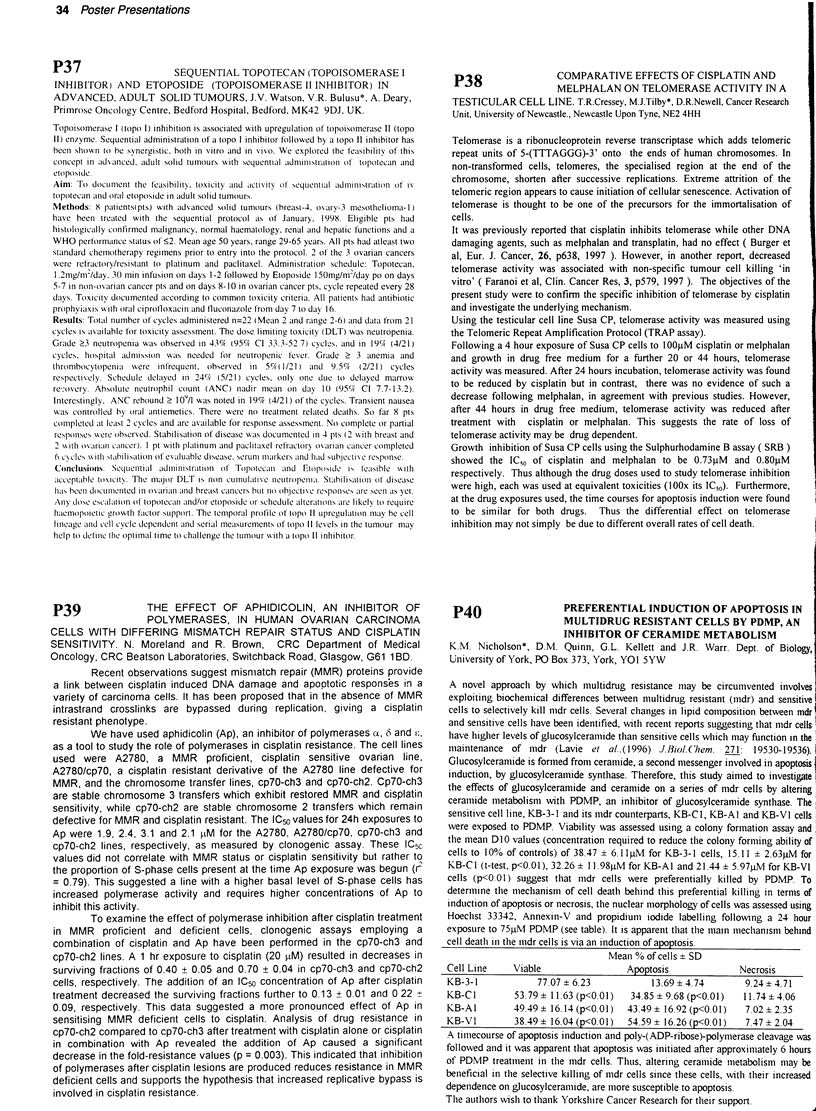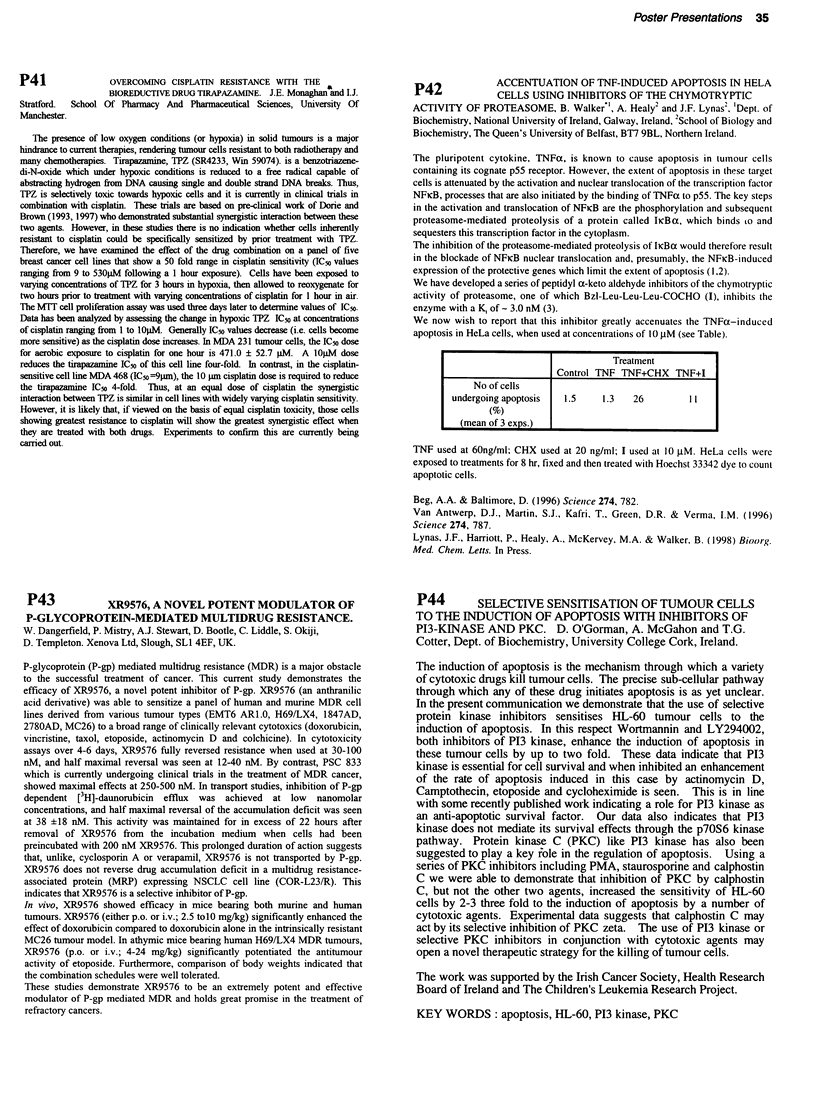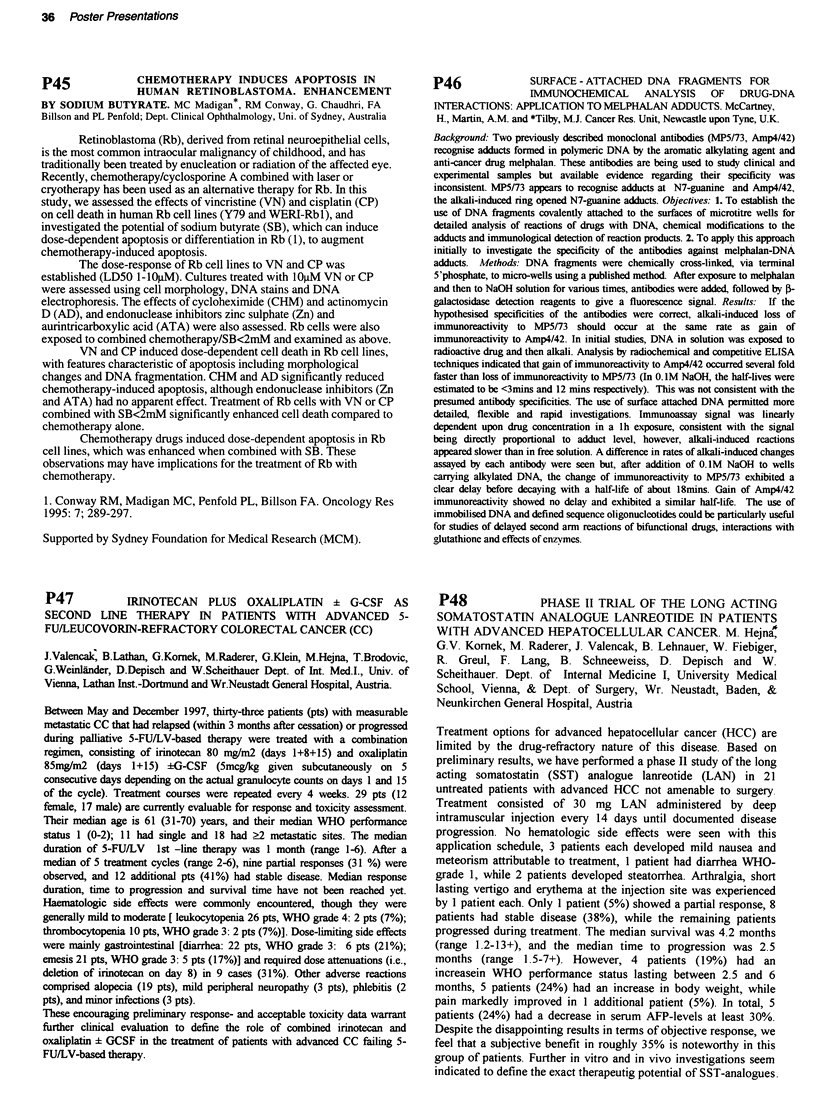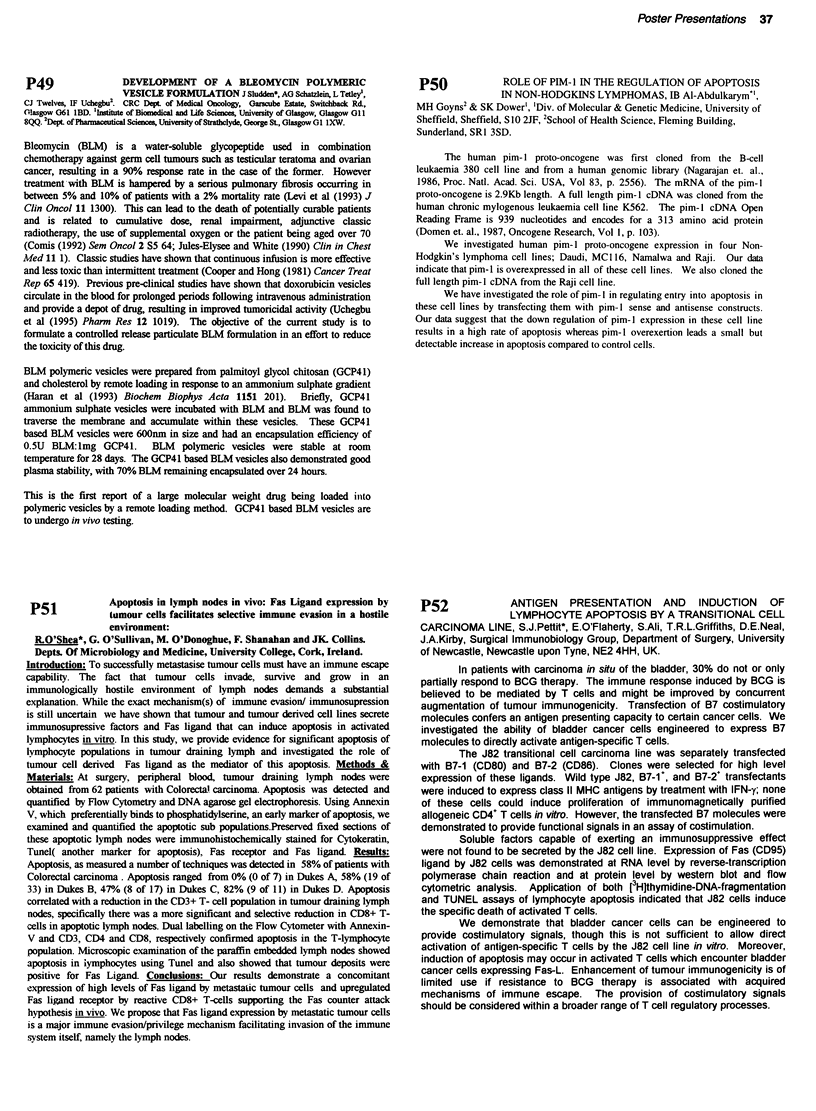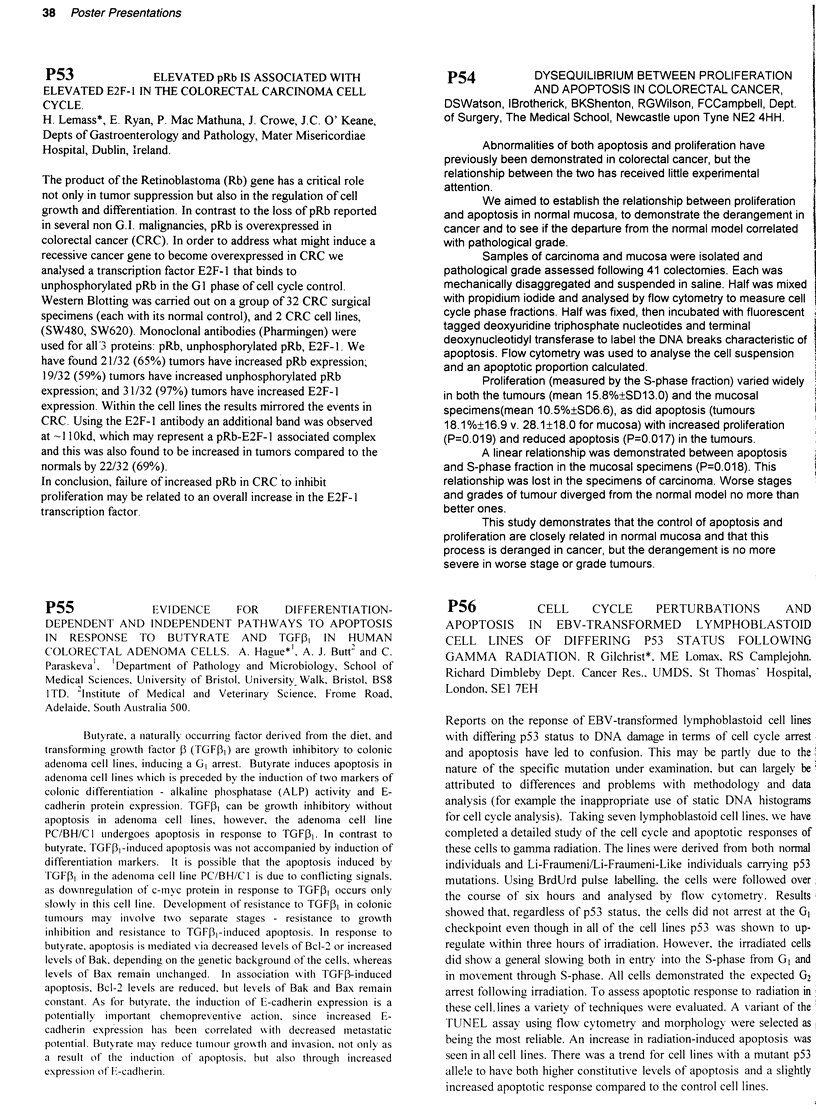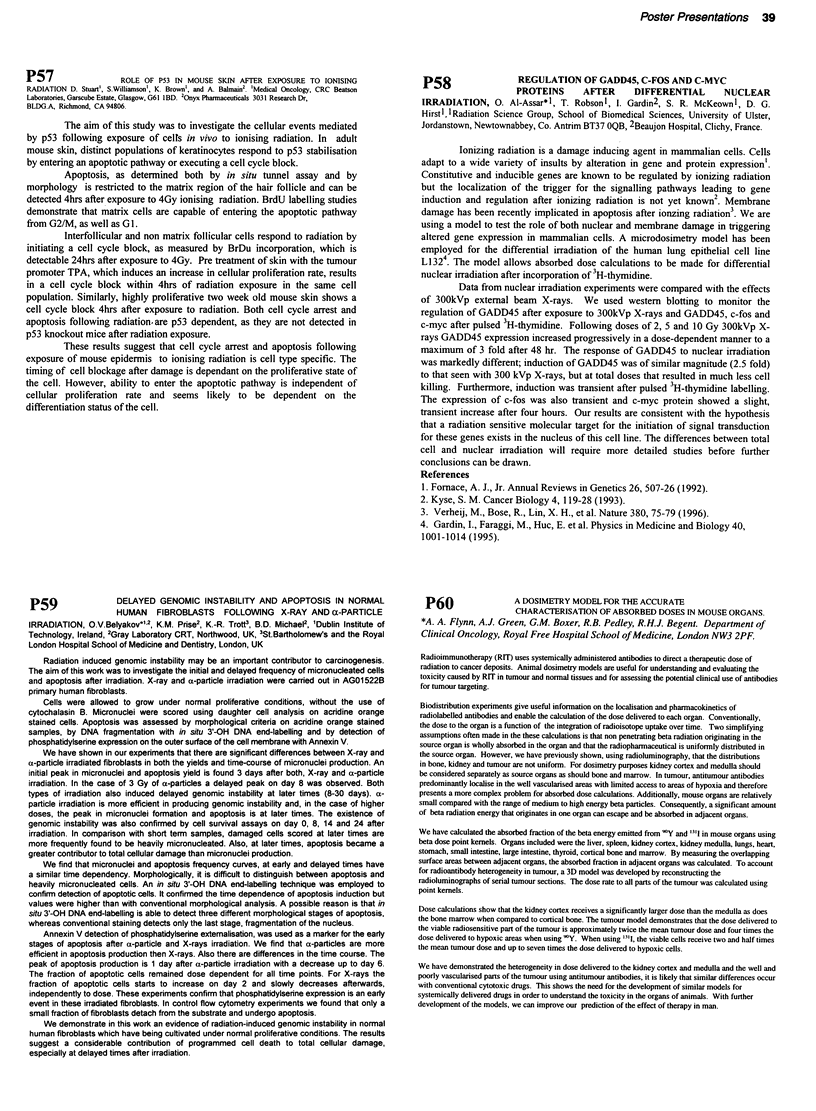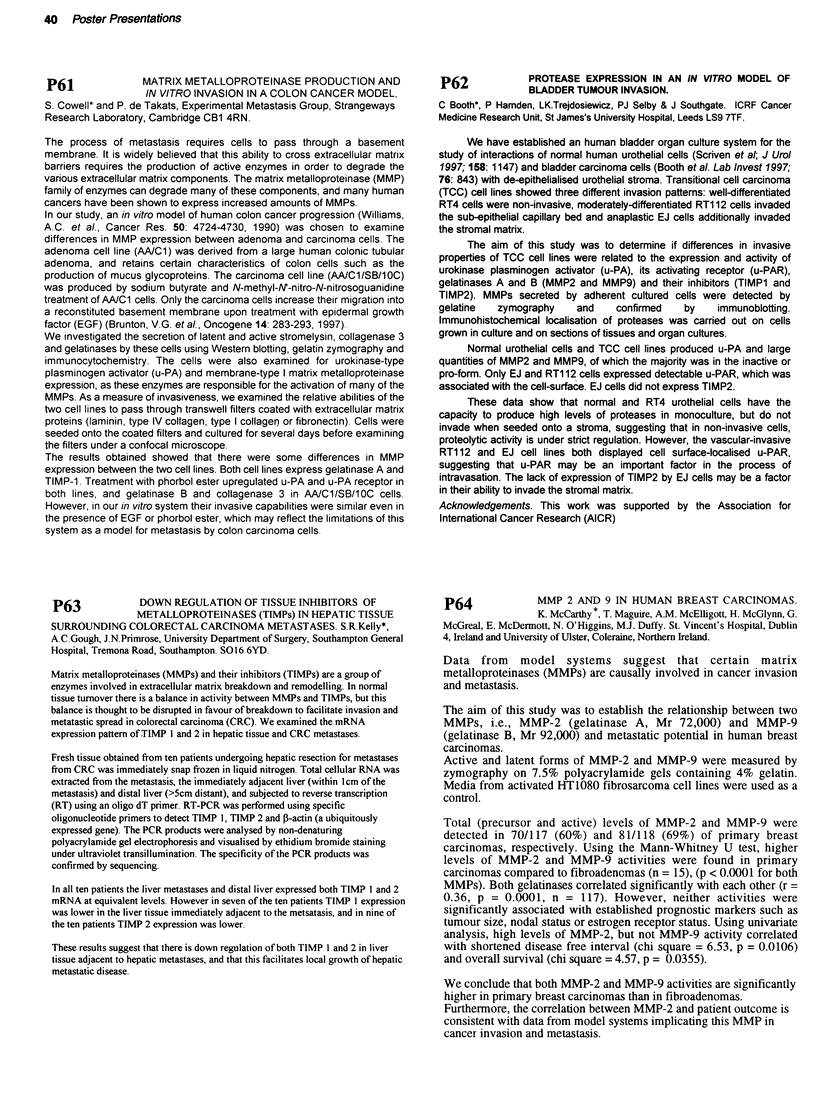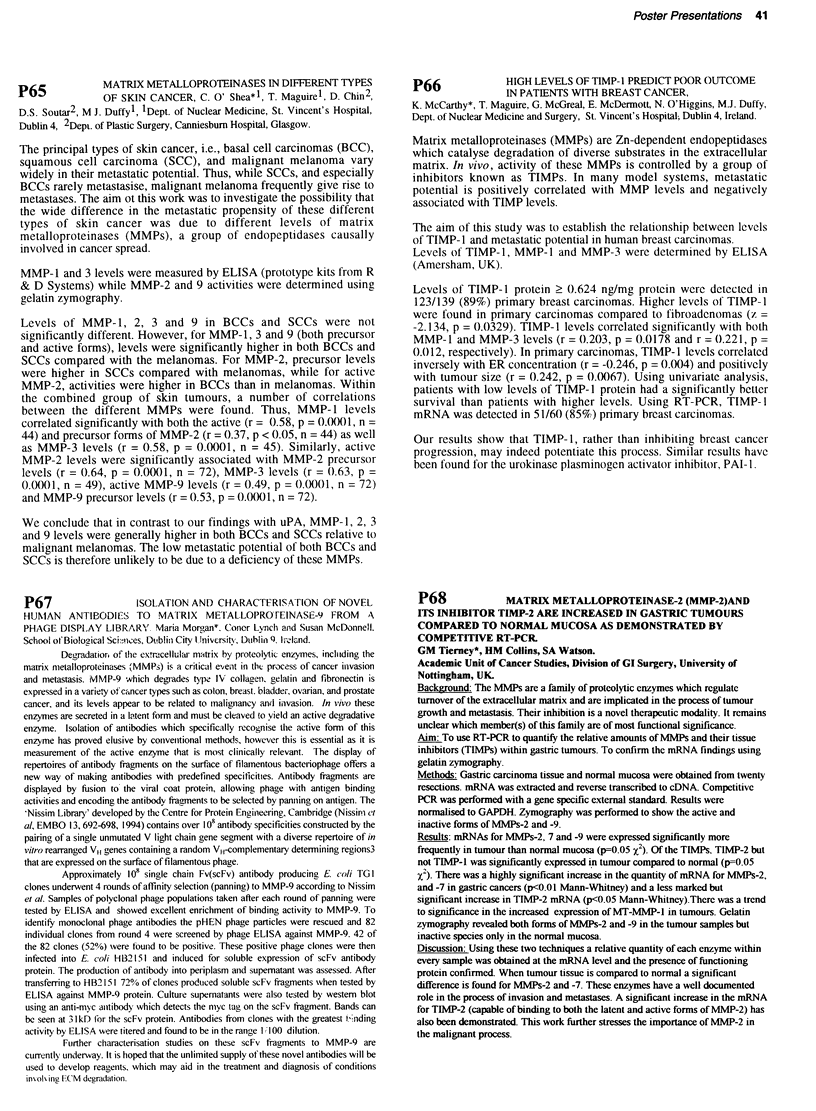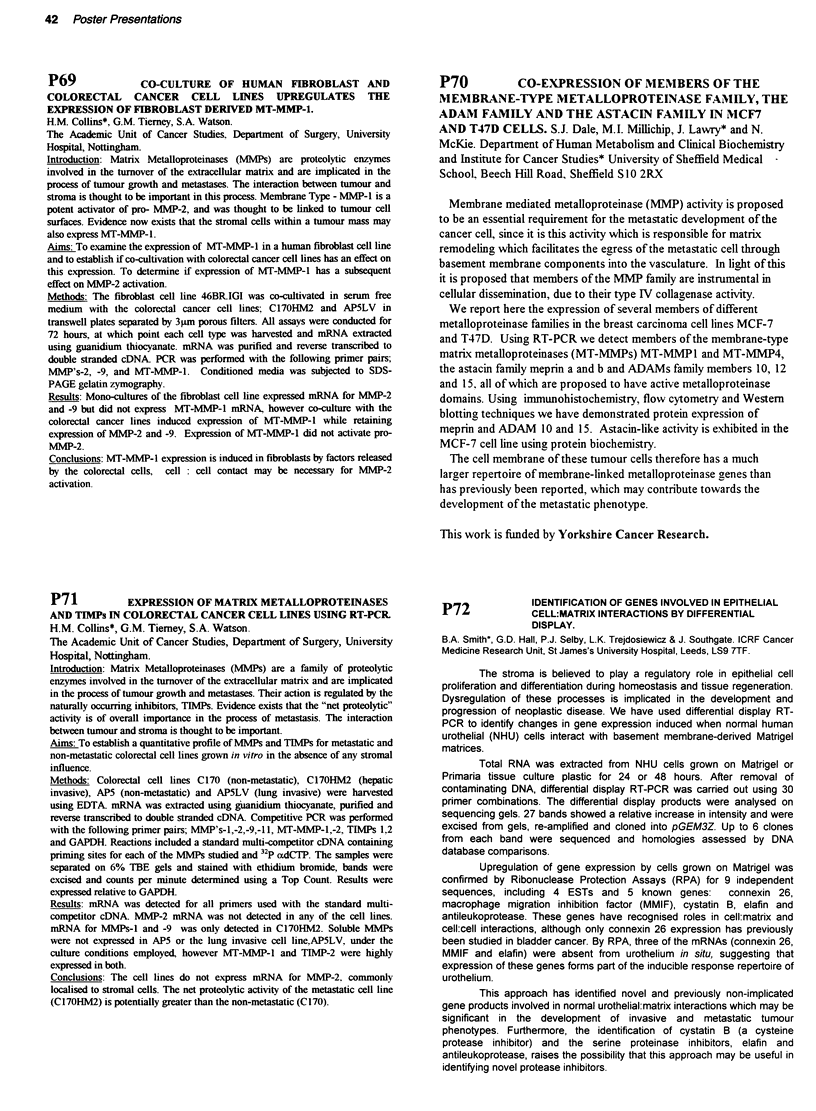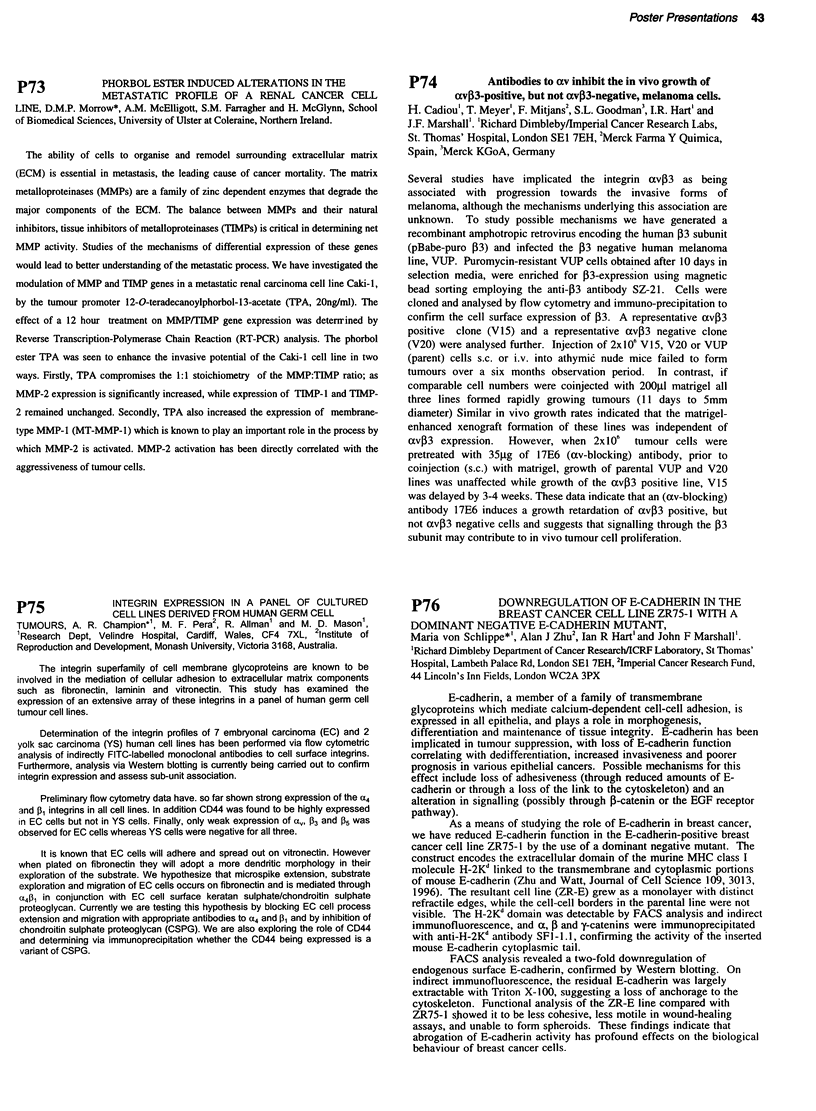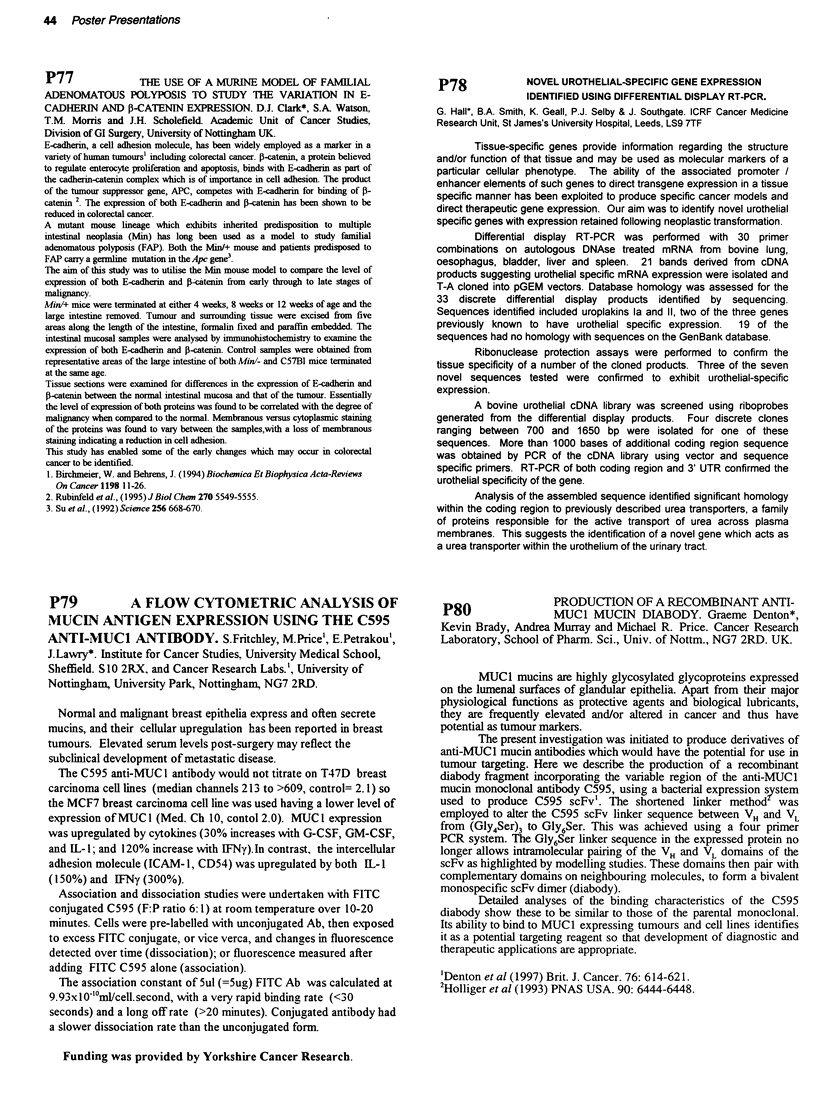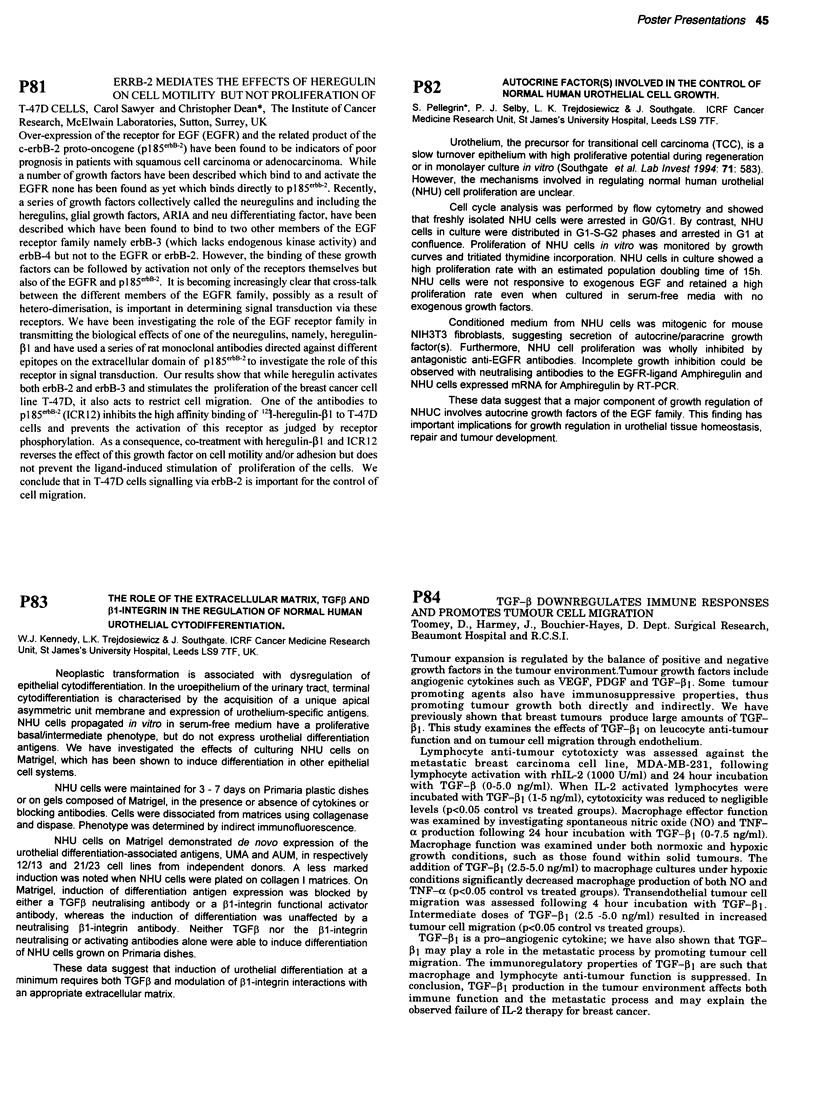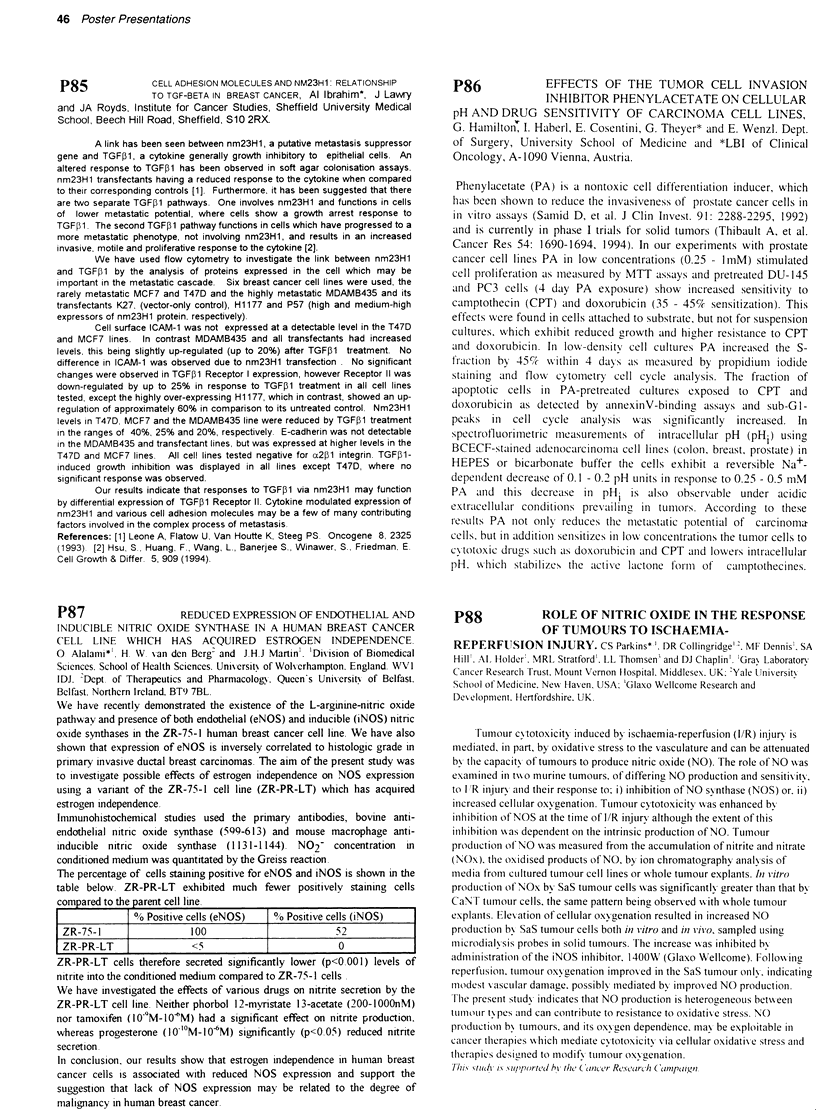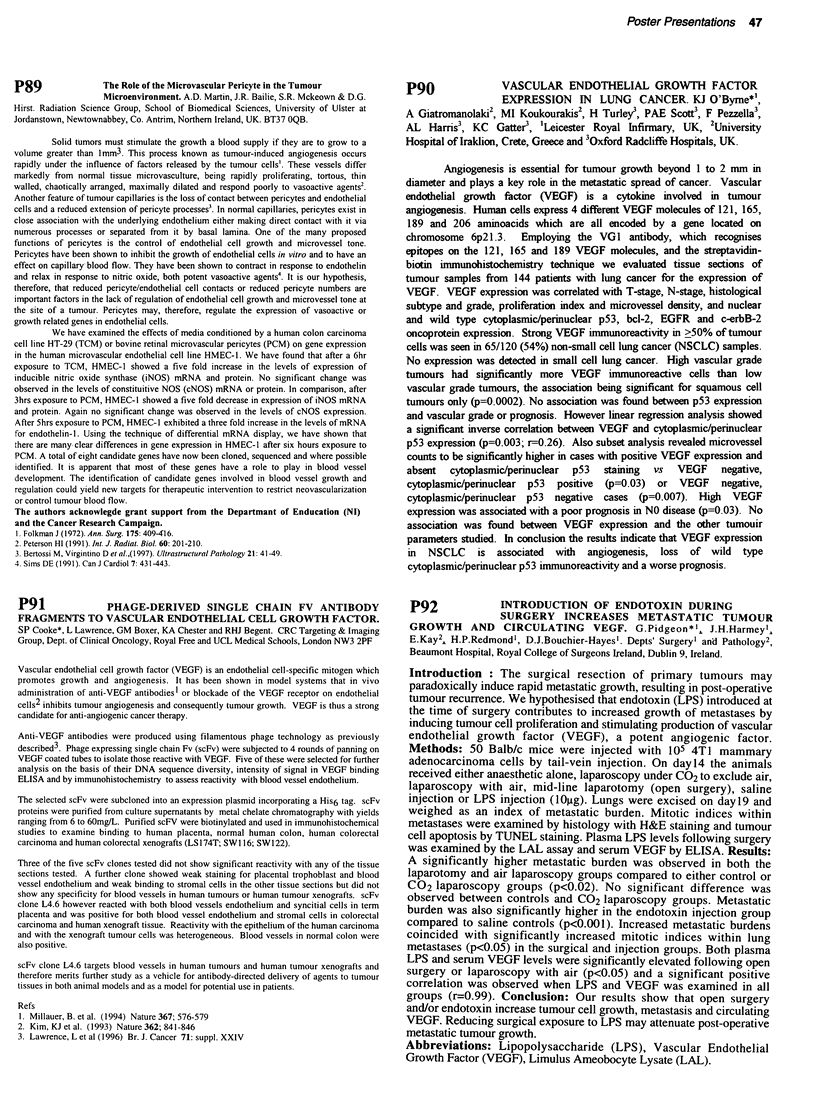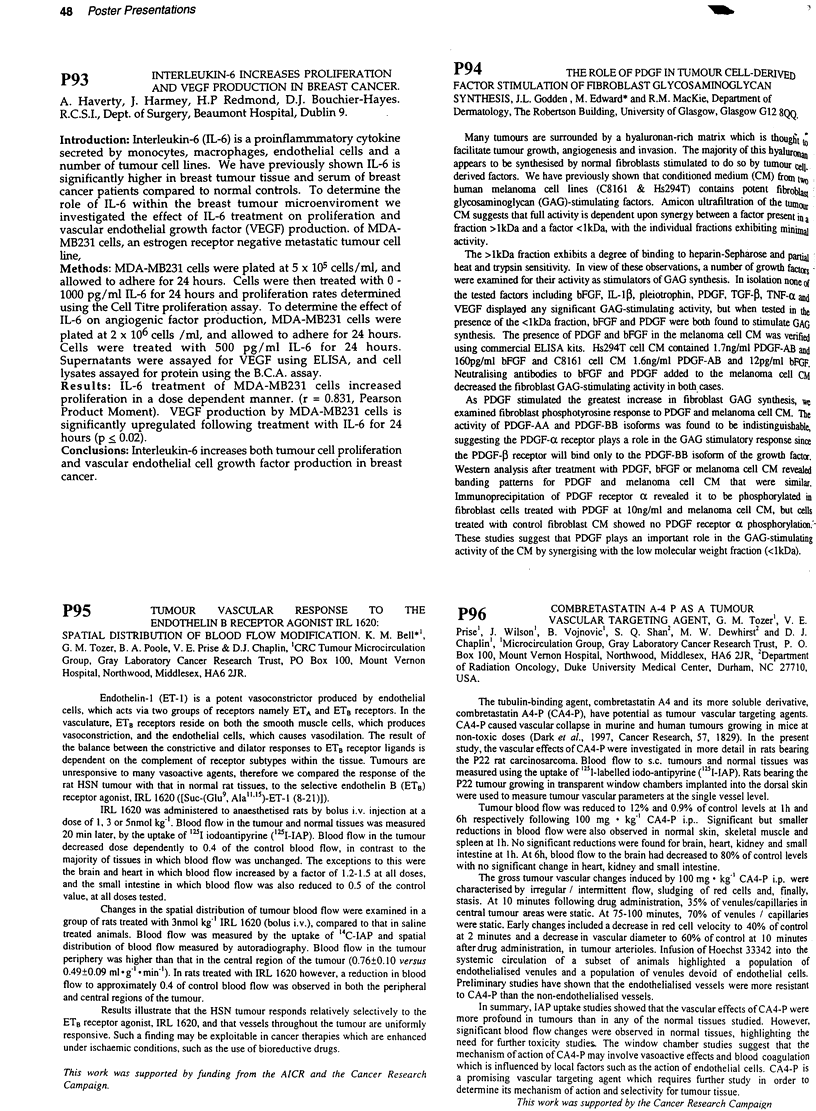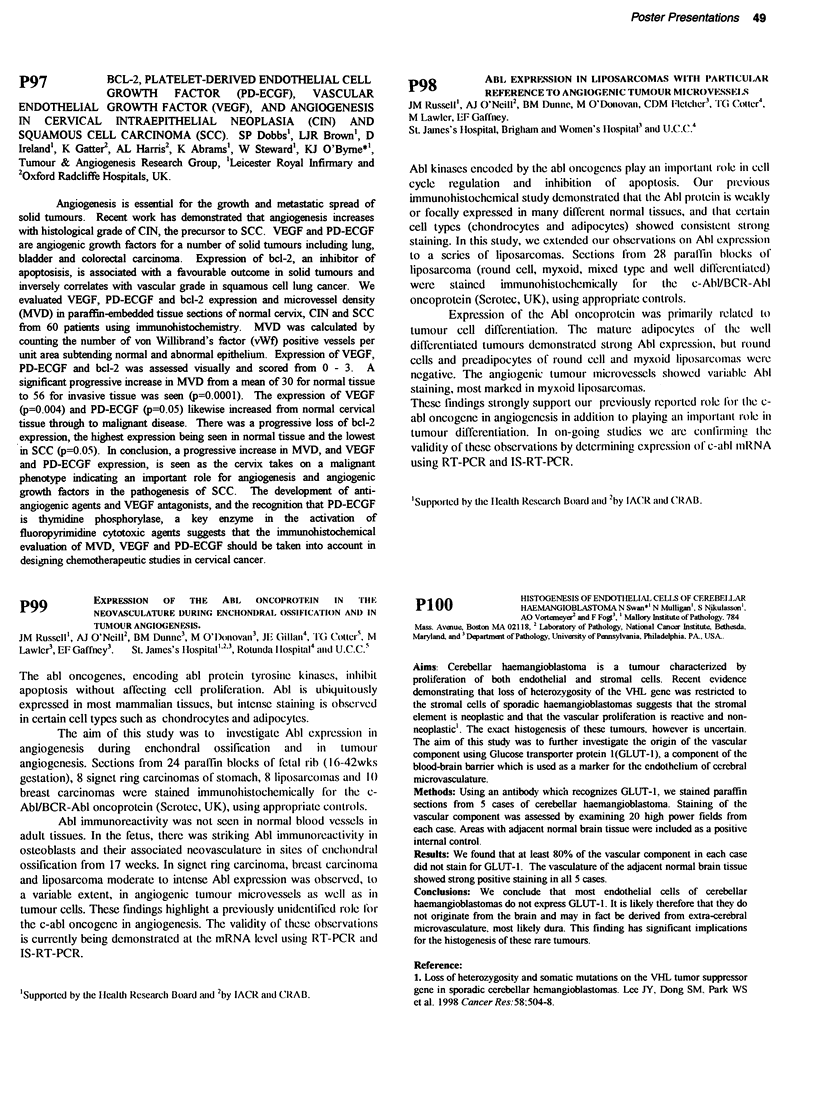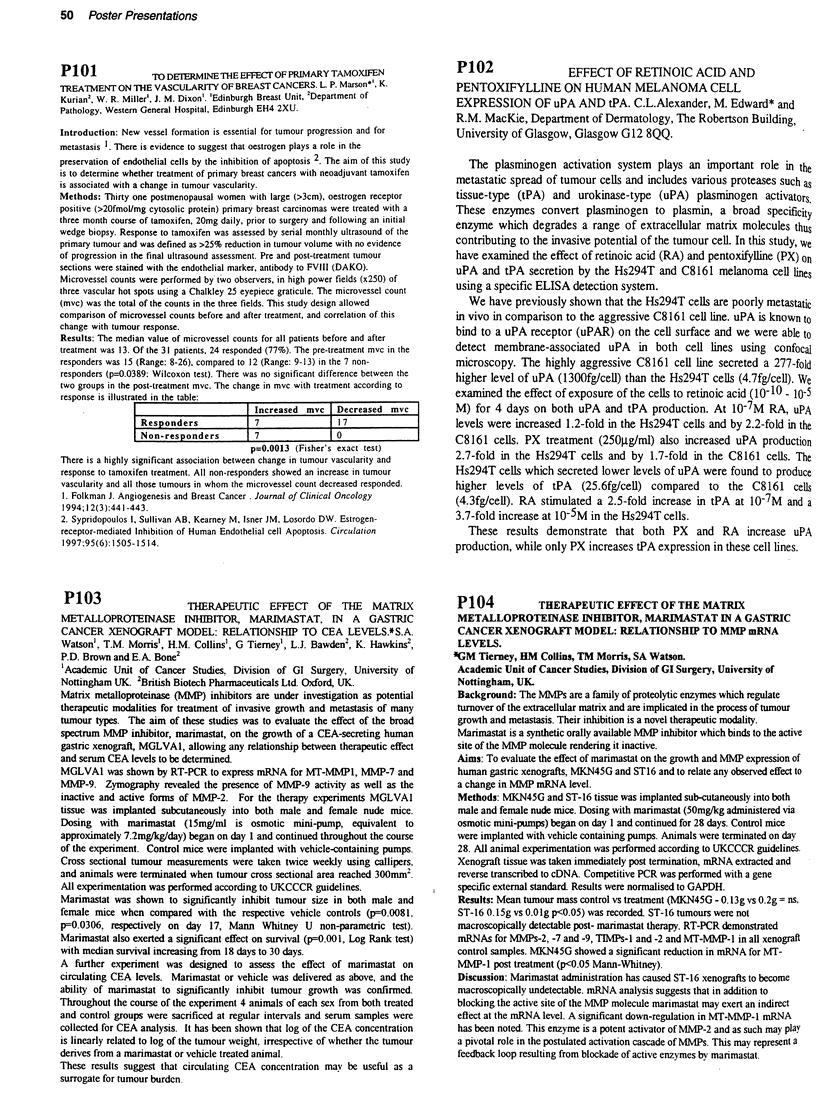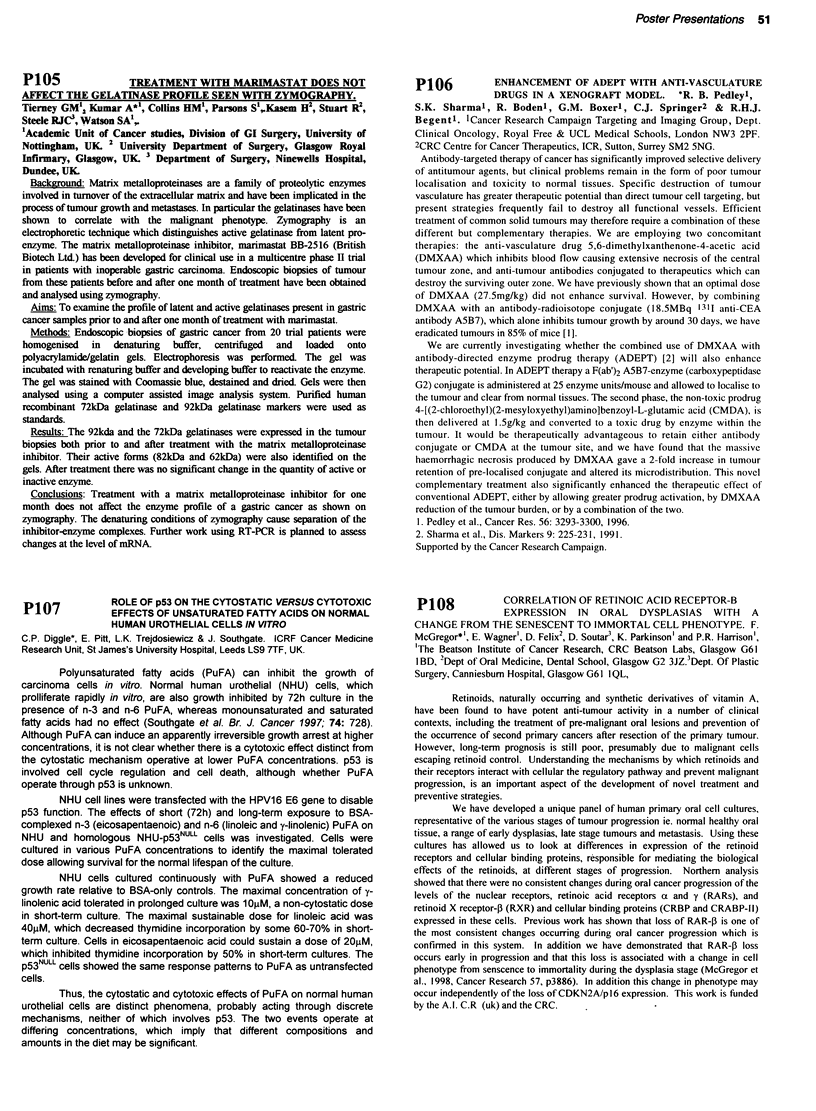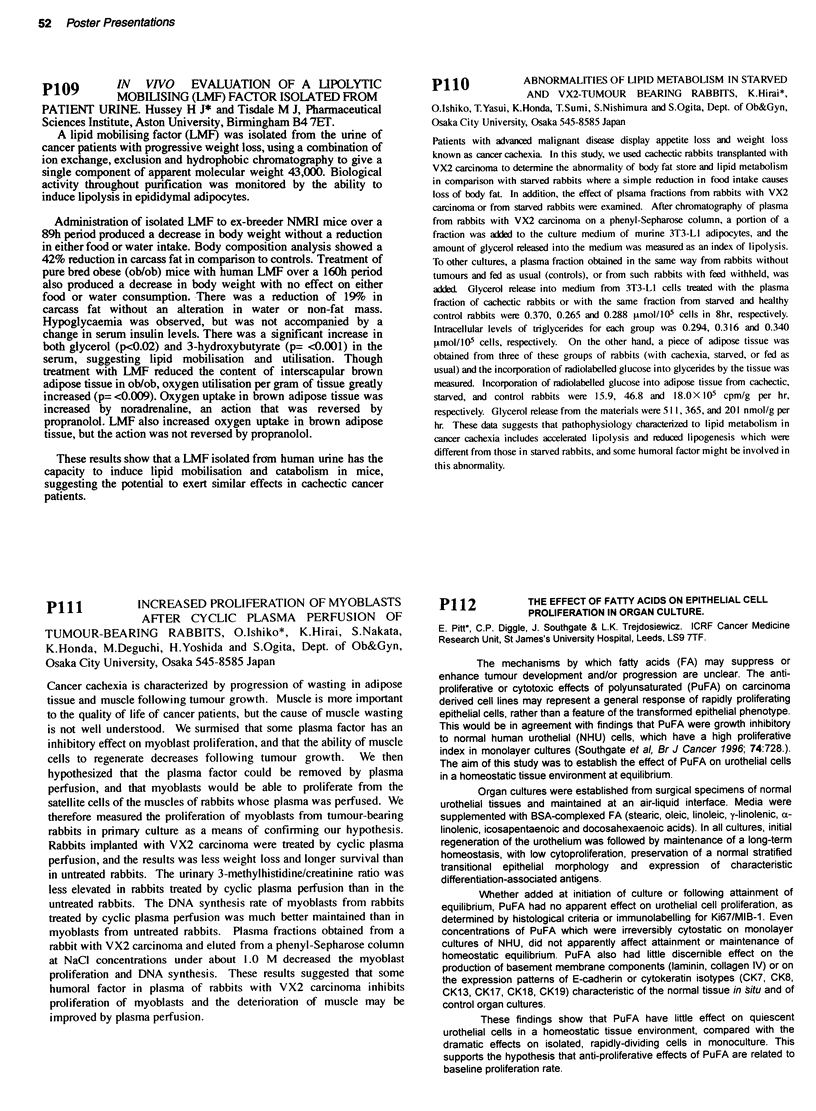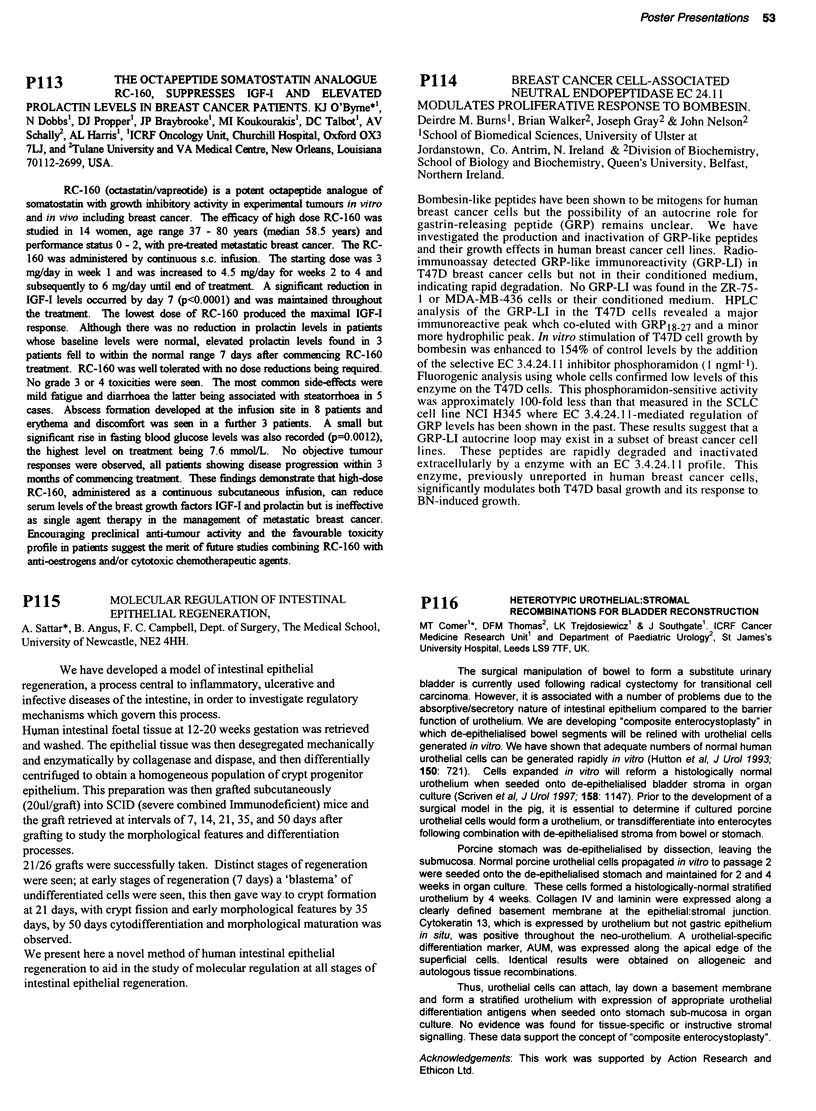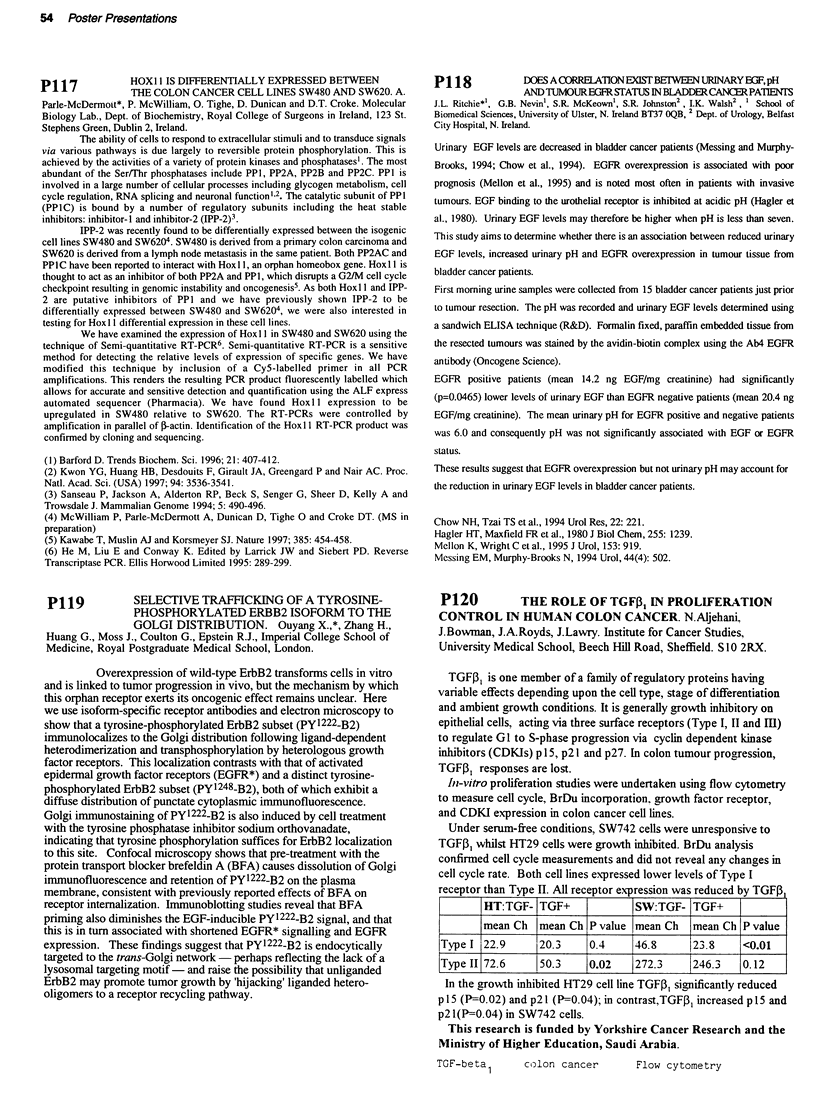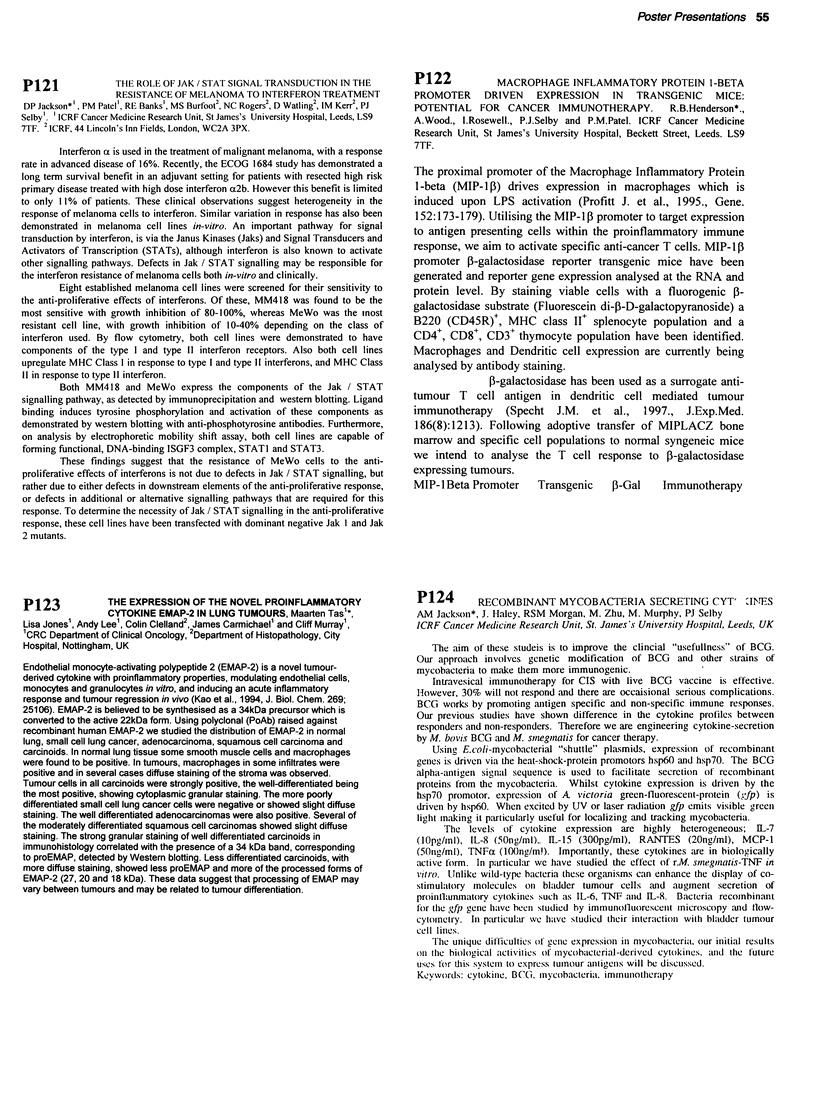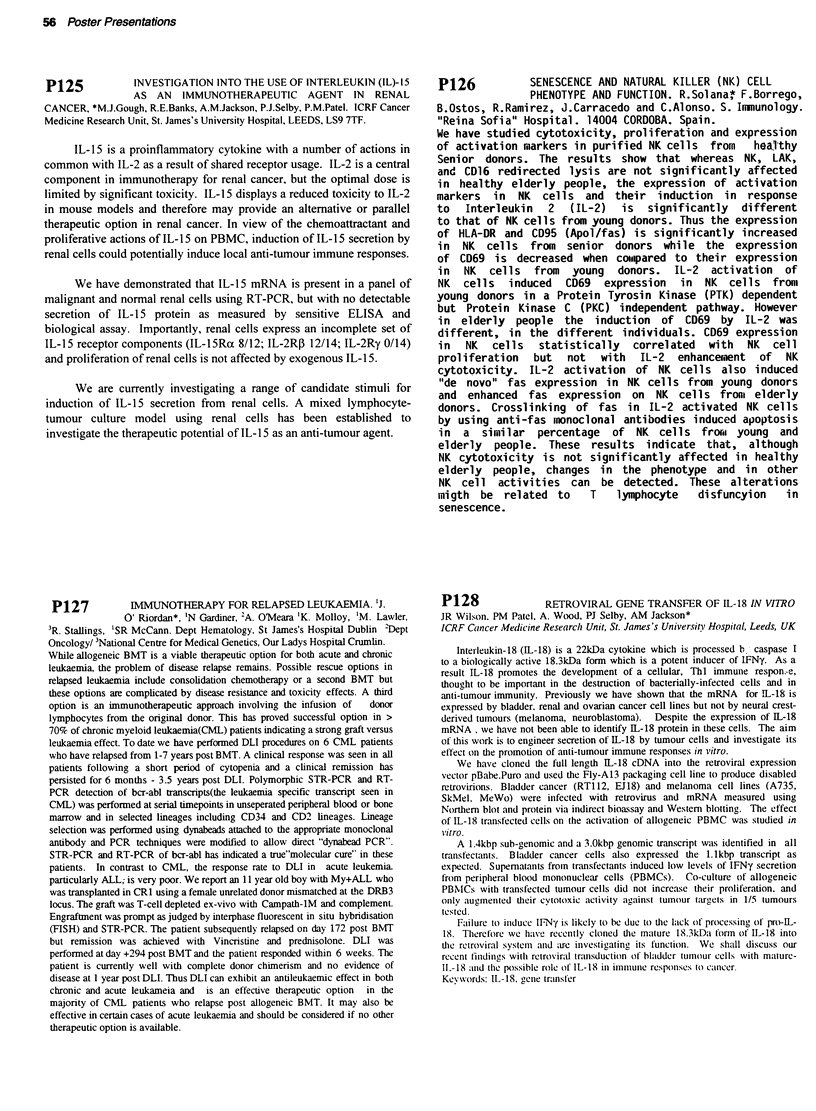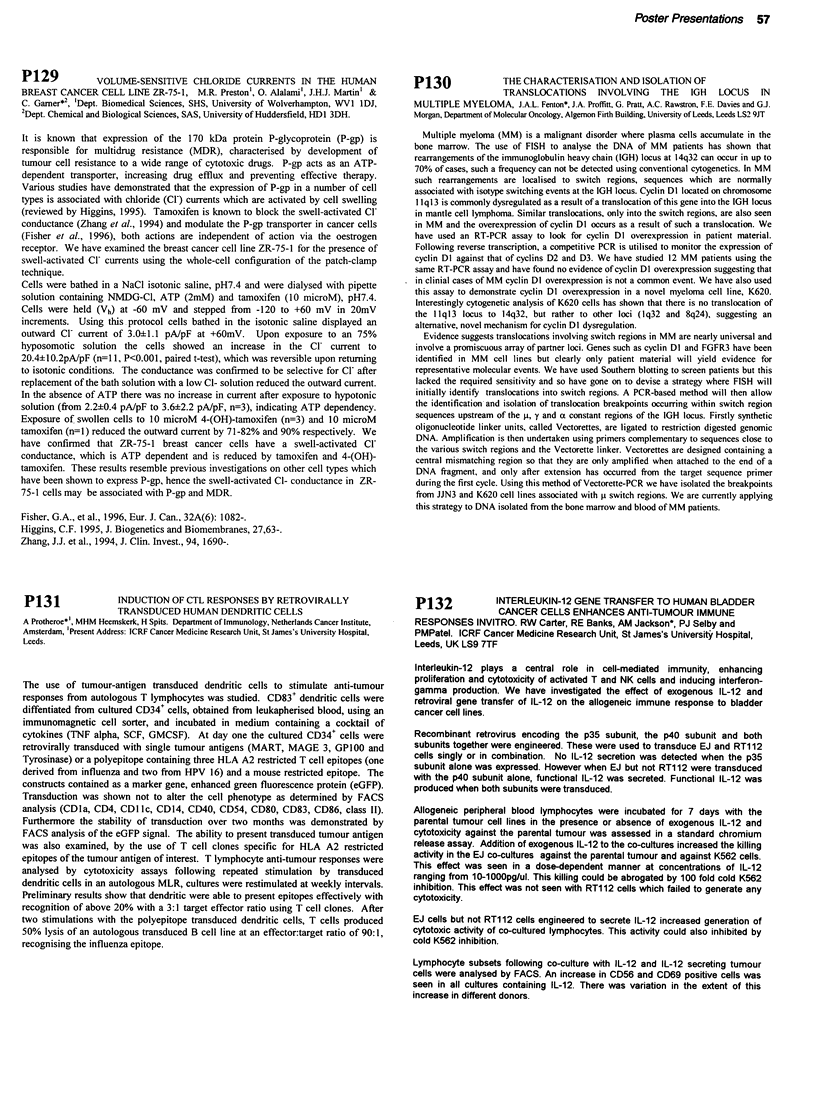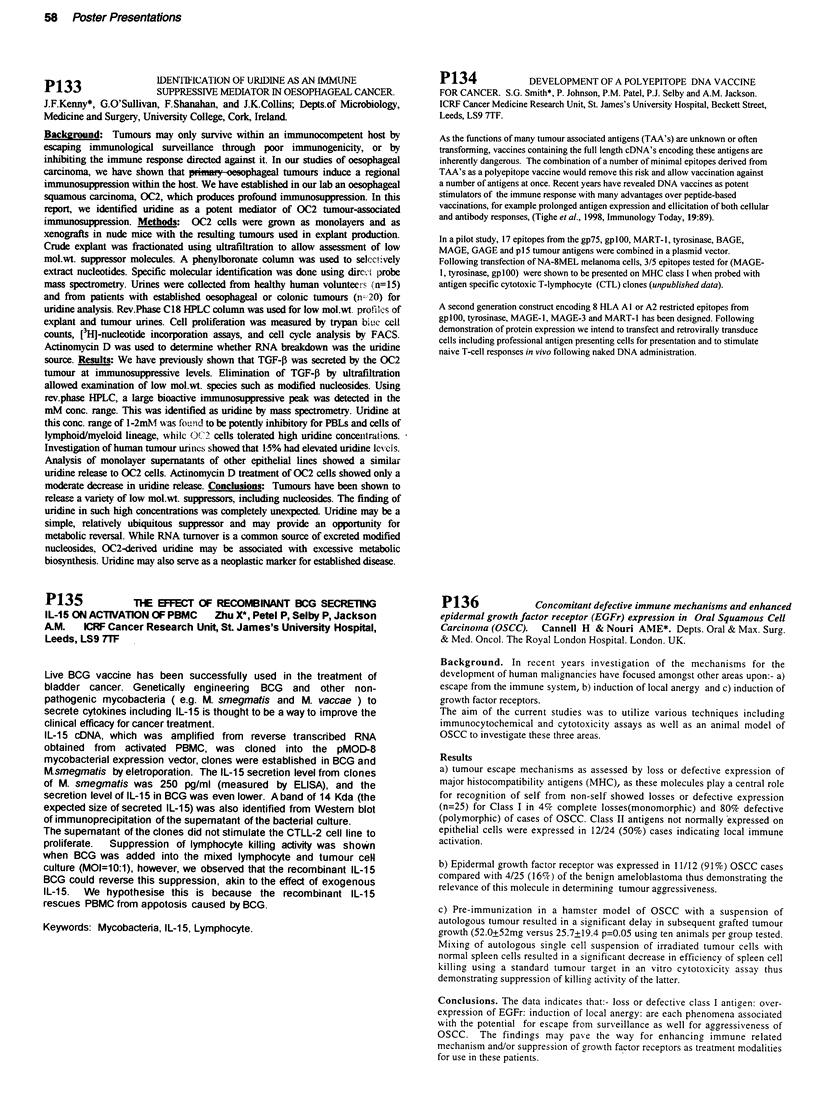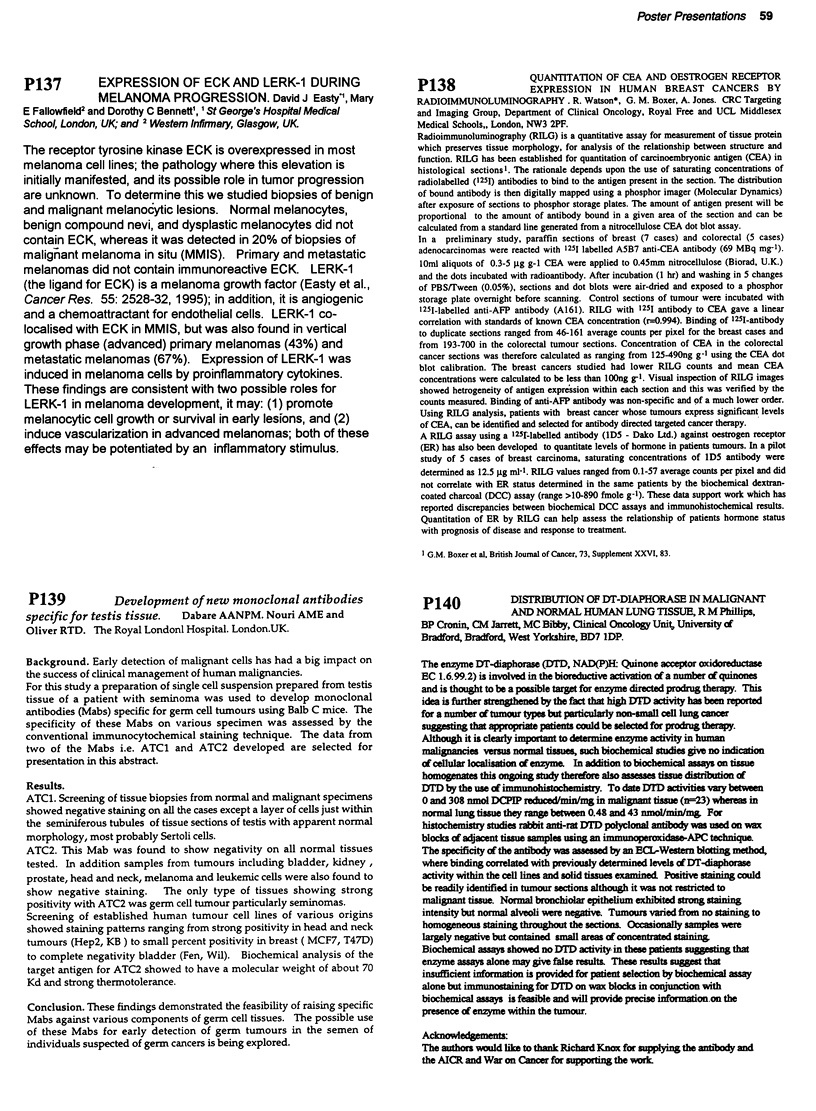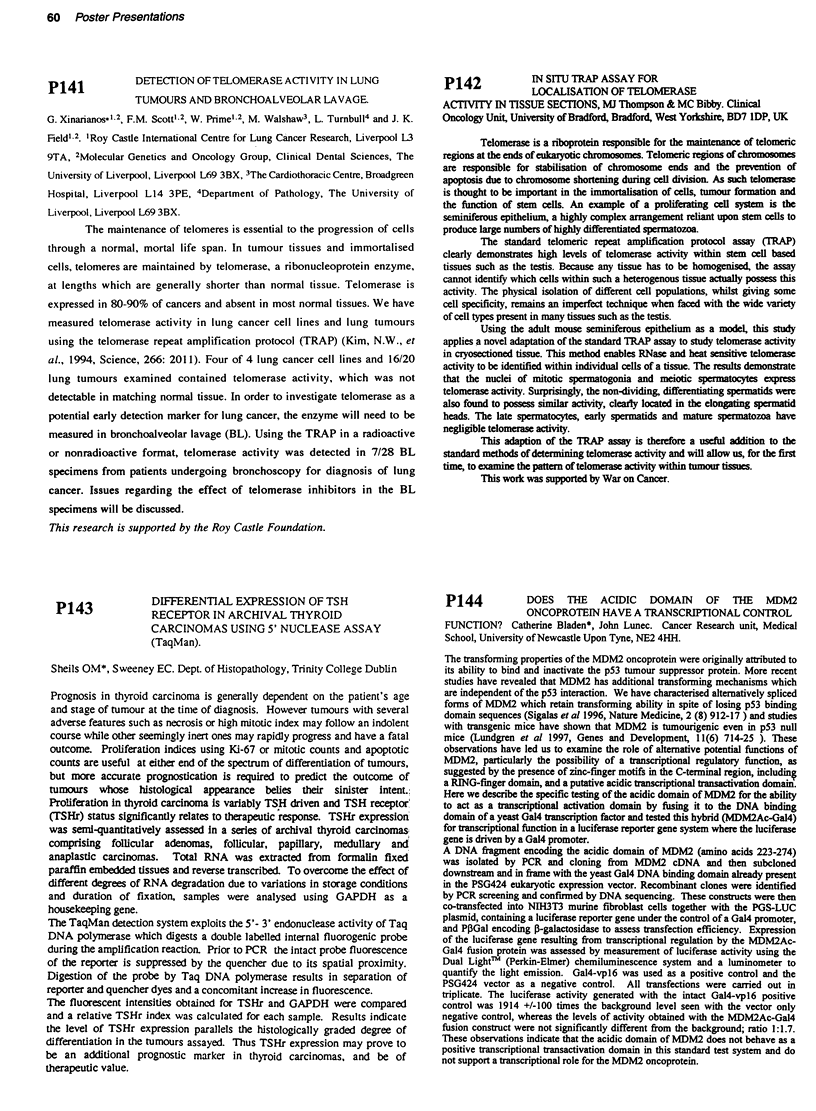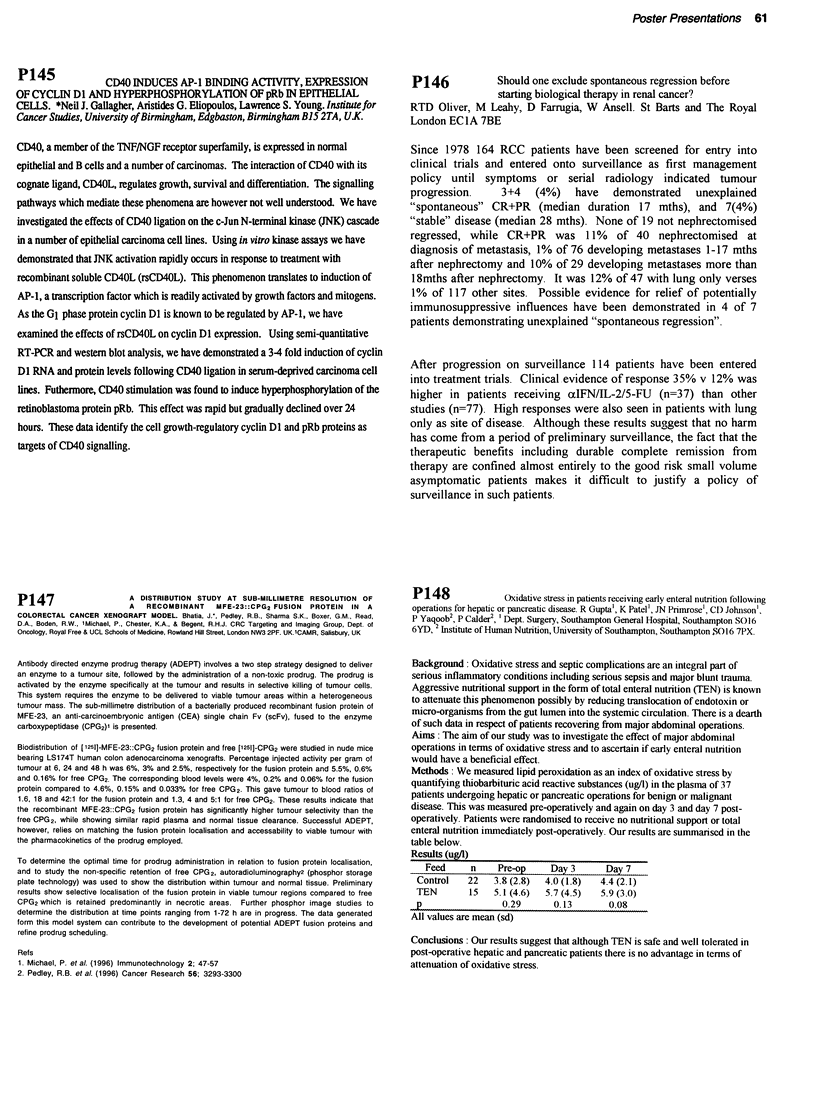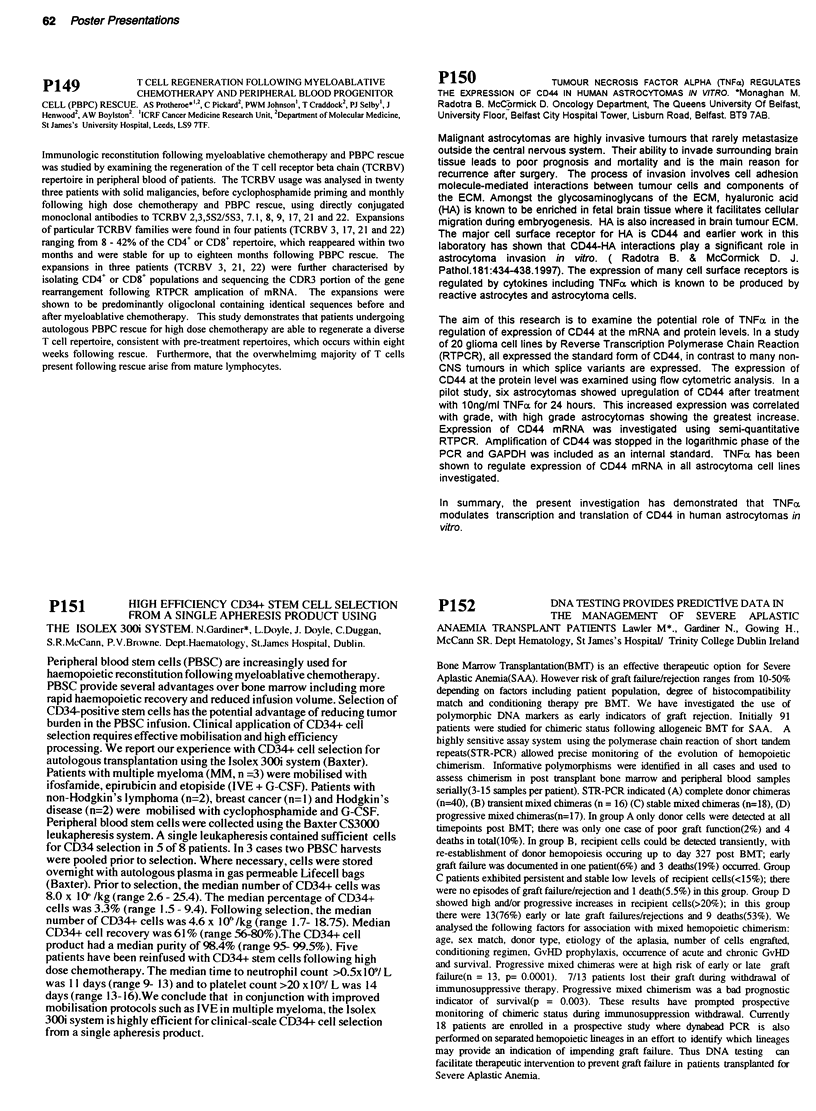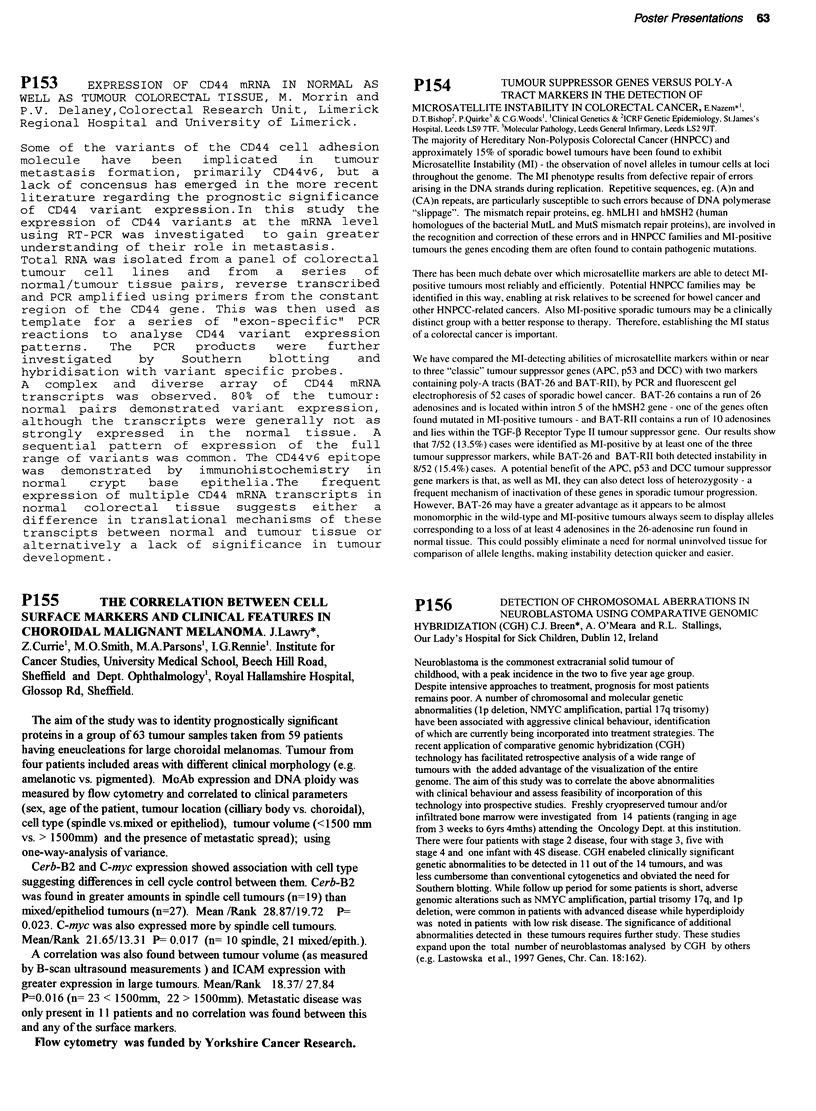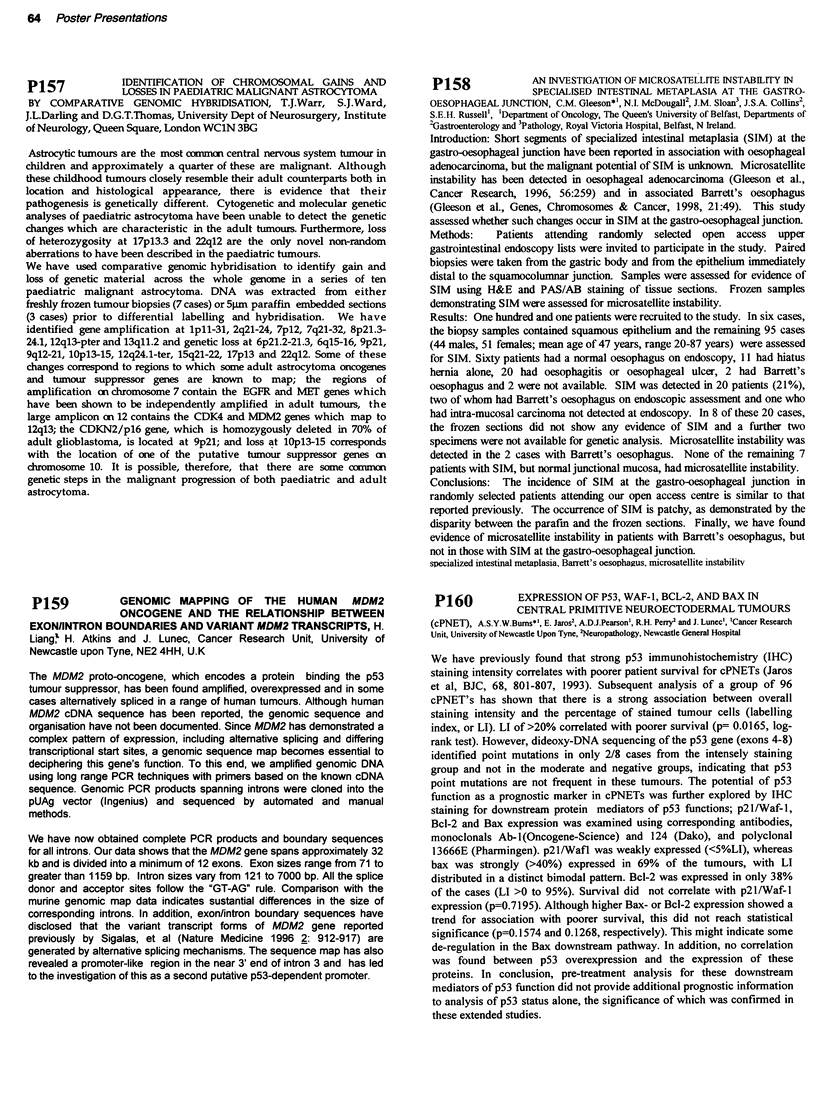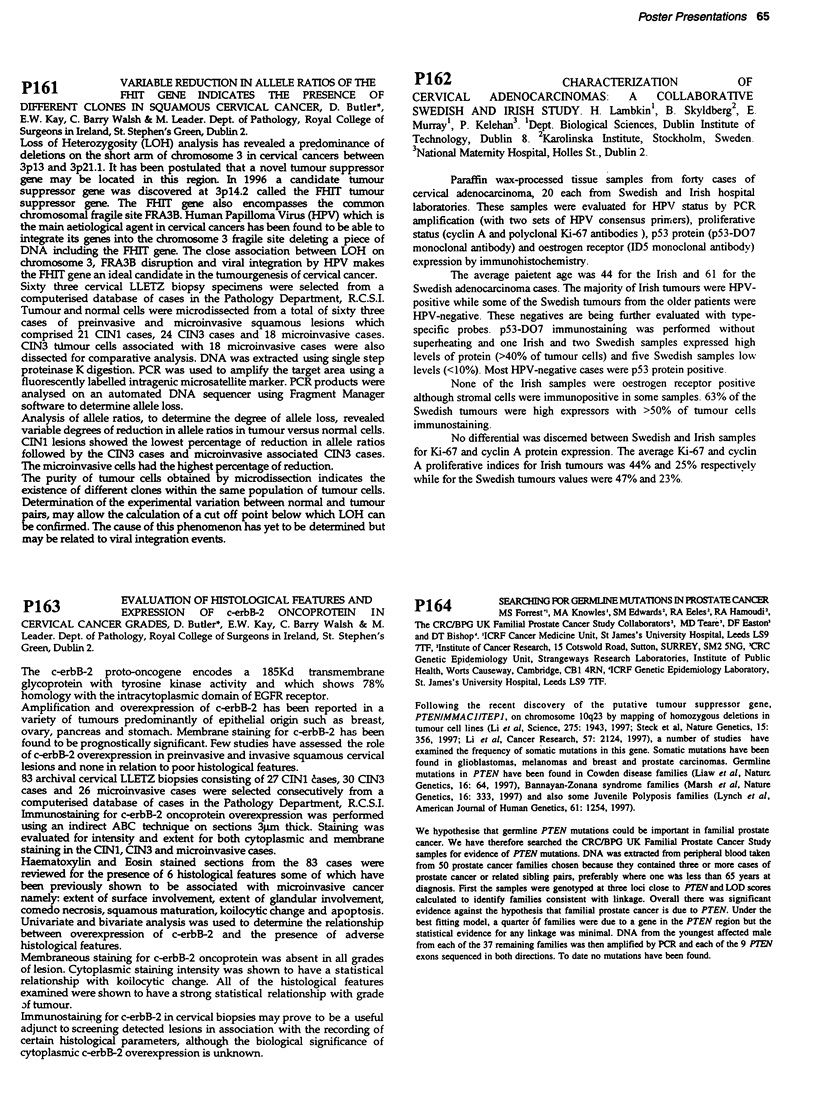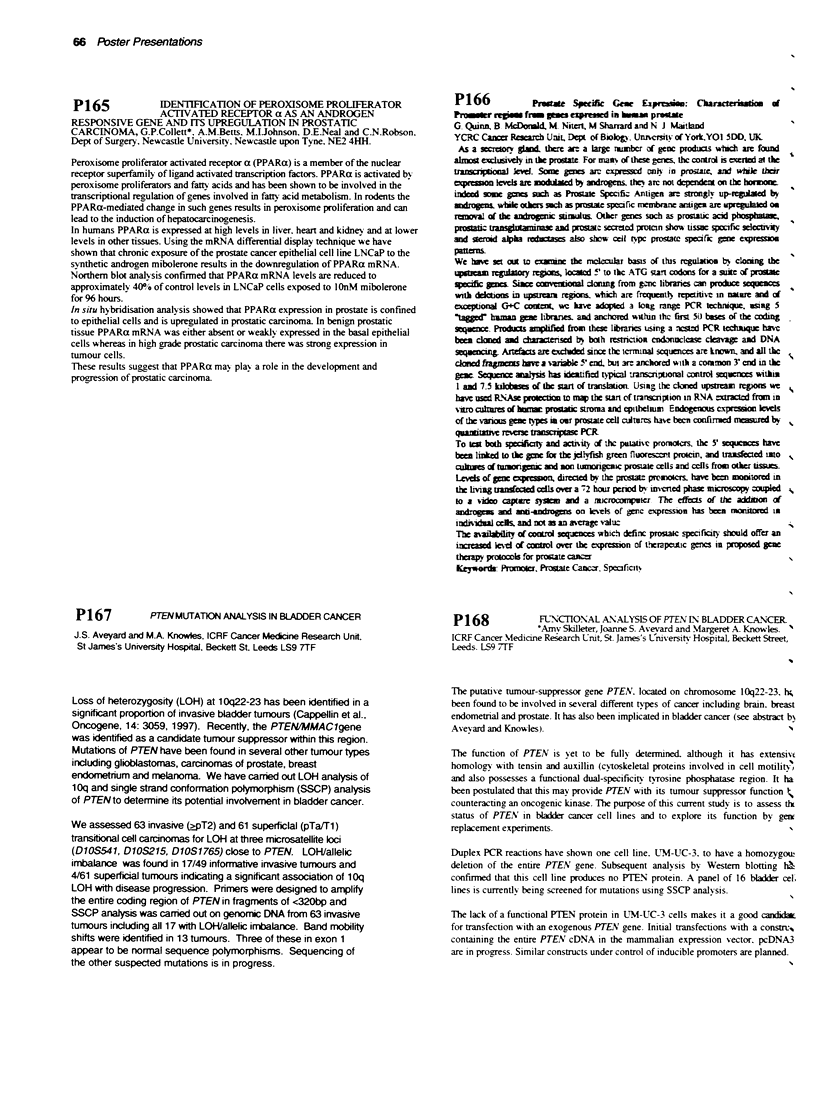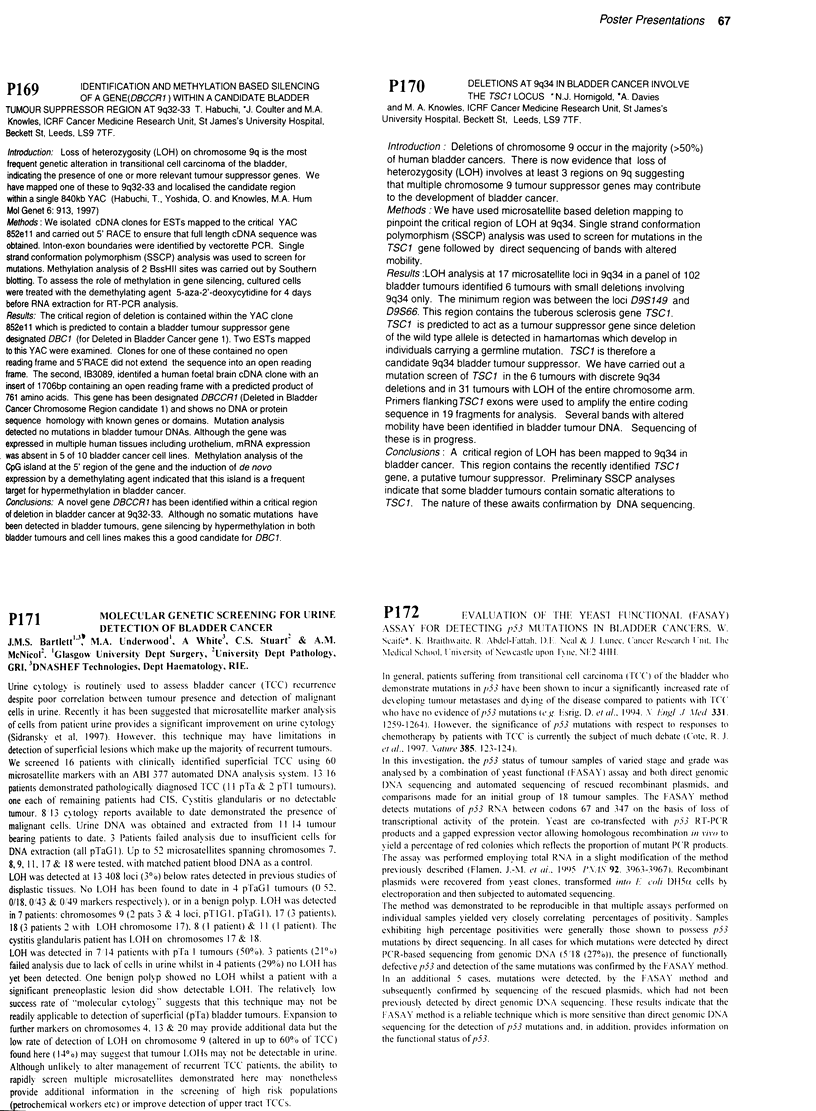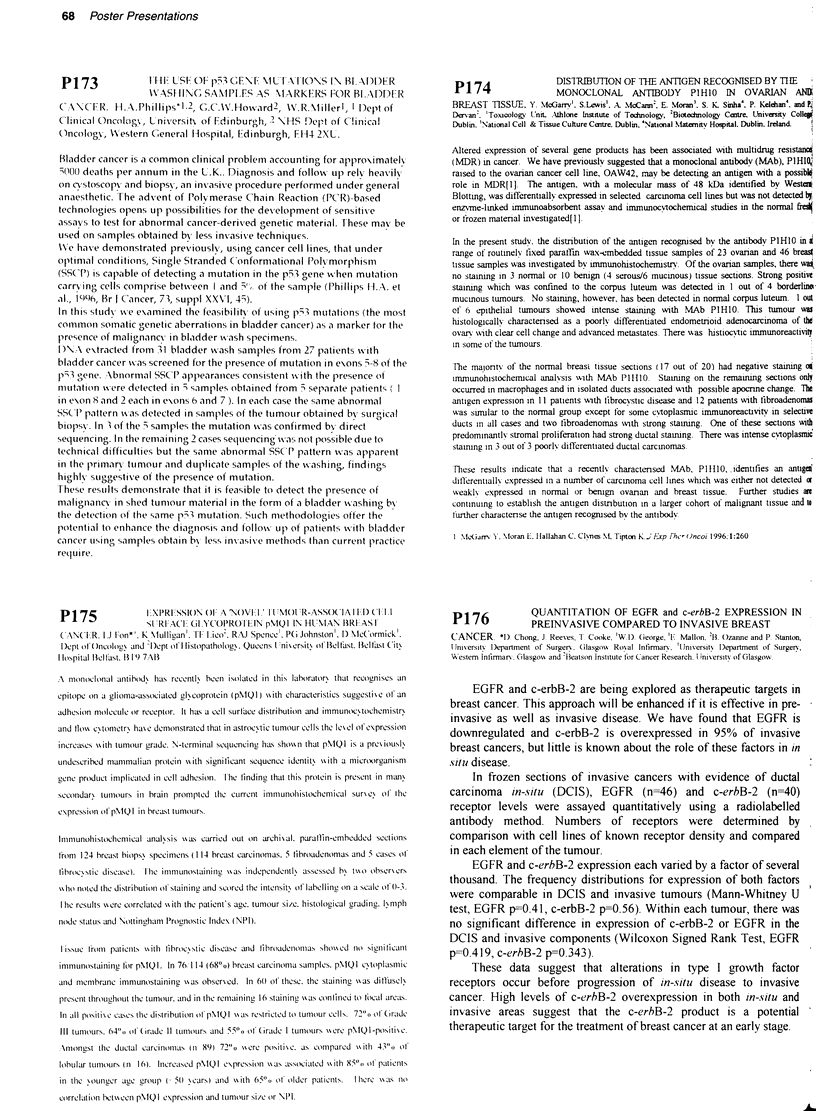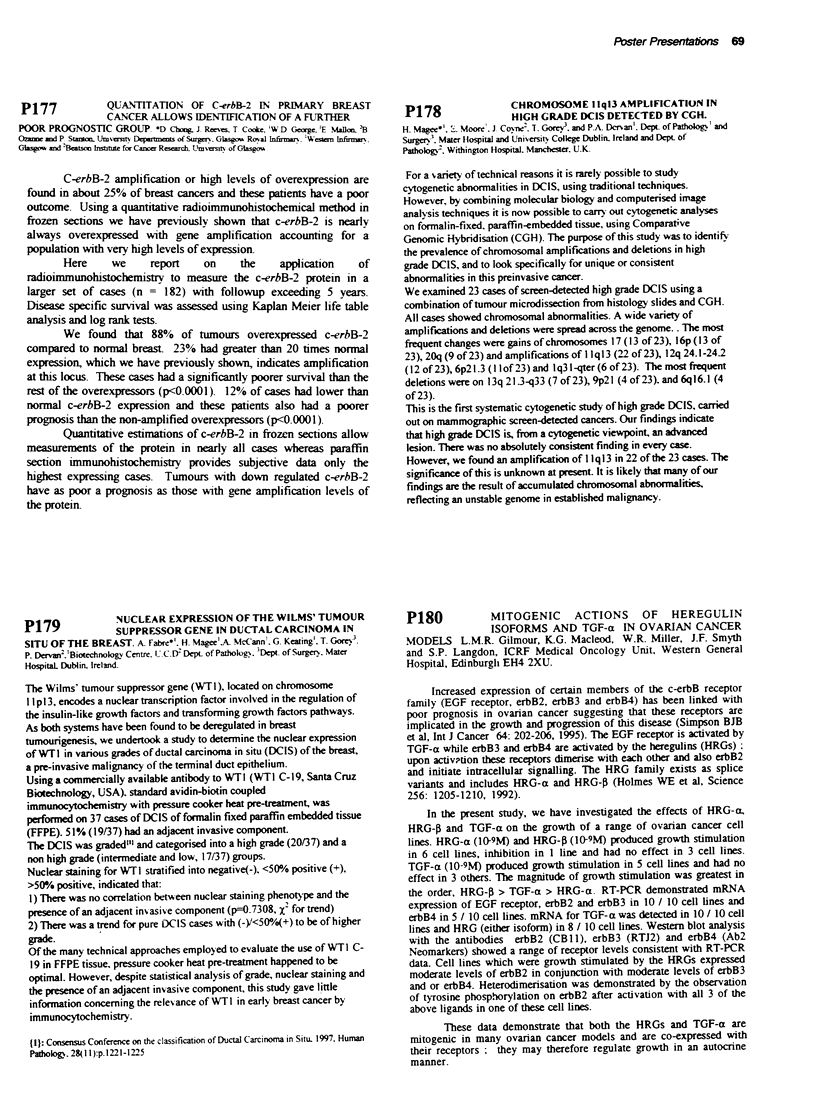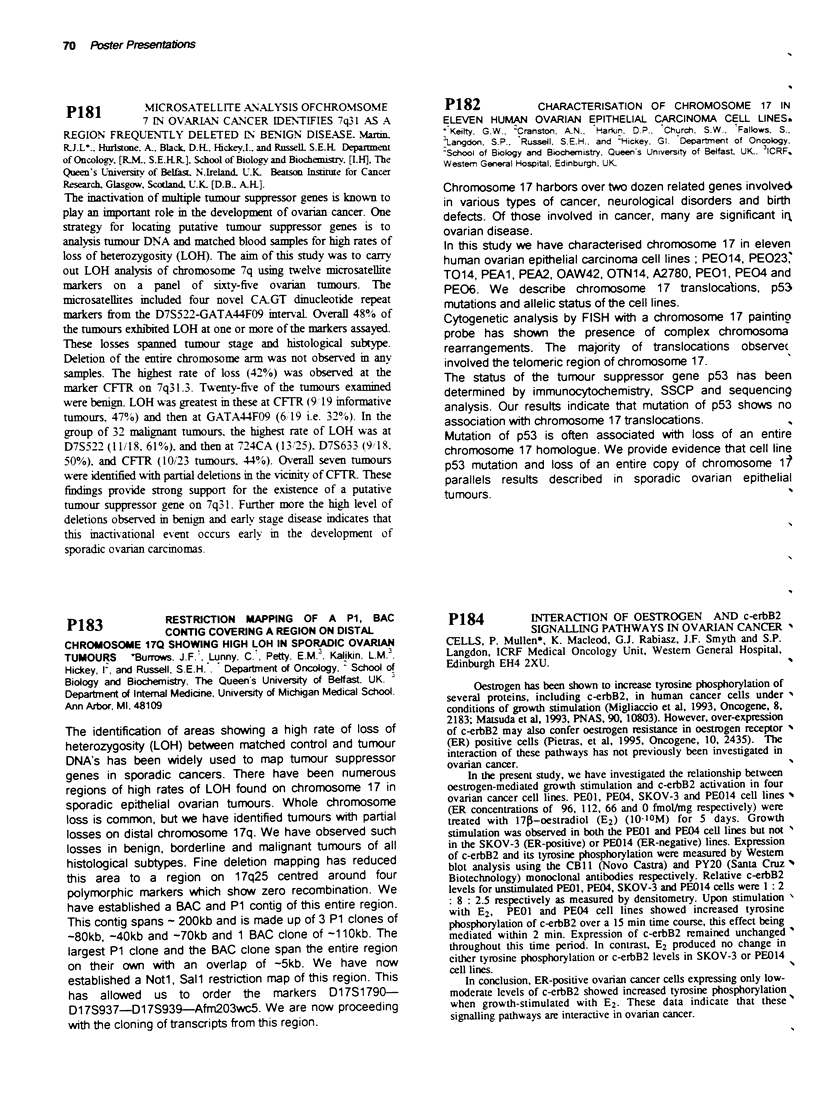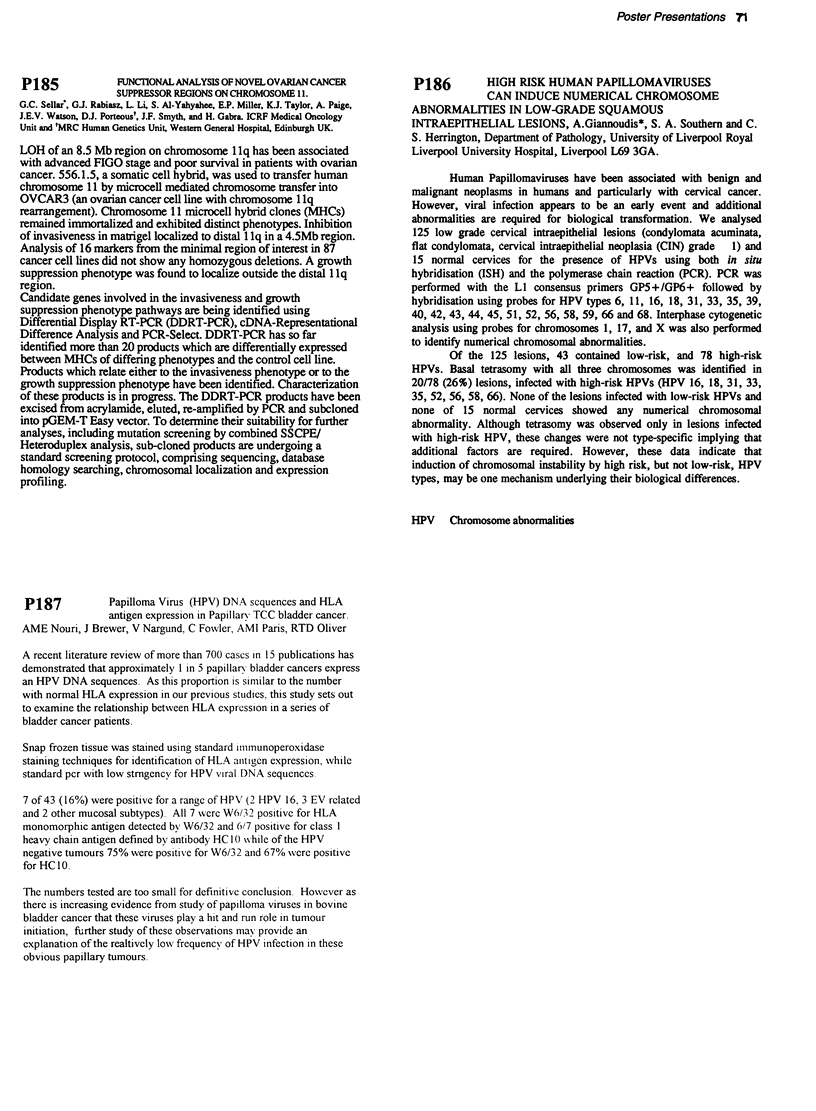# Poster Presentations

**Published:** 1998

**Authors:** 


British Journal of Cancer (1998) 78(Suppl 1), 25–71
© Cancer Research Campaign 1998

### P1

PRIMARY CULTURES OF HUMAN TUMOURS - *IN VIVO* INSIGHTS FROM *IN VITRO* RESULTS, B. C. Baguley* and E. S. Marshall, Auckland Cancer Society Research Centre, University of Auckland School of Medicine, Private Bag 92019, Auckland 1000, New Zealand.

We have developed methods for the short-term culture of tumour tissue taken at surgery from patients with cancer of lung, ovary and other sites. The challenge of this work is to determine how to identify *in vitro* characteristics of the tumour that might be useful for treatment decisions. We used 96-well agarose-coated plates for culture, an incubation time of 7 days and $^3$H-thymidine incorporation as a measure of proliferation. Culture doubling times, estimated from the reduction in S-phase cells following exposure to paclitaxel, varied upwards from 2.5 days with a median of 4.5 days. We compared dose-response curves of primary cultures and of several established cell lines using cisplatin, carboplatin, doxorubicin, etoposide, hydroperoxycyclophosphamide and an inhibitor of the epidermal growth factor receptor (EGFR) receptor kinase. $IC_{50}$ values (drug concentrations required for 50% inhibition of incorporation) for the EGFR inhibitor clearly delineated cultures into sensitive and resistant categories. $IC_{50}$ values for the DNA damaging drugs identified cultures that were apparently much more sensitive to a number of drugs than were any of the established cell lines. This was true even when values were corrected for doubling times, which were generally longer for primary cultures than for cell lines. It appears that cultures showing apparent sensitivity respond to small amounts of DNA damage either by induction of apoptosis or by sustained arrest in $G_1$- or $G_2$-phase. Such behaviour might rely on check-point and other responses that are reduced or lost during the development of established cell lines. Studies of responses of primary cultures may thus not only provide kinetic data of use to the choice of treatment, but might also provide information relevant to the development of new therapeutic approaches to cancer treatment.

*Supported by the Auckland Division Cancer Society of New Zealand*

### P2

INVESTIGATION OF MULTIPLE DRUG RESISTANCE (MDR) PROTEIN LEVELS ON PRE AND POST TREATMENT SAMPLES FROM BREAST CANCER PATIENTS, AM Larkin* [1], E Moran[1], SM Kennedy[2] and M Clynes[1], [1] National Cell and Tissue Culture Centre, Bioresearch Ireland, Dublin City University, Glasnevin, Dublin 9, Dept. of Pathology, St. Vincents Hospital Dublin, Elm Park, Dublin 4.

Development of drug resistance constitutes a major obstacle in the successful treatment of several human cancers. Breast cancer is usually initially responsive to a wide variety of chemotherapeutic drugs, but the duration of response is often short and many of patients will eventually relapse. More accurate prognostic information may allow for more precise targeting of chemotherapy for individual patients. Using immunohistochemistry we have screened pre- and post-chemotherapy archival tissue from 21 breast cancer patients who received adjuvant chemotherapy and subsequently relapsed, for a range of MDR related markers. The majority of these patients presented with high grade/ histologically undifferentiated cancers. Analysis of MDR-1 Pgp expression using two MAbs directed against different epitopes of the MDR-1 Pgp revealed significant levels of MDR-1 expression in the majority of patients at diagnosis; in general there was no significant change in Pgp levels following chemotherapy. Preliminary results of MRP protein expression indicate that MRP levels may be significantly increased following chemotherapy in a number of patients. Recent work has suggested that MRP may play a role in the prediction of response to chemotherapy in breast cancer. Results of other MDR related markers investigated will also be discussed.

### P3

THYMIDYLATE SYNTHASE EXPRESSION IN NOLATREXED AND METHOTREXATE-RESISTANT HUMAN LEUKAEMIA CELL LINES. Estlin, E.J., Hall, A.G., Lunec, J., Newell, D.R., Pearson, A.D.J. and Taylor, G.A. Cancer Research Unit and Department of Child Health, University of Newcastle upon Tyne, NE2 4HH, United Kingdom.

The classical antifolate methotrexate (MTX) is one of the mainstays of the therapy of childhood acute lymphoblastic leukaemia. MTX acts at multiple cytotoxic loci, including dihydrofolate reductase (DHFR) and thymidylate synthase (TS). Nolatrexed dihydrochoride (AG337) is a non-classical antifolate TS inhibitor which may circumvent resistance to MTX due to impaired uptake, deficient polyglutamation or overexpression of DHFR. To investigate the influence of TS expression on sensitivity to antifolates, the human leukaemia cell line clones K562:$R^{NOL}$ and Molt-4:$R^{NOL}$ were established by continuous exposure to gradually increasing concentrations of nolatrexed. A MTX-resistant clone, K562:$R^{MTX}$, was similarly established, but in medium supplemented with 10μM inosine as a TS selection pressure. When compared to parental lines, a 12-fold and 30-fold increase in nolatrexed $IC_{50}$, associated with a 4.6 and 12-fold increase in TS activity, a 3.4 and 14-fold increase in TS protein, and a 3.4 and 5.5-fold increase in TS mRNA was observed for K562:$R^{NOL}$ and Molt-4:$R^{NOL}$ respectively. The cell lines were cross-resistant to raltitrexed (a classical antifolate TS inhibitor), but not MTX. For K562:$R^{MTX}$, which was 16-fold resistant to MTX, and 8-fold resistant to trimetrexate (a specific inhibitor of DHFR), a 2-fold increase in resistance to nolatrexed and raltitrexed was observed. This was not associated with an increase in TS expression. For K562:$R^{MTX}$, cross resistance to specific antifolate TS inhibition may be the result of an increase in the intracellular reduced folate pool.

### P4

GLUTATHIONE LEVELS IN LEUKAEMIC BLASTS, P. Kearns[2,1], R. Pieters[1], M.M.A. Rottier[1], A.J.P. Veerman[1], K.Schmiegelow[3], A.D.J.Pearson[2] & A.G. Hall*[2]. [1]AZVU, Amsterdam, [2]Dept. of Paed. Oncology, University of Newcastle, [3]The Juliane Marie Centre, Copenhagen.

**Aims:** To test the hypothesis that glutathione (GSH) is an important determinant of treatment response for childhood acute leukaemia, blast cell GSH levels were studied in a cohort of children with acute lymphoblastic (ALL) and acute myeloid leukaemia (AML).

**Background:**. GSH is an intracellular thiol implicated in the development of cytotoxic drug resistance and appears to be involved in the control of cell proliferation and apoptosis. In both ALL and AML, the disease at relapse is more resistant to treatment. Several indicators of poor prognosis are well established but the underlying molecular mechanisms leading to resistant disease are still poorly understood. GSH may play a role in mechanisms of treatment failure.

**Materials and methods:** Total GSH was measured by a modified enzyme recycling assay. Cryopreserved blasts from 62 childhood ALL and 13 AML patients were analysed.

**Results:** Median GSH levels in leukaemic blasts were significantly higher in AML (11.48 nmol/mg protein) than in ALL ( 6.54 nmol/mg protein, p= 0.014). For ALL, there was a significant correlation between presenting white cell count (WCC) and GSH level (r =0.45, p=0.001). GSH was 2.2 fold higher in T lineage ALL compared with B lineage ($p < 0.0001$). Patients with ALL with a higher GSH level had a significantly worse survival (p=0.01).

**Conclusions:** GSH levels were higher in AML compared with ALL patients. Patients with a higher WCC had greater GSH levels and there was an independent correlation with T lineage immunophenotype. High GSH levels were also associated with increased risk of relapse. GSH may therefore provide a molecular mechanism by which a high WCC results in a poorer prognosis.

This work was supported by the Leukaemia Research Fund

### P5

### IDENTIFYING MOLECULAR MARKERS PREDICTIVE OF SURVIVAL AND BENEFIT FROM ADJUVANT 5-FLUOROURACIL IN COLON CANCER.

*P.L. Barratt*[*1], *M.T. Seymour*[2], *S. Stenning*[3], *S.P. Joel*[4] *and P. Quirke*[1]. [1]Institute of Pathology, Leeds University, Leeds [2]ICRF Cancer Medicine Research Unit, Leeds [3]AXIS Steering Committee, MRC Cancer Trials Office, Cambridge [4]Barry Reed Oncology Lab, St Bartholomew's Hospital, London

Adjuvant chemotherapy for colon cancer is of proven benefit overall. Treatment decisions are currently based mainly on Dukes' stage, however more accurate indication of which patients are destined to relapse and which of these will benefit from adjuvant chemotherapy, would allow better targeting of this treatment. We are looking for molecular markers which may help both to refine prognosis and to predict the usefulness of adjuvant therapy with 5-fluorouracil (5FU). This is a joint project with the collaborators of AXIS, a trial in which colon cancer patients have been randomised to receive either surgery alone or surgery plus intra-hepatic portal 5FU. Paraffin-embedded normal and tumour tissue from 400 AXIS patients with Dukes' stage B or C colon cancer has been retrieved for DNA analysis and immunohistochemistry. We have developed a fluorescent multiplex PCR allowing the co-amplification of 4 microsatellite loci from extracted DNA. By running products on an automated DNA sequencer, in conjunction with Genescan Analysis software, we obtain extremely accurate sizing and cross-sectional representation of the PCR products. The 4 loci chosen are at regions of interest in colorectal carcinogenesis: p53 (within the p53 locus); D5346 (close to the APC locus); D18S851 (close to the DCC locus) and D18S61 (distal to D18S851 on chromosome 18q). This allows detection of allelic imbalance between normal and tumour tissue and also the detection of microsatellite instability (MI) (which indicates DNA mismatch repair deficiency). We present here the methods developed, and preliminary data regarding the incidence of detection of allelic imbalance and MI. Among the first 100 patients analysed, MI is seen in 14%. Allelic imbalance at p53 was seen in 69% of informative cases; at D5346 in 42%; at D18S851 in 65% and at D18S61 in 64%. Tumours with MI will be further studied immunohistochemically using antibodies to hMLH1 and hMSH2 proteins. Additionally, immunohistochemical studies of p53, thymidylate synthase, bcl-2 and c-myc proteins are underway. The results obtained from all of these studies will, in the future, be linked with clinical data to determine correlations with survival and the effect of adjuvant 5FU.

### P6

### THE ROLE OF THE MISMATCH REPAIR PROTEIN MLH1 IN RESISTANCE TO DOXORUBICIN

H.J. Mackay[1], M. Illand[1], R. Borts[2] and R. Brown[1]. [1]Dept. of Medical Oncology, CRC Beatson Laboratories, Garscube Estate, Bearsden, Glasgow.G61 1BD. [2]Yeast Genetics, IMM,John Radcliffe Hospital, Oxford.

Loss of expression of mismatch repair proteins is associated with resistance to mono-functional and bifunctional alkylating agents. An association has been shown between loss of MLH1 and resistance to the clinically important chemotherapy agent doxorubicin(Drummond et al. J Biol Chem, 1996, **271**,19645-8). In order to define this association further we have examined the effect of loss of MLH1 in *S. Cerevisiae* and in human ovarian cell lines.

Sensitivity of wild type *S. Cerevisiae* to doxorubicin was compared to an isogenic strain deleted for the sc*Mlh1* gene. Clonogenic assay demonstrated an IC50 of 5μM for the wild type strain compared with 65μM for the sc*Mlh1* deleted strain. This represents a relative resistance of 13 fold ($p<0.05$).

The cell line A2780/cp70 is a cisplatin resistant derivative of A2780 known to be cross resistant to doxorubicin and to have lost expression of h*Mlh1*. Microcell mediated transfection of chromosome 3 into A2780/cp70 results in restoration of MLH1 expression. To assess the contribution of MLH1 to doxorubicin resistance, we compared doxorubicin sensitivity in these 2 cell lines with parental A2780. A2780/cp70 transfected with chromosome 2 was used as a control. Clonogenic assay demonstrated an IC50 value of 2.8nM for A2780. The IC50 value for A2780/cp70 was 6.3nM representing a 2.3 fold resistance compared to A2780. Introduction of chromosome 3 resulted in a partial restoration of sensitivity to doxorubicin IC50 of 4nM (1.4 fold resistance compared to parental A2780). The difference between all IC50 values was significant at $p<0.05$. The chromosome 2 transfectant line IC50 was not significantly different to A2780/cp70 (6.2nM). Finally, we have isolated multiple independent doxorubicin resistant lines from both human ovarian and breast tumour cell lines and are currently examining them for frequency of loss of mismatch repair.

In conclusion we have demonstrated that loss of MLH1 expression leads to doxorubicin resistance in *S. Cerevisiae.* Loss of MLH1 expression also contributes to the cross resistance to doxorubicin exhibited by the cisplatin resistant cell line A2780/cp70. These results support a role for loss of MLH1 in doxorubicin resistance.

### P7

### CISPLATIN INDUCED SISTER CHROMATID EXCHANGES IN OVARIAN CARCINOMA CELL LINES OF DIFFERING MISMATCH REPAIR STATUS.

M. Illand* and R. Brown. Dept. of Medical Oncology, CRC Beatson Laboratories, Garscube Estate, Bearsden, Glasgow.G61 1BD.

A2780/cp70 is a cisplatin resistant derivative of the A2780 human ovarian carcinoma cell line. A2780/cp70 has lost expression of the hMLH-1 mismatch repair gene as characterised by Western analysis and semi-quantitative RT-PCR. Complementation of mismatch repair activity by restoration of hMLH-1 expression via microcell mediated chromosome transfer of human chromosome 3 into the A2780/ Cp70 cell line results in a partial restoration of cisplatin sensitivity (Table 1). Thus, transfer of human chromosome 3 into A2780/cp70 cell lines (cp70/chr3) significantly increased the sensitivity by approximately five fold ($p < 0.05$), whereas transfer of human chromosome 2 (which fails to complement mismatch repair in these lines) does not significantly alter cisplatin sensitivity.

We propose that cells may be able to bypass lesions in DNA by recombination events during DNA replication and that this is increased upon loss of DNA mismatch repair activity. A prediction of this model would be that the induction of sister chromatid exchanges (SCE's) by cisplatin would be mismatch repair dependant. In order to address this we have examined SCE induction by cisplatin in A2780 models of differing mismatch repair status. SCE's were quantified by Hoechst staining, followed by Geimsa staining of BudR labelled metaphase spreads.This data is summarised in Table 1.

| Cell Line | I.C.$_{50}$ Values (μM cisplatin) | Mean Increase in SCE / cell | |
|---|---|---|---|
| A2780 | 10 | 5.3 | (1.2) |
| Cp70 | 65 | 13.5 | (1.6) |
| Cp70/Chr3 | 12 | 8.7 | (1.4) |
| Cp70/Chr2 | 56 | 17.1 | (1.1) |

I.C.$_{50}$ values (μM cisplatin*)* for 1h exposure to cisplatin, and the mean increase of SCE's scored in forty metaphase spreads/cell line following a 1h exposure to *10*μM cisplatin. Figures in parenthesis represent one standard error.

Cisplatin induced significantly more SCE's in the A2780/cp70 cell line than the A2780 parental line ($p < 0.01$). Similarly, the cisplatin induced SCE level in the A2780/cp70/chr3 transferrants (with restored mismatch repair) was significantly lower than the control A2780/cp70/chr2 transferrants ($p<0.01$). These observations supports the hypothesis that increased recombination bypass during DNA replication is a mechanism of cisplatin drug resistance and that this bypass occurs in a mismatch repair dependant manner.

### P8

### CHARACTERISATION OF MICROSATELLITE INSTABILITY, MUTANT FREQUENCY AND CHEMOSENSITIVITY IN TWO MURINE ADENOCARCINOMA MODELS,

MJ Thompson, R Slack, MC Bibby & JA Double. Clinical Oncology Unit, University of Bradford, Bradford, West Yorkshire, BD7 1DP, UK.

Loss of DNA mismatch repair (MMR) has been described in a number of tumour types and leads to microsatellite instability. This is observed in certain cases of colorectal adenocarcinoma and may have clinical relevance due to mismatch repair defects altering chemosensitivity towards certain classes of anti-tumour agent. This study has examined microsatellite instability, mutant frequency, and chemosensitivity of two murine adenocarcinoma tumour models, the MAC15A and MAC13 lines.

DNA was extracted from fresh and paraffin embedded normal and tumour tissues up to twenty years old. A preliminary study of two microsatellite regions (D19MIT36 and D7MIT62) demonstrated that both adenocarcinoma lines exhibit microsatellite instability, with an increase in the number of microsatellite alleles over time compared to normal tissues. The observed instability was associated with a HPRT mutant frequency (MF) of $39.1\times10^{-6}$ ($\pm5.943 \times10^{-6}$) for the MAC15A, which is significantly higher ($p<0.005$) than the $<1.74 \times10^{-6}$ ($\pm0.03 \times10^{-6}$) for the MAC13 adenocarcinoma. This compared to a MF of $152\times10^{-6}$ ($\pm1.68 \times10^{-6}$) and$2.22\times10^{-6}$ ($\pm1.077\times10^{-6}$) for the MMR deficient HCT116 and MMR normal SW620 human adenocarcinoma cell lines respectively. Defects in mismatch repair have been found to result in a particular pattern of chemosensitivity towards agents such as 6-thioguanine (6-TG) and cisplatin. The $IC_{50}$ for cisplatin was 1.003μM (±0.857) for the MAC15A and 0.785μM (±0.474) for the MAC13. For 6-TG, the $IC_{50}$ values were significantly different ($p<0.005$) at 0.623μM (±0.1347) and 0.0387μM (±0.0189) for MAC15A and MAC13 respectively.

This work therefore demonstrates two mouse adenocarcinoma tumour models showing evidence of microsatellite instability associated with significantly differing mutant frequencies and chemosensitivity towards 6-TG but not cisplatin. Further study of the MAC13 & 15A tumours, along with other MAC lines, should provide us with a number of well characterised in vivo models of microsatellite instability and chemosensitivity in colonic adenocarcinomas.

This work was supported by the War on Cancer charity.

### P9 THE ROLE OF THE DNA MISMATCH REPAIR PROTEIN, hMLH1 IN SENSITIVITY OF COLON CANCER CELLS TO CHEMOTHERAPEUTIC AGENTS USED CLINICALLY.

*P.L. Barratt*[1], M.T Seymour[2], R.M. Phillips[3], M.C. Bibby[3], J.A. Double[3] and P. Quirke[1]. [1]Institute of Pathology, Leeds University, LS2 9JT, [2] ICRF Cancer Medicine Research Unit, Leeds, [3]Clinical Oncology, University of Bradford, BD7 1DP.*

Mutations of hMLH1 or hMSH2 are the most common cause of loss of DNA mismatch repair (MMR) function in colorectal cancer. Absence of DNA MMR is observed in 90% of patients with Hereditary Non-Polyposis Colorectal Cancer (HNPCC) and 15% of sporadic colorectal cancer cases. It has previously been shown *in vitro* that loss of expression of the DNA MMR protein, hMLH1 increases resistance to MNNG, carboplatin and cisplatin. We have investigated, in an *in vitro* model, the importance of the hMLH1 protein in determining sensitivity to chemotherapeutic drugs currently in use in the treatment of colorectal cancer. The colon cancer cell lines used were HCT116, which has a homozygous mutation of hMLH1, and two derivative cell lines, HCT116+3 (corrected for expression of hMLH1 by the addition of a normal copy of chromosome 3) and HCT116+3M2 (addition of a normal copy of chromosome 3, with subsequent loss of expression of hMLH1). Expression of hMLH1 was confirmed by western blot and a microculture tetrazolium assay used to measure cell growth. Loss of hMLH1 protein expression conferred an increase in resistance to 5-Fluorouracil plus Leucovorin (1.8 fold), but an increase in sensitivity to Oxaliplatin (4.3 fold), Mitomycin C (3.3 fold) and SN38, the active metabolite of Irinotecan (2.8 fold). Therefore it was clearly demonstrated that hMLH1 does have a function in predicting response to these chemotherapeutic agents in an *in vitro* setting. As loss of hMLH1 expression was observed to confer altered sensitivity to agents which induce a range of insults to DNA a wide role for this protein in the recognition and signaling of cell damage was suggested. Further investigation of the role hMLH1 and of the other DNA MMR proteins may be important in aiding the targeting of chemotherapeutic agents for patients with abnormalities in this group of genes.

### P10 THE MISMATCH REPAIR GENE, MLH1 MEDIATES SENSITIVITY TO CISPLATIN IN *SACCHAROMYCES CEREVISIAE*.

S. Durant*[1], M. Morris[1], C. McCormick[1], G. Hirst[1], R. Borts[2] and R. Brown[1], [1]Medical Oncology, CRC Beatson Laboratories, Garscube Estate, Glasgow, G61 1BD; [2]Yeast Genetics, IMM, John Radcliffe Hospital, Oxford.

These results strongly suggest that the MMR gene, MLH1 confers sensitivity to CDDP but not to UV-induced DNA damage in *S.cerevisiae*. We propose that DNA damage induced by CDDP can mediate MLH1-dependent cell death.

### P11 MISMATCH REPAIR PROTEIN IMMUNOHISTOCHEMISTRY IN OVARIAN CANCER

On interim analysis there is an association between the ***I*** and **%** scores. Immunoscores for Ki67, as a marker of proliferation, are not associated with p53, MLH1, or MSH2 scores (Pearson correlation coefficient = 0.11, 0.07, 0.25 respectively). Comparing all the pre (n=32) against the post (n=28) chemotherapy samples there is a significant increase in immunoscores for MSH2 post chemotherapy, (mean = 3.87 and 4.65 respectively; two tail t-test p value = 0.02) but no significant change overall for MLH1, p53 or Ki67 scores. Restricting the analysis to paired samples (n=22) this difference in MSH2 is not seen. However, in the relapsed (at over 1 year) disease pairs (n=13) there is a significant increase of 11.1% in the Ki67 scores (95% CI = 1.6 to 20.6%), suggesting an increase in proliferation rate of these tumours with time. Comparing the residual disease pairs (n=9) to the relapse disease pairs (n=13) we have seen a trend for MLH1 socres to decrease immediately post chemotherapy (-0.47; 95% CI -1.55 to 0.61) compared to an increase one year or more post chemotherapy (+0.38; 95% CI -0.37 to 1.14).

In conclusion, we have developed a valid technique and scoring system for mismatch repair proteins. We have shown an increase in scores for MSH2 in post chemotherapy compared to pre chemotherapy samples on interim analysis. Work is ongoing to complete immunoscoring blind for ovarian cancer samples before analysing the clinical relevance of immunostaining for mismatch repair proteins.

### P12 THE MISMATCH REPAIR GENE *MLH2* AND SENSITIVITY TO CISPLATIN

The *scMLH2* gene was originally identified by virtue of its sequence homology to other yeast *MUTL* genes. In order to identify possible human homologues of the *scMLH2* gene we have utilised PCR with redundant primers against two regions of conserved amino acid sequence within the *MUTL* gene family. The 3' primer recognises the *MLH1* box which encodes for the amino acid sequence GFRGEAL and is completely conserved among the MutL proteins. The 5' primer anneals to a region encoding for a slightly less conserved amino acid sequence than the 3' primer. Redundant PCR product was amplified from normal human cDNA reverse transcribed from replicating, normal foreskin keratinocyte RNA. The resultant PCR product was cloned by TOPO TA cloning® and clones were screened by PCR for the presence of insert. The PCR products from this screen were Southern blotted and hybridised to radiolabelled full length human *MLH1*. Six out of 96 colonies picked were found to contain human *MLH1* sequences. Thus redundant PCR is successful for isolating *MUTL* homologues. We are currently analysing the remaining clones for novel *MUTL* homologues.

### P13 THE ROLE OF MISMATCH REPAIR MUTS HOMOLOGUES IN THE CISPLATIN AND UV SENSITIVITIES OF *SACCHAROMYCES CEREVISIAE*

G.Hirst,* S.Durant, M.Morris, C. McCormick, R Borts[Ψ] and R.Brown
CRC Dept Medical Oncology, CRC Beatson Laboratories, Switchback Rd, Glasgow G61 1BD and [Ψ]IMM, John Radcliffe Hospital, Oxford OX3 9DS

Recent evidence strongly supports a direct role for DNA mismatch repair in the sensitivity of mammalian cells to a wide range of DNA damaging agents. Amongst this data, it has been shown that hMSH2, the human homologue of the bacterial MutS mismatch repair protein, binds to the cisplatin 1,2-intrastrand crosslink *in vitro*. (Duckett et al. 1996. PNAS 93, 6443). Furthermore, cell lines deficient in hMSH2 gain increased sensitivity to cisplatin following reintroduction of the gene by chromosome transfer (Aebi et al. 1997. Clin Cancer Res 3,1763). To understand the mechanisms underlying mismatch repair involvement, we examined the clonogenic sensitivities to cisplatin and UV radiation in isogenic strains of the yeast *Saccharomyces cerevisiae* disrupted for one of three MutS homologues, MSH2, MSH3 or MSH6. 2x10[illegible] cells were treated with 0-3mM cisplatin for 24hours in liquid culture. 400 cells were subsequently plated on YPD culture plates, and colonies counted 3 days later. For UV treatment cells were plated, irradiated with 0-250 $J/m^2$ UVC irradiation at 254nM, and colonies counted three days later. The three single mutants were significantly 1.6 fold (MSH6), 1.7-fold (MSH3) and 2.3 fold (MSH2) more resistant to cisplatin treatment than the isogenic wild type strain, ID90=1.3mM ($p<0.005$). However, the sensitivity of these single mutants to UV treatment was not significantly changed from wild type (ID90 =170 $J/m^2$). Reintroduction of MSH2 back into the MSH2-disrupted mutant increased the sensitivity of this strain approximately 1.4 fold to cisplatin, (ID90=2.0mM) as compared to the mutant strain (ID90=3mM, $p<0.05$), implicating a direct involvement of MSH2 in cisplatin sensitivity. The clonogenic sensitivity to these agents was also examined in yeast disrupted for the recombinational repair gene, RAD52, which are hypersensitive to both cisplatin (ID90=0.73mM) and UV treatment (ID90=70 $J/m^2$) as compared to wild type. Results showed that a disruption of the MSH2 gene in this background did not significantly alter the hypersensitivity to cisplatin or UV. These findings provide direct evidence that members of the mismatch repair family are involved in the cytotoxicity to cisplatin and show that MSH2 may modulate cytotoxicity in a RAD52 dependent pathway. One possibility is that mismatch repair may influence a recombinational adduct bypass mechanism dependent on RAD52. When mismatch repair is disrupted, loss of control of this bypass mechanism may lead to resistance.

### P14 ROLE OF P53 IN HUMAN OVARIAN CANCER TUMOUR XENOGRAFTS CURABLE WITH CISPLATIN.

KE Pestell*, CJ Medlow, M Jones, M Valenti, L Brunton, LR Kelland and MI Walton. CRC Centre for Cancer Therapeutics, ICR, 15 Cotswold Road, Sutton, Surrey, SM2 5NG, UK.

Mutation of the tumour suppressor protein p53 has been implicated in resistance of ovarian cancer to cisplatin. We have determined the p53 and bcl-2 status of a pair of human ovarian tumour xenografts (PXN65 and PXN100) curable with cisplatin, and investigated the effects of a single dose of cisplatin on induction of genes downstream to p53 and on DNA repair. Both of these tumours fail to grow *in vitro*. Functional p53 status was determined by measuring MDM-2 and $p21^{WAF1/CIP1}$ mRNA induction 4 hours following 5 Gy by northern blotting. Single-strand conformational polymorphism and sequencing of exons 5, 6, 7 and 8 of P53 were carried out using standard techniques. Bcl-2 protein levels were determined by immunoblotting. Following a single dose of 6mg $kg^{-1}$ cisplatin i.p. induction of $p21^{WAF1/CIP1}$, MDM-2 and BAX mRNA was determined by northern blotting and gene-specific repair was determined by quantitative PCR of a 1.9Kb fragment of H-RAS. The PXN65 tumour showed no re-growth after 350 days following 3 X 6mg $kg^{-1}$ i.p. cisplatin The PXN100 tumour showed no re-growth after 300 days following 4 X 8mg $kg^{-1}$ i.p. cisplatin. Following a single dose of 6mg $kg^{-1}$ cisplatin the growth delay was 76.1 and 24.9 days for PXN65 and PXN100, respectively. $p21^{WAF1/CIP1}$ and MDM-2 mRNA levels were elevated 4 and 2.5 fold respectively following irradiation in PXN100, suggesting wild type p53 function. $p21^{WAF1/CIP1}$ and MDM-2 mRNA were barely detectable following irradiation in PXN65, suggesting no wild type p53 function. No mutations were found in PXN100, but a deletion of bp 868 at codon 290 and a insertion of GTGGTGAG was seen in PXN65 causing a frame shift and premature stop at codon 306. High levels of bcl-2 protein were present in both xenografts and levels were elevated following irradiation. $p21^{WAF1/CIP1}$, MDM-2 and BAX mRNA levels were induced 3-fold at 24 hours following a single dose of 6mg $kg^{-1}$ cisplatin in PXN100, but were undetectable in PXN65. There was measurable damage to the H-RAS gene following a single dose of 6mg $kg^{-1}$ cisplatin that was repaired by 24 hours post treatment in both tumours. This work suggests that p53 may not be the primary determinant of response to cisplatin in these human ovarian cancer tumour xenografts.

### P15 p53, p21 (WAF-1), APOPTOSIS AND DIFFERENTIATION IN RETINOBLASTOMAS,

A Divan*[1], R Stewart[5], P Mullaney[6], S Mesfer[6], ZA Karcioglu[6], IR Dunsmore[2], IG Rennie[3], J Lawry[2], MA Parsons[4] and JA Royds[4]. [1]Institute for Cancer Studies, [2]Mathematics & Statistics, [3]Opthalmology & Orthoptics, [4]Opthalmic Sciences Unit, [5]Pathology, University of Sheffield, Sheffield, UK; [6]Opthalmology Department, King Khaled Eye Hospital, Riyadh, Saudi Arabia

Retinoblastoma is a childhood tumour caused by the recessive inactivation of the RB1 tumour supressor gene. The RB1 product, pRb, acts to regulate the balance between cell cycle progression, differentiation and apoptosis and consequently loss of this protein results in the disruption of the differentiation programme and unlimited cell proliferation which is accompanied by extensive apoptosis.

We have quantified the distribution of p53, its downstream effector p21 (WAF-1) and apoptotic cells by immunohistochemistry in retinoblastomas within the proximal, middle and distal zones of tumour sleeves composed of viable tissue surrounding a central blood vessel with outer regions of confluent apoptosis, in tumour tissue of varying extents of differentiation.

By Kruskal-Wallis analysis, we have demonstrated that in poorly differentiated areas of the tumour, p53 expression increases from the proximal to the distal regions of the tumour as does the proportion of apoptotic cells while p21 expression is restricted primarily to the proximal regions of the tumour adjacent to the blood vessel. By contrast in well differentiated areas of the tumour, this pattern of p53 expression is reversed. However, the distribution of p21 and the proportion of apoptotic cells remain independent of the differentiation state. By Spearman rank test, p53 expression correlates positively with apoptosis but negatively with p21. Sequencing of exons 5-8 of retinoblastoma cell lines Y79 WERI-Rb1 and archival material indicates that the expressed p53 is wild type. Our in vitro study using the Y79 cell line indicates that the balance of cell proliferation and cell death is influenced markedly by FCS concentration and to a lesser extent oxygen availability and that optimum p53 induction is observed under growth factor and oxygen limiting conditions.

We conclude that retinoblastoma cells are protected from p53 mediated programmed cell death by the production of survival factors which are stimulated by the diffusion of oxygen and growth factors delivered to the tumour by the blood vessel and that p21 is involved not in cell death but in cell survival. Recent studies also indicate that the survival proto-oncogene Bcl-2 is not involved in the cell survival mechanism.

### P16 DOES TP53 FUNCTION IN NEUROBLASTOMA ?

D A Tweddle*[1,2], A D J Pearson[1], A J Malcolm[3] and J Lunec[2]. Departments of Child Health,[1] Cancer Research Unit[2] and Pathology[3], University of Newcastle, NE2 4HH, U.K.

Aims To test the functional integrity of the TP53 pathway in neuroblastoma cell lines by induction of TP53 in response to DNA damage and measuring the effect on 3 TP53 upregulated genes: *WAF1*, *BAX* and *MDM2*, together with one which it may downregulate: *BCL2*.

Background Wild-type TP53 has been reported to be overexpressed and abnormally located in the cytoplasm of neuroblastoma tissue and cell lines where it may be unable to function normally[1,2]

Methods 4 neuroblastoma cell lines with wild-type *TP53*, the neuroepithelioma cell line NB100, (which has a *TP53* mutation producing a non-functional truncated protein[3]) (-ve control) and the breast cancer MCF-7 cell line (+ve control[4]), were treated with 4Gy of ionising radiation (IR). Cells were harvested at different times and TP53, WAF1, MDM2, BCL2 and BAX expression detected by immunocytochemistry and Western Blotting using enhanced chemiluminescence. In addition, apoptosis was scored by Hoechst nuclear staining.

Results The peak values and times (hours) for the various proteins relative to untreated controls, and % apoptosis are shown below. In all neuroblastoma cell lines studied TP53 was predominantly nuclear pre and post IR.(→ =no change)

| | TP53 | WAF1 | BAX | MDM2 | BCL2 | apoptosis |
|---|---|---|---|---|---|---|
| NB100 (-) | 0 | 0 | → | 6 (4) | → | 7.5 (108) |
| MCF-7(+) | 7 (2) | 10 (6) | → | 2 (4) | 4 (6) | 2 (72) |
| SHSY5Y | 12 (2) | 20 (6) | 2 (48) | 0 | → | 15 (6) |
| SKNSH | 7 (6) | 1.5 (6) | → | 0 | → | 3 (6) |
| SHEP | 5 (4) | 3 (24) | 2 (6) | 10 (6) | 0 | 9 (24) |
| IMR-32 | 12 (4) | 16 (4) | 2 (2) | 7(4) | → | 34 (48) |

Conclusions Nuclear TP53 is inducible by IR in all the neuroblastoma cell lines studied with wild-type *TP53*. WAF1 induction correlates with TP53 induction but induction of other TP53 response genes is variable, suggesting that the TP53 pathway may be intact in some cell lines (IMR-32, SHEP, SHSY5Y) but not others (SKNSH). Further studies are in progress to confirm and extend these findings.

References 1) Moll *et al*, Proc.Natl Acad Sci USA 1995:92:4407.2) Moll *et al* Mol & Cell Biol 1996;1126.3) Davidoff *et al*. Oncogene 1992;7:127.4) Gudas *et al*. Carcinogenesis 1996:17(7);1417.

### P17 EXPRESSION OF P53, WAF1, MDM2 AND BCL-2 PROTEINS IN HUMAN GERM CELL TESTICULAR TUMOURS.

E Institoris*, H.Eid, I.Bodrogi, L.Géczi and M.Bak, National Institute of Oncology, 1122. Budapest, Ráth György str. 7-9. Hungary

We investigated the expression of p53 tumour suppressor gene protein and its related products mdm2, wafl and bcl-2 proteins in human germ cell testicular tumours (GCTTs) using Western blot or/and immunohistochemical analyses. Except differentiated teratoma, all GCT tumours of untreated patients (77) were immunoreactive for p53 to various extent. Seminomas and embryonal carcinoma components had the most positive immunostaining. P53 showed a significant inverse correlation with stage of disease (P< 0.003). Numerous recent studies strongly suggest that the p53 in GCT cancer has wild-type character. In contrast with mutant p53 the wild-type p53 is able to induce cyclin dependent kinase inhibitor i.e. wafl protein (El Deiry et al., Cell 75:817, 1993), which is a crucial protein in cell-cycle control. Therefore, we analysed GCTT samples (36) for wafl using Western blot technique. There was no sign of wafl expression in any tumours investigated. The same cohort studied for wafl was analysed for mdm2 by Western blot and immunohistochemistry as well. 30% of GCT tumours displayed mdm2 expression. The failure of wafl expression in GCTTs can not be explained by p53 inactivation caused by mdm2, since 70% of tumour samples did not express mdm2 protein at all. We also screened bcl-2 expression in patients with GCTTs using immunohistochemistry. 58% of tumours stained with anti-bcl-2. The bcl-2 expression was clearly dominant in tumours of advanced stages and the incidence of bcl-2 exprression was higher in tumours from metastatic patients than in tumours from metastatic-free patients (P < 0.000). Our findings suggest that bcl-2, during suppression of wafl may interfere with the functional properties of p53 protein and this interaction may be a mechanism by which bcl-2 exercises its oncogenic potential.

This work was supported by National Scientific Research Fund No T 019770.

### P18 REGULATION OF ETOPOSIDE-INDUCED APOPTOSIS IN NEURO-BLASTOMA: RELATIONSHIP TO p53 FUNCTION.

A.M. Rodríguez-López[1]*, O.B. Eden[2], J.A. Hickman[1] & C.M. Chresta[1]. [1]CRC Molecular Pharmacology Group, School of Biological Sciences. University of Manchester, Manchester M13 9PT. [2]Christie Hospital NHS Trust, Manchester M20 4BX.

Neuroblastoma is a chemosensitive tumour and combined chemotherapy is considered an important treatment modality. Like many early onset tumours, neuroblastomas do not have p53 mutations; this play a role in drug sensitivity. However, it has been suggested that p53 may be inactivated in neuroblastomas because of cytoplasmic sequestration. We have investigated if the p53 response pathway differs between drug sensitive and resistant neuroblastoma cell lines. The cell line model systems used were: SH-SY5Y, a neuroblastic clone (N-type), and SH-EP1, a substrate adherent clone (S-type). Both are subclones of SK-N-SH which has wtp53. Etoposide produces equal levels of DNA damage in both cell types but clonogenic assays demonstrate that SH-SY5Y are >3 fold more sensitive than SH-EP1. Before etoposide treatment p53 is located in the cytoplasm of both cell types, although at significantly higher levels in SH-EP1. Four hours after drug treatment the majority of p53 translocates to the nucleus in SH-SY5Y and cells undergo apoptosis whilst in SH-EP1 the majority of p53 remains in the cytoplasm. However, in SH-EP1 etoposide results in a 12.9-fold increase in $p21^{waf-1/cip-1}$ RNA and protein and cells arrest in G1-phase of the cell cycle whereas in SH-SY5Y p21 increases by only 3.4-fold and cells undergo apoptosis in less than four hours. In SH-EP1 cells 7 days after etoposide treatment (1 h) the cells have a morphologically distinct neuronal phenotype. These data suggest that neuroblastoma types that retain the p21 upregulation pathway may be relatively resistant to drug induced apoptosis and differentiate rather than die in response to etoposide. p21 has previously been demonstrated to be essential for survival of differentiating neuroblastoma cells in response to retinoic acid. Analysis of the expression of the Bcl-2 family members can not explain the differential sensitivity. Levels of expression of Bax, Bak and Bcl-XL are similar in the SH-EP1 and SH-SY5Y cells, whilst paradoxically Bcl-2 is overexpressed in SH-SY5Y, the apoptosis sensitive cell type. In conclusion, firstly we find that in neuroblastomas where the majority of wtp53 translocates to the nucleus cells rapidly undergo apoptosis. Secondly in cells which induce high levels of p21 cell cycle checkpoints are activated and cells undergo further differentiation and survival.

### P19 IDENTIFICATION OF AN APOPTOSIS-RELATED GENE UP-REGULATED IN LOW GRADE FOLLICULAR LYMPHOMA.

D.Carter[a]*, J.R.Goepel[b], P.Winship[c], M.H.Goyns[d]. [a]Y.C.R. Institute for Cancer Studies, [b]Department of Pathology, [c]Division of Molecular and General Medicine, University of Sheffield Medical School, Beech Hill Rd, Sheffield S10 2RX. [d]School of Health Sciences, University of Sunderland, Fleming Building, Wharncliffe St, Sunderland SR2 3SD.

It is well documented that a large proportion of low grade follicular lymphomas transform to a more aggressive high grade disease. Once this transformation has taken place the patients clinical outcome is particularly poor (1). A number of chromosomal and genetic changes have been described in low grade follicular lymphomas that have shown evolution to the high grade centroblastic lymphomas. Genetic analysis of this transformation has centred around the *bcl-2*, *p53* and *myc* genes.

We have used the RT-PCR technique of differential display (2) to analyse the differential gene expression pattern between the low grade follicular and high grade centroblastic lymphomas. Using this technique we have identified a cDNA that shows overexpression in low grade follicular lymphoma samples. Following cloning and sequencing, this cDNA was shown to have 100% homology with the 3' untranslated region of a recently identified pro-apoptopic gene, DP5 (3). This gene also shows some homology to the pro-apoptopic gene *hrk* (4), which interacts with Bcl-2 and $Bcl\text{-}X_L$ and may represent a family of genes involved in apoptosis regulation. Differential expression was confirmed using competitive RT-PCR and indicated that this gene showed an up-regulation of 100 fold in the low grade follicular lymphoma compared with the high grade centroblastic lymphoma samples. *In situ* hybridisation was also performed to identify the localisation of expression.

REFERENCES

(1) Bastion *et al.* (1997) J. Clin. Onc. **15**:4 p1587.
(2) Liang and Pardee (1992) Science. **257** p967.
(3) Imaizumi *et al.* (1997) J. Biol. Chem. **272**:30 p18842.
(4) Inohara *et al.* (1997) EMBO **16**:7 p1686.

### P20 BCL-W EXPRESSION IN COLORECTAL TUMOURS.

J.W. Wilson[1]*, M.C. Nostro[1], A. Becciolini[2], M. Balzi[2] and C.S. Potten[1]. [1]Section of Cell and Tumour Biology, Paterson Institute for Cancer Research, Manchester M20 4BX; [2]Universita Degli Studi, Florence, Italy.

We have examined the expression of the anti-apoptotic bcl-2 family protein, bcl-w, in more than 50 colorectal adenocarcinomas, using immunohistochemical techniques. We have found that >90% of tumours show expression of the protein, which was cytoplasmic in location. No expression of bcl-w was observed in adjacent normal epithelium taken from the same patients. No correlation was found between expression of bcl-w and bcl-2 and p53 expression and Duke's stage. In only 2 out of 9 adenocarcinomas of the stomach was sporadic bcl-w immunoreactivity observed. Adenocarcinomas from other epithelial tissues (breast, cervix) failed to demonstrate any expression of bcl-w. We are currently investigating the expression of bcl-w in adenomatous polyps. In the adenomas that have been examined so far, only 1/9 showed bcl-w expression although 6/9 demonstrated various levels of bcl-2 expression. We are currently seeking to expand this study to assess the importance of bcl-w in colorectal cancer.

### P21

**INVOLVEMENT OF Bcl-2 FAMILY MEMBERS IN DRUG RESISTANCE TO TOPOISOMERASE I (TOPO I) INHIBITORS**, J.S. Macpherson*, J. Cummings, J.F. Smyth and D. I. Jodrell, ICRF Medical Oncology Unit, Western General Hospital, Edinburgh, EH4 2XU.

Cellular responses to novel (NU/ICRF 505) and established (camptothecin, CPT) topo I inhibitors have been investigated in a panel of human cell lines and drug sensitivity was associated with the presence of functional p53 protein (with or without $p21^{WAF1/CIP1}$ induction and $G_1/S$ block). In order to gain insights into less sensitive p53-independent processes, basal expression and temporal induction of 4 key members of the Bcl-2 family were determined in wild type p53 chemosensitive A2780 ovarian cancer cells and mutant p53 chemoresistant HT29 colon cancer cells. Cells were exposed to drugs at their $IC_{50}$ concentrations and proteins measured by Western blot analysis for up to 72 hr. Bcl-2 was not detectable in both cell lines nor was it induced after drug exposure. Likewise $Bcl\text{-}x_S$ was not detectable prior to drug treatment nor stimulated post drug treatment. High basal levels of the apoptosis inhibitor protein $Bcl\text{-}x_L$ were measured in HT29 compared to A2780 whereas the reverse situation was recorded with the death inducing protein Bax. There was no evidence of $Bcl\text{-}x_L$ induction in both cell lines. CPT produced a 4-fold maximal increase in HT29 cells and 2-fold maximal increase in A2780 cells in Bax protein at 36 hr post-treatment. However, NU/ICRF 505 had no significant effect on Bax protein levels. Thus, in vitro drug resistance to topo I inhibitors is also associated with a high $Bcl\text{-}x_L$/Bax ratio as well as the presence of mutant p53. In addition, these data provide evidence that cells can respond to the DNA damage induced by different topo I inhibitors through the selective induction of apoptosis modulating proteins.

### P22

**APOPTOSIS REGULATOR BCL-2: A PREDICTIVE FACTOR IN COLORECTAL CARCINOMA**

D.T. Leahy*[1], H.E. Mulcahy[3], D.P. O'Donoghue[3] and N.A. Parfrey[1,2], [1]Dept. of Pathology, University College Dublin, Dublin 4; Depts of [2]Pathology and [3]Gastroenterology, St. Vincent's Hospital, Dublin 4, Ireland.

The bcl-2 gene encodes a protein that blocks apoptosis and may be associated with neoplastic progression by overriding programmed cell death. The bcl-2 gene was originally identified as being overexpressed in follicular lymphomas due to the t(14;18) chromosomal translocation. We investigated bcl-2 protein expression in 102 colorectal carcinomas with ten year follow-up. Formalin-fixed paraffin embedded tumour tissue sections were examined by immunohistochemistry using a monoclonal bcl-2 antibody (Dako) with microwave antigen retrieval. Genomic DNA was extracted from the tumour tissue of the cases immunohistochemically positive for bcl-2.

Cytoplasmic staining of the bcl-2 gene product was seen in the tumour cells of 22 cases (22%). Using a PCR technique we examined the possibility that the t(14;18) translocation contributes to bcl-2 overexpression in colorectal cancer but found no evidence of this. Expression of bcl-2 protein was related to tumour grade (p=0.009) but was unrelated to patient age, sex, tumour site, tumour size or Dukes' stage. In a sub-group of 66 cases, bcl-2 expression was unrelated to p53 status as assessed by immunohistochemistry and single-strand conformation polymorphism analysis.

There was a trend towards increased survival in those whose tumours expressed bcl-2 protein (p=0.055). When entered into a multivariate analysis, this survival difference was independent of tumour stage (p=0.05). These results suggest that bcl-2 expression in colorectal cancer is associated with a better long-term prognosis.

### P23

**Transfection of bax into resistant human ovarian carcinoma cell lines confers no sensitisation to chemotherapeutic agents.**

**P.Beale, P. Rogers, S. Hobbs, F. Boxall, L. Kelland. CRC Centre for Cancer Therapeutics, Institute of Cancer Research, Sutton, Surrey SM2 5NG, UK**

The BCL-2 family of proteins are known to be important in the process of apoptosis. The mechanism of action of BCL-2, an anti-apoptotic protein is unknown but it may act via ion channel pumps in the mitochondria. BAX can form heterodimers with BCL-2 and antagonise this function leading to a pro-apoptotic effect. Some studies have shown that overexpression of BAX can lead to sensitivity to some chemotherapeutic agents but not to others. We investigated two human ovarian carcinoma cell lines which are resistant to cisplatin (SKOV3, with intrinsic resistance and A2780cisR, with acquired resistance). Cell lines were transfected with bax coupled to an HA tag cloned into the bicistronic plasmid vector pIRES-P (EMBL:Z75185) or with an empty vector. Clones were selected using puromycin. BAX overexpression was confirmed by western blotting (1.4 fold increase for A2780cisR and 5.8 fold for SKOV3). Cell sensitivity to 8 drugs was assessed by 96 hour sulforhodamine B cytotoxicity assay (tables 1, 2). There was marginal sensitisation to cisplatin in the A2780cisR compared with the puromycin control (RF 1.3). For all other drugs tested there was no significant sensitisation.

| Table 1 | $IC_{50}\mu M$ | | | |
|---|---|---|---|---|
| Cell lines | Cisplatin | AMD473 | Doxorubicin | Etoposide |
| A2780cisR BAX | 5.2 ± 0.4 | 7.0 ± 0.3 | 0.08 ± 0.03 | 0.96 ± 0.5 |
| A2780 cisR puro | 6.8 ± 0.4 | 8.2 ± 0.6 | 0.18 ± 0.07 | 0.74 ± 0.5 |
| SKOV3 BAX | 11.5 ± 2.2 | 33.0 ± 4.8 | 0.22 ± 0.07 | 2.2 ± 0.6 |
| SKOV3 puro | 6.2 ±1.5 | 22.5 ± 2.4 | 0.15 ± 0.04 | 2.5 ± 1.1 |

| Table 2 | $IC_{50}nM$ | | | |
|---|---|---|---|---|
| Cell lines | Paclitaxel | Docetaxel | Vinblastine | Colchicine |
| A2780cisR BAX | 2.0 ± 0.3 | 1.1 ± 0.6 | 2.4 ± 1.5 | 16.5 ± 0.3 |
| A2780 cisR puro | 1.7 ± 0.06 | 0.47 ± 0.26 | 5.2 ± 3.4 | 16.2 ± 0.6 |
| SKOV3 BAX | 3.0 ± 1.0 | 0.69 ± 0.5 | 0.69 ± 0.5 | 17.5 ± 0.8 |
| SKOV3 puro | 3.5 ± 2.1 | 0.74 ± 0.4 | 4.2 ± 2.5 | 41.5 ± 24.5 |

Previously, we have demonstrated that transfection of bcl-2 into the A2780 cell line does not confer resistance to the same chemotherapeutic drugs and preliminary results have shown that transfection of CH1, a sensitive human ovarian carcinoma cell line with bclxl does not confer resistance to cisplatin.

Overall, these results suggest that altering the level of one of the Bcl-2 family members by transfection in these human ovarian carcinoma cell lines does not alter the chemosensitivity.

### P24

**BCL2 FAMILY PROTEIN EXPRESSION IN CHILDHOOD ACUTE LYMPHOBLASTIC LEUKAEMIA.** Hogarth L., Pearson A.D.J., and Hall, A. Paediatric Oncology, CRU, Medical School, Newcastle, NE2 4HH, UK.

*In vitro* studies have shown that expression of the bcl-2 family of proteins can determine the susceptibility of cells to undergo apoptosis in response to various chemotherapeutic agents but correlations with clinical response have been less extensively reported. We have studied protein expression of bcl-2, bax, bcl-xl, bcl-xs and mcl-1 in lymphoblasts from children with acute lymphoblastic leukaemia (ALL) using Western blotting with enhanced chemiluminescence detection and densitometry. Results were expressed as a ratio to actin as an internal control.

Samples from 46 cases at presentation and 16 on relapse showed no significant difference in protein expression (bcl-2; 3.4 vs 2.6, bax;1.35 vs 1.3, bcl-2/bax ratio; 2.3 vs 2.35, mcl-1/bax ratio; 1.1 vs 1.15 and mcl-1; 1.6 vs 1.5). Bcl-xl was only detected in 2 presentation cases. Bcl-xs was not detected in any of the samples tested. There was no correlation between levels of expression and event-free survival. Whereas *in vitro* sensitivity to prednisolone, determined in 30 patients using the 3-[4,5-dimethylthiazol-2,5-diphenyl] tetrazolium (MTT) assay, correlated with event-free survival there was no relationship between prednisolone or daunorubicin sensitivity and expression of bax, bcl-2/bax or mcl-1.This study suggests the measurement of expression of bcl-2, bax, bcl-xl, bcl-xs and mcl-1 is of limited prognostic value in childhood ALL.

This work was supported by the Leukaemia Research Fund

**P25** MITOCHONDRIAL CONTROL OF APOPTOSIS IN OVARIAN CARCINOMA CELLS.

*Aristides G. Eliopoulos, Maria Koffa, David J. Kerr and Lawrence S. Young. *Institute for Cancer Studies, University of Birmingham, Edgbaston, Birmingham B15 2TA, U.K.*

Ovarian cancer is one of the most fatal malignancies in women where resistance to chemotherapy often occurs. We have previously shown that the anti-apoptotic protein Bcl-2 may play a significant role in drug resistance in ovarian cancer. Thus, Bcl-2 is over-expressed in ovarian tumours and *cis*-platin-resistant ovarian tumour cell lines and its exogenous expression in A2780 ovarian cancer cells results in increased resistance to platinum. The anti-apoptotic properties of Bcl-2 have been associated with its ability to localise at the outer mitochondrial membrane and to regulate the mitochondrial membrane potential, $\Delta\Psi_m$. Loss of $\Delta\Psi_m$ is a central co-ordinating event of the apoptotic effector phase and involves formation of mitochondrial permeability transition pore (PT). We have found that Bcl-2-transfected A2780 cells possess constitutively elevated $\Delta\Psi_m$ levels compared to parental cells. Furthermore, whilst *cis*-platin treatment induces a rapid collapse of $\Delta\Psi_m$ in A2780 cells, Bcl-2 expression decreases this loss and delays apoptosis. Similar effects were observed in the *cis*-platin-resistant variant A2780CP which naturally over-expresses Bcl-2. Consistent with these phenomena, treatment of A2780 cells with the PT inhibitor bongkrekic acid (BA) protects against platinum-induced disruption of $\Delta\Psi_m$ and apoptosis. Thus, whilst a 14 hr treatment of A2780 cells with 50µM *cis*-platin induced a 73% loss in $\Delta\Psi_m$, addition of BA decreased this effect to 28%. Concomitantly, whilst 48% of platinum-treated A2780 cells stained positive for apoptosis, BA significantly decreased this number to 22%. Interestingly, both Bcl-2 expression and BA treatment delayed p53 accumulation in A2780 cells following exposure to *cis*-platin. As mitochondrial permeability transition pore opening may result in depletion of glutathione, a tripeptide with an important role in platinum resistance, we have examined the ability of Bcl-2 expression and BA treatment to stabilise glutathione levels. For this purpose, we determined the effects of the glutathione-depletion agent buthionine sulfoximine (BSO) on survival of Bcl-2-transfected or BA-treated A2780 cells using MTT conversion assays. In both cases a 3-4 fold increase in survival was noted, suggesting that modulation of thiol levels may be part of the mechanism by which Bcl-2 mediates drug resistance. Overall these data suggest a role for Bcl-2 in regulating mitochondrial membrane potential and platinum-induced apoptosis in carcinoma cells.

**P26** **Resistance to E1B attenuated adenoviral induced lysis of ovarian adenocarcinoma cell lines: cellular or viral change?**

Young Tae Kim*, Ian Ganly, Stanley Kaye and Robert Brown. CRC Dept of Medical Oncology, CRC Beatson Lab, Glasgow G61 1BD

The 55 kDa protein from the E1B region of adenovirus binds and inactivates p53. An E1B attenuated adenovirus (Onyx-015) can selectively replicate in and lyse cells with nonfunctional p53. Onyx-015 shows significantly higher replication in cisplatin resistant human ovarian adenocarcinoma cell line A2780/cp70 compared to the sensitive parental A2780 cell line. A2780/cp70 cells have previously been shown to have dysfunctional p53, while A2780 expresses wild-type p53. We have isolated 3 variants of A2780/cp70, A2780/cp70/vr1-3 which are resistance to Onyx-015 lysis at the concentration of 50 pfu. A2780/cp70 shows 100% Cytopathic Effect (CPE) at 10 pfu of Onyx-015 while the resistant lines show only 20% CPE at 10pfu. A2780/cp70/vr1 supports intracellular viral replication as determined by FACS analysis using an antibody to the adenovirus Hexon protein. A2780/cp70/vr1 is infected at equivalent efficiency with a lacZ reporter adenovirus Ad5CMVlacZ, compared to A2780 and A2780/cp70, demonstrating that there is no defect in the ability of these cells to be infected by adenovirus. To determine if resistance to lysis was due to loss of a viral lytic factor, we infected 293 cells with adenovirus isolated from A2780/cp70/vr1. This virus was able to produce CPE in 293 cells suggesting that the virus in A2780/cp70/vr1 retains its ability to lyse 293 cells. To determine if resistance to Onyx-015 was due to restoration of p53 function, p53 function was assessed by radiation induced G1 arrest assay, luciferase reporter assays, clonogenic sensitivity to cisplatin and Western immunoblotting against p53, p21, and E2F. These assays showed no restoration of p53 function and no inhibition of E2F induction in the A2780/cp70/vr1 line. Together this data suggests that the resistance to lysis in the A2780/cp70/vr1 cells is not due to restoration of p53 function, differences in the cells ability to be infected or mutation in the virus inhibiting viral induced cell lysis.

**P27** USING GENE THERAPY TO DETERMINE THE MECHANISM OF HYPOXIC ACTIVATION OF TIRAPAZAMINE,

E.C.Chinje*, M.P.Saunders, G.Franklin, N.Robertson, A.V.Patterson, I.J.Stratford, Experimental Oncology Group, Dept of Pharmacy, University of Manchester, M13 9PL.

The existence of regions of low oxygen tension in tumours is well documented and is a major reason for failure of radiotherapy and can cause resistance to some types of chemotherapy. Bioreductive drugs, such as tirapazamine(TPZ), are activated in hypoxic cells by a range of reductases including NADPH:P450 reductase (P450R). Under hypoxic conditions, TPZ is activated by one electron reduction to a cytotoxic radical intermediate prior to detoxification to further reduced species, including SR4317. It is proposed that these pro-drugs can be combined with radiotherapy or other forms of chemotherapy to target the hypoxic regions in tumours, whilst the conventional modality reduces the aerobic component. To gain a better understanding of the mechanism of activation and toxicity of TPZ, A549 human lung adenocarcinoma cells were exposed to escalating doses of TPZ over a six month period and clones were selected that were resistant to TPZ in air (Elwell JH et al, Biochem Pharmacol. 54,2,p249-257, 1997). Biochemical changes that may have accounted for the increased drug resistance included elevated levels of antioxidant enzymes and greatly diminished activity of P450R. We report here that one of the A549 clones that showed about a 40 fold reduction in P450R activity was also resistant to TPZ under hypoxic conditions. In order to determine whether this relationship was causal we transfected this clone with a vector containing P450R under the control of the moloney leukaemia virus LTR. A series of stable clones, with up to 200 times greater P450R activity than the parental clonal cell line, were generated. Using HPLC we have shown a significant relationship between P450R activity and SR4317 formation velocity in the transfected clones. Further, we have also shown that by artificially elevating the P450R activity, the hypoxic sensitivity to TPZ was restored. This provides further evidence that P450R is important in the hypoxic activation of this lead bioreductive drug, that is presently being evaluated in clinical trials. (MPS is a MRC Clinical training fellow).

**P28** USE OF TRIPLE HELIX-FORMING OLIGODEOXYNUCLEOTIDES AS A NOVEL THERAPEUTIC STRATEGY FOR ERB-B2 POSITIVE BREAST CANCER.

C Dolan*[1] A Wogan[1], D Hollywood[2], SR McCann[1], M Lawler[1], [1]Dept of Haematology, St James's Hospital, Dublin 8 and University of Dublin, Trinity College, Dublin 2, [2]Dept of Radiology, St Lukes Hospital, Dublin 6.

This study is an integrated approach to the development of new treatment strategies directed at specific genes involved in the pathogenesis of human breast cancer. Genetic abnormalities play a key role in the pathogenesis of breast cancer. If we could target these abnormalities and deregulate their expression, then we could have a powerful new therapeutic strategy against breast cancer. One gene that is aberrantly expressed in breast tumour tissue is the c-erbB2 oncogene. Breast cancer is the most common cancer found in European women (180,000 cases per year). It has been shown that patients who overexpress the c-erbB-2 proto-oncogene have aggressive tumours refractory to conventional therapies. Therefore, new therapeutic strategies are required for management of these high risk groups. We aim to downregulate expression of the erb-B2 gene by interfering with the transcriptional machinery of the c-erb-B2 promoter. We are evaluating a triplex strategy based on the ability to design molecules that bind to sequence-selected sites on DNA. These Triple helix-forming ODNs (TFOs) are small molecules designed to bind to the double helix of DNA in a specific manner and disrupt binding of proteins (transcription factors) which promote gene expression. We have identified a 28 base pair purine-rich region near the TATA box in the erb-B2 promoter which is a putative target for this triple helix approach. Specifically we have synthesised and tested a novel antigene oligodeoxynucleotides(ODNs) directed against this region within the erb-B2 promoter. Specific binding of the antisense TFO (when compared to nonsense or scrambled sequence TFOs) to the target promoter region has been achieved at physiological pH. Dissociation constants ($k_d$) for the selected TFOs has been determined using a concentration range of $10^{-11}$ to $10^{-7}$ M in Electromobility shift analysis (EMSAs) to permit selection of optimally binding TFOs and to define appropriate TFO concentrations to be used in subsequent DNA-binding protein/TFO competition experiments. EMSAs have indicated that the binding is strong and not easily reversible while DNase I Footprinting analysis has confirmed the exquisite specificity of binding to the erbB-2 promoter. Therefore, the use of antigene TFOs represents a highly specific method to target genes that play a role in the pathogenesis of breast cancer.

### P29 RUTHENIUM PHOTOSENSITISERS LINKED TO ANTISENSE ODNs - POTENTIAL FOR A MOLECULAR SCISSORS

[1]C O Keefe*. [2]M Lawler. [1]JM Kelly. Depts [1]Chemistry. [2]Hematology St James's Hospital/ Trinity College Dublin Ireland.

Antisense Oligodeoxynucleotides(ODNs) have great potential for specific targetting of genes involved in the development of human malignancy. One way in which antisense ODNs achieve their effect is through activation of the RNAse H cleavage pathway. However different ODN chemistries have variable abilities to promote RNAse H cleavage and an attractive option in antisense therapy would be to provide the antisense molecule itself with RNA or DNA cleaving activity. We have investigated the use of ruthenium photosensitisers as potential cleaving agents. Ruthenium molecules(Ru) are photo-activatable moieties which bind with a high dissociation constant to nucleic acids and when activated lead to strand cleavage. Initial experiments focussed on the use of free ruthenium(Ru-free) directed against a target ODN representing the junction region of the bcr-abl sequence(see below) observed in the majority of Chronic Myeloid Leukaemia patients. bcr-abl gives rise to the P210 protein which is impklicated in the pathogenesis of this disease. In all experiments described. radiolabelled target ODN was incubated with Ru-free and irradiated at 436nm using a Xenon arc lamp. Following irradiation. the products were analysed by denaturing polyacrylamide gel electrophoresis(PAGE). Incubations were performed in different buffers(containing different ions) and at differing salt conditions. Conditions were established which led to cleavage of the target ODN at Guanine (G) residues. Subsequently we have designed and synthesised a Ru-ODN to direct specific cleavage of the 24mer synthetic **bcr-abl** Target ODN) at residue G15 as indicated (underlined) in the *bcr*-**abl** junction sequence 5' *bcr TCAATAAGGAAG* **AA<u>G</u>CCCTTCAGC abl 3'** . The specificity of this Ru-ODN approach was confirmed by the observation of a single cleavage of the target ODN at G15 on PAGE. Thus we have established that ruthenium linked ODNs can bind to specific nucleic acid sequences and cleave these sequences in a highly specific manner. Current studies are focussing on the ability of these agents to direct cleavage of RNA under physiological conditions.

### P30 Benzoprims: dual action against DHFR and mutant Ki-*ras*

Stevens. M.F.G.[1]. Griffin. R.J.[2] and Richardson. M.L.[1] [1] Cancer Research Laboratories. University of Nottingham. NG7 2RD. UK and [2] Department of Chemistry. University of Newcastle. NE2 4HH. UK.

**Highly-substituted 2,4-diaminopyrimidines (benzoprims) as exemplified by methylbenzoprim 1 and dichlorobenzoprim 2 were developed as non-classical lipophilic analogues of methotrexate (MTX) and have potent inhibitory activity against mammalian DHFR (Ki = ~ $10^{-12}$M): the compounds have *in vivo* activity against the mouse M5076 reticulum cell sarcoma. a tumour resistant to MTX by virtue of modification of the folate transporter (Griffin et al. *J. Medicin. Chem.*, 1989. 32. 2468-2476). In certain cell lines (e.g. F28-7 murine breast) the inhibitory activity of 1 and 2 is reversed by hypoxanthine-thymidine or folinic acid. pointing to an antifolate locus of action. In confirmation in an NCI COMPARE analysis (59 cell panel) (Weinstein et al. *Science*. 1997. 275. 343-349) the highest Pearson Correlation Coefficients (PCC) were to MTX (PCC = 0.86) and trimetrexate (PCC = 0.83).**

**However there is a strong correlation (PCC = >0.75) with activity of benzoprims against mutant *ras* molecular target expression in a restricted panel of NSCL and colon tumour cell lines (n = 16). The following $GI_{50}$ values (µM) were obtained: in NSCL lines expressing wild-type *ras* — EKVX (1.78). HOP 92 (1.55). NCI-H522 (0.51): and lines expressing mutant Ki-*ras* — A549 (<0.01). NCI H23 (0.016). NCI-H460 (<0.01). A similar selectivity was observed against colon cell lines expressing mutant Ki-*ras* and this activity is not related to DHFR inhibition.**

**We have established a panel of lung. colon and pancreatic cell lines which have been characterised for *ras* expression (wild or mutant) and are seeking to redesign the lead benzoprim structure to identify a pure anti-*ras* molecule.**

### P31 PRECLINICAL STUDIES ON A NOVEL ANTITUMOUR AGENT. 2-(4-AMINO-3-METHYLPHENYL)BENZOTHIAZOLE.

M-S Chua*. I. Hutchinson. T. D. Bradshaw. C. S. Matthews. M. F. G. Stevens. Cancer Research Laboratories. University of Nottingham. Nottingham NG7 2RD.

2-(4-Amino-3-methylphenyl)benzothiazole (DF 203) is a potent and selective antitumour agent which demonstrates both *in vitro* and *in vivo* activity against breast, ovarian and colon cancer cell lines. It is COMPARE negative (Weinstein *et al*, 1997, Science, 275, 343) to all known classes of antitumour agents, suggesting a novel mechanism of action.

Sensitive breast cancer cell lines, MCF-7, MDA 468 and T-47D, rapidly take up and biotransform DF 203, while insensitive cell lines, MDA-MB-435, CAKI-1 and A498, do not. Metabolism studies may thus offer insights into its mechanism of action. Oxidation products (currently being characterised) from sensitive cell lines have been detected. However, *in vivo* pharmacokinetic studies in rats (dosed orally and intravenously) showed rapid conversion of DF 203 into a single *N*-acetylated metabolite (KF 498). No metabolites were detected in mice.

Ring hydroxylated and *N*-acetylated derivatives of DF 203 have been synthesised and their *in vitro* cytotoxicities evaluated in a panel of human tumour cell lines. These derivatives are largely inactive, with µM $IC_{50}$ values compared to the nM $IC_{50}$ values of DF 203 (Shi *et al*, 1996, J. Med. Chem., 39, 3375), suggesting these steps to be detoxifying.

The relative compositions of activating and detoxifying enzymes dictates the disposition and activity of many drugs and xenobiotics. In attempting to decipher the bioactivation pathway of DF 203, the compositions of common xenobiotic metabolising enzymes in sensitive, insensitive and resistant cells were examined by immunoblotting with specific antibodies. Cytochrome P450 (CYP)1A1 and CYP1B1, implicated in the critical *N*-oxidation of aromatic amines, were induced in MCF-7 cells and its acquired resistant variant MCF-7/LT 10µM, as well as MDA 468 cells. In contrast, glutathione *S*-transferase (GST)π, a vital detoxifying enzyme, was undetectable in these cells. *N*-Acetyltransferase (NAT)1 was observed at varying levels in all cell lines tested, although NAT2 was undetectable in MDA 468 cells. Intrinsically resistant HBL 100 cells lack CYP1A1 and CYP1B1 while possessing NAT1, together with enhanced levels of GSTπ. The implications of these observations are as yet unclear.

Efforts are underway to identify the antitumour entity of DF 203, which will help in mechanism elucidation. The ease of synthesis, potency and selectivity of DF 203 makes it a promising candidate for clinical trials.

### P32 ANTITUMOUR PROPERTIES OF NOVEL AZA-POLYCYCLIC COMPOUNDS

J. Stanslas[1], K.L. Marsh[2], C.A. Austin[2], R.A. Robins[3], M.R. Price[1], J.A. Double[4] and M.F.G. Stevens[1], [1]Cancer Research Laboratories, University of Nottingham, Nottingham NG7 2RD, England, [2]Department of Biochemistry and Genetics, Medical School, University of Newcastle, Newcastle NE2 4HH, [3]Division of Molecular and Clinical Immunology, Queens Medical Centre, Nottingham, [4]Clinical Oncology Unit, University of Bradford, Bradfrod BD7 1DP

Our studies on the synthesis of polycyclic acridines (eg **1**, R = H, Cl, Me, $NO_2$, $NH_2$) (Hagan et al, *J. Chem. Soc., Perkin Trans. 1, 279-2746, 1997*) indicate that these compounds bind to DNA in an intercalative mode at [Poly(dA-dT)]₂ sequences (Giménez-Arnau et al, *Anti-cancer Drug Design, in press*). A related polycyclic acridinium salt 1*H*,8*H*-2,3-dihydroindolizino[7,6,5-*kl*]acridinium chloride **2**, displays intriguing biological properties.

The *in vitro* antitumour screen by the National Cancer Institute of USA revealed a clear COMPARE activity profile of compound **2** to standard topo II inhibitors, with Pearson correlation coefficient values of more than 0.7. In the *in vitro* DNA-cleavage assay, compounds **1** and **2** failed to exhibit any selectivity for the recently discovered two distinct isoforms of topo II, α and β. They also did not show cytotoxicity preference in a panel of breast adenocarcinoma and non-small cell lung cancer (NSCLC) cells with various levels of topo II isoforms. Since these drugs, especially compound **2** damage DNA by poisoning topo II and also possibly by other DNA metabolism interfering mechanism, we have monitored the damage by analysing the cell cycle of drug treated breast cancer and NSCLC cells. Compound **2** induces $G_1$ phase accumulation in the wild-type p53 protein expressing cells, and also blocks cells in S and $G_2$ phases of cells expressing either wild-type or mutant p53 protein. The DNA damage causes upregulation of wild-type p53 protein and this in turn activates the cyclin-dependent kinase (cdk) inhibitor $p21^{CIP1/WAF1}$ protein expression, independent of the p53 protein levels. Compound **2** also has the interesting property of down-regulating (SKBr-3 and T47D) and upregulating (MDA-468) mutant p53 protein, especially in the high mutant p53 protein expressing breast cancer cells. Compound **2** is a potent inducer of apoptosis in breast and lung cancer cells, and this potential is independent of the p53 status, but very much dependent on the expression of the survival gene, bcl-2. Cells that express low level of bcl-2 readily undergo apoptosis, whereas high bcl-2 expressing cells (eg MCF-7wt), are very resistant to apoptosis. Preliminary study shows that compound **2** has the ability to retard the growth of chemoresistant MAC15A mouse colonic tumour *in vivo*.

### P33 17-(3-PYRIDYL)SUBSTITUTED IRREVERSIBLE INHIBITORS OF CYTOCHROME P450$_{17\alpha}$.

S.E.Barrie[1*], G.A.Potter[2], M.Jarman[1]. [1]CRC Centre for Cancer Therapeutics, Institute of Cancer Research, Sutton, Surrey, SM2 5NG. [2]De Montfort University, The Gateway, Leicester LE1 9BH.

We are developing inhibitors of the key enzyme in the androgenic biosynthetic pathway, cytochrome P450$_{17\alpha}$ (17α-hydroxylase/$C_{17,20}$ lyase) as potential drugs for the treatment of hormone dependent prostate cancer. Our lead compound, abiraterone (17-(3-pyridyl) androsta-5,16-dien-3β-ol), when tested as its O-acetate prodrug *in vivo* in mice, reduced circulating androgens and caused regression of androgen dependent organs. Studies with the enzyme from murine and human testes showed that abiraterone is a slow binding "irreversible" inhibitor. The extent of inhibition increased with time and appeared enhanced by preincubation with the enzyme. The apparent first order rate constant for the inhibition of the human enzyme was dependent on the concentration of abiraterone, being 0.14 ± 0.03 min$^{-1}$ with 5nM and 1.2 ± 0.3 min$^{-1}$ with 20nM abiraterone (in the presence of 1μM pregnenolone), and was inversely dependent on the pregnenolone concentration in the assay, being 1.2 ± 0.3 min$^{-1}$ at 1μM and 0.40 ± 0.03 min$^{-1}$ at 3μM pregnenolone (in the presence of 20nM abiraterone). This is as expected if the inhibitor competes with the substrate for binding at the active site. However, the rate constant was unaffected by the concentration of the cofactor NADPH. Once inhibited by abiraterone, the extent of inhibition was unaffected by the concentration of substrate in the assay and the measured IC$_{50}$ was 1.2nM. Furthermore the inhibition was subsequently unaffected by 24hr dialysis. The exact mechanism of the enzyme-abiraterone interaction is unknown. However, knowledge of the irreversible inhibitory behaviour of abiraterone is important for its clinical scheduling.

### P34 DEVELOPMENT, STRUCTURE-ACTIVITY RELATIONSHIPS AND ANTI-TUMOR ACTIVITY OF A NOVEL CLASS OF SPECIFIC, IRREVERSIBLE EGFR TK INHIBITORS.

J.B. Smaill*[1], W.A. Denny [1], D.W. Fry [2], J.M. Nelson [2], W. R. Leopold [2], P.W. Vincent [2], S. J. Patmore [2], W. L. Elliott [2], K.E. Hook [2], A.J. Bridges [2], H. Zhou [2], H.D.H. Showalter [2], D.J. McNamara [2], E.M. Dobrusin [2]. [1]Auckland Cancer Society Research Centre, Faculty of Medicine and Health Science, The University of Auckland, Auckland, New Zealand and [2]Parke-Davis Pharmaceutical Research, Division of Warner-Lambert Co., Ann Arbor, MI 48105.

Inhibition of the high levels of epidermal growth factor receptor tyrosine kinase (EGFr TK) activity frequently observed in a variety of human tumors may have a potential therapeutic benefit. To date a number of potent, reversible ATP competitive inhibitors of EGFr have been reported. High levels of intracellular ATP and a high rate of new receptor synthesis in some cell lines suggest that irreversible inhibition of EGFr autophosphorylation may be advantageous for good anti-tumor activity. This rationale has led to the design and synthesis of the 7-acrylamido-4-anilinoquinazoline, PD 160678, which is a sub-nanomolar, selective, irreversible inhibitor of the EGFr TK. Subsequent structure activity studies have established that introduction of the acrylamide functionality at the 6-position, as in PD 168393, is optimal for irreversible inhibition. Analogues possessing soluble cationic side-chains at the 7-position, such as the morpholinopropyloxy derivative PD 169540, are also potent, irreversible EGFr inhibitors showing significant anti-tumor activity *in vivo* using both IP and PO dosing protocols.

PD 160678 PD 168393

PD169540

### P35 p-MENTH-1,8-DIEN-10-OIC ACID, A NOVEL METABOLITE OF LIMONENE WITH INHIBITORY ACTIVITY AGAINST FARNESYL-TRANSFERASE AND GERANYLGERANYLTRANSFERASE I.

M.G. Rowlands *, R.M. Grimshaw, M.K. Mohan, B.P. Nutley, A. Moreno-Barber, I.R. Hardcastle and M. Jarman. CRC Centre for Cancer Therapeutics at the Institute of Cancer Research, Cotswold Road, Sutton, Surrey SM2 5NG, UK.

The monoterpene, limonene, is found as its R-enantiomer in orange peel and other plant oils. R-Limonene causes regression of both spontaneous and chemically induced tumours in rats and is in clinical trials in advanced cancer patients. R-Limonene undergoes extensive metabolism *in vivo* and in human plasma, R-perillic acid and its non-chiral reduction product, dihydroperillic acid are among the most abundant metabolites, whilst the glucuronide conjugates of R-perillic acid and R-perillyl alcohol are found in urine. Quantification and characterisation of the metabolites of limonene is important, because the known Phase I metabolites are more potent inhibitors of small G protein isoprenylation than limonene. Using HPLC-atmospheric pressure chemical ionization mass spectrometry (LC/APCI/MS), a novel metabolite has been detected in both human plasma and urine, which appeared to be an isomer of perillic acid (Poon et al., 1996 *Drug Metabolism and Disposition* **24**: 565).

The structure has now been confirmed by chemical synthesis. 4-Acetyl-1-methylcyclohexene was converted into its triflate by base catalysed reaction with triflic anhydride in the presence of the hindered base, 2,6-di-tert-butyl-4-methyl pyridine. The triflate was methoxycarbonylated to give p-menth-1,8-dien-10-oic acid, which by LC/APCI/MS, was identical with the novel metabolite detected in human plasma. Previous reports on the activity of perillic acid against isoprenylation enzymes refer to the S-enantiomer, although it is the R form which is produced from R-limonene. In this study, we also describe the synthesis of R-perillic acid via a two-step oxidation from the corresponding R-perillyl alcohol. The above monoterpenes were tested for inhibitory activity towards farnesyltransferase (FTase) and geranylgeranyltransferase (GGTase I) using published procedures. Rat brain cytosol provided a source of the enzymes, with H-Ras (WT) as protein acceptor for FTase and H-Ras (CVLL) for GGTase I. The results as IC$_{50}$ values in mM are shown below.

| | FTase | GGTase I |
|---|---|---|
| R-limonene | >40 | >40 |
| R-perillic acid | 8.1 ± 1.0 | 3.4 ± 0.3 |
| R-perillyl alcohol | 10.4 ± 1.5 | 2.1 ± 0.4 |
| p-menth-1,8-dien-10-oic acid | 5.0 ± 0.8 | 2.6 ± 0.4 |

In conclusion, p-menth-1,8-dien-10-oic acid is a major metabolite of limonene in humans with similar inhibitory activity towards the isoprenylation enzymes as the known metabolites, R-perillic acid and R-perillyl alcohol.

### P36 ANALYSIS OF CELL CYCLE EFFECTS AND SUBSEQUENT DEATH OF NON-SMALL CELL LUNG CANCER CELL LINES AFTER SHORT-TERM MITOMYCIN C EXPOSURE,

N.Robertson* and I.J,Stratford. Experimental Oncology Group, School of Pharmacy and Pharmaceutical Sciences, University of Manchester, Manchester M13 9PL.

Mitomycin C (MMC) is used clinically to treat a wide variety of solid tumours, and is the single most active agent for the treatment of non-small cell lung cancer (NSCLC).

For this study, 3 NSCLC cell lines have been chosen, H460, A549 and H647, which show a 73-fold range in their response to MMC as measured by the MTT assay after 3 hr drug exposure ( mean IC50 values of 0.18 μM, 3.55 μM and 13.1 μM respectively), but show little difference when assessed by colony-forming assay ( mean IC90 values of 0.11 μM, 0.19 μM and 0.17 μM respectively). These cell lines show similar levels of enzymes previously implicated in the metabolism, or detoxification, of MMC, and we have shown that the DNA damage formed after MMC exposure is equivalent (Robertson & Stratford, Br.J.Cancer, **75**, Suppl.I (27),1997), therefore further investigation was deemed appropriate.

Cell cycle effects after 3 hrs MMC exposure (1.5 μM) have been assessed using FACS analysis of fixed propidium iodide-stained cells.All three cell lines exhibit $G_2$ block 24 hrs post MMC exposure but vary in their subsequent response. H647 cells are attempting to divide by Day 3 , and then rapidly die. Some H460 cells persist in $G_2$ block for at least 7 days before dying, but a considerable number of A549 cells remain in apparently irreversible $G_2$ block, or as large cells, for at least 21 days after drug exposure (NB. Control cells maintained for this period were seen to be cycling normally).

In an attempt to gain further information with regards to the death of these cells after exposure to MMC a number of methods were employed. Estimation of total cell number over 21 days shows H647 and H460 cell numbers to fall far more rapidly than A549 cells as would be expected. Exclusion of propidium iodide combined with Rhodamine 123 uptake by the cells (analysed by FACS) allows a simultaneous measure of cell viability and of mitochondrial membrane potential (alterations associated with onset of apoptosis). Marked differences were apparent between the 3 cell lines with respect to changes in mitochodrial function, and the percentage of necrotic cells present at each timepoint, but there was no evidence of apoptosis using this technique (We are currently attempting to confirm these results using an assay of Annexin V-binding. Results will be presented).

In conclusion, the 3 NSCLC cell lines react very differently to short term MMC exposure but it would appear that their overall long term survival is the same at this drug concentration, and that the colony forming assay reflects this more accurately than the MTT assay. These results suggest that the MTT assay is not a suitable measure of cellular sensitivity for drugs, such as MMC, which cause cell cycle arrest over prolonged periods

### P37 SEQUENTIAL TOPOTECAN (TOPOISOMERASE I INHIBITOR) AND ETOPOSIDE (TOPOISOMERASE II INHIBITOR) IN ADVANCED, ADULT SOLID TUMOURS,

J.V. Watson, V.R. Bulusu*, A. Deary, Primrose Oncology Centre, Bedford Hospital, Bedford, MK42 9DJ, UK.

Topoisomerase I (topo I) inhibition is associated with upregulation of topoisomerase II (topo II) enzyme. Sequential administration of a topo I inhibitor followed by a topo II inhibitor has been shown to be synergistic, both in vitro and in vivo. We explored the feasibility of this concept in advanced, adult solid tumours with sequential administration of topotecan and etoposide.

**Aim**: To document the feasibility, toxicity and activity of sequential administration of iv topotecan and oral etoposide in adult solid tumours.

**Methods**: 8 patients(pts) with advanced solid tumours (breast-4, ovary-3 mesothelioma-1) have been treated with the sequential protocol as of January, 1998. Eligible pts had histologically confirmed malignancy, normal haematology, renal and hepatic functions and a WHO performance status of ≤2. Mean age 50 years, range 29-65 years. All pts had atleast two standard chemotherapy regimens prior to entry into the protocol. 2 of the 3 ovarian cancers were refractory/resistant to platinum and paclitaxel. Administration schedule: Topotecan, 1.2mg/m$^2$/day, 30 min infusion on days 1-2 followed by Etoposide 150mg/m$^2$/day po on days 5-7 in non-ovarian cancer pts and on days 8-10 in ovarian cancer pts, cycle repeated every 28 days. Toxicity documented according to common toxicity criteria. All patients had antibiotic prophylaxis with oral ciprofloxacin and fluconazole from day 7 to day 16.

**Results**: Total number of cycles administered n=22 (Mean 2 and range 2-6) and data from 21 cycles is available for toxicity assessment. The dose limiting toxicity (DLT) was neutropenia. Grade ≥3 neutropenia was observed in 43% (95% CI 33.3-52.7) cycles, and in 19% (4/21) cycles, hospital admission was needed for neutropenic fever. Grade ≥ 3 anemia and thrombocytopenia were infrequent, observed in 5%(1/21) and 9.5% (2/21) cycles respectively. Schedule delayed in 24% (5/21) cycles, only one due to delayed marrow recovery. Absolute neutrophil count (ANC) nadir mean on day 10 (95% CI 7.7-13.2). Interestingly, ANC rebound ≥ 10$^9$/l was noted in 19% (4/21) of the cycles. Transient nausea was controlled by oral antiemetics. There were no treatment related deaths. So far 8 pts completed at least 2 cycles and are available for response assessment. No complete or partial responses were observed. Stabilisation of disease was documented in 4 pts (2 with breast and 2 with ovarian cancer). 1 pt with platinum and paclitaxel refractory ovarian cancer completed 6 cycles with stabilisation of evaluable disease, serum markers and had subjective response.

### P38 COMPARATIVE EFFECTS OF CISPLATIN AND MELPHALAN ON TELOMERASE ACTIVITY IN A TESTICULAR CELL LINE.

T.R.Cressey, M.J.Tilby*, D.R.Newell, Cancer Research Unit, University of Newcastle., Newcastle Upon Tyne, NE2 4HH

Telomerase is a ribonucleoprotein reverse transcriptase which adds telomeric repeat units of 5-(TTTAGGG)-3' onto the ends of human chromosomes. In non-transformed cells, telomeres, the specialised region at the end of the chromosome, shorten after successive replications. Extreme attrition of the telomeric region appears to cause initiation of cellular senescence. Activation of telomerase is thought to be one of the precursors for the immortalisation of cells.

It was previously reported that cisplatin inhibits telomerase while other DNA damaging agents, such as melphalan and transplatin, had no effect ( Burger et al, Eur. J. Cancer, **26**, p638, 1997 ). However, in another report, decreased telomerase activity was associated with non-specific tumour cell killing 'in vitro' ( Faranoi et al, Clin. Cancer Res, **3**, p579, 1997 ). The objectives of the present study were to confirm the specific inhibition of telomerase by cisplatin and investigate the underlying mechanism.

Using the testicular cell line Susa CP, telomerase activity was measured using the Telomeric Repeat Amplification Protocol (TRAP assay).

Following a 4 hour exposure of Susa CP cells to 100μM cisplatin or melphalan and growth in drug free medium for a further 20 or 44 hours, telomerase activity was measured. After 24 hours incubation, telomerase activity was found to be reduced by cisplatin but in contrast, there was no evidence of such a decrease following melphalan, in agreement with previous studies. However, after 44 hours in drug free medium, telomerase activity was reduced after treatment with cisplatin or melphalan. This suggests the rate of loss of telomerase activity may be drug dependent.

Growth inhibition of Susa CP cells using the Sulphurhodamine B assay ( SRB ) showed the $IC_{50}$ of cisplatin and melphalan to be 0.73μM and 0.80μM respectively. Thus although the drug doses used to study telomerase inhibition were high, each was used at equivalent toxicities (100x its $IC_{50}$). Furthermore, at the drug exposures used, the time courses for apoptosis induction were found to be similar for both drugs. Thus the differential effect on telomerase inhibition may not simply be due to different overall rates of cell death.

### P39 THE EFFECT OF APHIDICOLIN, AN INHIBITOR OF POLYMERASES, IN HUMAN OVARIAN CARCINOMA CELLS WITH DIFFERING MISMATCH REPAIR STATUS AND CISPLATIN SENSITIVITY.

N. Moreland and R. Brown, CRC Department of Medical Oncology, CRC Beatson Laboratories, Switchback Road, Glasgow, G61 1BD.

Recent observations suggest mismatch repair (MMR) proteins provide a link between cisplatin induced DNA damage and apoptotic responses in a variety of carcinoma cells. It has been proposed that in the absence of MMR intrastrand crosslinks are bypassed during replication, giving a cisplatin resistant phenotype.

We have used aphidicolin (Ap), an inhibitor of polymerases α, δ and ε, as a tool to study the role of polymerases in cisplatin resistance. The cell lines used were A2780, a MMR proficient, cisplatin sensitive ovarian line, A2780/cp70, a cisplatin resistant derivative of the A2780 line defective for MMR, and the chromosome transfer lines, cp70-ch3 and cp70-ch2. Cp70-ch3 are stable chromosome 3 transfers which exhibit restored MMR and cisplatin sensitivity, while cp70-ch2 are stable chromosome 2 transfers which remain defective for MMR and cisplatin resistant. The $IC_{50}$ values for 24h exposures to Ap were 1.9, 2.4, 3.1 and 2.1 μM for the A2780, A2780/cp70, cp70-ch3 and cp70-ch2 lines, respectively, as measured by clonogenic assay. These $IC_{50}$ values did not correlate with MMR status or cisplatin sensitivity but rather to the proportion of S-phase cells present at the time Ap exposure was begun ($r^2$ = 0.79). This suggested a line with a higher basal level of S-phase cells has increased polymerase activity and requires higher concentrations of Ap to inhibit this activity.

To examine the effect of polymerase inhibition after cisplatin treatment in MMR proficient and deficient cells, clonogenic assays employing a combination of cisplatin and Ap have been performed in the cp70-ch3 and cp70-ch2 lines. A 1 hr exposure to cisplatin (20 μM) resulted in decreases in surviving fractions of 0.40 ± 0.05 and 0.70 ± 0.04 in cp70-ch3 and cp70-ch2 cells, respectively. The addition of an $IC_{50}$ concentration of Ap after cisplatin treatment decreased the surviving fractions further to 0.13 ± 0.01 and 0 22 ± 0.09, respectively. This data suggested a more pronounced effect of Ap in sensitising MMR deficient cells to cisplatin. Analysis of drug resistance in cp70-ch2 compared to cp70-ch3 after treatment with cisplatin alone or cisplatin in combination with Ap revealed the addition of Ap caused a significant decrease in the fold-resistance values (p = 0.003). This indicated that inhibition of polymerases after cisplatin lesions are produced reduces resistance in MMR deficient cells and supports the hypothesis that increased replicative bypass is involved in cisplatin resistance.

### P40 PREFERENTIAL INDUCTION OF APOPTOSIS IN MULTIDRUG RESISTANT CELLS BY PDMP, AN INHIBITOR OF CERAMIDE METABOLISM

K.M. Nicholson*, D.M. Quinn, G.L. Kellett and J.R. Warr. Dept. of Biology, University of York, PO Box 373, York, YO1 5YW

A novel approach by which multidrug resistance may be circumvented involves exploiting biochemical differences between multidrug resistant (mdr) and sensitive cells to selectively kill mdr cells. Several changes in lipid composition between mdr and sensitive cells have been identified, with recent reports suggesting that mdr cells have higher levels of glucosylceramide than sensitive cells which may function in the maintenance of mdr (Lavie *et al.*(1996) *J.Biol.Chem.* 271: 19530-19536). Glucosylceramide is formed from ceramide, a second messenger involved in apoptosis induction, by glucosylceramide synthase. Therefore, this study aimed to investigate the effects of glucosylceramide and ceramide on a series of mdr cells by altering ceramide metabolism with PDMP, an inhibitor of glucosylceramide synthase. The sensitive cell line, KB-3-1 and its mdr counterparts, KB-C1, KB-A1 and KB-V1 cells were exposed to PDMP. Viability was assessed using a colony formation assay and the mean D10 values (concentration required to reduce the colony forming ability of cells to 10% of controls) of 38.47 ± 6.11μM for KB-3-1 cells, 15.11 ± 2.63μM for KB-C1 (t-test, p<0.01), 32.26 ± 11.98μM for KB-A1 and 21.44 ± 5.97μM for KB-V1 cells (p<0.01) suggest that mdr cells were preferentially killed by PDMP. To determine the mechanism of cell death behind this preferential killing in terms of induction of apoptosis or necrosis, the nuclear morphology of cells was assessed using Hoechst 33342, Annexin-V and propidium iodide labelling following a 24 hour exposure to 75μM PDMP (see table). It is apparent that the main mechanism behind cell death in the mdr cells is via an induction of apoptosis.

| | Mean % of cells ± SD | | |
|---|---|---|---|
| Cell Line | Viable | Apoptosis | Necrosis |
| KB-3-1 | 77.07 ± 6.23 | 13.69 ± 4.74 | 9.24 ± 4.71 |
| KB-C1 | 53.79 ± 11.63 (p<0.01) | 34.85 ± 9.68 (p<0.01) | 11.74 ± 4.06 |
| KB-A1 | 49.49 ± 16.14 (p<0.01) | 43.49 ± 16.92 (p<0.01) | 7.02 ± 2.35 |
| KB-V1 | 38.49 ± 16.04 (p<0.01) | 54.59 ± 16.26 (p<0.01) | 7.47 ± 2.04 |

A timecourse of apoptosis induction and poly-(ADP-ribose)-polymerase cleavage was followed and it was apparent that apoptosis was initiated after approximately 6 hours of PDMP treatment in the mdr cells. Thus, altering ceramide metabolism may be beneficial in the selective killing of mdr cells since these cells, with their increased dependence on glucosylceramide, are more susceptible to apoptosis.

The authors wish to thank Yorkshire Cancer Research for their support.

### P41 OVERCOMING CISPLATIN RESISTANCE WITH THE BIOREDUCTIVE DRUG TIRAPAZAMINE.

J.E. Monaghan* and I.J. Stratford. School Of Pharmacy And Pharmaceutical Sciences, University Of Manchester.

The presence of low oxygen conditions (or hypoxia) in solid tumours is a major hindrance to current therapies, rendering tumour cells resistant to both radiotherapy and many chemotherapies. Tirapazamine, TPZ (SR4233, Win 59074). is a benzotriazene-di-N-oxide which under hypoxic conditions is reduced to a free radical capable of abstracting hydrogen from DNA causing single and double strand DNA breaks. Thus, TPZ is selectively toxic towards hypoxic cells and it is currently in clinical trials in combination with cisplatin. These trials are based on pre-clinical work of Dorie and Brown (1993, 1997) who demonstrated substantial synergistic interaction between these two agents. However, in these studies there is no indication whether cells inherently resistant to cisplatin could be specifically sensitized by prior treatment with TPZ. Therefore, we have examined the effect of the drug combination on a panel of five breast cancer cell lines that show a 50 fold range in cisplatin sensitivity ($IC_{50}$ values ranging from 9 to 530μM following a 1 hour exposure). Cells have been exposed to varying concentrations of TPZ for 3 hours in hypoxia, then allowed to reoxygenate for two hours prior to treatment with varying concentrations of cisplatin for 1 hour in air. The MTT cell proliferation assay was used three days later to determine values of $IC_{50}$. Data has been analyzed by assessing the change in hypoxic TPZ $IC_{50}$ at concentrations of cisplatin ranging from 1 to 10μM. Generally $IC_{50}$ values decrease (i.e. cells become more sensitive) as the cisplatin dose increases. In MDA 231 tumour cells, the $IC_{50}$ dose for aerobic exposure to cisplatin for one hour is 471.0 ± 52.7 μM. A 10μM dose reduces the tirapazamine $IC_{50}$ of this cell line four-fold. In contrast, in the cisplatin-sensitive cell line MDA 468 ($IC_{50}$=9μm), the 10 μm cisplatin dose is required to reduce the tirapazamine $IC_{50}$ 4-fold. Thus, at an equal dose of cisplatin the synergistic interaction between TPZ is similar in cell lines with widely varying cisplatin sensitivity. However, it is likely that, if viewed on the basis of equal cisplatin toxicity, those cells showing greatest resistance to cisplatin will show the greatest synergistic effect when they are treated with both drugs. Experiments to confirm this are currently being carried out.

### P42 ACCENTUATION OF TNF-INDUCED APOPTOSIS IN HELA CELLS USING INHIBITORS OF THE CHYMOTRYPTIC ACTIVITY OF PROTEASOME,

B. Walker*[1], A. Healy[2] and J.F. Lynas[2], [1]Dept. of Biochemistry, National University of Ireland, Galway, Ireland, [2]School of Biology and Biochemistry, The Queen's University of Belfast, BT7 9BL, Northern Ireland.

The pluripotent cytokine, TNFα, is known to cause apoptosis in tumour cells containing its cognate p55 receptor. However, the extent of apoptosis in these target cells is attenuated by the activation and nuclear translocation of the transcription factor NFκB, processes that are also initiated by the binding of TNFα to p55. The key steps in the activation and translocation of NFκB are the phosphorylation and subsequent proteasome-mediated proteolysis of a protein called IκBα, which binds to and sequesters this transcription factor in the cytoplasm.
The inhibition of the proteasome-mediated proteolysis of IκBα would therefore result in the blockade of NFκB nuclear translocation and, presumably, the NFκB-induced expression of the protective genes which limit the extent of apoptosis (1,2).
We have developed a series of peptidyl α-keto aldehyde inhibitors of the chymotryptic activity of proteasome, one of which Bzl-Leu-Leu-Leu-COCHO (**I**), inhibits the enzyme with a $K_i$ of ~ 3.0 nM (3).
We now wish to report that this inhibitor greatly accenuates the TNFα–induced apoptosis in HeLa cells, when used at concentrations of 10 μM (see Table).

| | Treatment | | | |
|---|---|---|---|---|
| | Control | TNF | TNF+CHX | TNF+I |
| No of cells undergoing apoptosis (%) (mean of 3 exps.) | 1.5 | 1.3 | 26 | 11 |

TNF used at 60ng/ml; CHX used at 20 ng/ml; **I** used at 10 μM. HeLa cells were exposed to treatments for 8 hr, fixed and then treated with Hoechst 33342 dye to count apoptotic cells.

Beg, A.A. & Baltimore, D. (1996) *Science* **274**, 782.
Van Antwerp, D.J., Martin, S.J., Kafri, T., Green, D.R. & Verma, I.M. (1996) *Science* **274**, 787.
Lynas, J.F., Harriott, P., Healy, A., McKervey, M.A. & Walker, B. (1998) *Bioorg. Med. Chem. Letts.* In Press.

### P43 XR9576, A NOVEL POTENT MODULATOR OF P-GLYCOPROTEIN-MEDIATED MULTIDRUG RESISTANCE.

W. Dangerfield, P. Mistry, A.J. Stewart, D. Bootle, C. Liddle, S. Okiji, D. Templeton. Xenova Ltd, Slough, SL1 4EF, UK.

P-glycoprotein (P-gp) mediated multidrug resistance (MDR) is a major obstacle to the successful treatment of cancer. This current study demonstrates the efficacy of XR9576, a novel potent inhibitor of P-gp. XR9576 (an anthranilic acid derivative) was able to sensitize a panel of human and murine MDR cell lines derived from various tumour types (EMT6 AR1.0, H69/LX4, 1847AD, 2780AD, MC26) to a broad range of clinically relevant cytotoxics (doxorubicin, vincristine, taxol, etoposide, actinomycin D and colchicine). In cytotoxicity assays over 4-6 days, XR9576 fully reversed resistance when used at 30-100 nM, and half maximal reversal was seen at 12-40 nM. By contrast, PSC 833 which is currently undergoing clinical trials in the treatment of MDR cancer, showed maximal effects at 250-500 nM. In transport studies, inhibition of P-gp dependent [$^3$H]-daunorubicin efflux was achieved at low nanomolar concentrations, and half maximal reversal of the accumulation deficit was seen at 38 ±18 nM. This activity was maintained for in excess of 22 hours after removal of XR9576 from the incubation medium when cells had been preincubated with 200 nM XR9576. This prolonged duration of action suggests that, unlike, cyclosporin A or verapamil, XR9576 is not transported by P-gp. XR9576 does not reverse drug accumulation deficit in a multidrug resistance-associated protein (MRP) expressing NSCLC cell line (COR-L23/R). This indicates that XR9576 is a selective inhibitor of P-gp.
*In vivo*, XR9576 showed efficacy in mice bearing both murine and human tumours. XR9576 (either p.o. or i.v.; 2.5 to10 mg/kg) significantly enhanced the effect of doxorubicin compared to doxorubicin alone in the intrinsically resistant MC26 tumour model. In athymic mice bearing human H69/LX4 MDR tumours, XR9576 (p.o. or i.v.; 4-24 mg/kg) significantly potentiated the antitumour activity of etoposide. Furthermore, comparison of body weights indicated that the combination schedules were well tolerated.
These studies demonstrate XR9576 to be an extremely potent and effective modulator of P-gp mediated MDR and holds great promise in the treatment of refractory cancers.

### P44 SELECTIVE SENSITISATION OF TUMOUR CELLS TO THE INDUCTION OF APOPTOSIS WITH INHIBITORS OF PI3-KINASE AND PKC.

D. O'Gorman, A. McGahon and T.G. Cotter, Dept. of Biochemistry, University College Cork, Ireland.

The induction of apoptosis is the mechanism through which a variety of cytotoxic drugs kill tumour cells. The precise sub-cellular pathway through which any of these drug initiates apoptosis is as yet unclear. In the present communication we demonstrate that the use of selective protein kinase inhibitors sensitises HL-60 tumour cells to the induction of apoptosis. In this respect Wortmannin and LY294002, both inhibitors of PI3 kinase, enhance the induction of apoptosis in these tumour cells by up to two fold. These data indicate that PI3 kinase is essential for cell survival and when inhibited an enhancement of the rate of apoptosis induced in this case by actinomycin D, Camptothecin, etoposide and cycloheximide is seen. This is in line with some recently published work indicating a role for PI3 kinase as an anti-apoptotic survival factor. Our data also indicates that PI3 kinase does not mediate its survival effects through the p70S6 kinase pathway. Protein kinase C (PKC) like PI3 kinase has also been suggested to play a key role in the regulation of apoptosis. Using a series of PKC inhibitors including PMA, staurosporine and calphostin C we were able to demonstrate that inhibition of PKC by calphostin C, but not the other two agents, increased the sensitivity of HL-60 cells by 2-3 three fold to the induction of apoptosis by a number of cytotoxic agents. Experimental data suggests that calphostin C may act by its selective inhibition of PKC zeta. The use of PI3 kinase or selective PKC inhibitors in conjunction with cytotoxic agents may open a novel therapeutic strategy for the killing of tumour cells.

The work was supported by the Irish Cancer Society, Health Research Board of Ireland and The Children's Leukemia Research Project.

KEY WORDS : apoptosis, HL-60, PI3 kinase, PKC

### P45 CHEMOTHERAPY INDUCES APOPTOSIS IN HUMAN RETINOBLASTOMA. ENHANCEMENT BY SODIUM BUTYRATE.

MC Madigan*, RM Conway, G. Chaudhri, FA Billson and PL Penfold; Dept. Clinical Ophthalmology, Uni. of Sydney, Australia

Retinoblastoma (Rb), derived from retinal neuroepithelial cells, is the most common intraocular malignancy of childhood, and has traditionally been treated by enucleation or radiation of the affected eye. Recently, chemotherapy/cyclosporine A combined with laser or cryotherapy has been used as an alternative therapy for Rb. In this study, we assessed the effects of vincristine (VN) and cisplatin (CP) on cell death in human Rb cell lines (Y79 and WERI-Rb1), and investigated the potential of sodium butyrate (SB), which can induce dose-dependent apoptosis or differentiation in Rb (1), to augment chemotherapy-induced apoptosis.

The dose-response of Rb cell lines to VN and CP was established (LD50 1-10μM). Cultures treated with 10μM VN or CP were assessed using cell morphology, DNA stains and DNA electrophoresis. The effects of cycloheximide (CHM) and actinomycin D (AD), and endonuclease inhibitors zinc sulphate (Zn) and aurintricarboxylic acid (ATA) were also assessed. Rb cells were also exposed to combined chemotherapy/SB<2mM and examined as above.

VN and CP induced dose-dependent cell death in Rb cell lines, with features characteristic of apoptosis including morphological changes and DNA fragmentation. CHM and AD significantly reduced chemotherapy-induced apoptosis, although endonuclease inhibitors (Zn and ATA) had no apparent effect. Treatment of Rb cells with VN or CP combined with SB<2mM significantly enhanced cell death compared to chemotherapy alone.

Chemotherapy drugs induced dose-dependent apoptosis in Rb cell lines, which was enhanced when combined with SB. These observations may have implications for the treatment of Rb with chemotherapy.

1. Conway RM, Madigan MC, Penfold PL, Billson FA. Oncology Res 1995: 7; 289-297.

Supported by Sydney Foundation for Medical Research (MCM).

### P46 SURFACE - ATTACHED DNA FRAGMENTS FOR IMMUNOCHEMICAL ANALYSIS OF DRUG-DNA INTERACTIONS: APPLICATION TO MELPHALAN ADDUCTS.

McCartney, H., Martin, A.M. and *Tilby, M.J. Cancer Res. Unit, Newcastle upon Tyne, U.K.

*Background:* Two previously described monoclonal antibodies (MP5/73, Amp4/42) recognise adducts formed in polymeric DNA by the aromatic alkylating agent and anti-cancer drug melphalan. These antibodies are being used to study clinical and experimental samples but available evidence regarding their specificity was inconsistent. MP5/73 appears to recognise adducts at N7-guanine and Amp4/42, the alkali-induced ring opened N7-guanine adducts. *Objectives:* **1.** To establish the use of DNA fragments covalently attached to the surfaces of microtitre wells for detailed analysis of reactions of drugs with DNA, chemical modifications to the adducts and immunological detection of reaction products. **2.** To apply this approach initially to investigate the specificity of the antibodies against melphalan-DNA adducts. *Methods:* DNA fragments were chemically cross-linked, via terminal 5'phosphate, to micro-wells using a published method. After exposure to melphalan and then to NaOH solution for various times, antibodies were added, followed by β-galactosidase detection reagents to give a fluorescence signal. *Results:* If the hypothesised specificities of the antibodies were correct, alkali-induced loss of immunoreactivity to MP5/73 should occur at the same rate as gain of immunoreactivity to Amp4/42. In initial studies, DNA in solution was exposed to radioactive drug and then alkali. Analysis by radiochemical and competitive ELISA techniques indicated that gain of immunoreactivity to Amp4/42 occurred several fold faster than loss of immunoreactivity to MP5/73 (In 0.1M NaOH, the half-lives were estimated to be <3mins and 12 mins respectively). This was not consistent with the presumed antibody specificities. The use of surface attached DNA permitted more detailed, flexible and rapid investigations. Immunoassay signal was linearly dependent upon drug concentration in a 1h exposure, consistent with the signal being directly proportional to adduct level, however, alkali-induced reactions appeared slower than in free solution. A difference in rates of alkali-induced changes assayed by each antibody were seen but, after addition of 0.1M NaOH to wells carrying alkylated DNA, the change of immunoreactivity to MP5/73 exhibited a clear delay before decaying with a half-life of about 18mins. Gain of Amp4/42 immunoreactivity showed no delay and exhibited a similar half-life. The use of immobilised DNA and defined sequence oligonucleotides could be particularly useful for studies of delayed second arm reactions of bifunctional drugs, interactions with glutathione and effects of enzymes.

### P47 IRINOTECAN PLUS OXALIPLATIN ± G-CSF AS SECOND LINE THERAPY IN PATIENTS WITH ADVANCED 5-FU/LEUCOVORIN-REFRACTORY COLORECTAL CANCER (CC)

J.Valencak*, B.Lathan, G.Kornek, M.Raderer, G.Klein, M.Hejna, T.Brodovic, G.Weinländer, D.Depisch and W.Scheithauer Dept. of Int. Med.I., Univ. of Vienna, Lathan Inst.-Dortmund and Wr.Neustadt General Hospital, Austria.

Between May and December 1997, thirty-three patients (pts) with measurable metastatic CC that had relapsed (within 3 months after cessation) or progressed during palliative 5-FU/LV-based therapy were treated with a combination regimen, consisting of irinotecan 80 mg/m2 (days 1+8+15) and oxaliplatin 85mg/m2 (days 1+15) ±G-CSF (5mcg/kg given subcutaneously on 5 consecutive days depending on the actual granulocyte counts on days 1 and 15 of the cycle). Treatment courses were repeated every 4 weeks. 29 pts (12 female, 17 male) are currently evaluable for response and toxicity assessment. Their median age is 61 (31-70) years, and their median WHO performance status 1 (0-2); 11 had single and 18 had ≥2 metastatic sites. The median duration of 5-FU/LV 1st –line therapy was 1 month (range 1-6). After a median of 5 treatment cycles (range 2-6), nine partial responses (31 %) were observed, and 12 additional pts (41%) had stable disease. Median response duration, time to progression and survival time have not been reached yet. Haematologic side effects were commonly encountered, though they were generally mild to moderate [ leukocytopenia 26 pts, WHO grade 4: 2 pts (7%); thrombocytopenia 10 pts, WHO grade 3: 2 pts (7%)]. Dose-limiting side effects were mainly gastrointestinal [diarrhea: 22 pts, WHO grade 3: 6 pts (21%); emesis 21 pts, WHO grade 3: 5 pts (17%)] and required dose attenuations (i.e., deletion of irinotecan on day 8) in 9 cases (31%). Other adverse reactions comprised alopecia (19 pts), mild peripheral neuropathy (3 pts), phlebitis (2 pts), and minor infections (3 pts).

These encouraging preliminary response- and acceptable toxicity data warrant further clinical evaluation to define the role of combined irinotecan and oxaliplatin ± GCSF in the treatment of patients with advanced CC failing 5-FU/LV-based therapy.

### P48 PHASE II TRIAL OF THE LONG ACTING SOMATOSTATIN ANALOGUE LANREOTIDE IN PATIENTS WITH ADVANCED HEPATOCELLULAR CANCER.

M. Hejna*, G.V. Kornek, M. Raderer, J. Valencak, B. Lehnauer, W. Fiebiger, R. Greul, F. Lang, B. Schneeweiss, D. Depisch and W. Scheithauer. Dept. of Internal Medicine I, University Medical School, Vienna, & Dept. of Surgery, Wr. Neustadt, Baden, & Neunkirchen General Hospital, Austria

Treatment options for advanced hepatocellular cancer (HCC) are limited by the drug-refractory nature of this disease. Based on preliminary results, we have performed a phase II study of the long acting somatostatin (SST) analogue lanreotide (LAN) in 21 untreated patients with advanced HCC not amenable to surgery. Treatment consisted of 30 mg LAN administered by deep intramuscular injection every 14 days until documented disease progression. No hematologic side effects were seen with this application schedule, 3 patients each developed mild nausea and meteorism attributable to treatment, 1 patient had diarrhea WHO-grade 1, while 2 patients developed steatorrhea. Arthralgia, short lasting vertigo and erythema at the injection site was experienced by 1 patient each. Only 1 patient (5%) showed a partial response, 8 patients had stable disease (38%), while the remaining patients progressed during treatment. The median survival was 4.2 months (range 1.2-13+), and the median time to progression was 2.5 months (range 1.5-7+). However, 4 patients (19%) had an increasein WHO performance status lasting between 2.5 and 6 months, 5 patients (24%) had an increase in body weight, while pain markedly improved in 1 additional patient (5%). In total, 5 patients (24%) had a decrease in serum AFP-levels at least 30%. Despite the disappointing results in terms of objective response, we feel that a subjective benefit in roughly 35% is noteworthy in this group of patients. Further in vitro and in vivo investigations seem indicated to define the exact therapeutig potential of SST-analogues.

### P49 DEVELOPMENT OF A BLEOMYCIN POLYMERIC VESICLE FORMULATION

J Sludden*, AG Schatzlein, L Tetley[1], CJ Twelves, IF Uchegbu[2]. CRC Dept. of Medical Oncology, Garscube Estate, Switchback Rd., Glasgow G61 1BD. [1]Institute of Biomedical and Life Sciences, University of Glasgow, Glasgow G11 8QQ. [2]Dept. of Pharmaceutical Sciences, University of Strathclyde, George St., Glasgow G1 1XW.

Bleomycin (BLM) is a water-soluble glycopeptide used in combination chemotherapy against germ cell tumours such as testicular teratoma and ovarian cancer, resulting in a 90% response rate in the case of the former. However treatment with BLM is hampered by a serious pulmonary fibrosis occurring in between 5% and 10% of patients with a 2% mortality rate (Levi et al (1993) *J Clin Oncol* **11** 1300). This can lead to the death of potentially curable patients and is related to cumulative dose, renal impairment, adjunctive classic radiotherapy, the use of supplemental oxygen or the patient being aged over 70 (Comis (1992) *Sem Oncol* **2** S5 64; Jules-Elysee and White (1990) *Clin in Chest Med* **11** 1). Classic studies have shown that continuous infusion is more effective and less toxic than intermittent treatment (Cooper and Hong (1981) *Cancer Treat Rep* **65** 419). Previous pre-clinical studies have shown that doxorubicin vesicles circulate in the blood for prolonged periods following intravenous administration and provide a depot of drug, resulting in improved tumoricidal activity (Uchegbu et al (1995) *Pharm Res* **12** 1019). The objective of the current study is to formulate a controlled release particulate BLM formulation in an effort to reduce the toxicity of this drug.

BLM polymeric vesicles were prepared from palmitoyl glycol chitosan (GCP41) and cholesterol by remote loading in response to an ammonium sulphate gradient (Haran et al (1993) *Biochem Biophys Acta* **1151** 201). Briefly, GCP41 ammonium sulphate vesicles were incubated with BLM and BLM was found to traverse the membrane and accumulate within these vesicles. These GCP41 based BLM vesicles were 600nm in size and had an encapsulation efficiency of 0.5U BLM:1mg GCP41. BLM polymeric vesicles were stable at room temperature for 28 days. The GCP41 based BLM vesicles also demonstrated good plasma stability, with 70% BLM remaining encapsulated over 24 hours.

This is the first report of a large molecular weight drug being loaded into polymeric vesicles by a remote loading method. GCP41 based BLM vesicles are to undergo *in vivo* testing.

### P50 ROLE OF PIM-1 IN THE REGULATION OF APOPTOSIS IN NON-HODGKINS LYMPHOMAS

IB Al-Abdulkarym*[1], MH Goyns[2] & SK Dower[1], [1]Div. of Molecular & Genetic Medicine, University of Sheffield, Sheffield, S10 2JF, [2]School of Health Science, Fleming Building, Sunderland, SR1 3SD.

The human pim-1 proto-oncogene was first cloned from the B-cell leukaemia 380 cell line and from a human genomic library (Nagarajan et. al., 1986, Proc. Natl. Acad. Sci. USA, Vol 83, p. 2556). The mRNA of the pim-1 proto-oncogene is 2.9Kb length. A full length pim-1 cDNA was cloned from the human chronic mylogenous leukaemia cell line K562. The pim-1 cDNA Open Reading Frame is 939 nucleotides and encodes for a 313 amino acid protein (Domen et. al., 1987, Oncogene Research, Vol 1, p. 103).

We investigated human pim-1 proto-oncogene expression in four Non-Hodgkin's lymphoma cell lines; Daudi, MC116, Namalwa and Raji. Our data indicate that pim-1 is overexpressed in all of these cell lines. We also cloned the full length pim-1 cDNA from the Raji cell line.

We have investigated the role of pim-1 in regulating entry into apoptosis in these cell lines by transfecting them with pim-1 sense and antisense constructs. Our data suggest that the down regulation of pim-1 expression in these cell line results in a high rate of apoptosis whereas pim-1 overexertion leads a small but detectable increase in apoptosis compared to control cells.

### P51 Apoptosis in lymph nodes in vivo: Fas Ligand expression by tumour cells facilitates selective immune evasion in a hostile environment:

R.O'Shea*, G. O'Sullivan, M. O'Donoghue, F. Shanahan and JK. Collins.
Depts. Of Microbiology and Medicine, University College, Cork, Ireland.

**Introduction:** To successfully metastasise tumour cells must have an immune escape capability. The fact that tumour cells invade, survive and grow in an immunologically hostile environment of lymph nodes demands a substantial explanation. While the exact mechanism(s) of immune evasion/ immunosupression is still uncertain we have shown that tumour and tumour derived cell lines secrete immunosupressive factors and Fas ligand that can induce apoptosis in activated lymphocytes in vitro. In this study, we provide evidence for significant apoptosis of lymphocyte populations in tumour draining lymph and investigated the role of tumour cell derived Fas ligand as the mediator of this apoptosis. **Methods & Materials:** At surgery, peripheral blood, tumour draining lymph nodes were obtained from 62 patients with Colorectal carcinoma. Apoptosis was detected and quantified by Flow Cytometry and DNA agarose gel electrophoresis. Using Annexin V, which preferentially binds to phosphatidylserine, an early marker of apoptosis, we examined and quantified the apoptotic sub populations.Preserved fixed sections of these apoptotic lymph nodes were immunohistochemically stained for Cytokeratin, Tunel( another marker for apoptosis), Fas receptor and Fas ligand. **Results:** Apoptosis, as measured a number of techniques was detected in 58% of patients with Colorectal carcinoma . Apoptosis ranged from 0% (0 of 7) in Dukes A, 58% (19 of 33) in Dukes B, 47% (8 of 17) in Dukes C, 82% (9 of 11) in Dukes D. Apoptosis correlated with a reduction in the CD3+ T- cell population in tumour draining lymph nodes, specifically there was a more significant and selective reduction in CD8+ T-cells in apoptotic lymph nodes. Dual labelling on the Flow Cytometer with Annexin-V and CD3, CD4 and CD8, respectively confirmed apoptosis in the T-lymphocyte population. Microscopic examination of the paraffin embedded lymph nodes showed apoptosis in lymphocytes using Tunel and also showed that tumour deposits were positive for Fas Ligand. **Conclusions:** Our results demonstrate a concomitant expression of high levels of Fas ligand by metastatic tumour cells and upregulated Fas ligand receptor by reactive CD8+ T-cells supporting the Fas counter attack hypothesis in vivo. We propose that Fas ligand expression by metastatic tumour cells is a major immune evasion/privilege mechanism facilitating invasion of the immune system itself, namely the lymph nodes.

### P52 ANTIGEN PRESENTATION AND INDUCTION OF LYMPHOCYTE APOPTOSIS BY A TRANSITIONAL CELL CARCINOMA LINE

S.J.Pettit*, E.O'Flaherty, S.Ali, T.R.L.Griffiths, D.E.Neal, J.A.Kirby, Surgical Immunobiology Group, Department of Surgery, University of Newcastle, Newcastle upon Tyne, NE2 4HH, UK.

In patients with carcinoma *in situ* of the bladder, 30% do not or only partially respond to BCG therapy. The immune response induced by BCG is believed to be mediated by T cells and might be improved by concurrent augmentation of tumour immunogenicity. Transfection of B7 costimulatory molecules confers an antigen presenting capacity to certain cancer cells. We investigated the ability of bladder cancer cells engineered to express B7 molecules to directly activate antigen-specific T cells.

The J82 transitional cell carcinoma line was separately transfected with B7-1 (CD80) and B7-2 (CD86). Clones were selected for high level expression of these ligands. Wild type J82, B7-1$^{+}$, and B7-2$^{+}$ transfectants were induced to express class II MHC antigens by treatment with IFN-γ; none of these cells could induce proliferation of immunomagnetically purified allogeneic CD4$^{+}$ T cells *in vitro*. However, the transfected B7 molecules were demonstrated to provide functional signals in an assay of costimulation.

Soluble factors capable of exerting an immunosuppressive effect were not found to be secreted by the J82 cell line. Expression of Fas (CD95) ligand by J82 cells was demonstrated at RNA level by reverse-transcription polymerase chain reaction and at protein level by western blot and flow cytometric analysis. Application of both [$^{3}$H]thymidine-DNA-fragmentation and TUNEL assays of lymphocyte apoptosis indicated that J82 cells induce the specific death of activated T cells.

We demonstrate that bladder cancer cells can be engineered to provide costimulatory signals, though this is not sufficient to allow direct activation of antigen-specific T cells by the J82 cell line *in vitro*. Moreover, induction of apoptosis may occur in activated T cells which encounter bladder cancer cells expressing Fas-L. Enhancement of tumour immunogenicity is of limited use if resistance to BCG therapy is associated with acquired mechanisms of immune escape. The provision of costimulatory signals should be considered within a broader range of T cell regulatory processes.

### P53 ELEVATED pRb IS ASSOCIATED WITH ELEVATED E2F-1 IN THE COLORECTAL CARCINOMA CELL CYCLE.

H. Lemass*, E. Ryan, P. Mac Mathuna, J. Crowe, J.C. O' Keane, Depts of Gastroenterology and Pathology, Mater Misericordiae Hospital, Dublin, Ireland.

The product of the Retinoblastoma (Rb) gene has a critical role not only in tumor suppression but also in the regulation of cell growth and differentiation. In contrast to the loss of pRb reported in several non G.I. malignancies, pRb is overexpressed in colorectal cancer (CRC). In order to address what might induce a recessive cancer gene to become overexpressed in CRC we analysed a transcription factor E2F-1 that binds to unphosphorylated pRb in the G1 phase of cell cycle control. Western Blotting was carried out on a group of 32 CRC surgical specimens (each with its normal control), and 2 CRC cell lines, (SW480, SW620). Monoclonal antibodies (Pharmingen) were used for all 3 proteins: pRb, unphosphorylated pRb, E2F-1. We have found 21/32 (65%) tumors have increased pRb expression; 19/32 (59%) tumors have increased unphosphorylated pRb expression; and 31/32 (97%) tumors have increased E2F-1 expression. Within the cell lines the results mirrored the events in CRC. Using the E2F-1 antibody an additional band was observed at ~110kd, which may represent a pRb-E2F-1 associated complex and this was also found to be increased in tumors compared to the normals by 22/32 (69%).
In conclusion, failure of increased pRb in CRC to inhibit proliferation may be related to an overall increase in the E2F-1 transcription factor.

### P54 DYSEQUILIBRIUM BETWEEN PROLIFERATION AND APOPTOSIS IN COLORECTAL CANCER,

DSWatson, IBrotherick, BKShenton, RGWilson, FCCampbell, Dept. of Surgery, The Medical School, Newcastle upon Tyne NE2 4HH.

Abnormalities of both apoptosis and proliferation have previously been demonstrated in colorectal cancer, but the relationship between the two has received little experimental attention.

We aimed to establish the relationship between proliferation and apoptosis in normal mucosa, to demonstrate the derangement in cancer and to see if the departure from the normal model correlated with pathological grade.

Samples of carcinoma and mucosa were isolated and pathological grade assessed following 41 colectomies. Each was mechanically disaggregated and suspended in saline. Half was mixed with propidium iodide and analysed by flow cytometry to measure cell cycle phase fractions. Half was fixed, then incubated with fluorescent tagged deoxyuridine triphosphate nucleotides and terminal deoxynucleotidyl transferase to label the DNA breaks characteristic of apoptosis. Flow cytometry was used to analyse the cell suspension and an apoptotic proportion calculated.

Proliferation (measured by the S-phase fraction) varied widely in both the tumours (mean 15.8%±SD13.0) and the mucosal specimens(mean 10.5%±SD6.6), as did apoptosis (tumours 18.1%±16.9 v. 28.1±18.0 for mucosa) with increased proliferation (P=0.019) and reduced apoptosis (P=0.017) in the tumours.

A linear relationship was demonstrated between apoptosis and S-phase fraction in the mucosal specimens (P=0.018). This relationship was lost in the specimens of carcinoma. Worse stages and grades of tumour diverged from the normal model no more than better ones.

This study demonstrates that the control of apoptosis and proliferation are closely related in normal mucosa and that this process is deranged in cancer, but the derangement is no more severe in worse stage or grade tumours.

### P55 EVIDENCE FOR DIFFERENTIATION-DEPENDENT AND INDEPENDENT PATHWAYS TO APOPTOSIS IN RESPONSE TO BUTYRATE AND TGFβ$_1$ IN HUMAN COLORECTAL ADENOMA CELLS.

A. Hague*[1], A. J. Butt[2] and C. Paraskeva[1]. [1]Department of Pathology and Microbiology, School of Medical Sciences, University of Bristol, University Walk, Bristol, BS8 1TD. [2]Institute of Medical and Veterinary Science, Frome Road, Adelaide, South Australia 500.

Butyrate, a naturally occurring factor derived from the diet, and transforming growth factor β (TGFβ$_1$) are growth inhibitory to colonic adenoma cell lines, inducing a $G_1$ arrest. Butyrate induces apoptosis in adenoma cell lines which is preceded by the induction of two markers of colonic differentiation - alkaline phosphatase (ALP) activity and E-cadherin protein expression. TGFβ$_1$ can be growth inhibitory without apoptosis in adenoma cell lines, however, the adenoma cell line PC/BH/C1 undergoes apoptosis in response to TGFβ$_1$. In contrast to butyrate, TGFβ$_1$-induced apoptosis was not accompanied by induction of differentiation markers. It is possible that the apoptosis induced by TGFβ$_1$ in the adenoma cell line PC/BH/C1 is due to conflicting signals, as downregulation of c-myc protein in response to TGFβ$_1$ occurs only slowly in this cell line. Development of resistance to TGFβ$_1$ in colonic tumours may involve two separate stages - resistance to growth inhibition and resistance to TGFβ$_1$-induced apoptosis. In response to butyrate, apoptosis is mediated via decreased levels of Bcl-2 or increased levels of Bak, depending on the genetic background of the cells, whereas levels of Bax remain unchanged. In association with TGFβ-induced apoptosis, Bcl-2 levels are reduced, but levels of Bak and Bax remain constant. As for butyrate, the induction of E-cadherin expression is a potentially important chemopreventive action, since increased E-cadherin expression has been correlated with decreased metastatic potential. Butyrate may reduce tumour growth and invasion, not only as a result of the induction of apoptosis, but also through increased expression of E-cadherin.

### P56 CELL CYCLE PERTURBATIONS AND APOPTOSIS IN EBV-TRANSFORMED LYMPHOBLASTOID CELL LINES OF DIFFERING P53 STATUS FOLLOWING GAMMA RADIATION.

R Gilchrist*, ME Lomax, RS Camplejohn. Richard Dimbleby Dept. Cancer Res., UMDS, St Thomas' Hospital, London, SE1 7EH

Reports on the reponse of EBV-transformed lymphoblastoid cell lines with differing p53 status to DNA damage in terms of cell cycle arrest and apoptosis have led to confusion. This may be partly due to the nature of the specific mutation under examination, but can largely be attributed to differences and problems with methodology and data analysis (for example the inappropriate use of static DNA histograms for cell cycle analysis). Taking seven lymphoblastoid cell lines, we have completed a detailed study of the cell cycle and apoptotic responses of these cells to gamma radiation. The lines were derived from both normal individuals and Li-Fraumeni/Li-Fraumeni-Like individuals carrying p53 mutations. Using BrdUrd pulse labelling, the cells were followed over the course of six hours and analysed by flow cytometry. Results showed that, regardless of p53 status, the cells did not arrest at the $G_1$ checkpoint even though in all of the cell lines p53 was shown to up-regulate within three hours of irradiation. However, the irradiated cells did show a general slowing both in entry into the S-phase from $G_1$ and in movement through S-phase. All cells demonstrated the expected $G_2$ arrest following irradiation. To assess apoptotic response to radiation in these cell lines a variety of techniques were evaluated. A variant of the TUNEL assay using flow cytometry and morphology were selected as being the most reliable. An increase in radiation-induced apoptosis was seen in all cell lines. There was a trend for cell lines with a mutant p53 allele to have both higher constitutive levels of apoptosis and a slightly increased apoptotic response compared to the control cell lines.

### P57 ROLE OF P53 IN MOUSE SKIN AFTER EXPOSURE TO IONISING RADIATION

D. Stuart[1], S.Williamson[1], K. Brown[1], and A. Balmain[2]. [1]Medical Oncology, CRC Beatson Laboratories, Garscube Estate, Glasgow, G61 1BD. [2]Onyx Pharmaceuticals 3031 Research Dr, BLDG.A, Richmond, CA 94806.

The aim of this study was to investigate the cellular events mediated by p53 following exposure of cells *in vivo* to ionising radiation. In adult mouse skin, distinct populations of keratinocytes respond to p53 stabilisation by entering an apoptotic pathway or executing a cell cycle block.

Apoptosis, as determined both by *in situ* tunnel assay and by morphology is restricted to the matrix region of the hair follicle and can be detected 4hrs after exposure to 4Gy ionising radiation. BrdU labelling studies demonstrate that matrix cells are capable of entering the apoptotic pathway from G2/M, as well as G1.

Interfollicular and non matrix follicular cells respond to radiation by initiating a cell cycle block, as measured by BrDu incorporation, which is detectable 24hrs after exposure to 4Gy. Pre treatment of skin with the tumour promoter TPA, which induces an increase in cellular proliferation rate, results in a cell cycle block within 4hrs of radiation exposure in the same cell population. Similarly, highly proliferative two week old mouse skin shows a cell cycle block 4hrs after exposure to radiation. Both cell cycle arrest and apoptosis following radiation are p53 dependent, as they are not detected in p53 knockout mice after radiation exposure.

These results suggest that cell cycle arrest and apoptosis following exposure of mouse epidermis to ionising radiation is cell type specific. The timing of cell blockage after damage is dependant on the proliferative state of the cell. However, ability to enter the apoptotic pathway is independent of cellular proliferation rate and seems likely to be dependent on the differentiation status of the cell.

### P58 REGULATION OF GADD45, C-FOS AND C-MYC PROTEINS AFTER DIFFERENTIAL NUCLEAR IRRADIATION

O. Al-Assar*[1], T. Robson[1], I. Gardin[2], S. R. McKeown[1], D. G. Hirst[1], [1]Radiation Science Group, School of Biomedical Sciences, University of Ulster, Jordanstown, Newtownabbey, Co. Antrim BT37 0QB, [2]Beaujon Hospital, Clichy, France.

Ionizing radiation is a damage inducing agent in mammalian cells. Cells adapt to a wide variety of insults by alteration in gene and protein expression[1]. Constitutive and inducible genes are known to be regulated by ionizing radiation but the localization of the trigger for the signalling pathways leading to gene induction and regulation after ionizing radiation is not yet known[2]. Membrane damage has been recently implicated in apoptosis after ionzing radiation[3]. We are using a model to test the role of both nuclear and membrane damage in triggering altered gene expression in mammalian cells. A microdosimetry model has been employed for the differential irradiation of the human lung epithelial cell line L132[4]. The model allows absorbed dose calculations to be made for differential nuclear irradiation after incorporation of $^3$H-thymidine.

Data from nuclear irradiation experiments were compared with the effects of 300kVp external beam X-rays. We used western blotting to monitor the regulation of GADD45 after exposure to 300kVp X-rays and GADD45, c-fos and c-myc after pulsed $^3$H-thymidine. Following doses of 2, 5 and 10 Gy 300kVp X-rays GADD45 expression increased progressively in a dose-dependent manner to a maximum of 3 fold after 48 hr. The response of GADD45 to nuclear irradiation was markedly different; induction of GADD45 was of similar magnitude (2.5 fold) to that seen with 300 kVp X-rays, but at total doses that resulted in much less cell killing. Furthermore, induction was transient after pulsed $^3$H-thymidine labelling. The expression of c-fos was also transient and c-myc protein showed a slight, transient increase after four hours. Our results are consistent with the hypothesis that a radiation sensitive molecular target for the initiation of signal transduction for these genes exists in the nucleus of this cell line. The differences between total cell and nuclear irradiation will require more detailed studies before further conclusions can be drawn.

**References**

1. Fornace, A. J., Jr. Annual Reviews in Genetics 26, 507-26 (1992).
2. Kyse, S. M. Cancer Biology 4, 119-28 (1993).
3. Verheij, M., Bose, R., Lin, X. H., et al. Nature 380, 75-79 (1996).
4. Gardin, I., Faraggi, M., Huc, E. et al. Physics in Medicine and Biology 40, 1001-1014 (1995).

### P59 DELAYED GENOMIC INSTABILITY AND APOPTOSIS IN NORMAL HUMAN FIBROBLASTS FOLLOWING X-RAY AND α-PARTICLE IRRADIATION

O.V.Belyakov*[1,2], K.M. Prise[2], K.-R. Trott[3], B.D. Michael[2], [1]Dublin Institute of Technology, Ireland, [2]Gray Laboratory CRT, Northwood, UK, [3]St.Bartholomew's and the Royal London Hospital School of Medicine and Dentistry, London, UK

Radiation induced genomic instability may be an important contributor to carcinogenesis. The aim of this work was to investigate the initial and delayed frequency of micronucleated cells and apoptosis after irradiation. X-ray and α-particle irradiation were carried out in AG01522B primary human fibroblasts.

Cells were allowed to grow under normal proliferative conditions, without the use of cytochalasin B. Micronuclei were scored using daughter cell analysis on acridine orange stained cells. Apoptosis was assessed by morphological criteria on acridine orange stained samples, by DNA fragmentation with *in situ* 3'-OH DNA end-labelling and by detection of phosphatidylserine expression on the outer surface of the cell membrane with Annexin V.

We have shown in our experiments that there are significant differences between X-ray and α-particle irradiated fibroblasts in both the yields and time-course of micronuclei production. An initial peak in micronuclei and apoptosis yield is found 3 days after both, X-ray and α-particle irradiation. In the case of 3 Gy of α-particles a delayed peak on day 8 was observed. Both types of irradiation also induced delayed genomic instability at later times (8-30 days). α-particle irradiation is more efficient in producing genomic instability and, in the case of higher doses, the peak in micronuclei formation and apoptosis is at later times. The existence of genomic instability was also confirmed by cell survival assays on day 0, 8, 14 and 24 after irradiation. In comparison with short term samples, damaged cells scored at later times are more frequently found to be heavily micronucleated. Also, at later times, apoptosis became a greater contributor to total cellular damage than micronuclei production.

We find that micronuclei and apoptosis frequency curves, at early and delayed times have a similar time dependency. Morphologically, it is difficult to distinguish between apoptosis and heavily micronucleated cells. An *in situ* 3'-OH DNA end-labelling technique was employed to confirm detection of apoptotic cells. It confirmed the time dependence of apoptosis induction but values were higher than with conventional morphological analysis. A possible reason is that *in situ* 3'-OH DNA end-labelling is able to detect three different morphological stages of apoptosis, whereas conventional staining detects only the last stage, fragmentation of the nucleus.

Annexin V detection of phosphatidylserine externalisation, was used as a marker for the early stages of apoptosis after α-particle and X-rays irradiation. We find that α-particles are more efficient in apoptosis production then X-rays. Also there are differences in the time course. The peak of apoptosis production is 1 day after α-particle irradiation with a decrease up to day 6. The fraction of apoptotic cells remained dose dependent for all time points. For X-rays the fraction of apoptotic cells starts to increase on day 2 and slowly decreases afterwards, independently to dose. These experiments confirm that phosphatidylserine expression is an early event in these irradiated fibroblasts. In control flow cytometry experiments we found that only a small fraction of fibroblasts detach from the substrate and undergo apoptosis.

We demonstrate in this work an evidence of radiation-induced genomic instability in normal human fibroblasts which have being cultivated under normal proliferative conditions. The results suggest a considerable contribution of programmed cell death to total cellular damage, especially at delayed times after irradiation.

### P60 A DOSIMETRY MODEL FOR THE ACCURATE CHARACTERISATION OF ABSORBED DOSES IN MOUSE ORGANS.

*A. A. Flynn, A.J. Green, G.M. Boxer, R.B. Pedley, R.H.J. Begent. *Department of Clinical Oncology, Royal Free Hospital School of Medicine, London NW3 2PF.*

Radioimmunotherapy (RIT) uses systemically administered antibodies to direct a therapeutic dose of radiation to cancer deposits. Animal dosimetry models are useful for understanding and evaluating the toxicity caused by RIT in tumour and normal tissues and for assessing the potential clinical use of antibodies for tumour targeting.

Biodistribution experiments give useful information on the localisation and pharmacokinetics of radiolabelled antibodies and enable the calculation of the dose delivered to each organ. Conventionally, the dose to the organ is a function of the integration of radioisotope uptake over time. Two simplifying assumptions often made in the these calculations is that non penetrating beta radiation originating in the source organ is wholly absorbed in the organ and that the radiopharmaceutical is uniformly distributed in the source organ. However, we have previously shown, using radioluminography, that the distributions in bone, kidney and tumour are not uniform. For dosimetry purposes kidney cortex and medulla should be considered separately as source organs as should bone and marrow. In tumour, antitumour antibodies predominantly localise in the well vascularised areas with limited access to areas of hypoxia and therefore presents a more complex problem for absorbed dose calculations. Additionally, mouse organs are relatively small compared with the range of medium to high energy beta particles. Consequently, a significant amount of beta radiation energy that originates in one organ can escape and be absorbed in adjacent organs.

We have calculated the absorbed fraction of the beta energy emitted from $^{90}$Y and $^{131}$I in mouse organs using beta dose point kernels. Organs included were the liver, spleen, kidney cortex, kidney medulla, lungs, heart, stomach, small intestine, large intestine, thyroid, cortical bone and marrow. By measuring the overlapping surface areas between adjacent organs, the absorbed fraction in adjacent organs was calculated. To account for radioantibody heterogeneity in tumour, a 3D model was developed by reconstructing the radioluminographs of serial tumour sections. The dose rate to all parts of the tumour was calculated using point kernels.

Dose calculations show that the kidney cortex receives a significantly larger dose than the medulla as does the bone marrow when compared to cortical bone. The tumour model demonstrates that the dose delivered to the viable radiosensitive part of the tumour is approximately twice the mean tumour dose and four times the dose delivered to hypoxic areas when using $^{90}$Y. When using $^{131}$I, the viable cells receive two and half times the mean tumour dose and up to seven times the dose delivered to hypoxic cells.

We have demonstrated the heterogeneity in dose delivered to the kidney cortex and medulla and the well and poorly vascularised parts of the tumour using antitumour antibodies, it is likely that similar differences occur with conventional cytotoxic drugs. This shows the need for the development of similar models for systemically delivered drugs in order to understand the toxicity in the organs of animals. With further development of the models, we can improve our prediction of the effect of therapy in man.

### P61 MATRIX METALLOPROTEINASE PRODUCTION AND *IN VITRO* INVASION IN A COLON CANCER MODEL,

S. Cowell* and P. de Takats, Experimental Metastasis Group, Strangeways Research Laboratory, Cambridge CB1 4RN.

The process of metastasis requires cells to pass through a basement membrane. It is widely believed that this ability to cross extracellular matrix barriers requires the production of active enzymes in order to degrade the various extracellular matrix components. The matrix metalloproteinase (MMP) family of enzymes can degrade many of these components, and many human cancers have been shown to express increased amounts of MMPs.

In our study, an *in vitro* model of human colon cancer progression (Williams, A.C. *et al.*, Cancer Res. **50**: 4724-4730, 1990) was chosen to examine differences in MMP expression between adenoma and carcinoma cells. The adenoma cell line (AA/C1) was derived from a large human colonic tubular adenoma, and retains certain characteristics of colon cells such as the production of mucus glycoproteins. The carcinoma cell line (AA/C1/SB/10C) was produced by sodium butyrate and *N*-methyl-*N'*-nitro-*N*-nitrosoguanidine treatment of AA/C1 cells. Only the carcinoma cells increase their migration into a reconstituted basement membrane upon treatment with epidermal growth factor (EGF) (Brunton, V.G. *et al.*, Oncogene **14**: 283-293, 1997).

We investigated the secretion of latent and active stromelysin, collagenase 3 and gelatinases by these cells using Western blotting, gelatin zymography and immunocytochemistry. The cells were also examined for urokinase-type plasminogen activator (u-PA) and membrane-type I matrix metalloproteinase expression, as these enzymes are responsible for the activation of many of the MMPs. As a measure of invasiveness, we examined the relative abilities of the two cell lines to pass through transwell filters coated with extracellular matrix proteins (laminin, type IV collagen, type I collagen or fibronectin). Cells were seeded onto the coated filters and cultured for several days before examining the filters under a confocal microscope.

The results obtained showed that there were some differences in MMP expression between the two cell lines. Both cell lines express gelatinase A and TIMP-1. Treatment with phorbol ester upregulated u-PA and u-PA receptor in both lines, and gelatinase B and collagenase 3 in AA/C1/SB/10C cells. However, in our *in vitro* system their invasive capabilities were similar even in the presence of EGF or phorbol ester, which may reflect the limitations of this system as a model for metastasis by colon carcinoma cells.

### P62 PROTEASE EXPRESSION IN AN *IN VITRO* MODEL OF BLADDER TUMOUR INVASION.

C Booth*, P Harnden, LK.Trejdosiewicz, PJ Selby & J Southgate. ICRF Cancer Medicine Research Unit, St James's University Hospital, Leeds LS9 7TF.

We have established an human bladder organ culture system for the study of interactions of normal human urothelial cells (Scriven *et al*; *J Urol 1997;* **158**: 1147) and bladder carcinoma cells (Booth *et al. Lab Invest 1997;* **76**: 843) with de-epithelialised urothelial stroma. Transitional cell carcinoma (TCC) cell lines showed three different invasion patterns: well-differentiated RT4 cells were non-invasive, moderately-differentiated RT112 cells invaded the sub-epithelial capillary bed and anaplastic EJ cells additionally invaded the stromal matrix.

The aim of this study was to determine if differences in invasive properties of TCC cell lines were related to the expression and activity of urokinase plasminogen activator (u-PA), its activating receptor (u-PAR), gelatinases A and B (MMP2 and MMP9) and their inhibitors (TIMP1 and TIMP2). MMPs secreted by adherent cultured cells were detected by gelatine zymography and confirmed by immunoblotting. Immunohistochemical localisation of proteases was carried out on cells grown in culture and on sections of tissues and organ cultures.

Normal urothelial cells and TCC cell lines produced u-PA and large quantities of MMP2 and MMP9, of which the majority was in the inactive or pro-form. Only EJ and RT112 cells expressed detectable u-PAR, which was associated with the cell-surface. EJ cells did not express TIMP2.

These data show that normal and RT4 urothelial cells have the capacity to produce high levels of proteases in monoculture, but do not invade when seeded onto a stroma, suggesting that in non-invasive cells, proteolytic activity is under strict regulation. However, the vascular-invasive RT112 and EJ cell lines both displayed cell surface-localised u-PAR, suggesting that u-PAR may be an important factor in the process of intravasation. The lack of expression of TIMP2 by EJ cells may be a factor in their ability to invade the stromal matrix.

*Acknowledgements.* This work was supported by the Association for International Cancer Research (AICR)

### P63 DOWN REGULATION OF TISSUE INHIBITORS OF METALLOPROTEINASES (TIMPs) IN HEPATIC TISSUE SURROUNDING COLORECTAL CARCINOMA METASTASES.

S.R.Kelly*, A.C.Gough, J.N.Primrose, University Department of Surgery, Southampton General Hospital, Tremona Road, Southampton. SO16 6YD.

Matrix metalloproteinases (MMPs) and their inhibitors (TIMPs) are a group of enzymes involved in extracellular matrix breakdown and remodelling. In normal tissue turnover there is a balance in activity between MMPs and TIMPs, but this balance is thought to be disrupted in favour of breakdown to facilitate invasion and metastatic spread in colorectal carcinoma (CRC). We examined the mRNA expression pattern of TIMP 1 and 2 in hepatic tissue and CRC metastases.

Fresh tissue obtained from ten patients undergoing hepatic resection for metastases from CRC was immediately snap frozen in liquid nitrogen. Total cellular RNA was extracted from the metastasis, the immediately adjacent liver (within 1cm of the metastasis) and distal liver (>5cm distant), and subjected to reverse transcription (RT) using an oligo dT primer. RT-PCR was performed using specific oligonucleotide primers to detect TIMP 1, TIMP 2 and β-actin (a ubiquitously expressed gene). The PCR products were analysed by non-denaturing polyacrylamide gel electrophoresis and visualised by ethidium bromide staining under ultraviolet transillumination. The specificity of the PCR products was confirmed by sequencing.

In all ten patients the liver metastases and distal liver expressed both TIMP 1 and 2 mRNA at equivalent levels. However in seven of the ten patients TIMP 1 expression was lower in the liver tissue immediately adjacent to the metsatasis, and in nine of the ten patients TIMP 2 expression was lower.

These results suggest that there is down regulation of both TIMP 1 and 2 in liver tissue adjacent to hepatic metastases, and that this facilitates local growth of hepatic metastatic disease.

### P64 MMP 2 AND 9 IN HUMAN BREAST CARCINOMAS.

K. McCarthy*, T. Maguire, A.M. McElligott, H. McGlynn, G. McGreal, E. McDermott, N. O'Higgins, M.J. Duffy. St. Vincent's Hospital, Dublin 4, Ireland and University of Ulster, Coleraine, Northern Ireland.

Data from model systems suggest that certain matrix metalloproteinases (MMPs) are causally involved in cancer invasion and metastasis.

The aim of this study was to establish the relationship between two MMPs, i.e., MMP-2 (gelatinase A, Mr 72,000) and MMP-9 (gelatinase B, Mr 92,000) and metastatic potential in human breast carcinomas.

Active and latent forms of MMP-2 and MMP-9 were measured by zymography on 7.5% polyacrylamide gels containing 4% gelatin. Media from activated HT1080 fibrosarcoma cell lines were used as a control.

Total (precursor and active) levels of MMP-2 and MMP-9 were detected in 70/117 (60%) and 81/118 (69%) of primary breast carcinomas, respectively. Using the Mann-Whitney U test, higher levels of MMP-2 and MMP-9 activities were found in primary carcinomas compared to fibroadenomas ($n = 15$), ($p < 0.0001$ for both MMPs). Both gelatinases correlated significantly with each other ($r = 0.36$, $p = 0.0001$, $n = 117$). However, neither activities were significantly associated with established prognostic markers such as tumour size, nodal status or estrogen receptor status. Using univariate analysis, high levels of MMP-2, but not MMP-9 activity correlated with shortened disease free interval (chi square = 6.53, $p = 0.0106$) and overall survival (chi square = 4.57, $p = 0.0355$).

We conclude that both MMP-2 and MMP-9 activities are significantly higher in primary breast carcinomas than in fibroadenomas. Furthermore, the correlation between MMP-2 and patient outcome is consistent with data from model systems implicating this MMP in cancer invasion and metastasis.

### P65 MATRIX METALLOPROTEINASES IN DIFFERENT TYPES OF SKIN CANCER

C. O' Shea*[1], T. Maguire[1], D. Chin[2], D.S. Soutar[2], M J. Duffy[1], [1]Dept. of Nuclear Medicine, St. Vincent's Hospital, Dublin 4, [2]Dept. of Plastic Surgery, Canniesburn Hospital, Glasgow.

The principal types of skin cancer, i.e., basal cell carcinomas (BCC), squamous cell carcinoma (SCC), and malignant melanoma vary widely in their metastatic potential. Thus, while SCCs, and especially BCCs rarely metastasise, malignant melanoma frequently give rise to metastases. The aim ot this work was to investigate the possibility that the wide difference in the metastatic propensity of these different types of skin cancer was due to different levels of matrix metalloproteinases (MMPs), a group of endopeptidases causally involved in cancer spread.

MMP-1 and 3 levels were measured by ELISA (prototype kits from R & D Systems) while MMP-2 and 9 activities were determined using gelatin zymography.

Levels of MMP-1, 2, 3 and 9 in BCCs and SCCs were not significantly different. However, for MMP-1, 3 and 9 (both precursor and active forms), levels were significantly higher in both BCCs and SCCs compared with the melanomas. For MMP-2, precursor levels were higher in SCCs compared with melanomas, while for active MMP-2, activities were higher in BCCs than in melanomas. Within the combined group of skin tumours, a number of correlations between the different MMPs were found. Thus, MMP-1 levels correlated significantly with both the active ($r = 0.58$, $p = 0.0001$, $n = 44$) and precursor forms of MMP-2 ($r = 0.37$, $p < 0.05$, $n = 44$) as well as MMP-3 levels ($r = 0.58$, $p = 0.0001$, $n = 45$). Similarly, active MMP-2 levels were significantly associated with MMP-2 precursor levels ($r = 0.64$, $p = 0.0001$, $n = 72$), MMP-3 levels ($r = 0.63$, $p = 0.0001$, $n = 49$), active MMP-9 levels ($r = 0.49$, $p = 0.0001$, $n = 72$) and MMP-9 precursor levels ($r = 0.53$, $p = 0.0001$, $n = 72$).

We conclude that in contrast to our findings with uPA, MMP-1, 2, 3 and 9 levels were generally higher in both BCCs and SCCs relative to malignant melanomas. The low metastatic potential of both BCCs and SCCs is therefore unlikely to be due to a deficiency of these MMPs.

### P66 HIGH LEVELS OF TIMP-1 PREDICT POOR OUTCOME IN PATIENTS WITH BREAST CANCER,

K. McCarthy*, T. Maguire, G. McGreal, E. McDermott, N. O'Higgins, M.J. Duffy, Dept. of Nuclear Medicine and Surgery, St. Vincent's Hospital; Dublin 4, Ireland.

Matrix metalloproteinases (MMPs) are Zn-dependent endopeptidases which catalyse degradation of diverse substrates in the extracellular matrix. *In vivo*, activity of these MMPs is controlled by a group of inhibitors known as TIMPs. In many model systems, metastatic potential is positively correlated with MMP levels and negatively associated with TIMP levels.

The aim of this study was to establish the relationship between levels of TIMP-1 and metastatic potential in human breast carcinomas. Levels of TIMP-1, MMP-1 and MMP-3 were determined by ELISA (Amersham, UK).

Levels of TIMP-1 protein ≥ 0.624 ng/mg protein were detected in 123/139 (89%) primary breast carcinomas. Higher levels of TIMP-1 were found in primary carcinomas compared to fibroadenomas ($z = -2.134$, $p = 0.0329$). TIMP-1 levels correlated significantly with both MMP-1 and MMP-3 levels ($r = 0.203$, $p = 0.0178$ and $r = 0.221$, $p = 0.012$, respectively). In primary carcinomas, TIMP-1 levels correlated inversely with ER concentration ($r = -0.246$, $p = 0.004$) and positively with tumour size ($r = 0.242$, $p = 0.0067$). Using univariate analysis, patients with low levels of TIMP-1 protein had a significantly better survival than patients with higher levels. Using RT-PCR, TIMP-1 mRNA was detected in 51/60 (85%) primary breast carcinomas.

Our results show that TIMP-1, rather than inhibiting breast cancer progression, may indeed potentiate this process. Similar results have been found for the urokinase plasminogen activator inhibitor, PAI-1.

### P67 ISOLATION AND CHARACTERISATION OF NOVEL HUMAN ANTIBODIES TO MATRIX METALLOPROTEINASE-9 FROM A PHAGE DISPLAY LIBRARY.

Maria Morgan*, Coner Lynch and Susan McDonnell, School of Biological Sciences, Dublin City University, Dublin 9, Ireland.

Degradation of the extracellular matrix by proteolytic enzymes, including the matrix metalloproteinases (MMPs) is a critical event in the process of cancer invasion and metastasis. MMP-9 which degrades type IV collagen, gelatin and fibronectin is expressed in a variety of cancer types such as colon, breast, bladder, ovarian, and prostate cancer, and its levels appear to be related to malignancy and invasion. *In vivo* these enzymes are secreted in a latent form and must be cleaved to yield an active degradative enzyme. Isolation of antibodies which specifically recognise the active form of this enzyme has proved elusive by conventional methods, however this is essential as it is measurement of the active enzyme that is most clinically relevant. The display of repertoires of antibody fragments on the surface of filamentous bacteriophage offers a new way of making antibodies with predefined specificities. Antibody fragments are displayed by fusion to the viral coat protein, allowing phage with antigen binding activities and encoding the antibody fragments to be selected by panning on antigen. The 'Nissim Library' developed by the Centre for Protein Engineering, Cambridge (Nissim *et al*, EMBO 13, 692-698, 1994) contains over $10^8$ antibody specificities constructed by the pairing of a single unmutated V light chain gene segment with a diverse repertoire of *in vitro* rearranged $V_H$ genes containing a random $V_H$-complementary determining regions3 that are expressed on the surface of filamentous phage.

Approximately $10^8$ single chain Fv(scFv) antibody producing *E. coli* TG1 clones underwent 4 rounds of affinity selection (panning) to MMP-9 according to Nissim *et al.* Samples of polyclonal phage populations taken after each round of panning were tested by ELISA and showed excellent enrichment of binding activity to MMP-9. To identify monoclonal phage antibodies the pHEN phage particles were rescued and 82 individual clones from round 4 were screened by phage ELISA against MMP-9. 42 of the 82 clones (52%) were found to be positive. These positive phage clones were then infected into *E. coli* HB2151 and induced for soluble expression of scFv antibody protein. The production of antibody into periplasm and supernatant was assessed. After transferring to HB2151 72% of clones produced soluble scFv fragments when tested by ELISA against MMP-9 protein. Culture supernatants were also tested by western blot using an anti-myc antibody which detects the myc tag on the scFv fragment. Bands can be seen at 31kD for the scFv protein. Antibodies from clones with the greatest binding activity by ELISA were titered and found to be in the range 1/100 dilution.

Further characterisation studies on these scFv fragments to MMP-9 are currently underway. It is hoped that the unlimited supply of these novel antibodies will be used to develop reagents, which may aid in the treatment and diagnosis of conditions involving ECM degradation.

### P68 MATRIX METALLOPROTEINASE-2 (MMP-2)AND ITS INHIBITOR TIMP-2 ARE INCREASED IN GASTRIC TUMOURS COMPARED TO NORMAL MUCOSA AS DEMONSTRATED BY COMPETITIVE RT-PCR.

**GM Tierney*, HM Collins, SA Watson.**
**Academic Unit of Cancer Studies, Division of GI Surgery, University of Nottingham, UK.**

**Background:** The MMPs are a family of proteolytic enzymes which regulate turnover of the extracellular matrix and are implicated in the process of tumour growth and metastasis. Their inhibition is a novel therapeutic modality. It remains unclear which member(s) of this family are of most functional significance.

**Aim:** To use RT-PCR to quantify the relative amounts of MMPs and their tissue inhibitors (TIMPs) within gastric tumours. To confirm the mRNA findings using gelatin zymography.

**Methods:** Gastric carcinoma tissue and normal mucosa were obtained from twenty resections. mRNA was extracted and reverse transcribed to cDNA. Competitive PCR was performed with a gene specific external standard. Results were normalised to GAPDH. Zymography was performed to show the active and inactive forms of MMPs-2 and -9.

**Results:** mRNAs for MMPs-2, 7 and -9 were expressed significantly more frequently in tumour than normal mucosa ($p=0.05$ $\chi^2$). Of the TIMPs, TIMP-2 but not TIMP-1 was significantly expressed in tumour compared to normal ($p=0.05$ $\chi^2$). There was a highly significant increase in the quantity of mRNA for MMPs-2, and -7 in gastric cancers ($p<0.01$ Mann-Whitney) and a less marked but significant increase in TIMP-2 mRNA ($p<0.05$ Mann-Whitney).There was a trend to significance in the increased expression of MT-MMP-1 in tumours. Gelatin zymography revealed both forms of MMPs-2 and -9 in the tumour samples but inactive species only in the normal mucosa.

**Discussion:** Using these two techniques a relative quantity of each enzyme within every sample was obtained at the mRNA level and the presence of functioning protein confirmed. When tumour tissue is compared to normal a significant difference is found for MMPs-2 and -7. These enzymes have a well documented role in the process of invasion and metastases. A significant increase in the mRNA for TIMP-2 (capable of binding to both the latent and active forms of MMP-2) has also been demonstrated. This work further stresses the importance of MMP-2 in the malignant process.

### P69 CO-CULTURE OF HUMAN FIBROBLAST AND COLORECTAL CANCER CELL LINES UPREGULATES THE EXPRESSION OF FIBROBLAST DERIVED MT-MMP-1.

H.M. Collins*, G.M. Tierney, S.A. Watson.
The Academic Unit of Cancer Studies, Department of Surgery, University Hospital, Nottingham.

Introduction: Matrix Metalloproteinases (MMPs) are proteolytic enzymes involved in the turnover of the extracellular matrix and are implicated in the process of tumour growth and metastases. The interaction between tumour and stroma is thought to be important in this process. Membrane Type - MMP-1 is a potent activator of pro- MMP-2, and was thought to be linked to tumour cell surfaces. Evidence now exists that the stromal cells within a tumour mass may also express MT-MMP-1.

Aims: To examine the expression of MT-MMP-1 in a human fibroblast cell line and to establish if co-cultivation with colorectal cancer cell lines has an effect on this expression. To determine if expression of MT-MMP-1 has a subsequent effect on MMP-2 activation.

Methods: The fibroblast cell line 46BR.IGI was co-cultivated in serum free medium with the colorectal cancer cell lines; C170HM2 and AP5LV in transwell plates separated by 3μm porous filters. All assays were conducted for 72 hours, at which point each cell type was harvested and mRNA extracted using guanidium thiocyanate. mRNA was purified and reverse transcribed to double stranded cDNA. PCR was performed with the following primer pairs; MMP's-2, -9, and MT-MMP-1. Conditioned media was subjected to SDS-PAGE gelatin zymography.

Results: Mono-cultures of the fibroblast cell line expressed mRNA for MMP-2 and -9 but did not express MT-MMP-1 mRNA, however co-culture with the colorectal cancer lines induced expression of MT-MMP-1 while retaining expression of MMP-2 and -9. Expression of MT-MMP-1 did not activate pro-MMP-2.

Conclusions: MT-MMP-1 expression is induced in fibroblasts by factors released by the colorectal cells, cell : cell contact may be necessary for MMP-2 activation.

### P70 CO-EXPRESSION OF MEMBERS OF THE MEMBRANE-TYPE METALLOPROTEINASE FAMILY, THE ADAM FAMILY AND THE ASTACIN FAMILY IN MCF7 AND T47D CELLS.

S.J. Dale, M.I. Millichip, J. Lawry* and N. McKie. Department of Human Metabolism and Clinical Biochemistry and Institute for Cancer Studies* University of Sheffield Medical School, Beech Hill Road, Sheffield S10 2RX

Membrane mediated metalloproteinase (MMP) activity is proposed to be an essential requirement for the metastatic development of the cancer cell, since it is this activity which is responsible for matrix remodeling which facilitates the egress of the metastatic cell through basement membrane components into the vasculature. In light of this it is proposed that members of the MMP family are instrumental in cellular dissemination, due to their type IV collagenase activity.

We report here the expression of several members of different metalloproteinase families in the breast carcinoma cell lines MCF-7 and T47D. Using RT-PCR we detect members of the membrane-type matrix metalloproteinases (MT-MMPs) MT-MMP1 and MT-MMP4, the astacin family meprin a and b and ADAMs family members 10, 12 and 15, all of which are proposed to have active metalloproteinase domains. Using immunohistochemistry, flow cytometry and Western blotting techniques we have demonstrated protein expression of meprin and ADAM 10 and 15. Astacin-like activity is exhibited in the MCF-7 cell line using protein biochemistry.

The cell membrane of these tumour cells therefore has a much larger repertoire of membrane-linked metalloproteinase genes than has previously been reported, which may contribute towards the development of the metastatic phenotype.

This work is funded by **Yorkshire Cancer Research.**

### P71 EXPRESSION OF MATRIX METALLOPROTEINASES AND TIMPs IN COLORECTAL CANCER CELL LINES USING RT-PCR.

H.M. Collins*, G.M. Tierney, S.A. Watson.
The Academic Unit of Cancer Studies, Department of Surgery, University Hospital, Nottingham.

Introduction: Matrix Metalloproteinases (MMPs) are a family of proteolytic enzymes involved in the turnover of the extracellular matrix and are implicated in the process of tumour growth and metastases. Their action is regulated by the naturally occurring inhibitors, TIMPs. Evidence exists that the "net proteolytic" activity is of overall importance in the process of metastasis. The interaction between tumour and stroma is thought to be important.

Aims: To establish a quantitative profile of MMPs and TIMPs for metastatic and non-metastatic colorectal cell lines grown *in vitro* in the absence of any stromal influence.

Methods: Colorectal cell lines C170 (non-metastatic), C170HM2 (hepatic invasive), AP5 (non-metastatic) and AP5LV (lung invasive) were harvested using EDTA. mRNA was extracted using guanidium thiocyanate, purified and reverse transcribed to double stranded cDNA. Competitive PCR was performed with the following primer pairs; MMP's-1,-2,-9,-11, MT-MMP-1,-2, TIMPs 1,2 and GAPDH. Reactions included a standard multi-competitor cDNA containing priming sites for each of the MMPs studied and $^{32}P$ αdCTP. The samples were separated on 6% TBE gels and stained with ethidium bromide, bands were excised and counts per minute determined using a Top Count. Results were expressed relative to GAPDH.

Results: mRNA was detected for all primers used with the standard multi-competitor cDNA. MMP-2 mRNA was not detected in any of the cell lines. mRNA for MMPs-1 and -9 was only detected in C170HM2. Soluble MMPs were not expressed in AP5 or the lung invasive cell line,AP5LV, under the culture conditions employed, however MT-MMP-1 and TIMP-2 were highly expressed in both.

Conclusions: The cell lines do not express mRNA for MMP-2, commonly localised to stromal cells. The net proteolytic activity of the metastatic cell line (C170HM2) is potentially greater than the non-metastatic (C170).

### P72 IDENTIFICATION OF GENES INVOLVED IN EPITHELIAL CELL:MATRIX INTERACTIONS BY DIFFERENTIAL DISPLAY.

B.A. Smith*, G.D. Hall, P.J. Selby, L.K. Trejdosiewicz & J. Southgate. ICRF Cancer Medicine Research Unit, St James's University Hospital, Leeds, LS9 7TF.

The stroma is believed to play a regulatory role in epithelial cell proliferation and differentiation during homeostasis and tissue regeneration. Dysregulation of these processes is implicated in the development and progression of neoplastic disease. We have used differential display RT-PCR to identify changes in gene expression induced when normal human urothelial (NHU) cells interact with basement membrane-derived Matrigel matrices.

Total RNA was extracted from NHU cells grown on Matrigel or Primaria tissue culture plastic for 24 or 48 hours. After removal of contaminating DNA, differential display RT-PCR was carried out using 30 primer combinations. The differential display products were analysed on sequencing gels. 27 bands showed a relative increase in intensity and were excised from gels, re-amplified and cloned into *pGEM3Z*. Up to 6 clones from each band were sequenced and homologies assessed by DNA database comparisons.

Upregulation of gene expression by cells grown on Matrigel was confirmed by Ribonuclease Protection Assays (RPA) for 9 independent sequences, including 4 ESTs and 5 known genes: connexin 26, macrophage migration inhibition factor (MMIF), cystatin B, elafin and antileukoprotease. These genes have recognised roles in cell:matrix and cell:cell interactions, although only connexin 26 expression has previously been studied in bladder cancer. By RPA, three of the mRNAs (connexin 26, MMIF and elafin) were absent from urothelium *in situ,* suggesting that expression of these genes forms part of the inducible response repertoire of urothelium.

This approach has identified novel and previously non-implicated gene products involved in normal urothelial:matrix interactions which may be significant in the development of invasive and metastatic tumour phenotypes. Furthermore, the identification of cystatin B (a cysteine protease inhibitor) and the serine proteinase inhibitors, elafin and antileukoprotease, raises the possibility that this approach may be useful in identifying novel protease inhibitors.

### P73 PHORBOL ESTER INDUCED ALTERATIONS IN THE METASTATIC PROFILE OF A RENAL CANCER CELL LINE

D.M.P. Morrow*, A.M. McElligott, S.M. Farragher and H. McGlynn, School of Biomedical Sciences, University of Ulster at Coleraine, Northern Ireland.

The ability of cells to organise and remodel surrounding extracellular matrix (ECM) is essential in metastasis, the leading cause of cancer mortality. The matrix metalloproteinases (MMPs) are a family of zinc dependent enzymes that degrade the major components of the ECM. The balance between MMPs and their natural inhibitors, tissue inhibitors of metalloproteinases (TIMPs) is critical in determining net MMP activity. Studies of the mechanisms of differential expression of these genes would lead to better understanding of the metastatic process. We have investigated the modulation of MMP and TIMP genes in a metastatic renal carcinoma cell line Caki-1, by the tumour promoter 12-*O*-teradecanoylphorbol-13-acetate (TPA, 20ng/ml). The effect of a 12 hour treatment on MMP/TIMP gene expression was determined by Reverse Transcription-Polymerase Chain Reaction (RT-PCR) analysis. The phorbol ester TPA was seen to enhance the invasive potential of the Caki-1 cell line in two ways. Firstly, TPA compromises the 1:1 stoichiometry of the MMP:TIMP ratio; as MMP-2 expression is significantly increased, while expression of TIMP-1 and TIMP-2 remained unchanged. Secondly, TPA also increased the expression of membrane-type MMP-1 (MT-MMP-1) which is known to play an important role in the process by which MMP-2 is activated. MMP-2 activation has been directly correlated with the aggressiveness of tumour cells.

### P74 Antibodies to αv inhibit the in vivo growth of αvβ3-positive, but not αvβ3-negative, melanoma cells.

H. Cadiou[1], T. Meyer[1], F. Mitjans[2], S.L. Goodman[3], I.R. Hart[1] and J.F. Marshall[1]. [1]Richard Dimbleby/Imperial Cancer Research Labs, St. Thomas' Hospital, London SE1 7EH, [2]Merck Farma Y Quimica, Spain, [3]Merck KGoA, Germany

Several studies have implicated the integrin αvβ3 as being associated with progression towards the invasive forms of melanoma, although the mechanisms underlying this association are unknown. To study possible mechanisms we have generated a recombinant amphotropic retrovirus encoding the human β3 subunit (pBabe-puro β3) and infected the β3 negative human melanoma line, VUP. Puromycin-resistant VUP cells obtained after 10 days in selection media, were enriched for β3-expression using magnetic bead sorting employing the anti-β3 antibody SZ-21. Cells were cloned and analysed by flow cytometry and immuno-precipitation to confirm the cell surface expression of β3. A representative αvβ3 positive clone (V15) and a representative αvβ3 negative clone (V20) were analysed further. Injection of $2x10^6$ V15, V20 or VUP (parent) cells s.c. or i.v. into athymic nude mice failed to form tumours over a six months observation period. In contrast, if comparable cell numbers were coinjected with 200μl matrigel all three lines formed rapidly growing tumours (11 days to 5mm diameter) Similar in vivo growth rates indicated that the matrigel-enhanced xenograft formation of these lines was independent of αvβ3 expression. However, when $2x10^6$ tumour cells were pretreated with 35μg of 17E6 (αv-blocking) antibody, prior to coinjection (s.c.) with matrigel, growth of parental VUP and V20 lines was unaffected while growth of the αvβ3 positive line, V15 was delayed by 3-4 weeks. These data indicate that an (αv-blocking) antibody 17E6 induces a growth retardation of αvβ3 positive, but not αvβ3 negative cells and suggests that signalling through the β3 subunit may contribute to in vivo tumour cell proliferation.

### P75 INTEGRIN EXPRESSION IN A PANEL OF CULTURED CELL LINES DERIVED FROM HUMAN GERM CELL TUMOURS

A. R. Champion*[1], M. F. Pera[2], R. Allman[1] and M. D. Mason[1], [1]Research Dept, Velindre Hospital, Cardiff, Wales, CF4 7XL, [2]Institute of Reproduction and Development, Monash University, Victoria 3168, Australia.

The integrin superfamily of cell membrane glycoproteins are known to be involved in the mediation of cellular adhesion to extracellular matrix components such as fibronectin, laminin and vitronectin. This study has examined the expression of an extensive array of these integrins in a panel of human germ cell tumour cell lines.

Determination of the integrin profiles of 7 embryonal carcinoma (EC) and 2 yolk sac carcinoma (YS) human cell lines has been performed via flow cytometric analysis of indirectly FITC-labelled monoclonal antibodies to cell surface integrins. Furthermore, analysis via Western blotting is currently being carried out to confirm integrin expression and assess sub-unit association.

Preliminary flow cytometry data have. so far shown strong expression of the $\alpha_4$ and $\beta_1$ integrins in all cell lines. In addition CD44 was found to be highly expressed in EC cells but not in YS cells. Finally, only weak expression of $\alpha_v$, $\beta_3$ and $\beta_5$ was observed for EC cells whereas YS cells were negative for all three.

It is known that EC cells will adhere and spread out on vitronectin. However when plated on fibronectin they will adopt a more dendritic morphology in their exploration of the substrate. We hypothesize that microspike extension, substrate exploration and migration of EC cells occurs on fibronectin and is mediated through $\alpha_4\beta_1$ in conjunction with EC cell surface keratan sulphate/chondroitin sulphate proteoglycan. Currently we are testing this hypothesis by blocking EC cell process extension and migration with appropriate antibodies to $\alpha_4$ and $\beta_1$ and by inhibition of chondroitin sulphate proteoglycan (CSPG). We are also exploring the role of CD44 and determining via immunoprecipitation whether the CD44 being expressed is a variant of CSPG.

### P76 DOWNREGULATION OF E-CADHERIN IN THE BREAST CANCER CELL LINE ZR75-1 WITH A DOMINANT NEGATIVE E-CADHERIN MUTANT,

Maria von Schlippe*[1], Alan J Zhu[2], Ian R Hart[1] and John F Marshall[1].
[1]Richard Dimbleby Department of Cancer Research/ICRF Laboratory, St Thomas' Hospital, Lambeth Palace Rd, London SE1 7EH, [2]Imperial Cancer Research Fund, 44 Lincoln's Inn Fields, London WC2A 3PX

E-cadherin, a member of a family of transmembrane glycoproteins which mediate calcium-dependent cell-cell adhesion, is expressed in all epithelia, and plays a role in morphogenesis, differentiation and maintenance of tissue integrity. E-cadherin has been implicated in tumour suppression, with loss of E-cadherin function correlating with dedifferentiation, increased invasiveness and poorer prognosis in various epithelial cancers. Possible mechanisms for this effect include loss of adhesiveness (through reduced amounts of E-cadherin or through a loss of the link to the cytoskeleton) and an alteration in signalling (possibly through β-catenin or the EGF receptor pathway).

As a means of studying the role of E-cadherin in breast cancer, we have reduced E-cadherin function in the E-cadherin-positive breast cancer cell line ZR75-1 by the use of a dominant negative mutant. The construct encodes the extracellular domain of the murine MHC class I molecule $H\text{-}2K^d$ linked to the transmembrane and cytoplasmic portions of mouse E-cadherin (Zhu and Watt, Journal of Cell Science 109, 3013, 1996). The resultant cell line (ZR-E) grew as a monolayer with distinct refractile edges, while the cell-cell borders in the parental line were not visible. The $H\text{-}2K^d$ domain was detectable by FACS analysis and indirect immunofluorescence, and α, β and γ-catenins were immunoprecipitated with anti-$H\text{-}2K^d$ antibody SF1-1.1, confirming the activity of the inserted mouse E-cadherin cytoplasmic tail.

FACS analysis revealed a two-fold downregulation of endogenous surface E-cadherin, confirmed by Western blotting. On indirect immunofluorescence, the residual E-cadherin was largely extractable with Triton X-100, suggesting a loss of anchorage to the cytoskeleton. Functional analysis of the ZR-E line compared with ZR75-1 showed it to be less cohesive, less motile in wound-healing assays, and unable to form spheroids. These findings indicate that abrogation of E-cadherin activity has profound effects on the biological behaviour of breast cancer cells.

### P77 THE USE OF A MURINE MODEL OF FAMILIAL ADENOMATOUS POLYPOSIS TO STUDY THE VARIATION IN E-CADHERIN AND β-CATENIN EXPRESSION.

D.J. Clark*, S.A. Watson, T.M. Morris and J.H. Scholefield. Academic Unit of Cancer Studies, Division of GI Surgery, University of Nottingham UK.

E-cadherin, a cell adhesion molecule, has been widely employed as a marker in a variety of human tumours[1] including colorectal cancer. β-catenin, a protein believed to regulate enterocyte proliferation and apoptosis, binds with E-cadherin as part of the cadherin-catenin complex which is of importance in cell adhesion. The product of the tumour suppressor gene, APC, competes with E-cadherin for binding of β-catenin [2]. The expression of both E-cadherin and β-catenin has been shown to be reduced in colorectal cancer.

A mutant mouse lineage which exhibits inherited predisposition to multiple intestinal neoplasia (Min) has long been used as a model to study familial adenomatous polyposis (FAP). Both the Min/+ mouse and patients predisposed to FAP carry a germline mutation in the *Apc* gene[3].

The aim of this study was to utilise the Min mouse model to compare the level of expression of both E-cadherin and β-catenin from early through to late stages of malignancy.

*Min/+* mice were terminated at either 4 weeks, 8 weeks or 12 weeks of age and the large intestine removed. Tumour and surrounding tissue were excised from five areas along the length of the intestine, formalin fixed and paraffin embedded. The intestinal mucosal samples were analysed by immunohistochemistry to examine the expression of both E-cadherin and β-catenin. Control samples were obtained from representative areas of the large intestine of both *Min/-* and C57Bl mice terminated at the same age.

Tissue sections were examined for differences in the expression of E-cadherin and β-catenin between the normal intestinal mucosa and that of the tumour. Essentially the level of expression of both proteins was found to be correlated with the degree of malignancy when compared to the normal. Membranous versus cytoplasmic staining of the proteins was found to vary between the samples,with a loss of membranous staining indicating a reduction in cell adhesion.

This study has enabled some of the early changes which may occur in colorectal cancer to be identified.

1. Birchmeier, W. and Behrens, J. (1994) *Biochemica Et Biophysica Acta-Reviews On Cancer* **1198** 11-26.
2. Rubinfeld *et al.*, (1995) *J Biol Chem* **270** 5549-5555.
3. Su *et al.*, (1992) *Science* **256** 668-670.

### P78 NOVEL UROTHELIAL-SPECIFIC GENE EXPRESSION IDENTIFIED USING DIFFERENTIAL DISPLAY RT-PCR.

G. Hall*, B.A. Smith, K. Geall, P.J. Selby & J. Southgate. ICRF Cancer Medicine Research Unit, St James's University Hospital, Leeds, LS9 7TF

Tissue-specific genes provide information regarding the structure and/or function of that tissue and may be used as molecular markers of a particular cellular phenotype. The ability of the associated promoter / enhancer elements of such genes to direct transgene expression in a tissue specific manner has been exploited to produce specific cancer models and direct therapeutic gene expression. Our aim was to identify novel urothelial specific genes with expression retained following neoplastic transformation.

Differential display RT-PCR was performed with 30 primer combinations on autologous DNAse treated mRNA from bovine lung, oesophagus, bladder, liver and spleen. 21 bands derived from cDNA products suggesting urothelial specific mRNA expression were isolated and T-A cloned into pGEM vectors. Database homology was assessed for the 33 discrete differential display products identified by sequencing. Sequences identified included uroplakins Ia and II, two of the three genes previously known to have urothelial specific expression. 19 of the sequences had no homology with sequences on the GenBank database.

Ribonuclease protection assays were performed to confirm the tissue specificity of a number of the cloned products. Three of the seven novel sequences tested were confirmed to exhibit urothelial-specific expression.

A bovine urothelial cDNA library was screened using riboprobes generated from the differential display products. Four discrete clones ranging between 700 and 1650 bp were isolated for one of these sequences. More than 1000 bases of additional coding region sequence was obtained by PCR of the cDNA library using vector and sequence specific primers. RT-PCR of both coding region and 3' UTR confirmed the urothelial specificity of the gene.

Analysis of the assembled sequence identified significant homology within the coding region to previously described urea transporters, a family of proteins responsible for the active transport of urea across plasma membranes. This suggests the identification of a novel gene which acts as a urea transporter within the urothelium of the urinary tract.

### P79 A FLOW CYTOMETRIC ANALYSIS OF MUCIN ANTIGEN EXPRESSION USING THE C595 ANTI-MUC1 ANTIBODY.

S.Fritchley, M.Price[1], E.Petrakou[1], J.Lawry*. Institute for Cancer Studies, University Medical School, Sheffield. S10 2RX, and Cancer Research Labs.[1], University of Nottingham, University Park, Nottingham, NG7 2RD.

Normal and malignant breast epithelia express and often secrete mucins, and their cellular upregulation has been reported in breast tumours. Elevated serum levels post-surgery may reflect the subclinical development of metastatic disease.

The C595 anti-MUC1 antibody would not titrate on T47D breast carcinoma cell lines (median channels 213 to >609, control= 2.1) so the MCF7 breast carcinoma cell line was used having a lower level of expression of MUC1 (Med. Ch 10, contol 2.0). MUC1 expression was upregulated by cytokines (30% increases with G-CSF, GM-CSF, and IL-1; and 120% increase with IFNγ).In contrast, the intercellular adhesion molecule (ICAM-1, CD54) was upregulated by both IL-1 (150%) and IFNγ (300%).

Association and dissociation studies were undertaken with FITC conjugated C595 (F:P ratio 6:1) at room temperature over 10-20 minutes. Cells were pre-labelled with unconjugated Ab, then exposed to excess FITC conjugate, or vice verca, and changes in fluorescence detected over time (dissociation); or fluorescence measured after adding FITC C595 alone (association).

The association constant of 5ul (=5ug) FITC Ab was calculated at $9.93 \times 10^{-10}$ml/cell.second, with a very rapid binding rate (<30 seconds) and a long off rate (>20 minutes). Conjugated antibody had a slower dissociation rate than the unconjugated form.

**Funding was provided by Yorkshire Cancer Research.**

### P80 PRODUCTION OF A RECOMBINANT ANTI-MUC1 MUCIN DIABODY.

Graeme Denton*, Kevin Brady, Andrea Murray and Michael R. Price. Cancer Research Laboratory, School of Pharm. Sci., Univ. of Nottm., NG7 2RD. UK.

MUC1 mucins are highly glycosylated glycoproteins expressed on the lumenal surfaces of glandular epithelia. Apart from their major physiological functions as protective agents and biological lubricants, they are frequently elevated and/or altered in cancer and thus have potential as tumour markers.

The present investigation was initiated to produce derivatives of anti-MUC1 mucin antibodies which would have the potential for use in tumour targeting. Here we describe the production of a recombinant diabody fragment incorporating the variable region of the anti-MUC1 mucin monoclonal antibody C595, using a bacterial expression system used to produce C595 scFv[1]. The shortened linker method[2] was employed to alter the C595 scFv linker sequence between $V_H$ and $V_L$ from $(Gly_4Ser)_3$ to $Gly_6Ser$. This was achieved using a four primer PCR system. The $Gly_6Ser$ linker sequence in the expressed protein no longer allows intramolecular pairing of the $V_H$ and $V_L$ domains of the scFv as highlighted by modelling studies. These domains then pair with complementary domains on neighbouring molecules, to form a bivalent monospecific scFv dimer (diabody).

Detailed analyses of the binding characteristics of the C595 diabody show these to be similar to those of the parental monoclonal. Its ability to bind to MUC1 expressing tumours and cell lines identifies it as a potential targeting reagent so that development of diagnostic and therapeutic applications are appropriate.

[1]Denton *et al* (1997) Brit. J. Cancer. 76: 614-621.
[2]Holliger *et al* (1993) PNAS USA. 90: 6444-6448.

### P81 ERRB-2 MEDIATES THE EFFECTS OF HEREGULIN ON CELL MOTILITY BUT NOT PROLIFERATION OF T-47D CELLS,

Carol Sawyer and Christopher Dean*, The Institute of Cancer Research, McElwain Laboratories, Sutton, Surrey, UK

Over-expression of the receptor for EGF (EGFR) and the related product of the c-erbB-2 proto-oncogene ($p185^{erbB-2}$) have been found to be indicators of poor prognosis in patients with squamous cell carcinoma or adenocarcinoma. While a number of growth factors have been described which bind to and activate the EGFR none has been found as yet which binds directly to $p185^{erbb-2}$. Recently, a series of growth factors collectively called the neuregulins and including the heregulins, glial growth factors, ARIA and neu differentiating factor, have been described which have been found to bind to two other members of the EGF receptor family namely erbB-3 (which lacks endogenous kinase activity) and erbB-4 but not to the EGFR or erbB-2. However, the binding of these growth factors can be followed by activation not only of the receptors themselves but also of the EGFR and $p185^{erbB-2}$. It is becoming increasingly clear that cross-talk between the different members of the EGFR family, possibly as a result of hetero-dimerisation, is important in determining signal transduction via these receptors. We have been investigating the role of the EGF receptor family in transmitting the biological effects of one of the neuregulins, namely, heregulin-β1 and have used a series of rat monoclonal antibodies directed against different epitopes on the extracellular domain of $p185^{erbB-2}$ to investigate the role of this receptor in signal transduction. Our results show that while heregulin activates both erbB-2 and erbB-3 and stimulates the proliferation of the breast cancer cell line T-47D, it also acts to restrict cell migration. One of the antibodies to $p185^{erbB-2}$ (ICR12) inhibits the high affinity binding of $^{125}$I-heregulin-β1 to T-47D cells and prevents the activation of this receptor as judged by receptor phosphorylation. As a consequence, co-treatment with heregulin-β1 and ICR12 reverses the effect of this growth factor on cell motility and/or adhesion but does not prevent the ligand-induced stimulation of proliferation of the cells. We conclude that in T-47D cells signalling via erbB-2 is important for the control of cell migration.

### P82 AUTOCRINE FACTOR(S) INVOLVED IN THE CONTROL OF NORMAL HUMAN UROTHELIAL CELL GROWTH.

S. Pellegrin*, P. J. Selby, L. K. Trejdosiewicz & J. Southgate. ICRF Cancer Medicine Research Unit, St James's University Hospital, Leeds LS9 7TF.

Urothelium, the precursor for transitional cell carcinoma (TCC), is a slow turnover epithelium with high proliferative potential during regeneration or in monolayer culture *in vitro* (Southgate *et al. Lab Invest 1994*: **71**: 583). However, the mechanisms involved in regulating normal human urothelial (NHU) cell proliferation are unclear.

Cell cycle analysis was performed by flow cytometry and showed that freshly isolated NHU cells were arrested in G0/G1. By contrast, NHU cells in culture were distributed in G1-S-G2 phases and arrested in G1 at confluence. Proliferation of NHU cells *in vitro* was monitored by growth curves and tritiated thymidine incorporation. NHU cells in culture showed a high proliferation rate with an estimated population doubling time of 15h. NHU cells were not responsive to exogenous EGF and retained a high proliferation rate even when cultured in serum-free media with no exogenous growth factors.

Conditioned medium from NHU cells was mitogenic for mouse NIH3T3 fibroblasts, suggesting secretion of autocrine/paracrine growth factor(s). Furthermore, NHU cell proliferation was wholly inhibited by antagonistic anti-EGFR antibodies. Incomplete growth inhibition could be observed with neutralising antibodies to the EGFR-ligand Amphiregulin and NHU cells expressed mRNA for Amphiregulin by RT-PCR.

These data suggest that a major component of growth regulation of NHUC involves autocrine growth factors of the EGF family. This finding has important implications for growth regulation in urothelial tissue homeostasis, repair and tumour development.

### P83 THE ROLE OF THE EXTRACELLULAR MATRIX, TGFβ AND β1-INTEGRIN IN THE REGULATION OF NORMAL HUMAN UROTHELIAL CYTODIFFERENTIATION.

W.J. Kennedy, L.K. Trejdosiewicz & J. Southgate. ICRF Cancer Medicine Research Unit, St James's University Hospital, Leeds LS9 7TF, UK.

Neoplastic transformation is associated with dysregulation of epithelial cytodifferentiation. In the uroepithelium of the urinary tract, terminal cytodifferentiation is characterised by the acquisition of a unique apical asymmetric unit membrane and expression of urothelium-specific antigens. NHU cells propagated *in vitro* in serum-free medium have a proliferative basal/intermediate phenotype, but do not express urothelial differentiation antigens. We have investigated the effects of culturing NHU cells on Matrigel, which has been shown to induce differentiation in other epithelial cell systems.

NHU cells were maintained for 3 - 7 days on Primaria plastic dishes or on gels composed of Matrigel, in the presence or absence of cytokines or blocking antibodies. Cells were dissociated from matrices using collagenase and dispase. Phenotype was determined by indirect immunofluorescence.

NHU cells on Matrigel demonstrated *de novo* expression of the urothelial differentiation-associated antigens, UMA and AUM, in respectively 12/13 and 21/23 cell lines from independent donors. A less marked induction was noted when NHU cells were plated on collagen I matrices. On Matrigel, induction of differentiation antigen expression was blocked by either a TGFβ neutralising antibody or a β1-integrin functional activator antibody, whereas the induction of differentiation was unaffected by a neutralising β1-integrin antibody. Neither TGFβ nor the β1-integrin neutralising or activating antibodies alone were able to induce differentiation of NHU cells grown on Primaria dishes.

These data suggest that induction of urothelial differentiation at a minimum requires both TGFβ and modulation of β1-integrin interactions with an appropriate extracellular matrix.

### P84 TGF-β DOWNREGULATES IMMUNE RESPONSES AND PROMOTES TUMOUR CELL MIGRATION

Toomey, D., Harmey, J., Bouchier-Hayes, D. Dept. Surgical Research, Beaumont Hospital and R.C.S.I.

Tumour expansion is regulated by the balance of positive and negative growth factors in the tumour environment.Tumour growth factors include angiogenic cytokines such as VEGF, PDGF and TGF-$\beta_1$. Some tumour promoting agents also have immunosuppressive properties, thus promoting tumour growth both directly and indirectly. We have previously shown that breast tumours produce large amounts of TGF-$\beta_1$. This study examines the effects of TGF-$\beta_1$ on leucocyte anti-tumour function and on tumour cell migration through endothelium.

Lymphocyte anti-tumour cytotoxicty was assessed against the metastatic breast carcinoma cell line, MDA-MB-231, following lymphocyte activation with rhIL-2 (1000 U/ml) and 24 hour incubation with TGF-β (0-5.0 ng/ml). When IL-2 activated lymphocytes were incubated with TGF-$\beta_1$ (1-5 ng/ml), cytotoxicity was reduced to negligible levels ($p<0.05$ control vs treated groups). Macrophage effector function was examined by investigating spontaneous nitric oxide (NO) and TNF-α production following 24 hour incubation with TGF-$\beta_1$ (0-7.5 ng/ml). Macrophage function was examined under both normoxic and hypoxic growth conditions, such as those found within solid tumours. The addition of TGF-$\beta_1$ (2.5-5.0 ng/ml) to macrophage cultures under hypoxic conditions significantly decreased macrophage production of both NO and TNF-α ($p<0.05$ control vs treated groups). Transendothelial tumour cell migration was assessed following 4 hour incubation with TGF-$\beta_1$. Intermediate doses of TGF-$\beta_1$ (2.5 -5.0 ng/ml) resulted in increased tumour cell migration ($p<0.05$ control vs treated groups).

TGF-$\beta_1$ is a pro-angiogenic cytokine; we have also shown that TGF-$\beta_1$ may play a role in the metastatic process by promoting tumour cell migration. The immunoregulatory properties of TGF-$\beta_1$ are such that macrophage and lymphocyte anti-tumour function is suppressed. In conclusion, TGF-$\beta_1$ production in the tumour environment affects both immune function and the metastatic process and may explain the observed failure of IL-2 therapy for breast cancer.

### P85 CELL ADHESION MOLECULES AND NM23H1: RELATIONSHIP TO TGF-BETA IN BREAST CANCER

AI Ibrahim*, J Lawry and JA Royds, Institute for Cancer Studies, Sheffield University Medical School, Beech Hill Road, Sheffield, S10 2RX.

A link has been seen between nm23H1, a putative metastasis suppressor gene and TGFβ1, a cytokine generally growth inhibitory to epithelial cells. An altered response to TGFβ1 has been observed in soft agar colonisation assays, nm23H1 transfectants having a reduced response to the cytokine when compared to their corresponding controls [1]. Furthermore, it has been suggested that there are two separate TGFβ1 pathways. One involves nm23H1 and functions in cells of lower metastatic potential, where cells show a growth arrest response to TGFβ1. The second TGFβ1 pathway functions in cells which have progressed to a more metastatic phenotype, not involving nm23H1, and results in an increased invasive, motile and proliferative response to the cytokine [2].

We have used flow cytometry to investigate the link between nm23H1 and TGFβ1 by the analysis of proteins expressed in the cell which may be important in the metastatic cascade. Six breast cancer cell lines were used, the rarely metastatic MCF7 and T47D and the highly metastatic MDAMB435 and its transfectants K27, (vector-only control), H1177 and P57 (high and medium-high expressors of nm23H1 protein, respectively).

Cell surface ICAM-1 was not expressed at a detectable level in the T47D and MCF7 lines. In contrast MDAMB435 and all transfectants had increased levels, this being slightly up-regulated (up to 20%) after TGFβ1 treatment. No difference in ICAM-1 was observed due to nm23H1 transfection. No significant changes were observed in TGFβ1 Receptor I expression, however Receptor II was down-regulated by up to 25% in response to TGFβ1 treatment in all cell lines tested, except the highly over-expressing H1177, which in contrast, showed an up-regulation of approximately 60% in comparison to its untreated control. Nm23H1 levels in T47D, MCF7 and the MDAMB435 line were reduced by TGFβ1 treatment in the ranges of 40%, 25% and 20%, respectively. E-cadherin was not detectable in the MDAMB435 and transfectant lines, but was expressed at higher levels in the T47D and MCF7 lines. All cell lines tested negative for α2β1 integrin. TGFβ1-induced growth inhibition was displayed in all lines except T47D, where no significant response was observed.

Our results indicate that responses to TGFβ1 via nm23H1 may function by differential expression of TGFβ1 Receptor II. Cytokine modulated expression of nm23H1 and various cell adhesion molecules may be a few of many contributing factors involved in the complex process of metastasis.

**References:** [1] Leone A, Flatow U, Van Houtte K, Steeg PS. Oncogene 8, 2325 (1993). [2] Hsu, S., Huang, F., Wang, L., Banerjee S., Winawer, S., Friedman, E. Cell Growth & Differ. 5, 909 (1994).

### P86 EFFECTS OF THE TUMOR CELL INVASION INHIBITOR PHENYLACETATE ON CELLULAR pH AND DRUG SENSITIVITY OF CARCINOMA CELL LINES

G. Hamilton*, I. Haberl, E. Cosentini, G. Theyer* and E. Wenzl. Dept. of Surgery, University School of Medicine and *LBI of Clinical Oncology, A-1090 Vienna, Austria.

Phenylacetate (PA) is a nontoxic cell differentiation inducer, which has been shown to reduce the invasiveness of prostate cancer cells in in vitro assays (Samid D, et al. J Clin Invest. 91: 2288-2295, 1992) and is currently in phase I trials for solid tumors (Thibault A, et al. Cancer Res 54: 1690-1694, 1994). In our experiments with prostate cancer cell lines PA in low concentrations (0.25 - 1mM) stimulated cell proliferation as measured by MTT assays and pretreated DU-145 and PC3 cells (4 day PA exposure) show increased sensitivity to camptothecin (CPT) and doxorubicin (35 - 45% sensitization). This effects were found in cells attached to substrate, but not for suspension cultures, which exhibit reduced growth and higher resistance to CPT and doxorubicin. In low-density cell cultures PA increased the S-fraction by 45% within 4 days as measured by propidium iodide staining and flow cytometry cell cycle analysis. The fraction of apoptotic cells in PA-pretreated cultures exposed to CPT and doxorubicin as detected by annexinV-binding assays and sub-G1-peaks in cell cycle analysis was significantly increased. In spectrofluorimetric measurements of intracellular pH ($pH_i$) using BCECF-stained adenocarcinoma cell lines (colon, breast, prostate) in HEPES or bicarbonate buffer the cells exhibit a reversible $Na^+$-dependent decrease of 0.1 - 0.2 pH units in response to 0.25 - 0.5 mM PA and this decrease in $pH_i$ is also observable under acidic extracellular conditions prevailing in tumors. According to these results PA not only reduces the metastatic potential of carcinoma cells, but in addition sensitizes in low concentrations the tumor cells to cytotoxic drugs such as doxorubicin and CPT and lowers intracellular pH, which stabilizes the active lactone form of camptothecines.

### P87 REDUCED EXPRESSION OF ENDOTHELIAL AND INDUCIBLE NITRIC OXIDE SYNTHASE IN A HUMAN BREAST CANCER CELL LINE WHICH HAS ACQUIRED ESTROGEN INDEPENDENCE

O. Alalami*[1], H. W. van den Berg[2] and J.H.J Martin[1]. [1]Division of Biomedical Sciences, School of Health Sciences, University of Wolverhampton, England. WV1 1DJ. [2]Dept. of Therapeutics and Pharmacology, Queen's University of Belfast, Belfast, Northern Ireland, BT9 7BL.

We have recently demonstrated the existence of the L-arginine-nitric oxide pathway and presence of both endothelial (eNOS) and inducible (iNOS) nitric oxide synthases in the ZR-75-1 human breast cancer cell line. We have also shown that expression of eNOS is inversely correlated to histologic grade in primary invasive ductal breast carcinomas. The aim of the present study was to investigate possible effects of estrogen independence on NOS expression using a variant of the ZR-75-1 cell line (ZR-PR-LT) which has acquired estrogen independence.

Immunohistochemical studies used the primary antibodies, bovine anti-endothelial nitric oxide synthase (599-613) and mouse macrophage anti-inducible nitric oxide synthase (1131-1144). $NO_2^-$ concentration in conditioned medium was quantitated by the Greiss reaction.

The percentage of cells staining positive for eNOS and iNOS is shown in the table below. ZR-PR-LT exhibited much fewer positively staining cells compared to the parent cell line.

| | % Positive cells (eNOS) | % Positive cells (iNOS) |
|---|---|---|
| ZR-75-1 | 100 | 52 |
| ZR-PR-LT | <5 | 0 |

ZR-PR-LT cells therefore secreted significantly lower ($p<0.001$) levels of nitrite into the conditioned medium compared to ZR-75-1 cells.

We have investigated the effects of various drugs on nitrite secretion by the ZR-PR-LT cell line. Neither phorbol 12-myristate 13-acetate (200-1000nM) nor tamoxifen ($10^{-9}$M-$10^{-6}$M) had a significant effect on nitrite production, whereas progesterone ($10^{-10}$M-$10^{-6}$M) significantly ($p<0.05$) reduced nitrite secretion.

In conclusion, our results show that estrogen independence in human breast cancer cells is associated with reduced NOS expression and support the suggestion that lack of NOS expression may be related to the degree of malignancy in human breast cancer.

### P88 ROLE OF NITRIC OXIDE IN THE RESPONSE OF TUMOURS TO ISCHAEMIA-REPERFUSION INJURY.

CS Parkins*[1], DR Collingridge[1,2], MF Dennis[1], SA Hill[1], AL Holder[1], MRL Stratford[1], LL Thomsen[3] and DJ Chaplin[1]. [1]Gray Laboratory Cancer Research Trust, Mount Vernon Hospital, Middlesex, UK; [2]Yale University School of Medicine, New Haven, USA; [3]Glaxo Wellcome Research and Development, Hertfordshire, UK.

Tumour cytotoxicity induced by ischaemia-reperfusion (I/R) injury is mediated, in part, by oxidative stress to the vasculature and can be attenuated by the capacity of tumours to produce nitric oxide (NO). The role of NO was examined in two murine tumours, of differing NO production and sensitivity, to I/R injury and their response to: i) inhibition of NO synthase (NOS) or, ii) increased cellular oxygenation. Tumour cytotoxicity was enhanced by inhibition of NOS at the time of I/R injury although the extent of this inhibition was dependent on the intrinsic production of NO. Tumour production of NO was measured from the accumulation of nitrite and nitrate (NOx), the oxidised products of NO, by ion chromatography analysis of media from cultured tumour cell lines or whole tumour explants. *In vitro* production of NOx by SaS tumour cells was significantly greater than that by CaNT tumour cells, the same pattern being observed with whole tumour explants. Elevation of cellular oxygenation resulted in increased NO production by SaS tumour cells both *in vitro* and *in vivo*, sampled using microdialysis probes in solid tumours. The increase was inhibited by administration of the iNOS inhibitor, 1400W (Glaxo Wellcome). Following reperfusion, tumour oxygenation improved in the SaS tumour only, indicating modest vascular damage, possibly mediated by improved NO production. The present study indicates that NO production is heterogeneous between tumour types and can contribute to resistance to oxidative stress. NO production by tumours, and its oxygen dependence, may be exploitable in cancer therapies which mediate cytotoxicity via cellular oxidative stress and therapies designed to modify tumour oxygenation.

*This study is supported by the Cancer Research Campaign.*

### P89 The Role of the Microvascular Pericyte in the Tumour Microenvironment.

A.D. Martin, J.R. Bailie, S.R. Mckeown & D.G. Hirst. Radiation Science Group, School of Biomedical Sciences, University of Ulster at Jordanstown, Newtownabbey, Co. Antrim, Northern Ireland, UK. BT37 0QB.

Solid tumors must stimulate the growth a blood supply if they are to grow to a volume greater than 1mm$^3$. This process known as tumour-induced angiogenesis occurs rapidly under the influence of factors released by the tumour cells[1]. These vessels differ markedly from normal tissue microvasculture, being rapidly proliferating, tortous, thin walled, chaotically arranged, maximally dilated and respond poorly to vasoactive agents[2]. Another feature of tumour capillaries is the loss of contact between pericytes and endothelial cells and a reduced extension of pericyte processes[3]. In normal capillaries, pericytes exist in close association with the underlying endothelium either making direct contact with it via numerous processes or separated from it by basal lamina. One of the many proposed functions of pericytes is the control of endothelial cell growth and microvessel tone. Pericytes have been shown to inhibit the growth of endothelial cells *in vitro* and to have an effect on capillary blood flow. They have been shown to contract in response to endothelin and relax in response to nitric oxide, both potent vasoactive agents[4]. It is our hypothesis, therefore, that reduced pericyte/endothelial cell contacts or reduced pericyte numbers are important factors in the lack of regulation of endothelial cell growth and microvessel tone at the site of a tumour. Pericytes may, therefore, regulate the expression of vasoactive or growth related genes in endothelial cells.

We have examined the effects of media conditioned by a human colon carcinoma cell line HT-29 (TCM) or bovine retinal microvascular pericytes (PCM) on gene expression in the human microvascular endothelial cell line HMEC-1. We have found that after a 6hr exposure to TCM, HMEC-1 showed a five fold increase in the levels of expression of inducible nitric oxide synthase (iNOS) mRNA and protein. No significant change was observed in the levels of constituitive NOS (cNOS) mRNA or protein. In comparison, after 3hrs exposure to PCM, HMEC-1 showed a five fold decrease in expression of iNOS mRNA and protein. Again no significant change was observed in the levels of cNOS expression. After 5hrs exposure to PCM, HMEC-1 exhibited a three fold increase in the levels of mRNA for endothelin-1. Using the technique of differential mRNA display, we have shown that there are many clear differences in gene expression in HMEC-1 after six hours exposure to PCM. A total of eight candidate genes have now been cloned, sequenced and where possible identified. It is apparent that most of these genes have a role to play in blood vessel development. The identification of candidate genes involved in blood vessel growth and regulation could yield new targets for therapeutic intervention to restrict neovascularization or control tumour blood flow.

**The authors acknowlegde grant support from the Departmant of Enducation (NI) and the Cancer Research Campaign.**

1. Folkman J (1972). *Ann. Surg.* **175**: 409-416.
2. Peterson HI (1991). *Int. J. Radiat. Biol.* **60**: 201-210.
3. Bertossi M, Virgintino D *et al.*,(1997). *Ultrastructural Pathology* **21**: 41-49.
4. Sims DE (1991). Can J Cardiol 7: 431-443.

### P90 VASCULAR ENDOTHELIAL GROWTH FACTOR EXPRESSION IN LUNG CANCER.

KJ O'Byrne*[1], A Giatromanolaki[2], MI Koukourakis[2], H Turley[3], PAE Scott[3], F Pezzella[3], AL Harris[3], KC Gatter[3], [1]Leicester Royal Infirmary, UK, [2]University Hospital of Iraklion, Crete, Greece and [3]Oxford Radcliffe Hospitals, UK.

Angiogenesis is essential for tumour growth beyond 1 to 2 mm in diameter and plays a key role in the metastatic spread of cancer. Vascular endothelial growth factor (VEGF) is a cytokine involved in tumour angiogenesis. Human cells express 4 different VEGF molecules of 121, 165, 189 and 206 aminoacids which are all encoded by a gene located on chromosome 6p21.3. Employing the VG1 antibody, which recognises epitopes on the 121, 165 and 189 VEGF molecules, and the streptavidin-biotin immunohistochemistry technique we evaluated tissue sections of tumour samples from 144 patients with lung cancer for the expression of VEGF. VEGF expression was correlated with T-stage, N-stage, histological subtype and grade, proliferation index and microvessel density, and nuclear and wild type cytoplasmic/perinuclear p53, bcl-2, EGFR and c-erbB-2 oncoprotein expression. Strong VEGF immunoreactivity in $\geq$50% of tumour cells was seen in 65/120 (54%) non-small cell lung cancer (NSCLC) samples. No expression was detected in small cell lung cancer. High vascular grade tumours had significantly more VEGF immunoreactive cells than low vascular grade tumours, the association being significant for squamous cell tumours only (p=0.0002). No association was found between p53 expression and vascular grade or prognosis. However linear regression analysis showed a significant inverse correlation between VEGF and cytoplasmic/perinuclear p53 expression (p=0.003; r=0.26). Also subset analysis revealed microvessel counts to be significantly higher in cases with positive VEGF expression and absent cytoplasmic/perinuclear p53 staining *vs* VEGF negative, cytoplasmic/perinuclear p53 positive (p=0.03) or VEGF negative, cytoplasmic/perinuclear p53 negative cases (p=0.007). High VEGF expression was associated with a poor prognosis in N0 disease (p=0.03). No association was found between VEGF expression and the other tumouir parameters studied. In conclusion the results indicate that VEGF expression in NSCLC is associated with angiogenesis, loss of wild type cytoplasmic/perinuclear p53 immunoreactivity and a worse prognosis.

### P91 PHAGE-DERIVED SINGLE CHAIN FV ANTIBODY FRAGMENTS TO VASCULAR ENDOTHELIAL CELL GROWTH FACTOR.

SP Cooke*, L Lawrence, GM Boxer, KA Chester and RHJ Begent. CRC Targeting & Imaging Group, Dept. of Clinical Oncology, Royal Free and UCL Medical Schools, London NW3 2PF

Vascular endothelial cell growth factor (VEGF) is an endothelial cell-specific mitogen which promotes growth and angiogenesis. It has been shown in model systems that in vivo administration of anti-VEGF antibodies[1] or blockade of the VEGF receptor on endothelial cells[2] inhibits tumour angiogenesis and consequently tumour growth. VEGF is thus a strong candidate for anti-angiogenic cancer therapy.

Anti-VEGF antibodies were produced using filamentous phage technology as previously described[3]. Phage expressing single chain Fv (scFv) were subjected to 4 rounds of panning on VEGF coated tubes to isolate those reactive with VEGF. Five of these were selected for further analysis on the basis of their DNA sequence diversity, intensity of signal in VEGF binding ELISA and by immunohistochemistry to assess reactivity with blood vessel endothelium.

The selected scFv were subcloned into an expression plasmid incorporating a $His_6$ tag. scFv proteins were purified from culture supernatants by metal chelate chromatography with yields ranging from 6 to 60mg/L. Purified scFV were biotinylated and used in immunohistochemical studies to examine binding to human placenta, normal human colon, human colorectal carcinoma and human colorectal xenografts (LS174T; SW116; SW122).

Three of the five scFv clones tested did not show significant reactivity with any of the tissue sections tested. A further clone showed weak staining for placental trophoblast and blood vessel endothelium and weak binding to stromal cells in the other tissue sections but did not show any specificity for blood vessels in human tumours or human tumour xenografts. scFv clone L4.6 however reacted with both blood vessels endothelium and syncitial cells in term placenta and was positive for both blood vessel endothelium and stromal cells in colorectal carcinoma and human xenograft tissue. Reactivity with the epithelium of the human carcinoma and with the xenograft tumour cells was heterogeneous. Blood vessels in normal colon were also positive.

scFv clone L4.6 targets blood vessels in human tumours and human tumour xenografts and therefore merits further study as a vehicle for antibody-directed delivery of agents to tumour tissues in both animal models and as a model for potential use in patients.

Refs
1. Millauer, B. et al. (1994) Nature **367**; 576-579
2. Kim, KJ et al. (1993) Nature **362**; 841-846
3. Lawrence, L et al (1996) Br. J. Cancer **71**: suppl. XXIV

### P92 INTRODUCTION OF ENDOTOXIN DURING SURGERY INCREASES METASTATIC TUMOUR GROWTH AND CIRCULATING VEGF.

G.Pidgeon*[1], J.H.Harmey[1], E.Kay[2], H.P.Redmond[1], D.J.Bouchier-Hayes[1]. Depts' Surgery[1] and Pathology[2], Beaumont Hospital, Royal College of Surgeons Ireland, Dublin 9, Ireland.

**Introduction :** The surgical resection of primary tumours may paradoxically induce rapid metastatic growth, resulting in post-operative tumour recurrence. We hypothesised that endotoxin (LPS) introduced at the time of surgery contributes to increased growth of metastases by inducing tumour cell proliferation and stimulating production of vascular endothelial growth factor (VEGF), a potent angiogenic factor. **Methods:** 50 Balb/c mice were injected with $10^5$ 4T1 mammary adenocarcinoma cells by tail-vein injection. On day14 the animals received either anaesthetic alone, laparoscopy under $CO_2$ to exclude air, laparoscopy with air, mid-line laparotomy (open surgery), saline injection or LPS injection (10μg). Lungs were excised on day19 and weighed as an index of metastatic burden. Mitotic indices within metastases were examined by histology with H&E staining and tumour cell apoptosis by TUNEL staining. Plasma LPS levels following surgery was examined by the LAL assay and serum VEGF by ELISA. **Results:** A significantly higher metastatic burden was observed in both the laparotomy and air laparoscopy groups compared to either control or $CO_2$ laparoscopy groups (p<0.02). No significant difference was observed between controls and $CO_2$ laparoscopy groups. Metastatic burden was also significantly higher in the endotoxin injection group compared to saline controls (p<0.001). Increased metastatic burdens coincided with significantly increased mitotic indices within lung metastases (p<0.05) in the surgical and injection groups. Both plasma LPS and serum VEGF levels were significantly elevated following open surgery or laparoscopy with air (p<0.05) and a significant positive correlation was observed when LPS and VEGF was examined in all groups (r=0.99). **Conclusion:** Our results show that open surgery and/or endotoxin increase tumour cell growth, metastasis and circulating VEGF. Reducing surgical exposure to LPS may attenuate post-operative metastatic tumour growth.

**Abbreviations:** Lipopolysaccharide (LPS), Vascular Endothelial Growth Factor (VEGF), Limulus Ameobocyte Lysate (LAL).

### P93 INTERLEUKIN-6 INCREASES PROLIFERATION AND VEGF PRODUCTION IN BREAST CANCER.

A. Haverty, J. Harmey, H.P Redmond, D.J. Bouchier-Hayes. R.C.S.I., Dept. of Surgery, Beaumont Hospital, Dublin 9.

**Introduction:** Interleukin-6 (IL-6) is a proinflammmatory cytokine secreted by monocytes, macrophages, endothelial cells and a number of tumour cell lines. We have previously shown IL-6 is significantly higher in breast tumour tissue and serum of breast cancer patients compared to normal controls. To determine the role of IL-6 within the breast tumour microenviroment we investigated the effect of IL-6 treatment on proliferation and vascular endothelial growth factor (VEGF) production. of MDA-MB231 cells, an estrogen receptor negative metastatic tumour cell line,
**Methods**: MDA-MB231 cells were plated at 5 x $10^5$ cells/ml, and allowed to adhere for 24 hours. Cells were then treated with 0 - 1000 pg/ml IL-6 for 24 hours and proliferation rates determined using the Cell Titre proliferation assay. To determine the effect of IL-6 on angiogenic factor production, MDA-MB231 cells were plated at 2 x $10^6$ cells /ml, and allowed to adhere for 24 hours. Cells were treated with 500 pg/ml IL-6 for 24 hours. Supernatants were assayed for VEGF using ELISA, and cell lysates assayed for protein using the B.C.A. assay.
**Results:** IL-6 treatment of MDA-MB231 cells increased proliferation in a dose dependent manner. (r = 0.831, Pearson Product Moment). VEGF production by MDA-MB231 cells is significantly upregulated following treatment with IL-6 for 24 hours ($p \leq 0.02$).
**Conclusions:** Interleukin-6 increases both tumour cell proliferation and vascular endothelial cell growth factor production in breast cancer.

### P94 THE ROLE OF PDGF IN TUMOUR CELL-DERIVED FACTOR STIMULATION OF FIBROBLAST GLYCOSAMINOGLYCAN SYNTHESIS,

J.L. Godden , M. Edward* and R.M. MacKie, Department of Dermatology, The Robertson Building, University of Glasgow, Glasgow G12 8QQ.

Many tumours are surrounded by a hyaluronan-rich matrix which is thought to facilitate tumour growth, angiogenesis and invasion. The majority of this hyaluronan appears to be synthesised by normal fibroblasts stimulated to do so by tumour cell-derived factors. We have previously shown that conditioned medium (CM) from two human melanoma cell lines (C8161 & Hs294T) contains potent fibroblast glycosaminoglycan (GAG)-stimulating factors. Amicon ultrafiltration of the tumour CM suggests that full activity is dependent upon synergy between a factor present in a fraction >1kDa and a factor <1kDa, with the individual fractions exhibiting minimal activity.

The >1kDa fraction exhibits a degree of binding to heparin-Sepharose and partial heat and trypsin sensitivity. In view of these observations, a number of growth factors were examined for their activity as stimulators of GAG synthesis. In isolation none of the tested factors including bFGF, IL-1β, pleiotrophin, PDGF, TGF-β, TNF-α and VEGF displayed any significant GAG-stimulating activity, but when tested in the presence of the <1kDa fraction, bFGF and PDGF were both found to stimulate GAG synthesis. The presence of PDGF and bFGF in the melanoma cell CM was verified using commercial ELISA kits. Hs294T cell CM contained 1.7ng/ml PDGF-AB and 160pg/ml bFGF and C8161 cell CM 1.6ng/ml PDGF-AB and 12pg/ml bFGF. Neutralising antibodies to bFGF and PDGF added to the melanoma cell CM decreased the fibroblast GAG-stimulating activity in both cases.

As PDGF stimulated the greatest increase in fibroblast GAG synthesis, we examined fibroblast phosphotyrosine response to PDGF and melanoma cell CM. The activity of PDGF-AA and PDGF-BB isoforms was found to be indistinguishable, suggesting the PDGF-α receptor plays a role in the GAG stimulatory response since the PDGF-β receptor will bind only to the PDGF-BB isoform of the growth factor. Western analysis after treatment with PDGF, bFGF or melanoma cell CM revealed banding patterns for PDGF and melanoma cell CM that were similar. Immunoprecipitation of PDGF receptor α revealed it to be phosphorylated in fibroblast cells treated with PDGF at 10ng/ml and melanoma cell CM, but cells treated with control fibroblast CM showed no PDGF receptor α phosphorylation. These studies suggest that PDGF plays an important role in the GAG-stimulating activity of the CM by synergising with the low molecular weight fraction (<1kDa).

### P95 TUMOUR VASCULAR RESPONSE TO THE ENDOTHELIN B RECEPTOR AGONIST IRL 1620: SPATIAL DISTRIBUTION OF BLOOD FLOW MODIFICATION.

K. M. Bell*[1], G. M. Tozer, B. A. Poole, V. E. Prise & D.J. Chaplin, [1]CRC Tumour Microcirculation Group, Gray Laboratory Cancer Research Trust, PO Box 100, Mount Vernon Hospital, Northwood, Middlesex, HA6 2JR.

Endothelin-1 (ET-1) is a potent vasoconstrictor produced by endothelial cells, which acts via two groups of receptors namely $ET_A$ and $ET_B$ receptors. In the vasculature, $ET_B$ receptors reside on both the smooth muscle cells, which produces vasoconstriction, and the endothelial cells, which causes vasodilation. The result of the balance between the constrictive and dilator responses to $ET_B$ receptor ligands is dependent on the complement of receptor subtypes within the tissue. Tumours are unresponsive to many vasoactive agents, therefore we compared the response of the rat HSN tumour with that in normal rat tissues, to the selective endothelin B ($ET_B$) receptor agonist, IRL 1620 ([Suc-(Glu$^9$, Ala$^{11,15}$)-ET-1 (8-21)]).

IRL 1620 was administered to anaesthetised rats by bolus i.v. injection at a dose of 1, 3 or 5nmol kg$^{-1}$. Blood flow in the tumour and normal tissues was measured 20 min later, by the uptake of $^{125}$I iodoantipyrine ($^{125}$I-IAP). Blood flow in the tumour decreased dose dependently to 0.4 of the control blood flow, in contrast to the majority of tissues in which blood flow was unchanged. The exceptions to this were the brain and heart in which blood flow increased by a factor of 1.2-1.5 at all doses, and the small intestine in which blood flow was also reduced to 0.5 of the control value, at all doses tested.

Changes in the spatial distribution of tumour blood flow were examined in a group of rats treated with 3nmol kg$^{-1}$ IRL 1620 (bolus i.v.), compared to that in saline treated animals. Blood flow was measured by the uptake of $^{14}$C-IAP and spatial distribution of blood flow measured by autoradiography. Blood flow in the tumour periphery was higher than that in the central region of the tumour (0.76±0.10 *versus* 0.49±0.09 ml•g$^{-1}$•min$^{-1}$). In rats treated with IRL 1620 however, a reduction in blood flow to approximately 0.4 of control blood flow was observed in both the peripheral and central regions of the tumour.

Results illustrate that the HSN tumour responds relatively selectively to the $ET_B$ receptor agonist, IRL 1620, and that vessels throughout the tumour are uniformly responsive. Such a finding may be exploitable in cancer therapies which are enhanced under ischaemic conditions, such as the use of bioreductive drugs.

*This work was supported by funding from the AICR and the Cancer Research Campaign.*

### P96 COMBRETASTATIN A-4 P AS A TUMOUR VASCULAR TARGETING AGENT,

G. M. Tozer[1], V. E. Prise[1], J. Wilson[1], B. Vojnovic[1], S. Q. Shan[2], M. W. Dewhirst[2] and D. J. Chaplin[1], [1]Microcirculation Group, Gray Laboratory Cancer Research Trust, P. O. Box 100, Mount Vernon Hospital, Northwood, Middlesex, HA6 2JR, [2]Department of Radiation Oncology, Duke University Medical Center, Durham, NC 27710, USA.

The tubulin-binding agent, combretastatin A4 and its more soluble derivative, combretastatin A4-P (CA4-P), have potential as tumour vascular targeting agents. CA4-P caused vascular collapse in murine and human tumours growing in mice at non-toxic doses (Dark *et al.*, 1997, Cancer Research, 57, 1829). In the present study, the vascular effects of CA4-P were investigated in more detail in rats bearing the P22 rat carcinosarcoma. Blood flow to s.c. tumours and normal tissues was measured using the uptake of $^{125}$I-labelled iodo-antipyrine ($^{125}$I-IAP). Rats bearing the P22 tumour growing in transparent window chambers implanted into the dorsal skin were used to measure tumour vascular parameters at the single vessel level.

Tumour blood flow was reduced to 12% and 0.9% of control levels at 1h and 6h respectively following 100 mg • kg$^{-1}$ CA4-P i.p.. Significant but smaller reductions in blood flow were also observed in normal skin, skeletal muscle and spleen at 1h. No significant reductions were found for brain, heart, kidney and small intestine at 1h. At 6h, blood flow to the brain had decreased to 80% of control levels with no significant change in heart, kidney and small intestine.

The gross tumour vascular changes induced by 100 mg • kg$^{-1}$ CA4-P i.p. were characterised by irregular / intermittent flow, sludging of red cells and, finally, stasis. At 10 minutes following drug administration, 35% of venules/capillaries in central tumour areas were static. At 75-100 minutes, 70% of venules / capillaries were static. Early changes included a decrease in red cell velocity to 40% of control at 2 minutes and a decrease in vascular diameter to 60% of control at 10 minutes after drug administration, in tumour arterioles. Infusion of Hoechst 33342 into the systemic circulation of a subset of animals highlighted a population of endothelialised venules and a population of venules devoid of endothelial cells. Preliminary studies have shown that the endothelialised vessels were more resistant to CA4-P than the non-endothelialised vessels.

In summary, IAP uptake studies showed that the vascular effects of CA4-P were more profound in tumours than in any of the normal tissues studied. However, significant blood flow changes were observed in normal tissues, highlighting the need for further toxicity studies. The window chamber studies suggest that the mechanism of action of CA4-P may involve vasoactive effects and blood coagulation which is influenced by local factors such as the action of endothelial cells. CA4-P is a promising vascular targeting agent which requires further study in order to determine its mechanism of action and selectivity for tumour tissue.

*This work was supported by the Cancer Research Campaign*

### P97 BCL-2, PLATELET-DERIVED ENDOTHELIAL CELL GROWTH FACTOR (PD-ECGF), VASCULAR ENDOTHELIAL GROWTH FACTOR (VEGF), AND ANGIOGENESIS IN CERVICAL INTRAEPITHELIAL NEOPLASIA (CIN) AND SQUAMOUS CELL CARCINOMA (SCC).

SP Dobbs[1], LJR Brown[1], D Ireland[1], K Gatter[2], AL Harris[2], K Abrams[1], W Steward[1], KJ O'Byrne*[1], Tumour & Angiogenesis Research Group, [1]Leicester Royal Infirmary and [2]Oxford Radcliffe Hospitals, UK.

Angiogenesis is essential for the growth and metastatic spread of solid tumours. Recent work has demonstrated that angiogenesis increases with histological grade of CIN, the precursor to SCC. VEGF and PD-ECGF are angiogenic growth factors for a number of solid tumours including lung, bladder and colorectal carcinoma. Expression of bcl-2, an inhibitor of apoptosisis, is associated with a favourable outcome in solid tumours and inversely correlates with vascular grade in squamous cell lung cancer. We evaluated VEGF, PD-ECGF and bcl-2 expression and microvessel density (MVD) in paraffin-embedded tissue sections of normal cervix, CIN and SCC from 60 patients using immunohistochemistry. MVD was calculated by counting the number of von Willibrand's factor (vWf) positive vessels per unit area subtending normal and abnormal epithelium. Expression of VEGF, PD-ECGF and bcl-2 was assessed visually and scored from 0 - 3. A significant progressive increase in MVD from a mean of 30 for normal tissue to 56 for invasive tissue was seen (p=0.0001). The expression of VEGF (p=0.004) and PD-ECGF (p=0.05) likewise increased from normal cervical tissue through to malignant disease. There was a progressive loss of bcl-2 expression, the highest expression being seen in normal tissue and the lowest in SCC (p=0.05). In conclusion, a progressive increase in MVD, and VEGF and PD-ECGF expression, is seen as the cervix takes on a malignant phenotype indicating an important role for angiogenesis and angiogenic growth factors in the pathogenesis of SCC. The development of anti-angiogenic agents and VEGF antagonists, and the recognition that PD-ECGF is thymidine phosphorylase, a key enzyme in the activation of fluoropyrimidine cytotoxic agents suggests that the immunohistochemical evaluation of MVD, VEGF and PD-ECGF should be taken into account in designing chemotherapeutic studies in cervical cancer.

### P98 ABL EXPRESSION IN LIPOSARCOMAS WITH PARTICULAR REFERENCE TO ANGIOGENIC TUMOUR MICROVESSELS

JM Russell[1], AJ O'Neill[2], BM Dunne, M O'Donovan, CDM Fletcher[3], TG Cotter[4], M Lawler, EF Gaffney.

St. James's Hospital, Brigham and Women's Hospital[3] and U.C.C.[4]

Abl kinases encoded by the abl oncogenes play an important role in cell cycle regulation and inhibition of apoptosis. Our previous immunohistochemical study demonstrated that the Abl protein is weakly or focally expressed in many different normal tissues, and that certain cell types (chondrocytes and adipocytes) showed consistent strong staining. In this study, we extended our observations on Abl expression to a series of liposarcomas. Sections from 28 paraffin blocks of liposarcoma (round cell, myxoid, mixed type and well differentiated) were stained immunohistochemically for the c-Abl/BCR-Abl oncoprotein (Serotec, UK), using appropriate controls.

Expression of the Abl oncoprotein was primarily related to tumour cell differentiation. The mature adipocytes of the well differentiated tumours demonstrated strong Abl expression, but round cells and preadipocytes of round cell and myxoid liposarcomas were negative. The angiogenic tumour microvessels showed variable Abl staining, most marked in myxoid liposarcomas.

These findings strongly support our previously reported role for the c-abl oncogene in angiogenesis in addition to playing an important role in tumour differentiation. In on-going studies we are confirming the validity of these observations by determining expression of c-abl mRNA using RT-PCR and IS-RT-PCR.

[1]Supported by the Health Research Board and [2]by IACR and CRAB.

### P99 EXPRESSION OF THE ABL ONCOPROTEIN IN THE NEOVASCULATURE DURING ENCHONDRAL OSSIFICATION AND IN TUMOUR ANGIOGENESIS.

JM Russell[1], AJ O'Neill[2], BM Dunne[3], M O'Donovan[3], JE Gillan[4], TG Cotter[5], M Lawler[3], EF Gaffney[3]. St. James's Hospital[1,2,3], Rotunda Hospital[4] and U.C.C.[5]

The abl oncogenes, encoding abl protein tyrosine kinases, inhibit apoptosis without affecting cell proliferation. Abl is ubiquitously expressed in most mammalian tissues, but intense staining is observed in certain cell types such as chondrocytes and adipocytes.

The aim of this study was to investigate Abl expression in angiogenesis during enchondral ossification and in tumour angiogenesis. Sections from 24 paraffin blocks of fetal rib (16-42wks gestation), 8 signet ring carcinomas of stomach, 8 liposarcomas and 10 breast carcinomas were stained immunohistochemically for the c-Abl/BCR-Abl oncoprotein (Serotec, UK), using appropriate controls.

Abl immunoreactivity was not seen in normal blood vessels in adult tissues. In the fetus, there was striking Abl immunoreactivity in osteoblasts and their associated neovasculature in sites of enchondral ossification from 17 weeks. In signet ring carcinoma, breast carcinoma and liposarcoma moderate to intense Abl expression was observed, to a variable extent, in angiogenic tumour microvessels as well as in tumour cells. These findings highlight a previously unidentified role for the c-abl oncogene in angiogenesis. The validity of these observations is currently being demonstrated at the mRNA level using RT-PCR and IS-RT-PCR.

[1]Supported by the Health Research Board and [2]by IACR and CRAB.

### P100 HISTOGENESIS OF ENDOTHELIAL CELLS OF CEREBELLAR HAEMANGIOBLASTOMA

N Swan*[1] N Mulligan[1], S Nikulasson[1], AO Vortemeyer[2] and F Fogt[3], [1] Mallory Institute of Pathology, 784 Mass. Avenue, Boston MA 02118, [2] Laboratory of Pathology, National Cancer Institute, Bethesda, Maryland, and [3] Department of Pathology, University of Pennsylvania, Philadelphia, PA., USA.

**Aims**: Cerebellar haemangioblastoma is a tumour characterized by proliferation of both endothelial and stromal cells. Recent evidence demonstrating that loss of heterozygosity of the VHL gene was restricted to the stromal cells of sporadic haemangioblastomas suggests that the stromal element is neoplastic and that the vascular proliferation is reactive and non-neoplastic[1]. The exact histogenesis of these tumours, however is uncertain. The aim of this study was to further investigate the origin of the vascular component using Glucose transporter protein 1(GLUT-1), a component of the blood-brain barrier which is used as a marker for the endothelium of cerebral microvasculature.

**Methods:** Using an antibody which recognizes GLUT-1, we stained paraffin sections from 5 cases of cerebellar haemangioblastoma. Staining of the vascular component was assessed by examining 20 high power fields from each case. Areas with adjacent normal brain tissue were included as a positive internal control.

**Results:** We found that at least 80% of the vascular component in each case did not stain for GLUT-1. The vasculature of the adjacent normal brain tissue showed strong positive staining in all 5 cases.

**Conclusions:** We conclude that most endothelial cells of cerebellar haemangioblastomas do not express GLUT-1. It is likely therefore that they do not originate from the brain and may in fact be derived from extra-cerebral microvasculature, most likely dura. This finding has significant implications for the histogenesis of these rare tumours.

**Reference:**

1. Loss of heterozygosity and somatic mutations on the VHL tumor suppressor gene in sporadic cerebellar hemangioblastomas. Lee JY, Dong SM, Park WS et al. 1998 *Cancer Res*:58;504-8.

### P101

TO DETERMINE THE EFFECT OF PRIMARY TAMOXIFEN TREATMENT ON THE VASCULARITY OF BREAST CANCERS. L. P. Marson*[1], K. Kurian[2], W. R. Miller[1], J. M. Dixon[1]. [1]Edinburgh Breast Unit, [2]Department of Pathology, Western General Hospital, Edinburgh EH4 2XU.

**Introduction**: New vessel formation is essential for tumour progression and for metastasis [1]. There is evidence to suggest that oestrogen plays a role in the preservation of endothelial cells by the inhibition of apoptosis [2]. The aim of this study is to determine whether treatment of primary breast cancers with neoadjuvant tamoxifen is associated with a change in tumour vascularity.
**Methods**: Thirty one postmenopausal women with large (>3cm), oestrogen receptor positive (>20fmol/mg cytosolic protein) primary breast carcinomas were treated with a three month course of tamoxifen, 20mg daily, prior to surgery and following an initial wedge biopsy. Response to tamoxifen was assessed by serial monthly ultrasound of the primary tumour and was defined as >25% reduction in tumour volume with no evidence of progression in the final ultrasound assessment. Pre and post-treatment tumour sections were stained with the endothelial marker, antibody to FVIII (DAKO). Microvessel counts were performed by two observers, in high power fields (x250) of three vascular hot spots using a Chalkley 25 eyepiece graticule. The microvessel count (mvc) was the total of the counts in the three fields. This study design allowed comparison of microvessel counts before and after treatment, and correlation of this change with tumour response.
**Results**: The median value of microvessel counts for all patients before and after treatment was 13. Of the 31 patients, 24 responded (77%). The pre-treatment mvc in the responders was 15 (Range: 8-26), compared to 12 (Range: 9-13) in the 7 non-responders (p=0.0389: Wilcoxon test). There was no significant difference between the two groups in the post-treatment mvc. The change in mvc with treatment according to response is illustrated in the table:

| | Increased mvc | Decreased mvc |
|---|---|---|
| **Responders** | 7 | 17 |
| **Non-responders** | 7 | 0 |

**p=0.0013** (Fisher's exact test)

There is a highly significant association between change in tumour vascularity and response to tamoxifen treatment. All non-responders showed an increase in tumour vascularity and all those tumours in whom the microvessel count decreased responded.

1. Folkman J. Angiogenesis and Breast Cancer . *Journal of Clinical Oncology* 1994;12(3):441-443.
2. Sypridopoulos I, Sullivan AB, Kearney M, Isner JM, Losordo DW. Estrogen-receptor-mediated Inhibition of Human Endothelial cell Apoptosis. *Circulation* 1997;95(6):1505-1514.

### P102

EFFECT OF RETINOIC ACID AND PENTOXIFYLLINE ON HUMAN MELANOMA CELL EXPRESSION OF uPA AND tPA. C.L.Alexander, M. Edward* and R.M. MacKie, Department of Dermatology, The Robertson Building, University of Glasgow, Glasgow G12 8QQ.

The plasminogen activation system plays an important role in the metastatic spread of tumour cells and includes various proteases such as tissue-type (tPA) and urokinase-type (uPA) plasminogen activators. These enzymes convert plasminogen to plasmin, a broad specificity enzyme which degrades a range of extracellular matrix molecules thus contributing to the invasive potential of the tumour cell. In this study, we have examined the effect of retinoic acid (RA) and pentoxifylline (PX) on uPA and tPA secretion by the Hs294T and C8161 melanoma cell lines using a specific ELISA detection system.

We have previously shown that the Hs294T cells are poorly metastatic in vivo in comparison to the aggressive C8161 cell line. uPA is known to bind to a uPA receptor (uPAR) on the cell surface and we were able to detect membrane-associated uPA in both cell lines using confocal microscopy. The highly aggressive C8161 cell line secreted a 277-fold higher level of uPA (1300fg/cell) than the Hs294T cells (4.7fg/cell). We examined the effect of exposure of the cells to retinoic acid ($10^{-10}$ - $10^{-5}$ M) for 4 days on both uPA and tPA production. At $10^{-7}$M RA, uPA levels were increased 1.2-fold in the Hs294T cells and by 2.2-fold in the C8161 cells. PX treatment (250µg/ml) also increased uPA production 2.7-fold in the Hs294T cells and by 1.7-fold in the C8161 cells. The Hs294T cells which secreted lower levels of uPA were found to produce higher levels of tPA (25.6fg/cell) compared to the C8161 cells (4.3fg/cell). RA stimulated a 2.5-fold increase in tPA at $10^{-7}$M and a 3.7-fold increase at $10^{-5}$M in the Hs294T cells.

These results demonstrate that both PX and RA increase uPA production, while only PX increases tPA expression in these cell lines.

### P103

THERAPEUTIC EFFECT OF THE MATRIX METALLOPROTEINASE INHIBITOR, MARIMASTAT, IN A GASTRIC CANCER XENOGRAFT MODEL: RELATIONSHIP TO CEA LEVELS.*S.A. Watson[1], T.M. Morris[1], H.M. Collins[1], G Tierney[1], L.J. Bawden[2], K. Hawkins[2], P.D. Brown and E.A. Bone[2]
[1]Academic Unit of Cancer Studies, Division of GI Surgery, University of Nottingham UK. [2]British Biotech Pharmaceuticals Ltd. Oxford, UK.

Matrix metalloproteinase (MMP) inhibitors are under investigation as potential therapeutic modalities for treatment of invasive growth and metastasis of many tumour types. The aim of these studies was to evaluate the effect of the broad spectrum MMP inhibitor, marimastat, on the growth of a CEA-secreting human gastric xenograft, MGLVA1, allowing any relationship between therapeutic effect and serum CEA levels to be determined.
MGLVA1 was shown by RT-PCR to express mRNA for MT-MMP1, MMP-7 and MMP-9. Zymography revealed the presence of MMP-9 activity as well as the inactive and active forms of MMP-2. For the therapy experiments MGLVA1 tissue was implanted subcutaneously into both male and female nude mice. Dosing with marimastat (15mg/ml is osmotic mini-pump, equivalent to approximately 7.2mg/kg/day) began on day 1 and continued throughout the course of the experiment. Control mice were implanted with vehicle-containing pumps. Cross sectional tumour measurements were taken twice weekly using callipers, and animals were terminated when tumour cross sectional area reached 300mm$^2$. All experimentation was performed according to UKCCCR guidelines.
Marimastat was shown to significantly inhibit tumour size in both male and female mice when compared with the respective vehicle controls (p=0.0081, p=0.0306, respectively on day 17, Mann Whitney U non-parametric test). Marimastat also exerted a significant effect on survival (p=0.001, Log Rank test) with median survival increasing from 18 days to 30 days.
A further experiment was designed to assess the effect of marimastat on circulating CEA levels. Marimastat or vehicle was delivered as above, and the ability of marimastat to significantly inhibit tumour growth was confirmed. Throughout the course of the experiment 4 animals of each sex from both treated and control groups were sacrificed at regular intervals and serum samples were collected for CEA analysis. It has been shown that log of the CEA concentration is linearly related to log of the tumour weight, irrespective of whether the tumour derives from a marimastat or vehicle treated animal.
These results suggest that circulating CEA concentration may be useful as a surrogate for tumour burden.

### P104

**THERAPEUTIC EFFECT OF THE MATRIX METALLOPROTEINASE INHIBITOR, MARIMASTAT IN A GASTRIC CANCER XENOGRAFT MODEL: RELATIONSHIP TO MMP mRNA LEVELS.**

***GM Tierney, HM Collins, TM Morris, SA Watson.**
**Academic Unit of Cancer Studies, Division of GI Surgery, University of Nottingham, UK.**

**Background:** The MMPs are a family of proteolytic enzymes which regulate turnover of the extracellular matrix and are implicated in the process of tumour growth and metastasis. Their inhibition is a novel therapeutic modality.
Marimastat is a synthetic orally available MMP inhibitor which binds to the active site of the MMP molecule rendering it inactive.
**Aims**: To evaluate the effect of marimastat on the growth and MMP expression of human gastric xenografts, MKN45G and ST16 and to relate any observed effect to a change in MMP mRNA level.
**Methods**: MKN45G and ST-16 tissue was implanted sub-cutaneously into both male and female nude mice. Dosing with marimastat (50mg/kg administered via osmotic mini-pumps) began on day 1 and continued for 28 days. Control mice were implanted with vehicle containing pumps. Animals were terminated on day 28. All animal experimentation was performed according to UKCCCR guidelines. Xenograft tissue was taken immediately post termination, mRNA extracted and reverse transcribed to cDNA. Competitive PCR was performed with a gene specific external standard. Results were normalised to GAPDH.
**Results:** Mean tumour mass control vs treatment (MKN45G - 0.13g vs 0.2g = ns, ST-16 0.15g vs 0.01g p<0.05) was recorded. ST-16 tumours were not macroscopically detectable post- marimastat therapy. RT-PCR demonstrated mRNAs for MMPs-2, -7 and -9, TIMPs-1 and -2 and MT-MMP-1 in all xenograft control samples. MKN45G showed a significant reduction in mRNA for MT-MMP-1 post treatment (p<0.05 Mann-Whitney).
**Discussion**: Marimastat administration has caused ST-16 xenografts to become macroscopically undetectable. mRNA analysis suggests that in addition to blocking the active site of the MMP molecule marimastat may exert an indirect effect at the mRNA level. A significant down-regulation in MT-MMP-1 mRNA has been noted. This enzyme is a potent activator of MMP-2 and as such may play a pivotal role in the postulated activation cascade of MMPs. This may represent a feedback loop resulting from blockade of active enzymes by marimastat.

### P105 TREATMENT WITH MARIMASTAT DOES NOT AFFECT THE GELATINASE PROFILE SEEN WITH ZYMOGRAPHY.

**Tierney GM[1], Kumar A*[1], Collins HM[1], Parsons S[1], Kasem H[2], Stuart R[2], Steele RJC[3], Watson SA[1].**
**[1]Academic Unit of Cancer studies, Division of GI Surgery, University of Nottingham, UK. [2] University Department of Surgery, Glasgow Royal Infirmary, Glasgow, UK. [3] Department of Surgery, Ninewells Hospital, Dundee, UK.**

Background: Matrix metalloproteinases are a family of proteolytic enzymes involved in turnover of the extracellular matrix and have been implicated in the process of tumour growth and metastases. In particular the gelatinases have been shown to correlate with the malignant phenotype. Zymography is an electrophoretic technique which distinguishes active gelatinase from latent pro-enzyme. The matrix metalloproteinase inhibitor, marimastat BB-2516 (British Biotech Ltd.) has been developed for clinical use in a multicentre phase II trial in patients with inoperable gastric carcinoma. Endoscopic biopsies of tumour from these patients before and after one month of treatment have been obtained and analysed using zymography.

Aims: To examine the profile of latent and active gelatinases present in gastric cancer samples prior to and after one month of treatment with marimastat.

Methods: Endoscopic biopsies of gastric cancer from 20 trial patients were homogenised in denaturing buffer, centrifuged and loaded onto polyacrylamide/gelatin gels. Electrophoresis was performed. The gel was incubated with renaturing buffer and developing buffer to reactivate the enzyme. The gel was stained with Coomassie blue, destained and dried. Gels were then analysed using a computer assisted image analysis system. Purified human recombinant 72kDa gelatinase and 92kDa gelatinase markers were used as standards.

Results: The 92kda and the 72kDa gelatinases were expressed in the tumour biopsies both prior to and after treatment with the matrix metalloproteinase inhibitor. Their active forms (82kDa and 62kDa) were also identified on the gels. After treatment there was no significant change in the quantity of active or inactive enzyme.

Conclusions: Treatment with a matrix metalloproteinase inhibitor for one month does not affect the enzyme profile of a gastric cancer as shown on zymography. The denaturing conditions of zymography cause separation of the inhibitor-enzyme complexes. Further work using RT-PCR is planned to assess changes at the level of mRNA.

### P106 ENHANCEMENT OF ADEPT WITH ANTI-VASCULATURE DRUGS IN A XENOGRAFT MODEL.

*R. B. Pedley[1], S.K. Sharma[1], R. Boden[1], G.M. Boxer[1], C.J. Springer[2] & R.H.J. Begent[1]. [1]Cancer Research Campaign Targeting and Imaging Group, Dept. Clinical Oncology, Royal Free & UCL Medical Schools, London NW3 2PF. [2]CRC Centre for Cancer Therapeutics, ICR, Sutton, Surrey SM2 5NG.

Antibody-targeted therapy of cancer has significantly improved selective delivery of antitumour agents, but clinical problems remain in the form of poor tumour localisation and toxicity to normal tissues. Specific destruction of tumour vasculature has greater therapeutic potential than direct tumour cell targeting, but present strategies frequently fail to destroy all functional vessels. Efficient treatment of common solid tumours may therefore require a combination of these different but complementary therapies. We are employing two concomitant therapies: the anti-vasculature drug 5,6-dimethylxanthenone-4-acetic acid (DMXAA) which inhibits blood flow causing extensive necrosis of the central tumour zone, and anti-tumour antibodies conjugated to therapeutics which can destroy the surviving outer zone. We have previously shown that an optimal dose of DMXAA (27.5mg/kg) did not enhance survival. However, by combining DMXAA with an antibody-radioisotope conjugate (18.5MBq $^{131}I$ anti-CEA antibody A5B7), which alone inhibits tumour growth by around 30 days, we have eradicated tumours in 85% of mice [1].

We are currently investigating whether the combined use of DMXAA with antibody-directed enzyme prodrug therapy (ADEPT) [2] will also enhance therapeutic potential. In ADEPT therapy a $F(ab')_2$ A5B7-enzyme (carboxypeptidase G2) conjugate is administered at 25 enzyme units/mouse and allowed to localise to the tumour and clear from normal tissues. The second phase, the non-toxic prodrug 4-[(2-chloroethyl)(2-mesyloxyethyl)amino]benzoyl-L-glutamic acid (CMDA), is then delivered at 1.5g/kg and converted to a toxic drug by enzyme within the tumour. It would be therapeutically advantageous to retain either antibody conjugate or CMDA at the tumour site, and we have found that the massive haemorrhagic necrosis produced by DMXAA gave a 2-fold increase in tumour retention of pre-localised conjugate and altered its microdistribution. This novel complementary treatment also significantly enhanced the therapeutic effect of conventional ADEPT, either by allowing greater prodrug activation, by DMXAA reduction of the tumour burden, or by a combination of the two.

1. Pedley et al., Cancer Res. 56: 3293-3300, 1996.
2. Sharma et al., Dis. Markers 9: 225-231, 1991.

Supported by the Cancer Research Campaign.

### P107 ROLE OF p53 ON THE CYTOSTATIC *VERSUS* CYTOTOXIC EFFECTS OF UNSATURATED FATTY ACIDS ON NORMAL HUMAN UROTHELIAL CELLS *IN VITRO*

C.P. Diggle*, E. Pitt, L.K. Trejdosiewicz & J. Southgate. ICRF Cancer Medicine Research Unit, St James's University Hospital, Leeds LS9 7TF, UK.

Polyunsaturated fatty acids (PuFA) can inhibit the growth of carcinoma cells *in vitro*. Normal human urothelial (NHU) cells, which prolliferate rapidly *in vitro*, are also growth inhibited by 72h culture in the presence of n-3 and n-6 PuFA, whereas monounsaturated and saturated fatty acids had no effect (Southgate *et al. Br. J. Cancer 1997;* **74:** 728). Although PuFA can induce an apparently irreversible growth arrest at higher concentrations, it is not clear whether there is a cytotoxic effect distinct from the cytostatic mechanism operative at lower PuFA concentrations. p53 is involved cell cycle regulation and cell death, although whether PuFA operate through p53 is unknown.

NHU cell lines were transfected with the HPV16 E6 gene to disable p53 function. The effects of short (72h) and long-term exposure to BSA-complexed n-3 (eicosapentaenoic) and n-6 (linoleic and γ-linolenic) PuFA on NHU and homologous NHU-p53$^{NULL}$ cells was investigated. Cells were cultured in various PuFA concentrations to identify the maximal tolerated dose allowing survival for the normal lifespan of the culture.

NHU cells cultured continuously with PuFA showed a reduced growth rate relative to BSA-only controls. The maximal concentration of γ-linolenic acid tolerated in prolonged culture was 10μM, a non-cytostatic dose in short-term culture. The maximal sustainable dose for linoleic acid was 40μM, which decreased thymidine incorporation by some 60-70% in short-term culture. Cells in eicosapentaenoic acid could sustain a dose of 20μM, which inhibited thymidine incorporation by 50% in short-term cultures. The p53$^{NULL}$ cells showed the same response patterns to PuFA as untransfected cells.

Thus, the cytostatic and cytotoxic effects of PuFA on normal human urothelial cells are distinct phenomena, probably acting through discrete mechanisms, neither of which involves p53. The two events operate at differing concentrations, which imply that different compositions and amounts in the diet may be significant.

### P108 CORRELATION OF RETINOIC ACID RECEPTOR-B EXPRESSION IN ORAL DYSPLASIAS WITH A CHANGE FROM THE SENESCENT TO IMMORTAL CELL PHENOTYPE.

F. McGregor*[1], E. Wagner[1], D. Felix[2], D. Soutar[3], K. Parkinson[1] and P.R. Harrison[1], [1]The Beatson Institute of Cancer Research, CRC Beatson Labs, Glasgow G61 1BD, [2]Dept of Oral Medicine, Dental School, Glasgow G2 3JZ.[3]Dept. Of Plastic Surgery, Canniesburn Hospital, Glasgow G61 1QL,

Retinoids, naturally occurring and synthetic derivatives of vitamin A, have been found to have potent anti-tumour activity in a number of clinical contexts, including the treatment of pre-malignant oral lesions and prevention of the occurrence of second primary cancers after resection of the primary tumour. However, long-term prognosis is still poor, presumably due to malignant cells escaping retinoid control. Understanding the mechanisms by which retinoids and their receptors interact with cellular the regulatory pathway and prevent malignant progression, is an important aspect of the development of novel treatment and preventive strategies.

We have developed a unique panel of human primary oral cell cultures, representative of the various stages of tumour progression ie. normal healthy oral tissue, a range of early dysplasias, late stage tumours and metastasis. Using these cultures has allowed us to look at differences in expression of the retinoid receptors and cellular binding proteins, responsible for mediating the biological effects of the retinoids, at different stages of progression. Northern analysis showed that there were no consistent changes during oral cancer progression of the levels of the nuclear receptors, retinoic acid receptors α and γ (RARs), and retinoid X receptor-β (RXR) and cellular binding proteins (CRBP and CRABP-II) expressed in these cells. Previous work has shown that loss of RAR-β is one of the most consistent changes occurring during oral cancer progression which is confirmed in this system. In addition we have demonstrated that RAR-β loss occurs early in progression and that this loss is associated with a change in cell phenotype from senscence to immortality during the dysplasia stage (McGregor et al., 1998, Cancer Research 57, p3886). In addition this change in phenotype may occur independently of the loss of CDKN2A/p16 expression. This work is funded by the A.I. C.R (uk) and the CRC.

### P109 *IN VIVO* EVALUATION OF A LIPOLYTIC MOBILISING (LMF) FACTOR ISOLATED FROM PATIENT URINE.

Hussey H J* and Tisdale M J, Pharmaceutical Sciences Institute, Aston University, Birmingham B4 7ET.

A lipid mobilising factor (LMF) was isolated from the urine of cancer patients with progressive weight loss, using a combination of ion exchange, exclusion and hydrophobic chromatography to give a single component of apparent molecular weight 43,000. Biological activity throughout purification was monitored by the ability to induce lipolysis in epididymal adipocytes.

Administration of isolated LMF to ex-breeder NMRI mice over a 89h period produced a decrease in body weight without a reduction in either food or water intake. Body composition analysis showed a 42% reduction in carcass fat in comparison to controls. Treatment of pure bred obese (ob/ob) mice with human LMF over a 160h period also produced a decrease in body weight with no effect on either food or water consumption. There was a reduction of 19% in carcass fat without an alteration in water or non-fat mass. Hypoglycaemia was observed, but was not accompanied by a change in serum insulin levels. There was a significant increase in both glycerol ($p<0.02$) and 3-hydroxybutyrate ($p= <0.001$) in the serum, suggesting lipid mobilisation and utilisation. Though treatment with LMF reduced the content of interscapular brown adipose tissue in ob/ob, oxygen utilisation per gram of tissue greatly increased ($p= <0.009$). Oxygen uptake in brown adipose tissue was increased by noradrenaline, an action that was reversed by propranolol. LMF also increased oxygen uptake in brown adipose tissue, but the action was not reversed by propranolol.

These results show that a LMF isolated from human urine has the capacity to induce lipid mobilisation and catabolism in mice, suggesting the potential to exert similar effects in cachectic cancer patients.

### P110 ABNORMALITIES OF LIPID METABOLISM IN STARVED AND VX2-TUMOUR BEARING RABBITS,

K.Hirai*, O.Ishiko, T.Yasui, K.Honda, T.Sumi, S.Nishimura and S.Ogita, Dept. of Ob&Gyn, Osaka City University, Osaka 545-8585 Japan

Patients with advanced malignant disease display appetite loss and weight loss known as cancer cachexia. In this study, we used cachectic rabbits transplanted with VX2 carcinoma to determine the abnormality of body fat store and lipid metabolism in comparison with starved rabbits where a simple reduction in food intake causes loss of body fat. In addition, the effect of plsama fractions from rabbits with VX2 carcinoma or from starved rabbits were examined. After chromatography of plasma from rabbits with VX2 carcinoma on a phenyl-Sepharose column, a portion of a fraction was added to the culture medium of murine 3T3-L1 adipocytes, and the amount of glycerol released into the medium was measured as an index of lipolysis. To other cultures, a plasma fraction obtained in the same way from rabbits without tumours and fed as usual (controls), or from such rabbits with feed withheld, was added. Glycerol release into medium from 3T3-L1 cells treated with the plasma fraction of cachectic rabbits or with the same fraction from starved and healthy control rabbits were 0.370, 0.265 and 0.288 $\mu mol/10^5$ cells in 8hr, respectively. Intracellular levels of triglycerides for each group was 0.294, 0.316 and 0.340 $\mu mol/10^5$ cells, respectively. On the other hand, a piece of adipose tissue was obtained from three of these groups of rabbits (with cachexia, starved, or fed as usual) and the incorporation of radiolabelled glucose into glycerides by the tissue was measured. Incorporation of radiolabelled glucose into adipose tissue from cachectic, starved, and control rabbits were 15.9, 46.8 and $18.0\times10^5$ cpm/g per hr, respectively. Glycerol release from the materials were 511, 365, and 201 nmol/g per hr. These data suggests that pathophysiology characterized to lipid metabolism in cancer cachexia includes accelerated lipolysis and reduced lipogenesis which were different from those in starved rabbits, and some humoral factor might be involved in this abnormality.

### P111 INCREASED PROLIFERATION OF MYOBLASTS AFTER CYCLIC PLASMA PERFUSION OF TUMOUR-BEARING RABBITS,

O.Ishiko*, K.Hirai, S.Nakata, K.Honda, M.Deguchi, H.Yoshida and S.Ogita, Dept. of Ob&Gyn, Osaka City University, Osaka 545-8585 Japan

Cancer cachexia is characterized by progression of wasting in adipose tissue and muscle following tumour growth. Muscle is more important to the quality of life of cancer patients, but the cause of muscle wasting is not well understood. We surmised that some plasma factor has an inhibitory effect on myoblast proliferation, and that the ability of muscle cells to regenerate decreases following tumour growth. We then hypothesized that the plasma factor could be removed by plasma perfusion, and that myoblasts would be able to proliferate from the satellite cells of the muscles of rabbits whose plasma was perfused. We therefore measured the proliferation of myoblasts from tumour-bearing rabbits in primary culture as a means of confirming our hypothesis. Rabbits implanted with VX2 carcinoma were treated by cyclic plasma perfusion, and the results was less weight loss and longer survival than in untreated rabbits. The urinary 3-methylhistidine/creatinine ratio was less elevated in rabbits treated by cyclic plasma perfusion than in the untreated rabbits. The DNA synthesis rate of myoblasts from rabbits treated by cyclic plasma perfusion was much better maintained than in myoblasts from untreated rabbits. Plasma fractions obtained from a rabbit with VX2 carcinoma and eluted from a phenyl-Sepharose column at NaCl concentrations under about 1.0 M decreased the myoblast proliferation and DNA synthesis. These results suggested that some humoral factor in plasma of rabbits with VX2 carcinoma inhibits proliferation of myoblasts and the deterioration of muscle may be improved by plasma perfusion.

### P112 THE EFFECT OF FATTY ACIDS ON EPITHELIAL CELL PROLIFERATION IN ORGAN CULTURE.

E. Pitt*, C.P. Diggle, J. Southgate & L.K. Trejdosiewicz. ICRF Cancer Medicine Research Unit, St James's University Hospital, Leeds, LS9 7TF.

The mechanisms by which fatty acids (FA) may suppress or enhance tumour development and/or progression are unclear. The anti-proliferative or cytotoxic effects of polyunsaturated (PuFA) on carcinoma derived cell lines may represent a general response of rapidly proliferating epithelial cells, rather than a feature of the transformed epithelial phenotype. This would be in agreement with findings that PuFA were growth inhibitory to normal human urothelial (NHU) cells, which have a high proliferative index in monolayer cultures (Southgate *et al, Br J Cancer 1996*; **74**:728.). The aim of this study was to establish the effect of PuFA on urothelial cells in a homeostatic tissue environment at equilibrium.

Organ cultures were established from surgical specimens of normal urothelial tissues and maintained at an air-liquid interface. Media were supplemented with BSA-complexed FA (stearic, oleic, linoleic, γ-linolenic, α-linolenic, icosapentaenoic and docosahexaenoic acids). In all cultures, initial regeneration of the urothelium was followed by maintenance of a long-term homeostasis, with low cytoproliferation, preservation of a normal stratified transitional epithelial morphology and expression of characteristic differentiation-associated antigens.

Whether added at initiation of culture or following attainment of equilibrium, PuFA had no apparent effect on urothelial cell proliferation, as determined by histological criteria or immunolabelling for Ki67/MIB-1. Even concentrations of PuFA which were irreversibly cytostatic on monolayer cultures of NHU, did not apparently affect attainment or maintenance of homeostatic equilibrium. PuFA also had little discernible effect on the production of basement membrane components (laminin, collagen IV) or on the expression patterns of E-cadherin or cytokeratin isotypes (CK7, CK8, CK13, CK17, CK18, CK19) characteristic of the normal tissue *in situ* and of control organ cultures.

These findings show that PuFA have little effect on quiescent urothelial cells in a homeostatic tissue environment, compared with the dramatic effects on isolated, rapidly-dividing cells in monoculture. This supports the hypothesis that anti-proliferative effects of PuFA are related to baseline proliferation rate.

### P113 THE OCTAPEPTIDE SOMATOSTATIN ANALOGUE RC-160, SUPPRESSES IGF-I AND ELEVATED PROLACTIN LEVELS IN BREAST CANCER PATIENTS.

KJ O'Byrne*[1], N Dobbs[1], DJ Propper[1], JP Braybrooke[1], MI Koukourakis[1], DC Talbot[1], AV Schally[2], AL Harris[1], [1]ICRF Oncology Unit, Churchill Hospital, Oxford OX3 7LJ, and [2]Tulane University and VA Medical Centre, New Orleans, Louisiana 70112-2699, USA.

RC-160 (octastatin/vapreotide) is a potent octapeptide analogue of somatostatin with growth inhibitory activity in experimental tumours *in vitro* and *in vivo* including breast cancer. The efficacy of high dose RC-160 was studied in 14 women, age range 37 - 80 years (median 58.5 years) and performance status 0 - 2, with pre-treated metastatic breast cancer. The RC-160 was administered by continuous s.c. infusion. The starting dose was 3 mg/day in week 1 and was increased to 4.5 mg/day for weeks 2 to 4 and subsequently to 6 mg/day until end of treatment. A significant reduction in IGF-I levels occurred by day 7 ($p<0.0001$) and was maintained throughout the treatment. The lowest dose of RC-160 produced the maximal IGF-I response. Although there was no reduction in prolactin levels in patients whose baseline levels were normal, elevated prolactin levels found in 3 patients fell to within the normal range 7 days after commencing RC-160 treatment. RC-160 was well tolerated with no dose reductions being required. No grade 3 or 4 toxicities were seen. The most common side-effects were mild fatigue and diarrhoea the latter being associated with steatorrhoea in 5 cases. Abscess formation developed at the infusion site in 8 patients and erythema and discomfort was seen in a further 3 patients. A small but significant rise in fasting blood glucose levels was also recorded ($p=0.0012$), the highest level on treatment being 7.6 mmol/L. No objective tumour responses were observed, all patients showing disease progression within 3 months of commencing treatment. These findings demonstrate that high-dose RC-160, administered as a continuous subcutaneous infusion, can reduce serum levels of the breast growth factors IGF-I and prolactin but is ineffective as single agent therapy in the management of metastatic breast cancer. Encouraging preclinical anti-tumour activity and the favourable toxicity profile in patients suggest the merit of future studies combining RC-160 with anti-oestrogens and/or cytotoxic chemotherapeutic agents.

### P114 BREAST CANCER CELL-ASSOCIATED NEUTRAL ENDOPEPTIDASE EC 24.11 MODULATES PROLIFERATIVE RESPONSE TO BOMBESIN.

Deirdre M. Burns[1], Brian Walker[2], Joseph Gray[2] & John Nelson[2]
[1]School of Biomedical Sciences, University of Ulster at Jordanstown, Co. Antrim, N. Ireland & [2]Division of Biochemistry, School of Biology and Biochemistry, Queen's University, Belfast, Northern Ireland.

Bombesin-like peptides have been shown to be mitogens for human breast cancer cells but the possibility of an autocrine role for gastrin-releasing peptide (GRP) remains unclear. We have investigated the production and inactivation of GRP-like peptides and their growth effects in human breast cancer cell lines. Radio-immunoassay detected GRP-like immunoreactivity (GRP-LI) in T47D breast cancer cells but not in their conditioned medium, indicating rapid degradation. No GRP-LI was found in the ZR-75-1 or MDA-MB-436 cells or their conditioned medium. HPLC analysis of the GRP-LI in the T47D cells revealed a major immunoreactive peak whch co-eluted with $GRP_{18-27}$ and a minor more hydrophilic peak. *In vitro* stimulation of T47D cell growth by bombesin was enhanced to 154% of control levels by the addition of the selective EC 3.4.24.11 inhibitor phosphoramidon (1 $ngml^{-1}$). Fluorogenic analysis using whole cells confirmed low levels of this enzyme on the T47D cells. This phosphoramidon-sensitive activity was approximately 100-fold less than that measured in the SCLC cell line NCI H345 where EC 3.4.24.11-mediated regulation of GRP levels has been shown in the past. These results suggest that a GRP-LI autocrine loop may exist in a subset of breast cancer cell lines. These peptides are rapidly degraded and inactivated extracellularly by a enzyme with an EC 3.4.24.11 profile. This enzyme, previously unreported in human breast cancer cells, significantly modulates both T47D basal growth and its response to BN-induced growth.

### P115 MOLECULAR REGULATION OF INTESTINAL EPITHELIAL REGENERATION,

A. Sattar*, B. Angus, F. C. Campbell, Dept. of Surgery, The Medical School, University of Newcastle, NE2 4HH.

We have developed a model of intestinal epithelial regeneration, a process central to inflammatory, ulcerative and infective diseases of the intestine, in order to investigate regulatory mechanisms which govern this process.
Human intestinal foetal tissue at 12-20 weeks gestation was retrieved and washed. The epithelial tissue was then desegregated mechanically and enzymatically by collagenase and dispase, and then differentially centrifuged to obtain a homogeneous population of crypt progenitor epithelium. This preparation was then grafted subcutaneously (20ul/graft) into SCID (severe combined Immunodeficient) mice and the graft retrieved at intervals of 7, 14, 21, 35, and 50 days after grafting to study the morphological features and differentiation processes.
21/26 grafts were successfully taken. Distinct stages of regeneration were seen; at early stages of regeneration (7 days) a 'blastema' of undifferentiated cells were seen, this then gave way to crypt formation at 21 days, with crypt fission and early morphological features by 35 days, by 50 days cytodifferentiation and morphological maturation was observed.
We present here a novel method of human intestinal epithelial regeneration to aid in the study of molecular regulation at all stages of intestinal epithelial regeneration.

### P116 HETEROTYPIC UROTHELIAL:STROMAL RECOMBINATIONS FOR BLADDER RECONSTRUCTION

MT Comer[1]*, DFM Thomas[2], LK Trejdosiewicz[1] & J Southgate[1]. ICRF Cancer Medicine Research Unit[1] and Department of Paediatric Urology[2], St James's University Hospital, Leeds LS9 7TF, UK.

The surgical manipulation of bowel to form a substitute urinary bladder is currently used following radical cystectomy for transitional cell carcinoma. However, it is associated with a number of problems due to the absorptive/secretory nature of intestinal epithelium compared to the barrier function of urothelium. We are developing "composite enterocystoplasty" in which de-epithelialised bowel segments will be relined with urothelial cells generated *in vitro*. We have shown that adequate numbers of normal human urothelial cells can be generated rapidly *in vitro* (Hutton *et al, J Urol 1993;* **150**: 721). Cells expanded *in vitro* will reform a histologically normal urothelium when seeded onto de-epithelialised bladder stroma in organ culture (Scriven *et al, J Urol 1997;* **158**: 1147). Prior to the development of a surgical model in the pig, it is essential to determine if cultured porcine urothelial cells would form a urothelium, or transdifferentiate into enterocytes following combination with de-epithelialised stroma from bowel or stomach.

Porcine stomach was de-epithelialised by dissection, leaving the submucosa. Normal porcine urothelial cells propagated *in vitro* to passage 2 were seeded onto the de-epithelialised stomach and maintained for 2 and 4 weeks in organ culture. These cells formed a histologically-normal stratified urothelium by 4 weeks. Collagen IV and laminin were expressed along a clearly defined basement membrane at the epithelial:stromal junction. Cytokeratin 13, which is expressed by urothelium but not gastric epithelium *in situ*, was positive throughout the neo-urothelium. A urothelial-specific differentiation marker, AUM, was expressed along the apical edge of the superficial cells. Identical results were obtained on allogeneic and autologous tissue recombinations.

Thus, urothelial cells can attach, lay down a basement membrane and form a stratified urothelium with expression of appropriate urothelial differentiation antigens when seeded onto stomach sub-mucosa in organ culture. No evidence was found for tissue-specific or instructive stromal signalling. These data support the concept of "composite enterocystoplasty".

*Acknowledgements*: This work was supported by Action Research and Ethicon Ltd.

### P117 HOX11 IS DIFFERENTIALLY EXPRESSED BETWEEN THE COLON CANCER CELL LINES SW480 AND SW620.

A. Parle-McDermott*, P. McWilliam, O. Tighe, D. Dunican and D.T. Croke. Molecular Biology Lab., Dept. of Biochemistry, Royal College of Surgeons in Ireland, 123 St. Stephens Green, Dublin 2, Ireland.

The ability of cells to respond to extracellular stimuli and to transduce signals *via* various pathways is due largely to reversible protein phosphorylation. This is achieved by the activities of a variety of protein kinases and phosphatases[1]. The most abundant of the Ser/Thr phosphatases include PP1, PP2A, PP2B and PP2C. PP1 is involved in a large number of cellular processes including glycogen metabolism, cell cycle regulation, RNA splicing and neuronal function[1,2]. The catalytic subunit of PP1 (PP1C) is bound by a number of regulatory subunits including the heat stable inhibitors: inhibitor-1 and inhibitor-2 (IPP-2)[3].

IPP-2 was recently found to be differentially expressed between the isogenic cell lines SW480 and SW620[4]. SW480 is derived from a primary colon carcinoma and SW620 is derived from a lymph node metastasis in the same patient. Both PP2AC and PP1C have been reported to interact with Hox11, an orphan homeobox gene. Hox11 is thought to act as an inhibitor of both PP2A and PP1, which disrupts a G2/M cell cycle checkpoint resulting in genomic instability and oncogenesis[5]. As both Hox11 and IPP-2 are putative inhibitors of PP1 and we have previously shown IPP-2 to be differentially expressed between SW480 and SW620[4], we were also interested in testing for Hox11 differential expression in these cell lines.

We have examined the expression of Hox11 in SW480 and SW620 using the technique of Semi-quantitative RT-PCR[6]. Semi-quantitative RT-PCR is a sensitive method for detecting the relative levels of expression of specific genes. We have modified this technique by inclusion of a Cy5-labelled primer in all PCR amplifications. This renders the resulting PCR product fluorescently labelled which allows for accurate and sensitive detection and quantification using the ALF express automated sequencer (Pharmacia). We have found Hox11 expression to be upregulated in SW480 relative to SW620. The RT-PCRs were controlled by amplification in parallel of β-actin. Identification of the Hox11 RT-PCR product was confirmed by cloning and sequencing.

(1) Barford D. Trends Biochem. Sci. 1996; 21: 407-412.
(2) Kwon YG, Huang HB, Desdouits F, Girault JA, Greengard P and Nair AC. Proc. Natl. Acad. Sci. (USA) 1997; 94: 3536-3541.
(3) Sanseau P, Jackson A, Alderton RP, Beck S, Senger G, Sheer D, Kelly A and Trowsdale J. Mammalian Genome 1994; 5: 490-496.
(4) McWilliam P, Parle-McDermott A, Dunican D, Tighe O and Croke DT. (MS in preparation)
(5) Kawabe T, Muslin AJ and Korsmeyer SJ. Nature 1997; 385: 454-458.
(6) He M, Liu E and Conway K. Edited by Larrick JW and Siebert PD. Reverse Transcriptase PCR. Ellis Horwood Limited 1995: 289-299.

### P118 DOES A CORRELATION EXIST BETWEEN URINARY EGF, pH AND TUMOUR EGFR STATUS IN BLADDER CANCER PATIENTS

J.L. Ritchie*[1], G.B. Nevin[1], S.R. McKeown[1], S.R. Johnston[2], I.K. Walsh[2], [1] School of Biomedical Sciences, University of Ulster, N. Ireland BT37 0QB, [2] Dept. of Urology, Belfast City Hospital, N. Ireland.

Urinary EGF levels are decreased in bladder cancer patients (Messing and Murphy-Brooks, 1994; Chow et al., 1994). EGFR overexpression is associated with poor prognosis (Mellon et al., 1995) and is noted most often in patients with invasive tumours. EGF binding to the urothelial receptor is inhibited at acidic pH (Hagler et al., 1980). Urinary EGF levels may therefore be higher when pH is less than seven. This study aims to determine whether there is an association between reduced urinary EGF levels, increased urinary pH and EGFR overexpression in tumour tissue from bladder cancer patients.

First morning urine samples were collected from 15 bladder cancer patients just prior to tumour resection. The pH was recorded and urinary EGF levels determined using a sandwich ELISA technique (R&D). Formalin fixed, paraffin embedded tissue from the resected tumours was stained by the avidin-biotin complex using the Ab4 EGFR antibody (Oncogene Science).

EGFR positive patients (mean 14.2 ng EGF/mg creatinine) had significantly (p=0.0465) lower levels of urinary EGF than EGFR negative patients (mean 20.4 ng EGF/mg creatinine). The mean urinary pH for EGFR positive and negative patients was 6.0 and consequently pH was not significantly associated with EGF or EGFR status.

These results suggest that EGFR overexpression but not urinary pH may account for the reduction in urinary EGF levels in bladder cancer patients.

Chow NH, Tzai TS et al., 1994 Urol Res, 22: 221.
Hagler HT, Maxfield FR et al., 1980 J Biol Chem, 255: 1239.
Mellon K, Wright C et al., 1995 J Urol, 153: 919.
Messing EM, Murphy-Brooks N, 1994 Urol, 44(4): 502.

### P119 SELECTIVE TRAFFICKING OF A TYROSINE-PHOSPHORYLATED ERBB2 ISOFORM TO THE GOLGI DISTRIBUTION.

Ouyang X.,*, Zhang H., Huang G., Moss J., Coulton G., Epstein R.J., Imperial College School of Medicine, Royal Postgraduate Medical School, London.

Overexpression of wild-type ErbB2 transforms cells in vitro and is linked to tumor progression in vivo, but the mechanism by which this orphan receptor exerts its oncogenic effect remains unclear. Here we use isoform-specific receptor antibodies and electron microscopy to show that a tyrosine-phosphorylated ErbB2 subset ($PY^{1222}$-B2) immunolocalizes to the Golgi distribution following ligand-dependent heterodimerization and transphosphorylation by heterologous growth factor receptors. This localization contrasts with that of activated epidermal growth factor receptors (EGFR*) and a distinct tyrosine-phosphorylated ErbB2 subset ($PY^{1248}$-B2), both of which exhibit a diffuse distribution of punctate cytoplasmic immunofluorescence. Golgi immunostaining of $PY^{1222}$-B2 is also induced by cell treatment with the tyrosine phosphatase inhibitor sodium orthovanadate, indicating that tyrosine phosphorylation suffices for ErbB2 localization to this site. Confocal microscopy shows that pre-treatment with the protein transport blocker brefeldin A (BFA) causes dissolution of Golgi immunofluorescence and retention of $PY^{1222}$-B2 on the plasma membrane, consistent with previously reported effects of BFA on receptor internalization. Immunoblotting studies reveal that BFA priming also diminishes the EGF-inducible $PY^{1222}$-B2 signal, and that this is in turn associated with shortened EGFR* signalling and EGFR expression. These findings suggest that $PY^{1222}$-B2 is endocytically targeted to the *trans*-Golgi network — perhaps reflecting the lack of a lysosomal targeting motif — and raise the possibility that unliganded ErbB2 may promote tumor growth by 'hijacking' liganded hetero-oligomers to a receptor recycling pathway.

### P120 THE ROLE OF TGFβ$_1$ IN PROLIFERATION CONTROL IN HUMAN COLON CANCER.

N.Aljehani, J.Bowman, J.A.Royds, J.Lawry. Institute for Cancer Studies, University Medical School, Beech Hill Road, Sheffield. S10 2RX.

TGFβ$_1$ is one member of a family of regulatory proteins having variable effects depending upon the cell type, stage of differentiation and ambient growth conditions. It is generally growth inhibitory on epithelial cells, acting via three surface receptors (Type I, II and III) to regulate G1 to S-phase progression via cyclin dependent kinase inhibitors (CDKIs) p15, p21 and p27. In colon tumour progression, TGFβ$_1$ responses are lost.

*In-vitro* proliferation studies were undertaken using flow cytometry to measure cell cycle, BrDu incorporation, growth factor receptor, and CDKI expression in colon cancer cell lines.

Under serum-free conditions, SW742 cells were unresponsive to TGFβ$_1$ whilst HT29 cells were growth inhibited. BrDu analysis confirmed cell cycle measurements and did not reveal any changes in cell cycle rate. Both cell lines expressed lower levels of Type I receptor than Type II. All receptor expression was reduced by TGFβ$_1$

| | HT:TGF- | TGF+ | | SW:TGF- | TGF+ | |
|---|---|---|---|---|---|---|
| | mean Ch | mean Ch | P value | mean Ch | mean Ch | P value |
| Type I | 22.9 | 20.3 | 0.4 | 46.8 | 23.8 | **<0.01** |
| Type II | 72.6 | 50.3 | **0.02** | 272.3 | 246.3 | 0.12 |

In the growth inhibited HT29 cell line TGFβ$_1$ significantly reduced p15 (P=0.02) and p21 (P=0.04); in contrast,TGFβ$_1$ increased p15 and p21(P=0.04) in SW742 cells.

**This research is funded by Yorkshire Cancer Research and the Ministry of Higher Education, Saudi Arabia.**

TGF-beta$_1$ colon cancer Flow cytometry

### P121 THE ROLE OF JAK / STAT SIGNAL TRANSDUCTION IN THE RESISTANCE OF MELANOMA TO INTERFERON TREATMENT

DP Jackson*[1], PM Patel[1], RE Banks[1], MS Burfoot[2], NC Rogers[2], D Watling[2], IM Kerr[2], PJ Selby[1]. [1] ICRF Cancer Medicine Research Unit, St James's University Hospital, Leeds, LS9 7TF. [2] ICRF, 44 Lincoln's Inn Fields, London, WC2A 3PX.

Interferon α is used in the treatment of malignant melanoma, with a response rate in advanced disease of 16%. Recently, the ECOG 1684 study has demonstrated a long term survival benefit in an adjuvant setting for patients with resected high risk primary disease treated with high dose interferon α2b. However this benefit is limited to only 11% of patients. These clinical observations suggest heterogeneity in the response of melanoma cells to interferon. Similar variation in response has also been demonstrated in melanoma cell lines *in-vitro*. An important pathway for signal transduction by interferon, is via the Janus Kinases (Jaks) and Signal Transducers and Activators of Transcription (STATs), although interferon is also known to activate other signalling pathways. Defects in Jak / STAT signalling may be responsible for the interferon resistance of melanoma cells both *in-vitro* and clinically.

Eight established melanoma cell lines were screened for their sensitivity to the anti-proliferative effects of interferons. Of these, MM418 was found to be the most sensitive with growth inhibition of 80-100%, whereas MeWo was the most resistant cell line, with growth inhibition of 10-40% depending on the class of interferon used. By flow cytometry, both cell lines were demonstrated to have components of the type I and type II interferon receptors. Also both cell lines upregulate MHC Class I in response to type I and type II interferons, and MHC Class II in response to type II interferon.

Both MM418 and MeWo express the components of the Jak / STAT signalling pathway, as detected by immunoprecipitation and western blotting. Ligand binding induces tyrosine phosphorylation and activation of these components as demonstrated by western blotting with anti-phosphotyrosine antibodies. Furthermore, on analysis by electrophoretic mobility shift assay, both cell lines are capable of forming functional, DNA-binding ISGF3 complex, STAT1 and STAT3.

These findings suggest that the resistance of MeWo cells to the anti-proliferative effects of interferons is not due to defects in Jak / STAT signalling, but rather due to either defects in downstream elements of the anti-proliferative response, or defects in additional or alternative signalling pathways that are required for this response. To determine the necessity of Jak / STAT signalling in the anti-proliferative response, these cell lines have been transfected with dominant negative Jak 1 and Jak 2 mutants.

### P122 MACROPHAGE INFLAMMATORY PROTEIN 1-BETA PROMOTER DRIVEN EXPRESSION IN TRANSGENIC MICE: POTENTIAL FOR CANCER IMMUNOTHERAPY.

R.B.Henderson*., A.Wood., I.Rosewell., P.J.Selby and P.M.Patel. ICRF Cancer Medicine Research Unit, St James's University Hospital, Beckett Street, Leeds. LS9 7TF.

The proximal promoter of the Macrophage Inflammatory Protein 1-beta (MIP-1β) drives expression in macrophages which is induced upon LPS activation (Profitt J. et al., 1995., Gene. 152:173-179). Utilising the MIP-1β promoter to target expression to antigen presenting cells within the proinflammatory immune response, we aim to activate specific anti-cancer T cells. MIP-1β promoter β-galactosidase reporter transgenic mice have been generated and reporter gene expression analysed at the RNA and protein level. By staining viable cells with a fluorogenic β-galactosidase substrate (Fluorescein di-β-D-galactopyranoside) a B220 (CD45R)$^+$, MHC class II$^+$ splenocyte population and a CD4$^+$, CD8$^+$, CD3$^+$ thymocyte population have been identified. Macrophages and Dendritic cell expression are currently being analysed by antibody staining.

β-galactosidase has been used as a surrogate anti-tumour T cell antigen in dendritic cell mediated tumour immunotherapy (Specht J.M. et al., 1997., J.Exp.Med. 186(8):1213). Following adoptive transfer of MIPLACZ bone marrow and specific cell populations to normal syngeneic mice we intend to analyse the T cell response to β-galactosidase expressing tumours.

MIP-1Beta Promoter Transgenic β-Gal Immunotherapy

### P123 THE EXPRESSION OF THE NOVEL PROINFLAMMATORY CYTOKINE EMAP-2 IN LUNG TUMOURS,

Maarten Tas[1]*, Lisa Jones[1], Andy Lee[1], Colin Clelland[2], James Carmichael[1] and Cliff Murray[1], [1]CRC Department of Clinical Oncology, [2]Department of Histopathology, City Hospital, Nottingham, UK

Endothelial monocyte-activating polypeptide 2 (EMAP-2) is a novel tumour-derived cytokine with proinflammatory properties, modulating endothelial cells, monocytes and granulocytes *in vitro*, and inducing an acute inflammatory response and tumour regression *in vivo* (Kao et al., 1994, J. Biol. Chem. 269; 25106). EMAP-2 is believed to be synthesised as a 34kDa precursor which is converted to the active 22kDa form. Using polyclonal (PoAb) raised against recombinant human EMAP-2 we studied the distribution of EMAP-2 in normal lung, small cell lung cancer, adenocarcinoma, squamous cell carcinoma and carcinoids. In normal lung tissue some smooth muscle cells and macrophages were found to be positive. In tumours, macrophages in some infiltrates were positive and in several cases diffuse staining of the stroma was observed. Tumour cells in all carcinoids were strongly positive, the well-differentiated being the most positive, showing cytoplasmic granular staining. The more poorly differentiated small cell lung cancer cells were negative or showed slight diffuse staining. The well differentiated adenocarcinomas were also positive. Several of the moderately differentiated squamous cell carcinomas showed slight diffuse staining. The strong granular staining of well differentiated carcinoids in immunohistology correlated with the presence of a 34 kDa band, corresponding to proEMAP, detected by Western blotting. Less differentiated carcinoids, with more diffuse staining, showed less proEMAP and more of the processed forms of EMAP-2 (27, 20 and 18 kDa). These data suggest that processing of EMAP may vary between tumours and may be related to tumour differentiation.

### P124 RECOMBINANT MYCOBACTERIA SECRETING CYT[illegible]INES

AM Jackson*, J. Haley, RSM Morgan, M. Zhu, M. Murphy, PJ Selby
*ICRF Cancer Medicine Research Unit, St. James's University Hospital, Leeds, UK*

The aim of these studeis is to improve the clincial "usefullness" of BCG. Our approach involves genetic modification of BCG and other strains of mycobacteria to make them more immunogenic.

Intravesical immunotherapy for CIS with live BCG vaccine is effective. However, 30% will not respond and there are occaisional serious complications. BCG works by promoting antigen specific and non-specific immune responses. Our previous studies have shown difference in the cytokine profiles between responders and non-responders. Therefore we are engineering cytokine-secretion by *M. bovis* BCG and *M. smegmatis* for cancer therapy.

Using *E.coli*-mycobacterial "shuttle" plasmids, expression of recombinant genes is driven via the heat-shock-protein promotors hsp60 and hsp70. The BCG alpha-antigen signal sequence is used to facilitate secretion of recombinant proteins from the mycobacteria. Whilst cytokine expression is driven by the hsp70 promotor, expression of *A. victoria* green-fluorescent-protein (*gfp*) is driven by hsp60. When excited by UV or laser radiation *gfp* emits visible green light making it particularly useful for localizing and tracking mycobacteria.

The levels of cytokine expression are highly heterogeneous; IL-7 (10pg/ml), IL-8 (50ng/ml),. IL-15 (300pg/ml), RANTES (20ng/ml), MCP-1 (50ng/ml), TNFα (100ng/m!). Importantly, these cytokines are in biologically active form. In particular we have studied the effect of r.*M. smegmatis*-TNF *in vitro*. Unlike wild-type bacteria these organisms can enhance the display of co-stimulatory molecules on bladder tumour cells and augment secretion of proinflammatory cytokines such as IL-6, TNF and IL-8. Bacteria recombinant for the *gfp* gene have been studied by immunofluorescent microscopy and flow-cytometry. In particular we have studied their interaction with bladder tumour cell lines.

The unique difficulties of gene expression in mycobacteria, our initial results on the biological activities of mycobacterial-derived cytokines, and the future uses for this system to express tumour antigens will be discussed.

Keywords: cytokine, BCG, mycobacteria, immunotherapy

**P125** INVESTIGATION INTO THE USE OF INTERLEUKIN (IL)-15 AS AN IMMUNOTHERAPEUTIC AGENT IN RENAL CANCER.

*M.J.Gough, R.E.Banks, A.M.Jackson, P.J.Selby, P.M.Patel. ICRF Cancer Medicine Research Unit, St. James's University Hospital, LEEDS, LS9 7TF.

IL-15 is a proinflammatory cytokine with a number of actions in common with IL-2 as a result of shared receptor usage. IL-2 is a central component in immunotherapy for renal cancer, but the optimal dose is limited by significant toxicity. IL-15 displays a reduced toxicity to IL-2 in mouse models and therefore may provide an alternative or parallel therapeutic option in renal cancer. In view of the chemoattractant and proliferative actions of IL-15 on PBMC, induction of IL-15 secretion by renal cells could potentially induce local anti-tumour immune responses.

We have demonstrated that IL-15 mRNA is present in a panel of malignant and normal renal cells using RT-PCR, but with no detectable secretion of IL-15 protein as measured by sensitive ELISA and biological assay. Importantly, renal cells express an incomplete set of IL-15 receptor components (IL-15R$\alpha$ 8/12; IL-2R$\beta$ 12/14; IL-2R$\gamma$ 0/14) and proliferation of renal cells is not affected by exogenous IL-15.

We are currently investigating a range of candidate stimuli for induction of IL-15 secretion from renal cells. A mixed lymphocyte-tumour culture model using renal cells has been established to investigate the therapeutic potential of IL-15 as an anti-tumour agent.

**P126** SENESCENCE AND NATURAL KILLER (NK) CELL PHENOTYPE AND FUNCTION.

R.Solana*, F.Borrego, B.Ostos, R.Ramirez, J.Carracedo and C.Alonso. S. Immunology. "Reina Sofia" Hospital. 14004 CORDOBA. Spain.

We have studied cytotoxicity, proliferation and expression of activation markers in purified NK cells from healthy Senior donors. The results show that whereas NK, LAK, and CD16 redirected lysis are not significantly affected in healthy elderly people, the expression of activation markers in NK cells and their induction in response to Interleukin 2 (IL-2) is significantly different to that of NK cells from young donors. Thus the expression of HLA-DR and CD95 (Apol/fas) is significantly increased in NK cells from senior donors while the expression of CD69 is decreased when compared to their expression in NK cells from young donors. IL-2 activation of NK cells induced CD69 expression in NK cells from young donors in a Protein Tyrosin Kinase (PTK) dependent but Protein Kinase C (PKC) independent pathway. However in elderly people the induction of CD69 by IL-2 was different, in the different individuals. CD69 expression in NK cells statistically correlated with NK cell proliferation but not with IL-2 enhancement of NK cytotoxicity. IL-2 activation of NK cells also induced "de novo" fas expression in NK cells from young donors and enhanced fas expression on NK cells from elderly donors. Crosslinking of fas in IL-2 activated NK cells by using anti-fas monoclonal antibodies induced apoptosis in a similar percentage of NK cells from young and elderly people. These results indicate that, although NK cytotoxicity is not significantly affected in healthy elderly people, changes in the phenotype and in other NK cell activities can be detected. These alterations migth be related to T lymphocyte disfuncyion in senescence.

**P127** IMMUNOTHERAPY FOR RELAPSED LEUKAEMIA.

[1]J. O' Riordan*, [1]N Gardiner, [2]A. O'Meara [1]K. Molloy, [1]M. Lawler, [3]R. Stallings, [1]SR McCann. Dept Hematology, St James's Hospital Dublin [2]Dept Oncology/ [3]National Centre for Medical Genetics, Our Ladys Hospital Crumlin.

While allogeneic BMT is a viable therapeutic option for both acute and chronic leukaemia, the problem of disease relapse remains. Possible rescue options in relapsed leukaemia include consolidation chemotherapy or a second BMT but these options are complicated by disease resistance and toxicity effects. A third option is an immunotherapeutic approach involving the infusion of donor lymphocytes from the original donor. This has proved successful option in > 70% of chronic myeloid leukaemia(CML) patients indicating a strong graft versus leukaemia effect. To date we have performed DLI procedures on 6 CML patients who have relapsed from 1-7 years post BMT. A clinical response was seen in all patients following a short period of cytopenia and a clinical remission has persisted for 6 months - 3.5 years post DLI. Polymorphic STR-PCR and RT-PCR detection of bcr-abl transcripts(the leukaemia specific transcript seen in CML) was performed at serial timepoints in unseperated peripheral blood or bone marrow and in selected lineages including CD34 and CD2 lineages. Lineage selection was performed using dynabeads attached to the appropriate monoclonal antibody and PCR techniques were modified to allow direct "dynabead PCR". STR-PCR and RT-PCR of bcr-abl has indicated a true"molecular cure" in these patients. In contrast to CML, the response rate to DLI in acute leukemia. particularly ALL; is very poor. We report an 11 year old boy with My+ALL who was transplanted in CR1 using a female unrelated donor mismatched at the DRB3 locus. The graft was T-cell depleted ex-vivo with Campath-1M and complement. Engraftment was prompt as judged by interphase fluorescent in situ hybridisation (FISH) and STR-PCR. The patient subsequently relapsed on day 172 post BMT but remission was achieved with Vincristine and prednisolone. DLI was performed at day +294 post BMT and the patient responded within 6 weeks. The patient is currently well with complete donor chimerism and no evidence of disease at 1 year post DLI. Thus DLI can exhibit an antileukaemic effect in both chronic and acute leukameia and is an effective therapeutic option in the majority of CML patients who relapse post allogeneic BMT. It may also be effective in certain cases of acute leukaemia and should be considered if no other therapeutic option is available.

**P128** RETROVIRAL GENE TRANSFER OF IL-18 *IN VITRO*

JR Wilson, PM Patel, A. Wood, PJ Selby, AM Jackson*
*ICRF Cancer Medicine Research Unit, St. James's University Hospital, Leeds, UK*

Interleukin-18 (IL-18) is a 22kDa cytokine which is processed by caspase I to a biologically active 18.3kDa form which is a potent inducer of IFN$\gamma$. As a result IL-18 promotes the development of a cellular, Th1 immune response, thought to be important in the destruction of bacterially-infected cells and in anti-tumour immunity. Previously we have shown that the mRNA for IL-18 is expressed by bladder, renal and ovarian cancer cell lines but not by neural crest-derived tumours (melanoma, neuroblastoma). Despite the expression of IL-18 mRNA , we have not been able to identify IL-18 protein in these cells. The aim of this work is to engineer secretion of IL-18 by tumour cells and investigate its effect on the promotion of anti-tumour immune responses *in vitro*.

We have cloned the full length IL-18 cDNA into the retroviral expression vector pBabe.Puro and used the Fly-A13 packaging cell line to produce disabled retrovirions. Bladder cancer (RT112, EJ18) and melanoma cell lines (A735, SkMel, MeWo) were infected with retrovirus and mRNA measured using Northern blot and protein via indirect bioassay and Western blotting. The effect of IL-18 transfected cells on the activation of allogeneic PBMC was studied *in vitro*.

A 1.4kbp sub-genomic and a 3.0kbp genomic transcript was identified in all transfectants. Bladder cancer cells also expressed the 1.1kbp transcript as expected. Supernatants from transfectants induced low levels of IFN$\gamma$ secretion from peripheral blood mononuclear cells (PBMCs). Co-culture of allogeneic PBMCs with transfected tumour cells did not increase their proliferation, and only augmented their cytotoxic activity against tumour targets in 1/5 tumours tested.

Failure to induce IFN$\gamma$ is likely to be due to the lack of processing of pro-IL-18. Therefore we have recently cloned the mature 18.3kDa form of IL-18 into the retroviral system and are investigating its function. We shall discuss our recent findings with retroviral transduction of bladder tumour cells with mature-IL-18 and the possible role of IL-18 in immune responses to cancer.

Keywords: IL-18, gene transfer

### P129 VOLUME-SENSITIVE CHLORIDE CURRENTS IN THE HUMAN BREAST CANCER CELL LINE ZR-75-1,

M.R. Preston[1], O. Alalami[1], J.H.J. Martin[1] & C. Garner*[2], [1]Dept. Biomedical Sciences, SHS, University of Wolverhampton, WV1 1DJ, [2]Dept. Chemical and Biological Sciences, SAS, University of Huddersfield, HD1 3DH.

It is known that expression of the 170 kDa protein P-glycoprotein (P-gp) is responsible for multidrug resistance (MDR), characterised by development of tumour cell resistance to a wide range of cytotoxic drugs. P-gp acts as an ATP-dependent transporter, increasing drug efflux and preventing effective therapy. Various studies have demonstrated that the expression of P-gp in a number of cell types is associated with chloride ($Cl^-$) currents which are activated by cell swelling (reviewed by Higgins, 1995). Tamoxifen is known to block the swell-activated $Cl^-$ conductance (Zhang *et al.*, 1994) and modulate the P-gp transporter in cancer cells (Fisher *et al.*, 1996), both actions are independent of action via the oestrogen receptor. We have examined the breast cancer cell line ZR-75-1 for the presence of swell-activated $Cl^-$ currents using the whole-cell configuration of the patch-clamp technique.

Cells were bathed in a NaCl isotonic saline, pH7.4 and were dialysed with pipette solution containing NMDG-Cl, ATP (2mM) and tamoxifen (10 microM), pH7.4. Cells were held ($V_h$) at -60 mV and stepped from -120 to +60 mV in 20mV increments. Using this protocol cells bathed in the isotonic saline displayed an outward $Cl^-$ current of 3.0±1.1 pA/pF at +60mV. Upon exposure to an 75% hyposomotic solution the cells showed an increase in the $Cl^-$ current to 20.4±10.2pA/pF (n=11, P<0.001, paired t-test), which was reversible upon returning to isotonic conditions. The conductance was confirmed to be selective for $Cl^-$ after replacement of the bath solution with a low Cl- solution reduced the outward current. In the absence of ATP there was no increase in current after exposure to hypotonic solution (from 2.2±0.4 pA/pF to 3.6±2.2 pA/pF, n=3), indicating ATP dependency. Exposure of swollen cells to 10 microM 4-(OH)-tamoxifen (n=3) and 10 microM tamoxifen (n=1) reduced the outward current by 71-82% and 90% respectively. We have confirmed that ZR-75-1 breast cancer cells have a swell-activated $Cl^-$ conductance, which is ATP dependent and is reduced by tamoxifen and 4-(OH)-tamoxifen. These results resemble previous investigations on other cell types which have been shown to express P-gp, hence the swell-activated Cl- conductance in ZR-75-1 cells may be associated with P-gp and MDR.

Fisher, G.A., et al., 1996, Eur. J. Can., 32A(6): 1082-.
Higgins, C.F. 1995, J. Biogenetics and Biomembranes, 27,63-.
Zhang, J.J. et al., 1994, J. Clin. Invest., 94, 1690-.

### P130 THE CHARACTERISATION AND ISOLATION OF TRANSLOCATIONS INVOLVING THE IGH LOCUS IN MULTIPLE MYELOMA,

J.A.L. Fenton*, J.A. Proffitt, G. Pratt, A.C. Rawstron, F.E. Davies and G.J. Morgan, Department of Molecular Oncology, Algernon Firth Building, University of Leeds, Leeds LS2 9JT

Multiple myeloma (MM) is a malignant disorder where plasma cells accumulate in the bone marrow. The use of FISH to analyse the DNA of MM patients has shown that rearrangements of the immunoglobulin heavy chain (IGH) locus at 14q32 can occur in up to 70% of cases, such a frequency can not be detected using conventional cytogenetics. In MM such rearrangements are localised to switch regions, sequences which are normally associated with isotype switching events at the IGH locus. Cyclin D1 located on chromosome 11q13 is commonly dysregulated as a result of a translocation of this gene into the IGH locus in mantle cell lymphoma. Similar translocations, only into the switch regions, are also seen in MM and the overexpression of cyclin D1 occurs as a result of such a translocation. We have used an RT-PCR assay to look for cyclin D1 overexpression in patient material. Following reverse transcription, a competitive PCR is utilised to monitor the expression of cyclin D1 against that of cyclins D2 and D3. We have studied 12 MM patients using the same RT-PCR assay and have found no evidence of cyclin D1 overexpression suggesting that in clinial cases of MM cyclin D1 overexpression is not a common event. We have also used this assay to demonstrate cyclin D1 overexpression in a novel myeloma cell line, K620. Interestingly cytogenetic analysis of K620 cells has shown that there is no translocation of the 11q13 locus to 14q32, but rather to other loci (1q32 and 8q24), suggesting an alternative, novel mechanism for cyclin D1 dysregulation.

Evidence suggests translocations involving switch regions in MM are nearly universal and involve a promiscuous array of partner loci. Genes such as cyclin D1 and FGFR3 have been identified in MM cell lines but clearly only patient material will yield evidence for representative molecular events. We have used Southern blotting to screen patients but this lacked the required sensitivity and so have gone on to devise a strategy where FISH will initially identify translocations into switch regions. A PCR-based method will then allow the identification and isolation of translocation breakpoints occurring within switch region sequences upstream of the $\mu$, $\gamma$ and $\alpha$ constant regions of the IGH locus. Firstly synthetic oligonucleotide linker units, called Vectorettes, are ligated to restriction digested genomic DNA. Amplification is then undertaken using primers complementary to sequences close to the various switch regions and the Vectorette linker. Vectorettes are designed containing a central mismatching region so that they are only amplified when attached to the end of a DNA fragment, and only after extension has occurred from the target sequence primer during the first cycle. Using this method of Vectorette-PCR we have isolated the breakpoints from JJN3 and K620 cell lines associated with $\mu$ switch regions. We are currently applying this strategy to DNA isolated from the bone marrow and blood of MM patients.

### P131 INDUCTION OF CTL RESPONSES BY RETROVIRALLY TRANSDUCED HUMAN DENDRITIC CELLS

A Protheroe*[1], MHM Heemskerk, H Spits. Department of Immunology, Netherlands Cancer Institute, Amsterdam, [1]Present Address: ICRF Cancer Medicine Research Unit, St James's University Hospital, Leeds.

The use of tumour-antigen transduced dendritic cells to stimulate anti-tumour responses from autologous T lymphocytes was studied. $CD83^+$ dendritic cells were diffentiated from cultured $CD34^+$ cells, obtained from leukapherised blood, using an immunomagnetic cell sorter, and incubated in medium containing a cocktail of cytokines (TNF alpha, SCF, GMCSF). At day one the cultured $CD34^+$ cells were retrovirally transduced with single tumour antigens (MART, MAGE 3, GP100 and Tyrosinase) or a polyepitope containing three HLA A2 restricted T cell epitopes (one derived from influenza and two from HPV 16) and a mouse restricted epitope. The constructs contained as a marker gene, enhanced green fluorescence protein (eGFP). Transduction was shown not to alter the cell phenotype as determined by FACS analysis (CD1a, CD4, CD11c, CD14, CD40, CD54, CD80, CD83, CD86, class II). Furthermore the stability of transduction over two months was demonstrated by FACS analysis of the eGFP signal. The ability to present transduced tumour antigen was also examined, by the use of T cell clones specific for HLA A2 restricted epitopes of the tumour antigen of interest. T lymphocyte anti-tumour responses were analysed by cytotoxicity assays following repeated stimulation by transduced dendritic cells in an autologous MLR, cultures were restimulated at weekly intervals. Preliminary results show that dendritic were able to present epitopes effectively with recognition of above 20% with a 3:1 target effector ratio using T cell clones. After two stimulations with the polyepitope transduced dendritic cells, T cells produced 50% lysis of an autologous transduced B cell line at an effector:target ratio of 90:1, recognising the influenza epitope.

### P132 INTERLEUKIN-12 GENE TRANSFER TO HUMAN BLADDER CANCER CELLS ENHANCES ANTI-TUMOUR IMMUNE RESPONSES INVITRO.

RW Carter, RE Banks, AM Jackson*, PJ Selby and PMPatel. ICRF Cancer Medicine Research Unit, St James's University Hospital, Leeds, UK LS9 7TF

Interleukin-12 plays a central role in cell-mediated immunity, enhancing proliferation and cytotoxicity of activated T and NK cells and inducing interferon-gamma production. We have investigated the effect of exogenous IL-12 and retroviral gene transfer of IL-12 on the allogeneic immune response to bladder cancer cell lines.

Recombinant retrovirus encoding the p35 subunit, the p40 subunit and both subunits together were engineered. These were used to transduce EJ and RT112 cells singly or in combination. No IL-12 secretion was detected when the p35 subunit alone was expressed. However when EJ but not RT112 were transduced with the p40 subunit alone, functional IL-12 was secreted. Functional IL-12 was produced when both subunits were transduced.

Allogeneic peripheral blood lymphocytes were incubated for 7 days with the parental tumour cell lines in the presence or absence of exogenous IL-12 and cytotoxicity against the parental tumour was assessed in a standard chromium release assay. Addition of exogenous IL-12 to the co-cultures increased the killing activity in the EJ co-cultures against the parental tumour and against K562 cells. This effect was seen in a dose-dependent manner at concentrations of IL-12 ranging from 10-1000pg/ul. This killing could be abrogated by 100 fold cold K562 inhibition. This effect was not seen with RT112 cells which failed to generate any cytotoxicity.

EJ cells but not RT112 cells engineered to secrete IL-12 increased generation of cytotoxic activity of co-cultured lymphocytes. This activity could also inhibited by cold K562 inhibition.

Lymphocyte subsets following co-culture with IL-12 and IL-12 secreting tumour cells were analysed by FACS. An increase in CD56 and CD69 positive cells was seen in all cultures containing IL-12. There was variation in the extent of this increase in different donors.

### P133 IDENTIFICATION OF URIDINE AS AN IMMUNE SUPPRESSIVE MEDIATOR IN OESOPHAGEAL CANCER.

J.F.Kenny*, G.O'Sullivan, F.Shanahan, and J.K.Collins; Depts.of Microbiology, Medicine and Surgery, University College, Cork, Ireland.

**Background:** Tumours may only survive within an immunocompetent host by escaping immunological surveillance through poor immunogenicity, or by inhibiting the immune response directed against it. In our studies of oesophageal carcinoma, we have shown that ~~primary oesophageal~~ tumours induce a regional immunosuppression within the host. We have established in our lab an oesophageal squamous carcinoma, OC2, which produces profound immunosuppression. In this report, we identified uridine as a potent mediator of OC2 tumour-associated immunosuppression. **Methods:** OC2 cells were grown as monolayers and as xenografts in nude mice with the resulting tumours used in explant production. Crude explant was fractionated using ultrafiltration to allow assessment of low mol.wt. suppressor molecules. A phenylboronate column was used to selectively extract nucleotides. Specific molecular identification was done using direct probe mass spectrometry. Urines were collected from healthy human volunteers (n=15) and from patients with established oesophageal or colonic tumours (n=20) for uridine analysis. Rev.Phase C18 HPLC column was used for low mol.wt. profiles of explant and tumour urines. Cell proliferation was measured by trypan blue cell counts, [$^3$H]-nucleotide incorporation assays, and cell cycle analysis by FACS. Actinomycin D was used to determine whether RNA breakdown was the uridine source. **Results:** We have previously shown that TGF-β was secreted by the OC2 tumour at immunosuppressive levels. Elimination of TGF-β by ultrafiltration allowed examination of low mol.wt. species such as modified nucleosides. Using rev.phase HPLC, a large bioactive immunosuppressive peak was detected in the mM conc. range. This was identified as uridine by mass spectrometry. Uridine at this conc. range of 1-2mM was found to be potently inhibitory for PBLs and cells of lymphoid/myeloid lineage, while OC2 cells tolerated high uridine concentrations. Investigation of human tumour urines showed that 15% had elevated uridine levels. Analysis of monolayer supernatants of other epithelial lines showed a similar uridine release to OC2 cells. Actinomycin D treatment of OC2 cells showed only a moderate decrease in uridine release. **Conclusions:** Tumours have been shown to release a variety of low mol.wt. suppressors, including nucleosides. The finding of uridine in such high concentrations was completely unexpected. Uridine may be a simple, relatively ubiquitous suppressor and may provide an opportunity for metabolic reversal. While RNA turnover is a common source of excreted modified nucleosides, OC2-derived uridine may be associated with excessive metabolic biosynthesis. Uridine may also serve as a neoplastic marker for established disease.

### P134 DEVELOPMENT OF A POLYEPITOPE DNA VACCINE FOR CANCER.

S.G. Smith*, P. Johnson, P.M. Patel, P.J. Selby and A.M. Jackson. ICRF Cancer Medicine Research Unit, St. James's University Hospital, Beckett Street, Leeds, LS9 7TF.

As the functions of many tumour associated antigens (TAA's) are unknown or often transforming, vaccines containing the full length cDNA's encoding these antigens are inherently dangerous. The combination of a number of minimal epitopes derived from TAA's as a polyepitope vaccine would remove this risk and allow vaccination against a number of antigens at once. Recent years have revealed DNA vaccines as potent stimulators of the immune response with many advantages over peptide-based vaccinations, for example prolonged antigen expression and ellicitation of both cellular and antibody responses, (Tighe *et al.*, 1998, Immunology Today, **19**:89).

In a pilot study, 17 epitopes from the gp75, gp100, MART-1, tyrosinase, BAGE, MAGE, GAGE and p15 tumour antigens were combined in a plasmid vector. Following transfection of NA-8MEL melanoma cells, 3/5 epitopes tested for (MAGE-1, tyrosinase, gp100) were shown to be presented on MHC class I when probed with antigen specific cytotoxic T-lymphocyte (CTL) clones (*unpublished data*).

A second generation construct encoding 8 HLA A1 or A2 restricted epitopes from gp100, tyrosinase, MAGE-1, MAGE-3 and MART-1 has been designed. Following demonstration of protein expression we intend to transfect and retrovirally transduce cells including professional antigen presenting cells for presentation and to stimulate naive T-cell responses *in vivo* following naked DNA administration.

### P135 THE EFFECT OF RECOMBINANT BCG SECRETING IL-15 ON ACTIVATION OF PBMC

Zhu X*, Petel P, Selby P, Jackson A.M. ICRF Cancer Research Unit, St. James's University Hospital, Leeds, LS9 7TF

Live BCG vaccine has been successfully used in the treatment of bladder cancer. Genetically engineering BCG and other non-pathogenic mycobacteria ( e.g. M. *smegmatis* and M. *vaccae* ) to secrete cytokines including IL-15 is thought to be a way to improve the clinical efficacy for cancer treatment.

IL-15 cDNA, which was amplified from reverse transcribed RNA obtained from activated PBMC, was cloned into the pMOD-8 mycobacterial expression vector, clones were established in BCG and M.*smegmatis* by eletroporation. The IL-15 secretion level from clones of M. *smegmatis* was 250 pg/ml (measured by ELISA), and the secretion level of IL-15 in BCG was even lower. A band of 14 Kda (the expected size of secreted IL-15) was also identified from Western blot of immunoprecipitation of the supernatant of the bacterial culture.

The supernatant of the clones did not stimulate the CTLL-2 cell line to proliferate. Suppression of lymphocyte killing activity was shown when BCG was added into the mixed lymphocyte and tumour cell culture (MOI=10:1), however, we observed that the recombinant IL-15 BCG could reverse this suppression, akin to the effect of exogenous IL-15. We hypothesise this is because the recombinant IL-15 rescues PBMC from appotosis caused by BCG.

Keywords: Mycobacteria, IL-15, Lymphocyte.

### P136 *Concomitant defective immune mechanisms and enhanced epidermal growth factor receptor (EGFr) expression in Oral Squamous Cell Carcinoma (OSCC).*

Cannell H & Nouri AME*. Depts. Oral & Max. Surg. & Med. Oncol. The Royal London Hospital. London. UK.

**Background.** In recent years investigation of the mechanisms for the development of human malignancies have focused amongst other areas upon:- a) escape from the immune system, b) induction of local anergy and c) induction of growth factor receptors.

The aim of the current studies was to utilize various techniques including immunocytochemical and cytotoxicity assays as well as an animal model of OSCC to investigate these three areas.

**Results**

a) tumour escape mechanisms as assessed by loss or defective expression of major histocompatibility antigens (MHC), as these molecules play a central role for recognition of self from non-self showed losses or defective expression (n=25) for Class I in 4% complete losses(monomorphic) and 80% defective (polymorphic) of cases of OSCC. Class II antigens not normally expressed on epithelial cells were expressed in 12/24 (50%) cases indicating local immune activation.

b) Epidermal growth factor receptor was expressed in 11/12 (91%) OSCC cases compared with 4/25 (16%) of the benign ameloblastoma thus demonstrating the relevance of this molecule in determining tumour aggressiveness.

c) Pre-immunization in a hamster model of OSCC with a suspension of autologous tumour resulted in a significant delay in subsequent grafted tumour growth (52.0±52mg versus 25.7±19.4 p=0.05 using ten animals per group tested. Mixing of autologous single cell suspension of irradiated tumour cells with normal spleen cells resulted in a significant decrease in efficiency of spleen cell killing using a standard tumour target in an vitro cytotoxicity assay thus demonstrating suppression of killing activity of the latter.

**Conclusions.** The data indicates that:- loss or defective class I antigen: over-expression of EGFr: induction of local anergy: are each phenomena associated with the potential for escape from surveillance as well for aggressiveness of OSCC. The findings may pave the way for enhancing immune related mechanism and/or suppression of growth factor receptors as treatment modalities for use in these patients.

### P137 EXPRESSION OF ECK AND LERK-1 DURING MELANOMA PROGRESSION.

David J Easty*[1], Mary E Fallowfield[2] and Dorothy C Bennett[1], [1] *St George's Hospital Medical School, London, UK; and* [2] *Western Infirmary, Glasgow, UK.*

The receptor tyrosine kinase ECK is overexpressed in most melanoma cell lines; the pathology where this elevation is initially manifested, and its possible role in tumor progression are unknown. To determine this we studied biopsies of benign and malignant melanocytic lesions. Normal melanocytes, benign compound nevi, and dysplastic melanocytes did not contain ECK, whereas it was detected in 20% of biopsies of malignant melanoma in situ (MMIS). Primary and metastatic melanomas did not contain immunoreactive ECK. LERK-1 (the ligand for ECK) is a melanoma growth factor (Easty et al., *Cancer Res.* 55: 2528-32, 1995); in addition, it is angiogenic and a chemoattractant for endothelial cells. LERK-1 co-localised with ECK in MMIS, but was also found in vertical growth phase (advanced) primary melanomas (43%) and metastatic melanomas (67%). Expression of LERK-1 was induced in melanoma cells by proinflammatory cytokines. These findings are consistent with two possible roles for LERK-1 in melanoma development, it may: (1) promote melanocytic cell growth or survival in early lesions, and (2) induce vascularization in advanced melanomas; both of these effects may be potentiated by an inflammatory stimulus.

### P138 QUANTITATION OF CEA AND OESTROGEN RECEPTOR EXPRESSION IN HUMAN BREAST CANCERS BY RADIOIMMUNOLUMINOGRAPHY.

R. Watson*, G. M. Boxer, A. Jones. CRC Targeting and Imaging Group, Department of Clinical Oncology, Royal Free and UCL Middlesex Medical Schools,, London, NW3 2PF.

Radioimmunoluminography (RILG) is a quantitative assay for measurement of tissue protein which preserves tissue morphology, for analysis of the relationship between structure and function. RILG has been established for quantitation of carcinoembryonic antigen (CEA) in histological sections[1]. The rationale depends upon the use of saturating concentrations of radiolabelled ($^{125}I$) antibodies to bind to the antigen present in the section. The distribution of bound antibody is then digitally mapped using a phosphor imager (Molecular Dynamics) after exposure of sections to phosphor storage plates. The amount of antigen present will be proportional to the amount of antibody bound in a given area of the section and can be calculated from a standard line generated from a nitrocellulose CEA dot blot assay.

In a preliminary study, paraffin sections of breast (7 cases) and colorectal (5 cases) adenocarcinomas were reacted with $^{125}I$ labelled A5B7 anti-CEA antibody (69 MBq mg$^{-1}$). 10ml aliquots of 0.3-5 µg g-1 CEA were applied to 0.45mm nitrocellulose (Biorad, U.K.) and the dots incubated with radioantibody. After incubation (1 hr) and washing in 5 changes of PBS/Tween (0.05%), sections and dot blots were air-dried and exposed to a phosphor storage plate overnight before scanning. Control sections of tumour were incubated with $^{125}I$-labelled anti-AFP antibody (A161). RILG with $^{125}I$ antibody to CEA gave a linear correlation with standards of known CEA concentration (r=0.994). Binding of $^{125}I$-antibody to duplicate sections ranged from 46-161 average counts per pixel for the breast cases and from 193-700 in the colorectal tumour sections. Concentration of CEA in the colorectal cancer sections was therefore calculated as ranging from 125-490ng g$^{-1}$ using the CEA dot blot calibration. The breast cancers studied had lower RILG counts and mean CEA concentrations were calculated to be less than 100ng g$^{-1}$. Visual inspection of RILG images showed hetrogeneity of antigen expression within each section and this was verified by the counts measured. Binding of anti-AFP antibody was non-specific and of a much lower order. Using RILG analysis, patients with breast cancer whose tumours express significant levels of CEA, can be identified and selected for antibody directed targeted cancer therapy.

A RILG assay using a $^{125}I$-labelled antibody (1D5 - Dako Ltd.) against oestrogen receptor (ER) has also been developed to quantitate levels of hormone in patients tumours. In a pilot study of 5 cases of breast carcinoma, saturating concentrations of 1D5 antibody were determined as 12.5 µg ml$^{-1}$. RILG values ranged from 0.1-57 average counts per pixel and did not correlate with ER status determined in the same patients by the biochemical dextran-coated charcoal (DCC) assay (range >10-890 fmole g$^{-1}$). These data support work which has reported discrepancies between biochemical DCC assays and immunohistochemical results. Quantitation of ER by RILG can help assess the relationship of patients hormone status with prognosis of disease and response to treatment.

[1] G.M. Boxer et al, British Journal of Cancer, 73, Supplement XXVI, 83.

### P139 *Development of new monoclonal antibodies specific for testis tissue.*

Dabare AANPM. Nouri AME and Oliver RTD. The Royal Londonl Hospital. London.UK.

Background. Early detection of malignant cells has had a big impact on the success of clinical management of human malignancies.

For this study a preparation of single cell suspension prepared from testis tissue of a patient with seminoma was used to develop monoclonal antibodies (Mabs) specific for germ cell tumours using Balb C mice. The specificity of these Mabs on various specimen was assessed by the conventional immunocytochemical staining technique. The data from two of the Mabs i.e. ATC1 and ATC2 developed are selected for presentation in this abstract.

**Results.**

ATC1. Screening of tissue biopsies from normal and malignant specimens showed negative staining on all the cases except a layer of cells just within the seminiferous tubules of tissue sections of testis with apparent normal morphology, most probably Sertoli cells.

ATC2. This Mab was found to show negativity on all normal tissues tested. In addition samples from tumours including bladder, kidney , prostate, head and neck, melanoma and leukemic cells were also found to show negative staining. The only type of tissues showing strong positivity with ATC2 was germ cell tumour particularly seminomas.

Screening of established human tumour cell lines of various origins showed staining patterns ranging from strong positivity in head and neck tumours (Hep2, KB ) to small percent positivity in breast ( MCF7, T47D) to complete negativity bladder (Fen, Wil). Biochemical analysis of the target antigen for ATC2 showed to have a molecular weight of about 70 Kd and strong thermotolerance.

Conclusion. These findings demonstrated the feasibility of raising specific Mabs against various components of germ cell tissues. The possible use of these Mabs for early detection of germ tumours in the semen of individuals suspected of germ cancers is being explored.

### P140 DISTRIBUTION OF DT-DIAPHORASE IN MALIGNANT AND NORMAL HUMAN LUNG TISSUE,

R M Phillips, BP Cronin, CM Jarrett, MC Bibby, Clinical Oncology Unit, University of Bradford, Bradford, West Yorkshire, BD7 1DP.

The enzyme DT-diaphorase (DTD, NAD(P)H: Quinone acceptor oxidoreductase EC 1.6.99.2) is involved in the bioreductive activation of a number of quinones and is thought to be a possible target for enzyme directed prodrug therapy. This idea is further strengthened by the fact that high DTD activity has been reported for a number of tumour types but particularly non-small cell lung cancer suggesting that appropriate patients could be selected for prodrug therapy. Although it is clearly important to determine enzyme activity in human malignancies versus normal tissues, such biochemical studies give no indication of cellular localisation of enzyme. In addition to biochemical assays on tissue homogenates this ongoing study therefore also assesses tissue distribution of DTD by the use of immunohistochemistry. To date DTD activities vary between 0 and 308 nmol DCPIP reduced/min/mg in malignant tissue (n=23) whereas in normal lung tissue they range between 0.48 and 43 nmol/min/mg. For histochemistry studies rabbit anti-rat DTD polyclonal antibody was used on wax blocks of adjacent tissue samples using an immunoperoxidase-APC technique. The specificity of the antibody was assessed by an ECL-Western blotting method, where binding correlated with previously determined levels of DT-diaphorase activity within the cell lines and solid tissues examined. Positive staining could be readily identified in tumour sections although it was not restricted to malignant tissue. Normal bronchiolar epithelium exhibited strong staining intensity but normal alveoli were negative. Tumours varied from no staining to homogeneous staining throughout the sections. Occasionally samples were largely negative but contained small areas of concentrated staining. Biochemical assays showed no DTD activity in these patients suggesting that enzyme assays alone may give false results. These results suggest that insufficient information is provided for patient selection by biochemical assay alone but immunostaining for DTD on wax blocks in conjunction with biochemical assays is feasible and will provide precise information on the presence of enzyme within the tumour.

Acknowledgements:
The authors would like to thank Richard Knox for supplying the antibody and the AICR and War on Cancer for supporting the work.

### P141 DETECTION OF TELOMERASE ACTIVITY IN LUNG TUMOURS AND BRONCHOALVEOLAR LAVAGE.

G. Xinarianos*[1,2], F.M. Scott[1,2], W. Prime[1,2], M. Walshaw[3], L. Turnbull[4] and J. K. Field[1,2]. [1]Roy Castle International Centre for Lung Cancer Research, Liverpool L3 9TA, [2]Molecular Genetics and Oncology Group, Clinical Dental Sciences, The University of Liverpool, Liverpool L69 3BX, [3]The Cardiothoracic Centre, Broadgreen Hospital, Liverpool L14 3PE, [4]Department of Pathology, The University of Liverpool, Liverpool L69 3BX.

The maintenance of telomeres is essential to the progression of cells through a normal, mortal life span. In tumour tissues and immortalised cells, telomeres are maintained by telomerase, a ribonucleoprotein enzyme, at lengths which are generally shorter than normal tissue. Telomerase is expressed in 80-90% of cancers and absent in most normal tissues. We have measured telomerase activity in lung cancer cell lines and lung tumours using the telomerase repeat amplification protocol (TRAP) (Kim, N.W., *et al.*, 1994, Science, 266: 2011). Four of 4 lung cancer cell lines and 16/20 lung tumours examined contained telomerase activity, which was not detectable in matching normal tissue. In order to investigate telomerase as a potential early detection marker for lung cancer, the enzyme will need to be measured in bronchoalveolar lavage (BL). Using the TRAP in a radioactive or nonradioactive format, telomerase activity was detected in 7/28 BL specimens from patients undergoing bronchoscopy for diagnosis of lung cancer. Issues regarding the effect of telomerase inhibitors in the BL specimens will be discussed.

*This research is supported by the Roy Castle Foundation.*

### P142 IN SITU TRAP ASSAY FOR LOCALISATION OF TELOMERASE ACTIVITY IN TISSUE SECTIONS,

MJ Thompson & MC Bibby. Clinical Oncology Unit, University of Bradford, Bradford, West Yorkshire, BD7 1DP, UK

Telomerase is a riboprotein responsible for the maintenance of telomeric regions at the ends of eukaryotic chromosomes. Telomeric regions of chromosomes are responsible for stabilisation of chromosome ends and the prevention of apoptosis due to chromosome shortening during cell division. As such telomerase is thought to be important in the immortalisation of cells, tumour formation and the function of stem cells. An example of a proliferating cell system is the seminiferous epithelium, a highly complex arrangement reliant upon stem cells to produce large numbers of highly differentiated spermatozoa.

The standard telomeric repeat amplification protocol assay (TRAP) clearly demonstrates high levels of telomerase activity within stem cell based tissues such as the testis. Because any tissue has to be homogenised, the assay cannot identify which cells within such a heterogenous tissue actually possess this activity. The physical isolation of different cell populations, whilst giving some cell specificity, remains an imperfect technique when faced with the wide variety of cell types present in many tissues such as the testis.

Using the adult mouse seminiferous epithelium as a model, this study applies a novel adaptation of the standard TRAP assay to study telomerase activity in cryosectioned tissue. This method enables RNase and heat sensitive telomerase activity to be identified within individual cells of a tissue. The results demonstrate that the nuclei of mitotic spermatogonia and meiotic spermatocytes express telomerase activity. Surprisingly, the non-dividing, differentiating spermatids were also found to possess similar activity, clearly located in the elongating spermatid heads. The late spermatocytes, early spermatids and mature spermatozoa have negligible telomerase activity.

This adaption of the TRAP assay is therefore a useful addition to the standard methods of determining telomerase activity and will allow us, for the first time, to examine the pattern of telomerase activity within tumour tissues.

This work was supported by War on Cancer.

### P143 DIFFERENTIAL EXPRESSION OF TSH RECEPTOR IN ARCHIVAL THYROID CARCINOMAS USING 5' NUCLEASE ASSAY (TaqMan).

Sheils OM*, Sweeney EC. Dept. of Histopathology, Trinity College Dublin

Prognosis in thyroid carcinoma is generally dependent on the patient's age and stage of tumour at the time of diagnosis. However tumours with several adverse features such as necrosis or high mitotic index may follow an indolent course while other seemingly inert ones may rapidly progress and have a fatal outcome. Proliferation indices using Ki-67 or mitotic counts and apoptotic counts are useful at either end of the spectrum of differentiation of tumours, but more accurate prognostication is required to predict the outcome of tumours whose histological appearance belies their sinister intent. Proliferation in thyroid carcinoma is variably TSH driven and TSH receptor (TSHr) status significantly relates to therapeutic response. TSHr expression was semi-quantitatively assessed in a series of archival thyroid carcinomas comprising follicular adenomas, follicular, papillary, medullary and anaplastic carcinomas. Total RNA was extracted from formalin fixed paraffin embedded tissues and reverse transcribed. To overcome the effect of different degrees of RNA degradation due to variations in storage conditions and duration of fixation, samples were analysed using GAPDH as a housekeeping gene.

The TaqMan detection system exploits the 5'- 3' endonuclease activity of Taq DNA polymerase which digests a double labelled internal fluorogenic probe during the amplification reaction. Prior to PCR the intact probe fluorescence of the reporter is suppressed by the quencher due to its spatial proximity. Digestion of the probe by Taq DNA polymerase results in separation of reporter and quencher dyes and a concomitant increase in fluorescence.

The fluorescent intensities obtained for TSHr and GAPDH were compared and a relative TSHr index was calculated for each sample. Results indicate the level of TSHr expression parallels the histologically graded degree of differentiation in the tumours assayed. Thus TSHr expression may prove to be an additional prognostic marker in thyroid carcinomas, and be of therapeutic value.

### P144 DOES THE ACIDIC DOMAIN OF THE MDM2 ONCOPROTEIN HAVE A TRANSCRIPTIONAL CONTROL FUNCTION?

Catherine Bladen*, John Lunec. Cancer Research unit, Medical School, University of Newcastle Upon Tyne, NE2 4HH.

The transforming properties of the MDM2 oncoprotein were originally attributed to its ability to bind and inactivate the p53 tumour suppressor protein. More recent studies have revealed that MDM2 has additional transforming mechanisms which are independent of the p53 interaction. We have characterised alternatively spliced forms of MDM2 which retain transforming ability in spite of losing p53 binding domain sequences (Sigalas *et al* 1996, Nature Medicine, 2 (8) 912-17 ) and studies with transgenic mice have shown that MDM2 is tumourigenic even in p53 null mice (Lundgren *et al* 1997, Genes and Development, 11(6) 714-25 ). These observations have led us to examine the role of alternative potential functions of MDM2, particularly the possibility of a transcriptional regulatory function, as suggested by the presence of zinc-finger motifs in the C-terminal region, including a RING-finger domain, and a putative acidic transcriptional transactivation domain. Here we describe the specific testing of the acidic domain of MDM2 for the ability to act as a transcriptional activation domain by fusing it to the DNA binding domain of a yeast Gal4 transcription factor and tested this hybrid (MDM2Ac-Gal4) for transcriptional function in a luciferase reporter gene system where the luciferase gene is driven by a Gal4 promoter.

A DNA fragment encoding the acidic domain of MDM2 (amino acids 223-274) was isolated by PCR and cloning from MDM2 cDNA and then subcloned downstream and in frame with the yeast Gal4 DNA binding domain already present in the PSG424 eukaryotic expression vector. Recombinant clones were identified by PCR screening and confirmed by DNA sequencing. These constructs were then co-transfected into NIH3T3 murine fibroblast cells together with the PGS-LUC plasmid, containing a luciferase reporter gene under the control of a Gal4 promoter, and PβGal encoding β-galactosidase to assess transfection efficiency. Expression of the luciferase gene resulting from transcriptional regulation by the MDM2Ac-Gal4 fusion protein was assessed by measurement of luciferase activity using the Dual Light™ (Perkin-Elmer) chemiluminescence system and a luminometer to quantify the light emission. Gal4-vp16 was used as a positive control and the PSG424 vector as a negative control. All transfections were carried out in triplicate. The luciferase activity generated with the intact Gal4-vp16 positive control was 1914 +/-100 times the background level seen with the vector only negative control, whereas the levels of activity obtained with the MDM2Ac-Gal4 fusion construct were not significantly different from the background; ratio 1:1.7. These observations indicate that the acidic domain of MDM2 does not behave as a positive transcriptional transactivation domain in this standard test system and do not support a transcriptional role for the MDM2 oncoprotein.

### P145 CD40 INDUCES AP-1 BINDING ACTIVITY, EXPRESSION OF CYCLIN D1 AND HYPERPHOSPHORYLATION OF pRb IN EPITHELIAL CELLS.

*Neil J. Gallagher, Aristides G. Eliopoulos, Lawrence S. Young. *Institute for Cancer Studies, University of Birmingham, Edgbaston, Birmingham B15 2TA, U.K.*

CD40, a member of the TNF/NGF receptor superfamily, is expressed in normal epithelial and B cells and a number of carcinomas. The interaction of CD40 with its cognate ligand, CD40L, regulates growth, survival and differentiation. The signalling pathways which mediate these phenomena are however not well understood. We have investigated the effects of CD40 ligation on the c-Jun N-terminal kinase (JNK) cascade in a number of epithelial carcinoma cell lines. Using *in vitro* kinase assays we have demonstrated that JNK activation rapidly occurs in response to treatment with recombinant soluble CD40L (rsCD40L). This phenomenon translates to induction of AP-1, a transcription factor which is readily activated by growth factors and mitogens. As the $G_1$ phase protein cyclin D1 is known to be regulated by AP-1, we have examined the effects of rsCD40L on cyclin D1 expression. Using semi-quantitative RT-PCR and western blot analysis, we have demonstrated a 3-4 fold induction of cyclin D1 RNA and protein levels following CD40 ligation in serum-deprived carcinoma cell lines. Futhermore, CD40 stimulation was found to induce hyperphosphorylation of the retinoblastoma protein pRb. This effect was rapid but gradually declined over 24 hours. These data identify the cell growth-regulatory cyclin D1 and pRb proteins as targets of CD40 signalling.

### P146 Should one exclude spontaneous regression before starting biological therapy in renal cancer?

RTD Oliver, M Leahy, D Farrugia, W Ansell. St Barts and The Royal London EC1A 7BE

Since 1978 164 RCC patients have been screened for entry into clinical trials and entered onto surveillance as first management policy until symptoms or serial radiology indicated tumour progression. 3+4 (4%) have demonstrated unexplained "spontaneous" CR+PR (median duration 17 mths), and 7(4%) "stable" disease (median 28 mths). None of 19 not nephrectomised regressed, while CR+PR was 11% of 40 nephrectomised at diagnosis of metastasis, 1% of 76 developing metastases 1-17 mths after nephrectomy and 10% of 29 developing metastases more than 18mths after nephrectomy. It was 12% of 47 with lung only verses 1% of 117 other sites. Possible evidence for relief of potentially immunosuppressive influences have been demonstrated in 4 of 7 patients demonstrating unexplained "spontaneous regression".

After progression on surveillance 114 patients have been entered into treatment trials. Clinical evidence of response 35% v 12% was higher in patients receiving αIFN/IL-2/5-FU (n=37) than other studies (n=77). High responses were also seen in patients with lung only as site of disease. Although these results suggest that no harm has come from a period of preliminary surveillance, the fact that the therapeutic benefits including durable complete remission from therapy are confined almost entirely to the good risk small volume asymptomatic patients makes it difficult to justify a policy of surveillance in such patients.

### P147 A DISTRIBUTION STUDY AT SUB-MILLIMETRE RESOLUTION OF A RECOMBINANT MFE-23::CPG2 FUSION PROTEIN IN A COLORECTAL CANCER XENOGRAFT MODEL.

Bhatia, J.*, Pedley, R.B., Sharma S.K., Boxer, G.M., Read, D.A., Boden, R.W., [1]Michael, P., Chester, K.A., & Begent, R.H.J. CRC Targeting and Imaging Group, Dept. of Oncology, Royal Free & UCL Schools of Medicine, Rowland Hill Street, London NW3 2PF. UK.[1]CAMR, Salisbury, UK

Antibody directed enzyme prodrug therapy (ADEPT) involves a two step strategy designed to deliver an enzyme to a tumour site, followed by the administration of a non-toxic prodrug. The prodrug is activated by the enzyme specifically at the tumour and results in selective killing of tumour cells. This system requires the enzyme to be delivered to viable tumour areas within a heterogeneous tumour mass. The sub-millimetre distribution of a bacterially produced recombinant fusion protein of MFE-23, an anti-carcinoembryonic antigen (CEA) single chain Fv (scFv), fused to the enzyme carboxypeptidase ($CPG_2$)[1] is presented.

Biodistribution of [$^{125}$I]-MFE-23::$CPG_2$ fusion protein and free [$^{125}$I]-$CPG_2$ were studied in nude mice bearing LS174T human colon adenocarcinoma xenografts. Percentage injected activity per gram of tumour at 6, 24 and 48 h was 6%, 3% and 2.5%, respectively for the fusion protein and 5.5%, 0.6% and 0.16% for free $CPG_2$. The corresponding blood levels were 4%, 0.2% and 0.06% for the fusion protein compared to 4.6%, 0.15% and 0.033% for free $CPG_2$. This gave tumour to blood ratios of 1.6, 18 and 42:1 for the fusion protein and 1.3, 4 and 5:1 for free $CPG_2$. These results indicate that the recombinant MFE-23::$CPG_2$ fusion protein has significantly higher tumour selectivity than the free $CPG_2$, while showing similar rapid plasma and normal tissue clearance. Successful ADEPT, however, relies on matching the fusion protein localisation and accessability to viable tumour with the pharmacokinetics of the prodrug employed.

To determine the optimal time for prodrug administration in relation to fusion protein localisation, and to study the non-specific retention of free $CPG_2$, autoradioluminography[2] (phosphor storage plate technology) was used to show the distribution within tumour and normal tissue. Preliminary results show selective localisation of the fusion protein in viable tumour regions compared to free $CPG_2$ which is retained predominantly in necrotic areas. Further phosphor image studies to determine the distribution at time points ranging from 1-72 h are in progress. The data generated form this model system can contribute to the development of potential ADEPT fusion proteins and refine prodrug scheduling.

Refs
1. Michael, P. *et al.* (1996) Immunotechnology **2**; 47-57
2. Pedley, R.B. *et al.* (1996) Cancer Research **56**; 3293-3300

### P148 Oxidative stress in patients receiving early enteral nutrition following operations for hepatic or pancreatic disease.

R Gupta[1], K Patel[1], JN Primrose[1], CD Johnson[1], P Yaqoob[2], P Calder[2], [1] Dept. Surgery, Southampton General Hospital, Southampton SO16 6YD, [2] Institute of Human Nutrition, University of Southampton, Southampton SO16 7PX.

**Background** : Oxidative stress and septic complications are an integral part of serious inflammatory conditions including serious sepsis and major blunt trauma. Aggressive nutritional support in the form of total enteral nutrition (TEN) is known to attenuate this phenomenon possibly by reducing translocation of endotoxin or micro-organisms from the gut lumen into the systemic circulation. There is a dearth of such data in respect of patients recovering from major abdominal operations.
**Aims** : The aim of our study was to investigate the effect of major abdominal operations in terms of oxidative stress and to ascertain if early enteral nutrition would have a beneficial effect.
**Methods** : We measured lipid peroxidation as an index of oxidative stress by quantifying thiobarbituric acid reactive substances (ug/l) in the plasma of 37 patients undergoing hepatic or pancreatic operations for benign or malignant disease. This was measured pre-operatively and again on day 3 and day 7 post-operatively. Patients were randomised to receive no nutritional support or total enteral nutrition immediately post-operatively. Our results are summarised in the table below.

**Results (ug/l)**

| Feed | n | Pre-op | Day 3 | Day 7 |
|---|---|---|---|---|
| Control | 22 | 3.8 (2.8) | 4.0 (1.8) | 4.4 (2.1) |
| TEN | 15 | 5.1 (4.6) | 5.7 (4.5) | 5.9 (3.0) |
| p | | 0.29 | 0.13 | 0.08 |

All values are mean (sd)

**Conclusions** : Our results suggest that although TEN is safe and well tolerated in post-operative hepatic and pancreatic patients there is no advantage in terms of attenuation of oxidative stress.

### P149 T CELL REGENERATION FOLLOWING MYELOABLATIVE CHEMOTHERAPY AND PERIPHERAL BLOOD PROGENITOR CELL (PBPC) RESCUE.

AS Protheroe*[1,2], C Pickard[2], PWM Johnson[1], T Craddock[2], PJ Selby[1], J Henwood[2], AW Boylston[2]. [1]ICRF Cancer Medicine Research Unit, [2]Department of Molecular Medicine, St James's University Hospital, Leeds, LS9 7TF.

Immunologic reconstitution following myeloablative chemotherapy and PBPC rescue was studied by examining the regeneration of the T cell receptor beta chain (TCRBV) repertoire in peripheral blood of patients. The TCRBV usage was analysed in twenty three patients with solid maligancies, before cyclophosphamide priming and monthly following high dose chemotherapy and PBPC rescue, using directly conjugated monoclonal antibodies to TCRBV 2,3,5S2/5S3, 7.1, 8, 9, 17, 21 and 22. Expansions of particular TCRBV families were found in four patients (TCRBV 3, 17, 21 and 22) ranging from 8 - 42% of the $CD4^+$ or $CD8^+$ repertoire, which reappeared within two months and were stable for up to eighteen months following PBPC rescue. The expansions in three patients (TCRBV 3, 21, 22) were further characterised by isolating $CD4^+$ or $CD8^+$ populations and sequencing the CDR3 portion of the gene rearrangement following RTPCR amplification of mRNA. The expansions were shown to be predominantly oligoclonal containing identical sequences before and after myeloablative chemotherapy. This study demonstrates that patients undergoing autologous PBPC rescue for high dose chemotherapy are able to regenerate a diverse T cell repertoire, consistent with pre-treatment repertoires, which occurs within eight weeks following rescue. Furthermore, that the overwhelmimg majority of T cells present following rescue arise from mature lymphocytes.

### P150 TUMOUR NECROSIS FACTOR ALPHA (TNFα) REGULATES THE EXPRESSION OF CD44 IN HUMAN ASTROCYTOMAS *IN VITRO*.

*Monaghan M. Radotra B. McCormick D. Oncology Department, The Queens University Of Belfast, University Floor, Belfast City Hospital Tower, Lisburn Road, Belfast. BT9 7AB.

Malignant astrocytomas are highly invasive tumours that rarely metastasize outside the central nervous system. Their ability to invade surrounding brain tissue leads to poor prognosis and mortality and is the main reason for recurrence after surgery. The process of invasion involves cell adhesion molecule-mediated interactions between tumour cells and components of the ECM. Amongst the glycosaminoglycans of the ECM, hyaluronic acid (HA) is known to be enriched in fetal brain tissue where it facilitates cellular migration during embryogenesis. HA is also increased in brain tumour ECM. The major cell surface receptor for HA is CD44 and earlier work in this laboratory has shown that CD44-HA interactions play a significant role in astrocytoma invasion *in vitro*. ( Radotra B. & McCormick D. J. Pathol.181:434-438.1997). The expression of many cell surface receptors is regulated by cytokines including TNFα which is known to be produced by reactive astrocytes and astrocytoma cells.

The aim of this research is to examine the potential role of TNFα in the regulation of expression of CD44 at the mRNA and protein levels. In a study of 20 glioma cell lines by Reverse Transcription Polymerase Chain Reaction (RTPCR), all expressed the standard form of CD44, in contrast to many non-CNS tumours in which splice variants are expressed. The expression of CD44 at the protein level was examined using flow cytometric analysis. In a pilot study, six astrocytomas showed upregulation of CD44 after treatment with 10ng/ml TNFα for 24 hours. This increased expression was correlated with grade, with high grade astrocytomas showing the greatest increase. Expression of CD44 mRNA was investigated using semi-quantitative RTPCR. Amplification of CD44 was stopped in the logarithmic phase of the PCR and GAPDH was included as an internal standard. TNFα has been shown to regulate expression of CD44 mRNA in all astrocytoma cell lines investigated.

In summary, the present investigation has demonstrated that TNFα modulates transcription and translation of CD44 in human astrocytomas *in vitro*.

### P151 HIGH EFFICIENCY CD34+ STEM CELL SELECTION FROM A SINGLE APHERESIS PRODUCT USING THE ISOLEX 300i SYSTEM.

N.Gardiner*, L.Doyle, J. Doyle, C.Duggan, S.R.McCann, P.V.Browne. Dept.Haematology, St.James Hospital, Dublin.

Peripheral blood stem cells (PBSC) are increasingly used for haemopoietic reconstitution following myeloablative chemotherapy. PBSC provide several advantages over bone marrow including more rapid haemopoietic recovery and reduced infusion volume. Selection of CD34-positive stem cells has the potential advantage of reducing tumor burden in the PBSC infusion. Clinical application of CD34+ cell selection requires effective mobilisation and high efficiency processing. We report our experience with CD34+ cell selection for autologous transplantation using the Isolex 300i system (Baxter). Patients with multiple myeloma (MM, n =3) were mobilised with ifosfamide, epirubicin and etopiside (IVE + G-CSF). Patients with non-Hodgkin's lymphoma (n=2), breast cancer (n=1) and Hodgkin's disease (n=2) were mobilised with cyclophosphamide and G-CSF. Peripheral blood stem cells were collected using the Baxter CS3000 leukapheresis system. A single leukapheresis contained sufficient cells for CD34 selection in 5 of 8 patients. In 3 cases two PBSC harvests were pooled prior to selection. Where necessary, cells were stored overnight with autologous plasma in gas permeable Lifecell bags (Baxter). Prior to selection, the median number of CD34+ cells was 8.0 x $10^6$ /kg (range 2.6 - 25.4). The median percentage of CD34+ cells was 3.3% (range 1.5 - 9.4). Following selection, the median number of CD34+ cells was 4.6 x $10^6$ /kg (range 1.7- 18.75). Median CD34+ cell recovery was 61% (range 56-80%).The CD34+ cell product had a median purity of 98.4% (range 95- 99.5%). Five patients have been reinfused with CD34+ stem cells following high dose chemotherapy. The median time to neutrophil count >0.5x$10^9$/ L was 11 days (range 9- 13) and to platelet count >20 x$10^9$/ L was 14 days (range 13-16).We conclude that in conjunction with improved mobilisation protocols such as IVE in multiple myeloma, the Isolex 300i system is highly efficient for clinical-scale CD34+ cell selection from a single apheresis product.

### P152 DNA TESTING PROVIDES PREDICTIVE DATA IN THE MANAGEMENT OF SEVERE APLASTIC ANAEMIA TRANSPLANT PATIENTS

Lawler M*., Gardiner N., Gowing H., McCann SR. Dept Hematology, St James's Hospital/ Trinity College Dublin Ireland

Bone Marrow Transplantation(BMT) is an effective therapeutic option for Severe Aplastic Anemia(SAA). However risk of graft failure/rejection ranges from 10-50% depending on factors including patient population, degree of histocompatibility match and conditioning therapy pre BMT. We have investigated the use of polymorphic DNA markers as early indicators of graft rejection. Initially 91 patients were studied for chimeric status following allogeneic BMT for SAA. A highly sensitive assay system using the polymerase chain reaction of short tandem repeats(STR-PCR) allowed precise monitoring of the evolution of hemopoietic chimerism. Informative polymorphisms were identified in all cases and used to assess chimerism in post transplant bone marrow and peripheral blood samples serially(3-15 samples per patient). STR-PCR indicated (A) complete donor chimeras (n=40), (B) transient mixed chimeras (n = 16) (C) stable mixed chimeras (n=18), (D) progressive mixed chimeras(n=17). In group A only donor cells were detected at all timepoints post BMT; there was only one case of poor graft function(2%) and 4 deaths in total(10%). In group B, recipient cells could be detected transiently, with re-establishment of donor hemopoiesis occuring up to day 327 post BMT; early graft failure was documented in one patient(6%) and 3 deaths(19%) occurred. Group C patients exhibited persistent and stable low levels of recipient cells(<15%); there were no episodes of graft failure/rejection and 1 death(5.5%) in this group. Group D showed high and/or progressive increases in recipient cells(>20%); in this group there were 13(76%) early or late graft failures/rejections and 9 deaths(53%). We analysed the following factors for association with mixed hemopoietic chimerism: age, sex match, donor type, etiology of the aplasia, number of cells engrafted, conditioning regimen, GvHD prophylaxis, occurrence of acute and chronic GvHD and survival. Progressive mixed chimeras were at high risk of early or late graft failure(n = 13, p= 0.0001). 7/13 patients lost their graft during withdrawal of immunosuppressive therapy. Progressive mixed chimerism was a bad prognostic indicator of survival(p = 0.003). These results have prompted prospective monitoring of chimeric status during immunosuppression withdrawal. Currently 18 patients are enrolled in a prospective study where dynabead PCR is also performed on separated hemopoietic lineages in an effort to identify which lineages may provide an indication of impending graft failure. Thus DNA testing can facilitate therapeutic intervention to prevent graft failure in patients transplanted for Severe Aplastic Anemia.

**P153** EXPRESSION OF CD44 mRNA IN NORMAL AS WELL AS TUMOUR COLORECTAL TISSUE, M. Morrin and P.V. Delaney, Colorectal Research Unit, Limerick Regional Hospital and University of Limerick.

Some of the variants of the CD44 cell adhesion molecule have been implicated in tumour metastasis formation, primarily CD44v6, but a lack of concensus has emerged in the more recent literature regarding the prognostic significance of CD44 variant expression. In this study the expression of CD44 variants at the mRNA level using RT-PCR was investigated to gain greater understanding of their role in metastasis.
Total RNA was isolated from a panel of colorectal tumour cell lines and from a series of normal/tumour tissue pairs, reverse transcribed and PCR amplified using primers from the constant region of the CD44 gene. This was then used as template for a series of "exon-specific" PCR reactions to analyse CD44 variant expression patterns. The PCR products were further investigated by Southern blotting and hybridisation with variant specific probes.
A complex and diverse array of CD44 mRNA transcripts was observed. 80% of the tumour: normal pairs demonstrated variant expression, although the transcripts were generally not as strongly expressed in the normal tissue. A sequential pattern of expression of the full range of variants was common. The CD44v6 epitope was demonstrated by immunohistochemistry in normal crypt base epithelia. The frequent expression of multiple CD44 mRNA transcripts in normal colorectal tissue suggests either a difference in translational mechanisms of these transcipts between normal and tumour tissue or alternatively a lack of significance in tumour development.

**P154** TUMOUR SUPPRESSOR GENES VERSUS POLY-A TRACT MARKERS IN THE DETECTION OF MICROSATELLITE INSTABILITY IN COLORECTAL CANCER, E.Nazem*[1], D.T.Bishop[2], P.Quirke[3] & C.G.Woods[1], [1]Clinical Genetics & [2]ICRF Genetic Epidemiology, St.James's Hospital, Leeds LS9 7TF, [3]Molecular Pathology, Leeds General Infirmary, Leeds LS2 9JT.
The majority of Hereditary Non-Polyposis Colorectal Cancer (HNPCC) and approximately 15% of sporadic bowel tumours have been found to exhibit Microsatellite Instability (MI) - the observation of novel alleles in tumour cells at loci throughout the genome. The MI phenotype results from defective repair of errors arising in the DNA strands during replication. Repetitive sequences, eg. (A)n and (CA)n repeats, are particularly susceptible to such errors because of DNA polymerase "slippage". The mismatch repair proteins, eg. hMLH1 and hMSH2 (human homologues of the bacterial MutL and MutS mismatch repair proteins), are involved in the recognition and correction of these errors and in HNPCC families and MI-positive tumours the genes encoding them are often found to contain pathogenic mutations.

There has been much debate over which microsatellite markers are able to detect MI-positive tumours most reliably and efficiently. Potential HNPCC families may be identified in this way, enabling at risk relatives to be screened for bowel cancer and other HNPCC-related cancers. Also MI-positive sporadic tumours may be a clinically distinct group with a better response to therapy. Therefore, establishing the MI status of a colorectal cancer is important.

We have compared the MI-detecting abilities of microsatellite markers within or near to three "classic" tumour suppressor genes (APC, p53 and DCC) with two markers containing poly-A tracts (BAT-26 and BAT-RII), by PCR and fluorescent gel electrophoresis of 52 cases of sporadic bowel cancer. BAT-26 contains a run of 26 adenosines and is located within intron 5 of the hMSH2 gene - one of the genes often found mutated in MI-positive tumours - and BAT-RII contains a run of 10 adenosines and lies within the TGF-β Receptor Type II tumour suppressor gene. Our results show that 7/52 (13.5%) cases were identified as MI-positive by at least one of the three tumour suppressor markers, while BAT-26 and BAT-RII both detected instability in 8/52 (15.4%) cases. A potential benefit of the APC, p53 and DCC tumour suppressor gene markers is that, as well as MI, they can also detect loss of heterozygosity - a frequent mechanism of inactivation of these genes in sporadic tumour progression. However, BAT-26 may have a greater advantage as it appears to be almost monomorphic in the wild-type and MI-positive tumours always seem to display alleles corresponding to a loss of at least 4 adenosines in the 26-adenosine run found in normal tissue. This could possibly eliminate a need for normal uninvolved tissue for comparison of allele lengths, making instability detection quicker and easier.

**P155 THE CORRELATION BETWEEN CELL SURFACE MARKERS AND CLINICAL FEATURES IN CHOROIDAL MALIGNANT MELANOMA.** **J.Lawry*, Z.Currie[1], M.O.Smith, M.A.Parsons[1], I.G.Rennie[1]. Institute for Cancer Studies, University Medical School, Beech Hill Road, Sheffield and Dept. Ophthalmology[1], Royal Hallamshire Hospital, Glossop Rd, Sheffield.**

**The aim of the study was to identity prognostically significant proteins in a group of 63 tumour samples taken from 59 patients having eneucleations for large choroidal melanomas. Tumour from four patients included areas with different clinical morphology (e.g. amelanotic vs. pigmented). McAb expression and DNA ploidy was measured by flow cytometry and correlated to clinical parameters (sex, age of the patient, tumour location (cilliary body vs. choroidal), cell type (spindle vs.mixed or epitheliod), tumour volume (<1500 mm vs. > 1500mm) and the presence of metastatic spread); using one-way-analysis of variance.**

**C*erb*-B2 and C-*myc* expression showed association with cell type suggesting differences in cell cycle control between them. C*erb*-B2 was found in greater amounts in spindle cell tumours (n=19) than mixed/epitheliod tumours (n=27). Mean /Rank 28.87/19.72 P= 0.023. C-*myc* was also expressed more by spindle cell tumours. Mean/Rank 21.65/13.31 P= 0.017 (n= 10 spindle, 21 mixed/epith.).**

**A correlation was also found between tumour volume (as measured by B-scan ultrasound measurements ) and ICAM expression with greater expression in large tumours. Mean/Rank 18.37/ 27.84 P=0.016 (n= 23 < 1500mm, 22 > 1500mm). Metastatic disease was only present in 11 patients and no correlation was found between this and any of the surface markers.**

**Flow cytometry was funded by Yorkshire Cancer Research.**

**P156** DETECTION OF CHROMOSOMAL ABERRATIONS IN NEUROBLASTOMA USING COMPARATIVE GENOMIC HYBRIDIZATION (CGH) C.J. Breen*, A. O'Meara and R.L. Stallings, Our Lady's Hospital for Sick Children, Dublin 12, Ireland

Neuroblastoma is the commonest extracranial solid tumour of childhood, with a peak incidence in the two to five year age group. Despite intensive approaches to treatment, prognosis for most patients remains poor. A number of chromosomal and molecular genetic abnormalities (1p deletion, NMYC amplification, partial 17q trisomy) have been associated with aggressive clinical behaviour, identification of which are currently being incorporated into treatment strategies. The recent application of comparative genomic hybridization (CGH) technology has facilitated retrospective analysis of a wide range of tumours with the added advantage of the visualization of the entire genome. The aim of this study was to correlate the above abnormalities with clinical behaviour and assess feasibility of incorporation of this technology into prospective studies. Freshly cryopreserved tumour and/or infiltrated bone marrow were investigated from 14 patients (ranging in age from 3 weeks to 6yrs 4mths) attending the Oncology Dept. at this institution. There were four patients with stage 2 disease, four with stage 3, five with stage 4 and one infant with 4S disease. CGH enabeled clinically significant genetic abnormalities to be detected in 11 out of the 14 tumours, and was less cumbersome than conventional cytogenetics and obviated the need for Southern blotting. While follow up period for some patients is short, adverse genomic alterations such as NMYC amplification, partial trisomy 17q, and 1p deletion, were common in patients with advanced disease while hyperdiploidy was noted in patients with low risk disease. The significance of additional abnormalities detected in these tumours requires further study. These studies expand upon the total number of neuroblastomas analysed by CGH by others (e.g. Lastowska et al., 1997 Genes, Chr. Can. 18:162).

### P157 IDENTIFICATION OF CHROMOSOMAL GAINS AND LOSSES IN PAEDIATRIC MALIGNANT ASTROCYTOMA BY COMPARATIVE GENOMIC HYBRIDISATION

T.J.Warr, S.J.Ward, J.L.Darling and D.G.T.Thomas, University Dept of Neurosurgery, Institute of Neurology, Queen Square, London WC1N 3BG

Astrocytic tumours are the most common central nervous system tumour in children and approximately a quarter of these are malignant. Although these childhood tumours closely resemble their adult counterparts both in location and histological appearance, there is evidence that their pathogenesis is genetically different. Cytogenetic and molecular genetic analyses of paediatric astrocytoma have been unable to detect the genetic changes which are characteristic in the adult tumours. Furthermore, loss of heterozygosity at 17p13.3 and 22q12 are the only novel non-random aberrations to have been described in the paediatric tumours.

We have used comparative genomic hybridisation to identify gain and loss of genetic material across the whole genome in a series of ten paediatric malignant astrocytoma. DNA was extracted from either freshly frozen tumour biopsies (7 cases) or 5µm paraffin embedded sections (3 cases) prior to differential labelling and hybridisation. We have identified gene amplification at 1p11-31, 2q21-24, 7p12, 7q21-32, 8p21.3-24.1, 12q13-pter and 13q11.2 and genetic loss at 6p21.2-21.3, 6q15-16, 9p21, 9q12-21, 10p13-15, 12q24.1-ter, 15q21-22, 17p13 and 22q12. Some of these changes correspond to regions to which some adult astrocytoma oncogenes and tumour suppressor genes are known to map; the regions of amplification on chromosome 7 contain the EGFR and MET genes which have been shown to be independently amplified in adult tumours, the large amplicon on 12 contains the CDK4 and MDM2 genes which map to 12q13; the CDKN2/p16 gene, which is homozygously deleted in 70% of adult glioblastoma, is located at 9p21; and loss at 10p13-15 corresponds with the location of one of the putative tumour suppressor genes on chromosome 10. It is possible, therefore, that there are some common genetic steps in the malignant progression of both paediatric and adult astrocytoma.

### P158 AN INVESTIGATION OF MICROSATELLITE INSTABILITY IN SPECIALISED INTESTINAL METAPLASIA AT THE GASTRO-OESOPHAGEAL JUNCTION

C.M. Gleeson*[1], N.I. McDougall[2], J.M. Sloan[3], J.S.A. Collins[2], S.E.H. Russell[1], [1]Department of Oncology, The Queen's University of Belfast, Departments of [2]Gastroenterology and [3]Pathology, Royal Victoria Hospital, Belfast, N Ireland.

Introduction: Short segments of specialized intestinal metaplasia (SIM) at the gastro-oesophageal junction have been reported in association with oesophageal adenocarcinoma, but the malignant potential of SIM is unknown. Microsatellite instability has been detected in oesophageal adenocarcinoma (Gleeson et al., Cancer Research, 1996, 56:259) and in associated Barrett's oesophagus (Gleeson et al., Genes, Chromosomes & Cancer, 1998, 21:49). This study assessed whether such changes occur in SIM at the gastro-oesophageal junction.

Methods: Patients attending randomly selected open access upper gastrointestinal endoscopy lists were invited to participate in the study. Paired biopsies were taken from the gastric body and from the epithelium immediately distal to the squamocolumnar junction. Samples were assessed for evidence of SIM using H&E and PAS/AB staining of tissue sections. Frozen samples demonstrating SIM were assessed for microsatellite instability.

Results: One hundred and one patients were recruited to the study. In six cases, the biopsy samples contained squamous epithelium and the remaining 95 cases (44 males, 51 females; mean age of 47 years, range 20-87 years) were assessed for SIM. Sixty patients had a normal oesophagus on endoscopy, 11 had hiatus hernia alone, 20 had oesophagitis or oesophageal ulcer, 2 had Barrett's oesophagus and 2 were not available. SIM was detected in 20 patients (21%), two of whom had Barrett's oesophagus on endoscopic assessment and one who had intra-mucosal carcinoma not detected at endoscopy. In 8 of these 20 cases, the frozen sections did not show any evidence of SIM and a further two specimens were not available for genetic analysis. Microsatellite instability was detected in the 2 cases with Barrett's oesophagus. None of the remaining 7 patients with SIM, but normal junctional mucosa, had microsatellite instability.

Conclusions: The incidence of SIM at the gastro-oesophageal junction in randomly selected patients attending our open access centre is similar to that reported previously. The occurrence of SIM is patchy, as demonstrated by the disparity between the parafin and the frozen sections. Finally, we have found evidence of microsatellite instability in patients with Barrett's oesophagus, but not in those with SIM at the gastro-oesophageal junction.

specialized intestinal metaplasia, Barrett's oesophagus, microsatellite instability

### P159 GENOMIC MAPPING OF THE HUMAN *MDM2* ONCOGENE AND THE RELATIONSHIP BETWEEN EXON/INTRON BOUNDARIES AND VARIANT *MDM2* TRANSCRIPTS

H. Liang*, H. Atkins and J. Lunec, Cancer Research Unit, University of Newcastle upon Tyne, NE2 4HH, U.K

The *MDM2* proto-oncogene, which encodes a protein binding the p53 tumour suppressor, has been found amplified, overexpressed and in some cases alternatively spliced in a range of human tumours. Although human *MDM2* cDNA sequence has been reported, the genomic sequence and organisation have not been documented. Since *MDM2* has demonstrated a complex pattern of expression, including alternative splicing and differing transcriptional start sites, a genomic sequence map becomes essential to deciphering this gene's function. To this end, we amplified genomic DNA using long range PCR techniques with primers based on the known cDNA sequence. Genomic PCR products spanning introns were cloned into the pUAg vector (Ingenius) and sequenced by automated and manual methods.

We have now obtained complete PCR products and boundary sequences for all introns. Our data shows that the *MDM2* gene spans approximately 32 kb and is divided into a minimum of 12 exons. Exon sizes range from 71 to greater than 1159 bp. Intron sizes vary from 121 to 7000 bp. All the splice donor and acceptor sites follow the "GT-AG" rule. Comparison with the murine genomic map data indicates sustantial differences in the size of corresponding introns. In addition, exon/intron boundary sequences have disclosed that the variant transcript forms of *MDM2* gene reported previously by Sigalas, et al (Nature Medicine 1996 2: 912-917) are generated by alternative splicing mechanisms. The sequence map has also revealed a promoter-like region in the near 3' end of intron 3 and has led to the investigation of this as a second putative p53-dependent promoter.

### P160 EXPRESSION OF P53, WAF-1, BCL-2, AND BAX IN CENTRAL PRIMITIVE NEUROECTODERMAL TUMOURS (cPNET)

A.S.Y.W.Burns*[1], E. Jaros[2], A.D.J.Pearson[1], R.H. Perry[2] and J. Lunec[1], [1]Cancer Research Unit, University of Newcastle Upon Tyne, [2]Neuropathology, Newcastle General Hospital

We have previously found that strong p53 immunohistochemistry (IHC) staining intensity correlates with poorer patient survival for cPNETs (Jaros et al, BJC, 68, 801-807, 1993). Subsequent analysis of a group of 96 cPNET's has shown that there is a strong association between overall staining intensity and the percentage of stained tumour cells (labelling index, or LI). LI of >20% correlated with poorer survival (p= 0.0165, log-rank test). However, dideoxy-DNA sequencing of the p53 gene (exons 4-8) identified point mutations in only 2/8 cases from the intensely staining group and not in the moderate and negative groups, indicating that p53 point mutations are not frequent in these tumours. The potential of p53 function as a prognostic marker in cPNETs was further explored by IHC staining for downstream protein mediators of p53 functions; p21/Waf-1, Bcl-2 and Bax expression was examined using corresponding antibodies, monoclonals Ab-1(Oncogene-Science) and 124 (Dako), and polyclonal 13666E (Pharmingen). p21/Wafl was weakly expressed (<5%LI), whereas bax was strongly (>40%) expressed in 69% of the tumours, with LI distributed in a distinct bimodal pattern. Bcl-2 was expressed in only 38% of the cases (LI >0 to 95%). Survival did not correlate with p21/Waf-1 expression (p=0.7195). Although higher Bax- or Bcl-2 expression showed a trend for association with poorer survival, this did not reach statistical significance (p=0.1574 and 0.1268, respectively). This might indicate some de-regulation in the Bax downstream pathway. In addition, no correlation was found between p53 overexpression and the expression of these proteins. In conclusion, pre-treatment analysis for these downstream mediators of p53 function did not provide additional prognostic information to analysis of p53 status alone, the significance of which was confirmed in these extended studies.

### P161 VARIABLE REDUCTION IN ALLELE RATIOS OF THE FHIT GENE INDICATES THE PRESENCE OF DIFFERENT CLONES IN SQUAMOUS CERVICAL CANCER

D. Butler*, E.W. Kay, C. Barry Walsh & M. Leader. Dept. of Pathology, Royal College of Surgeons in Ireland, St. Stephen's Green, Dublin 2.

Loss of Heterozygosity (LOH) analysis has revealed a predominance of deletions on the short arm of chromosome 3 in cervical cancers between 3p13 and 3p21.1. It has been postulated that a novel tumour suppressor gene may be located in this region. In 1996 a candidate tumour suppressor gene was discovered at 3p14.2 called the FHIT tumour suppressor gene. The FHIT gene also encompasses the common chromosomal fragile site FRA3B. Human Papilloma Virus (HPV) which is the main aetiological agent in cervical cancers has been found to be able to integrate its genes into the chromosome 3 fragile site deleting a piece of DNA including the FHIT gene. The close association between LOH on chromosome 3, FRA3B disruption and viral integration by HPV makes the FHIT gene an ideal candidate in the tumourgenesis of cervical cancer.

Sixty three cervical LLETZ biopsy specimens were selected from a computerised database of cases in the Pathology Department, R.C.S.I. Tumour and normal cells were microdissected from a total of sixty three cases of preinvasive and microinvasive squamous lesions which comprised 21 CIN1 cases, 24 CIN3 cases and 18 microinvasive cases. CIN3 tumour cells associated with 18 microinvasive cases were also dissected for comparative analysis. DNA was extracted using single step proteinase K digestion. PCR was used to amplify the target area using a fluorescently labelled intragenic microsatellite marker. PCR products were analysed on an automated DNA sequencer using Fragment Manager software to determine allele loss.

Analysis of allele ratios, to determine the degree of allele loss, revealed variable degrees of reduction in allele ratios in tumour versus normal cells. CIN1 lesions showed the lowest percentage of reduction in allele ratios followed by the CIN3 cases and microinvasive associated CIN3 cases. The microinvasive cells had the highest percentage of reduction.

The purity of tumour cells obtained by microdissection indicates the existence of different clones within the same population of tumour cells. Determination of the experimental variation between normal and tumour pairs, may allow the calculation of a cut off point below which LOH can be confirmed. The cause of this phenomenon has yet to be determined but may be related to viral integration events.

### P162 CHARACTERIZATION OF CERVICAL ADENOCARCINOMAS: A COLLABORATIVE SWEDISH AND IRISH STUDY

H. Lambkin[1], B. Skyldberg[2], E. Murray[1], P. Kelehan[3]. [1]Dept. Biological Sciences, Dublin Institute of Technology, Dublin 8. [2]Karolinska Institute, Stockholm, Sweden. [3]National Maternity Hospital, Holles St., Dublin 2.

Paraffin wax-processed tissue samples from forty cases of cervical adenocarcinoma, 20 each from Swedish and Irish hospital laboratories. These samples were evaluated for HPV status by PCR amplification (with two sets of HPV consensus primers), proliferative status (cyclin A and polyclonal Ki-67 antibodies ), p53 protein (p53-DO7 monoclonal antibody) and oestrogen receptor (ID5 monoclonal antibody) expression by immunohistochemistry.

The average paietent age was 44 for the Irish and 61 for the Swedish adenocarcinoma cases. The majority of Irish tumours were HPV-positive while some of the Swedish tumours from the older patients were HPV-negative. These negatives are being further evaluated with type-specific probes. p53-DO7 immunostaining was performed without superheating and one Irish and two Swedish samples expressed high levels of protein (>40% of tumour cells) and five Swedish samples low levels (<10%). Most HPV-negative cases were p53 protein positive.

None of the Irish samples were oestrogen receptor positive although stromal cells were immunopositive in some samples. 63% of the Swedish tumours were high expressors with >50% of tumour cells immunostaining.

No differential was discerned between Swedish and Irish samples for Ki-67 and cyclin A protein expression. The average Ki-67 and cyclin A proliferative indices for Irish tumours was 44% and 25% respectively while for the Swedish tumours values were 47% and 23%.

### P163 EVALUATION OF HISTOLOGICAL FEATURES AND EXPRESSION OF c-erbB-2 ONCOPROTEIN IN CERVICAL CANCER GRADES

D. Butler*, E.W. Kay, C. Barry Walsh & M. Leader. Dept. of Pathology, Royal College of Surgeons in Ireland, St. Stephen's Green, Dublin 2.

The c-erbB-2 proto-oncogene encodes a 185Kd transmembrane glycoprotein with tyrosine kinase activity and which shows 78% homology with the intracytoplasmic domain of EGFR receptor.

Amplification and overexpression of c-erbB-2 has been reported in a variety of tumours predominantly of epithelial origin such as breast, ovary, pancreas and stomach. Membrane staining for c-erbB-2 has been found to be prognostically significant. Few studies have assessed the role of c-erbB-2 overexpression in preinvasive and invasive squamous cervical lesions and none in relation to poor histological features.

83 archival cervical LLETZ biopsies consisting of 27 CIN1 cases, 30 CIN3 cases and 26 microinvasive cases were selected consecutively from a computerised database of cases in the Pathology Department, R.C.S.I. Immunostaining for c-erbB-2 oncoprotein overexpression was performed using an indirect ABC technique on sections 3µm thick. Staining was evaluated for intensity and extent for both cytoplasmic and membrane staining in the CIN1, CIN3 and microinvasive cases.

Haematoxylin and Eosin stained sections from the 83 cases were reviewed for the presence of 6 histological features some of which have been previously shown to be associated with microinvasive cancer namely: extent of surface involvement, extent of glandular involvement, comedo necrosis, squamous maturation, koilocytic change and apoptosis. Univariate and bivariate analysis was used to determine the relationship between overexpression of c-erbB-2 and the presence of adverse histological features.

Membraneous staining for c-erbB-2 oncoprotein was absent in all grades of lesion. Cytoplasmic staining intensity was shown to have a statistical relationship with koilocytic change. All of the histological features examined were shown to have a strong statistical relationship with grade of tumour.

Immunostaining for c-erbB-2 in cervical biopsies may prove to be a useful adjunct to screening detected lesions in association with the recording of certain histological parameters, although the biological significance of cytoplasmic c-erbB-2 overexpression is unknown.

### P164 SEARCHING FOR GERMLINE MUTATIONS IN PROSTATE CANCER

MS Forrest*[1], MA Knowles[1], SM Edwards[2], RA Eeles[2], RA Hamoudi[2], The CRC/BPG UK Familial Prostate Cancer Study Collaborators[2], MD Teare[3], DF Easton[3] and DT Bishop[4]. [1]ICRF Cancer Medicine Unit, St James's University Hospital, Leeds LS9 7TF, [2]Institute of Cancer Research, 15 Cotswold Road, Sutton, SURREY, SM2 5NG, [3]CRC Genetic Epidemiology Unit, Strangeways Research Laboratories, Institute of Public Health, Worts Causeway, Cambridge, CB1 4RN, [4]ICRF Genetic Epidemiology Laboratory, St. James's University Hospital, Leeds LS9 7TF.

Following the recent discovery of the putative tumour suppressor gene, *PTEN/MMAC1/TEP1*, on chromosome 10q23 by mapping of homozygous deletions in tumour cell lines (Li *et al*, Science, 275: 1943, 1997; Steck et al, Nature Genetics, 15: 356, 1997; Li *et al*, Cancer Research, 57: 2124, 1997), a number of studies have examined the frequency of somatic mutations in this gene. Somatic mutations have been found in glioblastomas, melanomas and breast and prostate carcinomas. Germline mutations in *PTEN* have been found in Cowden disease families (Liaw *et al*, Nature Genetics, 16: 64, 1997), Bannayan-Zonana syndrome families (Marsh *et al*, Nature Genetics, 16: 333, 1997) and also some Juvenile Polyposis families (Lynch *et al*, American Journal of Human Genetics, 61: 1254, 1997).

We hypothesise that germline *PTEN* mutations could be important in familial prostate cancer. We have therefore searched the CRC/BPG UK Familial Prostate Cancer Study samples for evidence of *PTEN* mutations. DNA was extracted from peripheral blood taken from 50 prostate cancer families chosen because they contained three or more cases of prostate cancer or related sibling pairs, preferably where one was less than 65 years at diagnosis. First the samples were genotyped at three loci close to *PTEN* and LOD scores calculated to identify families consistent with linkage. Overall there was significant evidence against the hypothesis that familial prostate cancer is due to *PTEN*. Under the best fitting model, a quarter of families were due to a gene in the *PTEN* region but the statistical evidence for any linkage was minimal. DNA from the youngest affected male from each of the 37 remaining families was then amplified by PCR and each of the 9 *PTEN* exons sequenced in both directions. To date no mutations have been found.

### P165 IDENTIFICATION OF PEROXISOME PROLIFERATOR ACTIVATED RECEPTOR α AS AN ANDROGEN RESPONSIVE GENE AND ITS UPREGULATION IN PROSTATIC CARCINOMA.

G.P.Collett*, A.M.Betts, M.I.Johnson, D.E.Neal and C.N.Robson, Dept of Surgery, Newcastle University, Newcastle upon Tyne, NE2 4HH.

Peroxisome proliferator activated receptor α (PPARα) is a member of the nuclear receptor superfamily of ligand activated transcription factors. PPARα is activated by peroxisome proliferators and fatty acids and has been shown to be involved in the transcriptional regulation of genes involved in fatty acid metabolism. In rodents the PPARα-mediated change in such genes results in peroxisome proliferation and can lead to the induction of hepatocarcinogenesis.

In humans PPARα is expressed at high levels in liver, heart and kidney and at lower levels in other tissues. Using the mRNA differential display technique we have shown that chronic exposure of the prostate cancer epithelial cell line LNCaP to the synthetic androgen mibolerone results in the downregulation of PPARα mRNA. Northern blot analysis confirmed that PPARα mRNA levels are reduced to approximately 40% of control levels in LNCaP cells exposed to 10nM mibolerone for 96 hours.

*In situ* hybridisation analysis showed that PPARα expression in prostate is confined to epithelial cells and is upregulated in prostatic carcinoma. In benign prostatic tissue PPARα mRNA was either absent or weakly expressed in the basal epithelial cells whereas in high grade prostatic carcinoma there was strong expression in tumour cells.

These results suggest that PPARα may play a role in the development and progression of prostatic carcinoma.

### P166 Prostate Specific Gene Expression: Characterisation of Promoter regions from genes expressed in human prostate

G. Quinn, B. McDonald, M. Nitert, M Sharrard and N. J. Maitland

YCRC Cancer Research Unit, Dept of Biology, University of York, YO1 5DD, UK

The availability of control sequences which define prostate specificity should offer an increased level of control over the expression of therapeutic genes in proposed gene therapy protocols for prostate cancer.

**Keywords**: Promoter, Prostate Cancer, Specificity

### P167 *PTEN* MUTATION ANALYSIS IN BLADDER CANCER

J.S. Aveyard and M.A. Knowles, ICRF Cancer Medicine Research Unit, St James's University Hospital, Beckett St, Leeds LS9 7TF

Loss of heterozygosity (LOH) at 10q22-23 has been identified in a significant proportion of invasive bladder tumours (Cappellin et al., Oncogene, 14: 3059, 1997). Recently, the *PTEN/MMAC1* gene was identified as a candidate tumour suppressor within this region. Mutations of *PTEN* have been found in several other tumour types including glioblastomas, carcinomas of prostate, breast endometrium and melanoma. We have carried out LOH analysis of 10q and single strand conformation polymorphism (SSCP) analysis of *PTEN* to determine its potential involvement in bladder cancer.

We assessed 63 invasive (≥pT2) and 61 superficial (pTa/T1) transitional cell carcinomas for LOH at three microsatellite loci (*D10S541, D10S215, D10S1765)* close to *PTEN*. LOH/allelic imbalance was found in 17/49 informative invasive tumours and 4/61 superficial tumours indicating a significant association of 10q LOH with disease progression. Primers were designed to amplify the entire coding region of *PTEN* in fragments of <320bp and SSCP analysis was carried out on genomic DNA from 63 invasive tumours including all 17 with LOH/allelic imbalance. Band mobility shifts were identified in 13 tumours. Three of these in exon 1 appear to be normal sequence polymorphisms. Sequencing of the other suspected mutations is in progress.

### P168 FUNCTIONAL ANALYSIS OF *PTEN* IN BLADDER CANCER.

*Amy Skilleter, Joanne S. Aveyard and Margeret A. Knowles.
ICRF Cancer Medicine Research Unit, St. James's University Hospital, Beckett Street, Leeds. LS9 7TF

The putative tumour-suppressor gene *PTEN*, located on chromosome 10q22-23, has been found to be involved in several different types of cancer including brain, breast endometrial and prostate. It has also been implicated in bladder cancer (see abstract by Aveyard and Knowles).

The function of *PTEN* is yet to be fully determined, although it has extensive homology with tensin and auxillin (cytoskeletal proteins involved in cell motility) and also possesses a functional dual-specificity tyrosine phosphatase region. It has been postulated that this may provide *PTEN* with its tumour suppressor function by counteracting an oncogenic kinase. The purpose of this current study is to assess the status of *PTEN* in bladder cancer cell lines and to explore its function by gene replacement experiments.

Duplex PCR reactions have shown one cell line, UM-UC-3, to have a homozygous deletion of the entire *PTEN* gene. Subsequent analysis by Western blotting has confirmed that this cell line produces no PTEN protein. A panel of 16 bladder cell lines is currently being screened for mutations using SSCP analysis.

The lack of a functional PTEN protein in UM-UC-3 cells makes it a good candidate for transfection with an exogenous *PTEN* gene. Initial transfections with a construct containing the entire *PTEN* cDNA in the mammalian expression vector, pcDNA3 are in progress. Similar constructs under control of inducible promoters are planned.

### P169 IDENTIFICATION AND METHYLATION BASED SILENCING OF A GENE(*DBCCR1*) WITHIN A CANDIDATE BLADDER TUMOUR SUPPRESSOR REGION AT 9q32-33

T. Habuchi, *J. Coulter and M.A. Knowles, ICRF Cancer Medicine Research Unit, St James's University Hospital, Beckett St, Leeds, LS9 7TF.

*Introduction:* Loss of heterozygosity (LOH) on chromosome 9q is the most frequent genetic alteration in transitional cell carcinoma of the bladder, indicating the presence of one or more relevant tumour suppressor genes. We have mapped one of these to 9q32-33 and localised the candidate region within a single 840kb YAC (Habuchi, T., Yoshida, O. and Knowles, M.A. Hum Mol Genet 6: 913, 1997)
*Methods* : We isolated cDNA clones for ESTs mapped to the critical YAC 852e11 and carried out 5' RACE to ensure that full length cDNA sequence was obtained. Inton-exon boundaries were identified by vectorette PCR. Single strand conformation polymorphism (SSCP) analysis was used to screen for mutations. Methylation analysis of 2 BssHII sites was carried out by Southern blotting. To assess the role of methylation in gene silencing, cultured cells were treated with the demethylating agent 5-aza-2'-deoxycytidine for 4 days before RNA extraction for RT-PCR analysis.
*Results:* The critical region of deletion is contained within the YAC clone 852e11 which is predicted to contain a bladder tumour suppressor gene designated *DBC1* (for Deleted in Bladder Cancer gene 1). Two ESTs mapped to this YAC were examined. Clones for one of these contained no open reading frame and 5'RACE did not extend the sequence into an open reading frame. The second, IB3089, identifed a human foetal brain cDNA clone with an insert of 1706bp containing an open reading frame with a predicted product of 761 amino acids. This gene has been designated *DBCCR1* (Deleted in Bladder Cancer Chromosome Region candidate 1) and shows no DNA or protein sequence homology with known genes or domains. Mutation analysis detected no mutations in bladder tumour DNAs. Although the gene was expressed in multiple human tissues including urothelium, mRNA expression was absent in 5 of 10 bladder cancer cell lines. Methylation analysis of the CpG island at the 5' region of the gene and the induction of *de novo* expression by a demethylating agent indicated that this island is a frequent target for hypermethylation in bladder cancer.
*Conclusions:* A novel gene *DBCCR1* has been identified within a critical region of deletion in bladder cancer at 9q32-33. Although no somatic mutations have been detected in bladder tumours, gene silencing by hypermethylation in both bladder tumours and cell lines makes this a good candidate for *DBC1*.

### P170 DELETIONS AT 9q34 IN BLADDER CANCER INVOLVE THE *TSC1* LOCUS

*N.J. Hornigold, *A. Davies and M. A. Knowles, ICRF Cancer Medicine Research Unit, St James's University Hospital, Beckett St, Leeds, LS9 7TF.

*Introduction :* Deletions of chromosome 9 occur in the majority (>50%) of human bladder cancers. There is now evidence that loss of heterozygosity (LOH) involves at least 3 regions on 9q suggesting that multiple chromosome 9 tumour suppressor genes may contribute to the development of bladder cancer.
*Methods :* We have used microsatellite based deletion mapping to pinpoint the critical region of LOH at 9q34. Single strand conformation polymorphism (SSCP) analysis was used to screen for mutations in the *TSC1* gene followed by direct sequencing of bands with altered mobility.
*Results* :LOH analysis at 17 microsatellite loci in 9q34 in a panel of 102 bladder tumours identified 6 tumours with small deletions involving 9q34 only. The minimum region was between the loci *D9S149* and *D9S66*. This region contains the tuberous sclerosis gene *TSC1*. *TSC1* is predicted to act as a tumour suppressor gene since deletion of the wild type allele is detected in hamartomas which develop in individuals carrying a germline mutation. *TSC1* is therefore a candidate 9q34 bladder tumour suppressor. We have carried out a mutation screen of *TSC1* in the 6 tumours with discrete 9q34 deletions and in 31 tumours with LOH of the entire chromosome arm. Primers flanking*TSC1* exons were used to amplify the entire coding sequence in 19 fragments for analysis. Several bands with altered mobility have been identified in bladder tumour DNA. Sequencing of these is in progress.
*Conclusions* : A critical region of LOH has been mapped to 9q34 in bladder cancer. This region contains the recently identified *TSC1* gene, a putative tumour suppressor. Preliminary SSCP analyses indicate that some bladder tumours contain somatic alterations to *TSC1*. The nature of these awaits confirmation by DNA sequencing.

### P171 MOLECULAR GENETIC SCREENING FOR URINE DETECTION OF BLADDER CANCER

**J.M.S. Bartlett[1,3]*, M.A. Underwood[1], A White[3], C.S. Stuart[2] & A.M. McNicol[2]. [1]Glasgow University Dept Surgery, [2]University Dept Pathology, GRI, [3]DNASHEF Technologies, Dept Haematology, RIE.**

Urine cytology is routinely used to assess bladder cancer (TCC) recurrence despite poor correlation between tumour presence and detection of malignant cells in urine. Recently it has been suggested that microsatellite marker analysis of cells from patient urine provides a significant improvement on urine cytology (Sidransky et al, 1997). However, this technique may have limitations in detection of superficial lesions which make up the majority of recurrent tumours. We screened 16 patients with clinically identified superficial TCC using 60 microsatellite markers with an ABI 377 automated DNA analysis system. 13/16 patients demonstrated pathologically diagnosed TCC (11 pTa & 2 pT1 tumours), one each of remaining patients had CIS, Cystitis glandularis or no detectable tumour. 8/13 cytology reports available to date demonstrated the presence of malignant cells. Urine DNA was obtained and extracted from 11/14 tumour bearing patients to date. 3 Patients failed analysis due to insufficient cells for DNA extraction (all pTaG1). Up to 52 microsatellites spanning chromosomes 7, 8, 9, 11, 17 & 18 were tested, with matched patient blood DNA as a control.
LOH was detected at 13/408 loci (3%) below rates detected in previous studies of displastic tissues. No LOH has been found to date in 4 pTaG1 tumours (0/52, 0/18, 0/43 & 0/49 markers respectively), or in a benign polyp. LOH was detected in 7 patients: chromosomes 9 (2 pats 3 & 4 loci, pT1G1, pTaG1), 17 (3 patients), 18 (3 patients 2 with LOH chromosome 17), 8 (1 patient) & 11 (1 patient). The cystitis glandularis patient has LOH on chromosomes 17 & 18.
LOH was detected in 7/14 patients with pTa 1 tumours (50%). 3 patients (21%) failed analysis due to lack of cells in urine whilst in 4 patients (29%) no LOH has yet been detected. One benign polyp showed no LOH whilst a patient with a significant preneoplastic lesion did show detectable LOH. The relatively low success rate of "molecular cytology" suggests that this technique may not be readily applicable to detection of superficial (pTa) bladder tumours. Expansion to further markers on chromosomes 4, 13 & 20 may provide additional data but the low rate of detection of LOH on chromosome 9 (altered in up to 60% of TCC) found here (14%) may suggest that tumour LOHs may not be detectable in urine. Although unlikely to alter management of recurrent TCC patients, the ability to rapidly screen multiple microsatellites demonstrated here may nonetheless provide additional information in the screening of high risk populations (petrochemical workers etc) or improve detection of upper tract TCCs.

### P172 EVALUATION OF THE YEAST FUNCTIONAL (FASAY) ASSAY FOR DETECTING *p53* MUTATIONS IN BLADDER CANCERS.

W. Scaife*, K. Braithwaite, R. Abdel-Fattah, D.E. Neal & J. Lunec. Cancer Research Unit, The Medical School, University of Newcastle upon Tyne, NE2 4HH.

In general, patients suffering from transitional cell carcinoma (TCC) of the bladder who demonstrate mutations in *p53* have been shown to incur a significantly increased rate of developing tumour metastases and dying of the disease compared to patients with TCC who have no evidence of *p53* mutations (*e.g.* Esrig, D. *et al.*, 1994. *N. Engl J Med* **331**, 1259-1264). However, the significance of *p53* mutations with respect to responses to chemotherapy by patients with TCC is currently the subject of much debate (Cote, R. J. *et al.*, 1997. *Nature* **385**, 123-124).

In this investigation, the *p53* status of tumour samples of varied stage and grade was analysed by a combination of yeast functional (FASAY) assay and both direct genomic DNA sequencing and automated sequencing of rescued recombinant plasmids, and comparisons made for an initial group of 18 tumour samples. The FASAY method detects mutations of *p53* RNA between codons 67 and 347 on the basis of loss of transcriptional activity of the protein. Yeast are co-transfected with *p53* RT-PCR products and a gapped expression vector allowing homologous recombination *in vivo* to yield a percentage of red colonies which reflects the proportion of mutant PCR products. The assay was performed employing total RNA in a slight modification of the method previously described (Flamen, J.-M. *et al.*, 1995. *PNAS* **92**, 3963-3967). Recombinant plasmids were recovered from yeast clones, transformed *into E. coli* DH5$\alpha$ cells by electroporation and then subjected to automated sequencing.

The method was demonstrated to be reproducible in that multiple assays performed on individual samples yielded very closely correlating percentages of positivity. Samples exhibiting high percentage positivities were generally those shown to possess *p53* mutations by direct sequencing. In all cases for which mutations were detected by direct PCR-based sequencing from genomic DNA (5/18 (27%)), the presence of functionally defective *p53* and detection of the same mutations was confirmed by the FASAY method. In an additional 5 cases, mutations were detected, by the FASAY method and subsequently confirmed by sequencing of the rescued plasmids, which had not been previously detected by direct genomic DNA sequencing. These results indicate that the FASAY method is a reliable technique which is more sensitive than direct genomic DNA sequencing for the detection of *p53* mutations and, in addition, provides information on the functional status of *p53*.

### P173 THE USE OF p53 GENE MUTATIONS IN BLADDER WASHING SAMPLES AS MARKERS FOR BLADDER CANCER.

H.A.Phillips*[1,2], G.C.W.Howard[2], W.R.Miller[1], [1] Dept of Clinical Oncology, University of Edinburgh, [2] NHS Dept of Clinical Oncology, Western General Hospital, Edinburgh, EH4 2XU.

Bladder cancer is a common clinical problem accounting for approximately 5000 deaths per annum in the U.K.. Diagnosis and follow up rely heavily on cystoscopy and biopsy, an invasive procedure performed under general anaesthetic. The advent of Polymerase Chain Reaction (PCR)-based technologies opens up possibilities for the development of sensitive assays to test for abnormal cancer-derived genetic material. These may be used on samples obtained by less invasive techniques.

We have demonstrated previously, using cancer cell lines, that under optimal conditions, Single Stranded Conformational Polymorphism (SSCP) is capable of detecting a mutation in the p53 gene when mutation carrying cells comprise between 1 and 5% of the sample (Phillips H.A. et al., 1996, Br J Cancer, 73, suppl XXVI, 45).

In this study we examined the feasibility of using p53 mutations (the most common somatic genetic aberrations in bladder cancer) as a marker for the presence of malignancy in bladder wash specimens.

DNA extracted from 31 bladder wash samples from 27 patients with bladder cancer was screened for the presence of mutation in exons 5-8 of the p53 gene. Abnormal SSCP appearances consistent with the presence of mutation were detected in 5 samples obtained from 5 separate patients ( 1 in exon 8 and 2 each in exons 6 and 7 ). In each case the same abnormal SSCP pattern was detected in samples of the tumour obtained by surgical biopsy. In 3 of the 5 samples the mutation was confirmed by direct sequencing. In the remaining 2 cases sequencing was not possible due to technical difficulties but the same abnormal SSCP pattern was apparent in the primary tumour and duplicate samples of the washing, findings highly suggestive of the presence of mutation.

These results demonstrate that it is feasible to detect the presence of malignancy in shed tumour material in the form of a bladder washing by the detection of the same p53 mutation. Such methodologies offer the potential to enhance the diagnosis and follow up of patients with bladder cancer using samples obtain by less invasive methods than current practice require.

### P174 DISTRIBUTION OF THE ANTIGEN RECOGNISED BY THE MONOCLONAL ANTIBODY P1H10 IN OVARIAN AND BREAST TISSUE.

Y. McGarry[1], S.Lewis[1], A. McCann[2], E. Moran[3], S. K. Sinha[4], P. Kelehan[4], and P. Dervan[2]. [1]Toxicology Unit, Athlone Institute of Technology, [2]Biotechnology Centre, University College Dublin. [3]National Cell & Tissue Culture Centre, Dublin, [4]National Maternity Hospital, Dublin, Ireland.

Altered expression of several gene products has been associated with multidrug resistance (MDR) in cancer. We have previously suggested that a monoclonal antibody (MAb), P1H10, raised to the ovarian cancer cell line, OAW42, may be detecting an antigen with a possible role in MDR[1]. The antigen, with a molecular mass of 48 kDa identified by Western Blotting, was differentially expressed in selected carcinoma cell lines but was not detected by enzyme-linked immunoabsorbent assay and immunocytochemical studies in the normal fresh or frozen material investigated[1].

In the present study, the distribution of the antigen recognised by the antibody P1H10 in a range of routinely fixed paraffin wax-embedded tissue samples of 23 ovarian and 46 breast tissue samples was investigated by immunohistochemistry. Of the ovarian samples, there was no staining in 3 normal or 10 benign (4 serous/6 mucinous) tissue sections. Strong positive staining which was confined to the corpus luteum was detected in 1 out of 4 borderline mucinous tumours. No staining, however, has been detected in normal corpus luteum. 1 out of 6 epithelial tumours showed intense staining with MAb P1H10. This tumour was histologically characterised as a poorly differentiated endometrioid adenocarcinoma of the ovary with clear cell change and advanced metastates. There was histiocytic immunoreactivity in some of the tumours.

The majority of the normal breast tissue sections (17 out of 20) had negative staining on immunohistochemical analysis with MAb P1H10. Staining on the remaining sections only occurred in macrophages and in isolated ducts associated with possible apocrine change. The antigen expression in 11 patients with fibrocystic disease and 12 patients with fibroadenomas was similar to the normal group except for some cytoplasmic immunoreactivity in selective ducts in all cases and two fibroadenomas with strong staining. One of these sections with predominantly stromal proliferation had strong ductal staining. There was intense cytoplasmic staining in 3 out of 3 poorly differentiated ductal carcinomas.

These results indicate that a recently characterised MAb, P1H10, identifies an antigen differentially expressed in a number of carcinoma cell lines which was either not detected or weakly expressed in normal or benign ovarian and breast tissue. Further studies are continuing to establish the antigen distribution in a larger cohort of malignant tissue and to further characterise the antigen recognised by the antibody.

1. McGarry Y. Moran E. Hallahan C. Clynes M. Tipton K. *J Exp Ther Oncol* 1996;1:260

### P175 EXPRESSION OF A 'NOVEL' TUMOUR-ASSOCIATED CELL SURFACE GLYCOPROTEIN pMQ1 IN HUMAN BREAST CANCER.

LJ Fon*[1], K Mulligan[1], TF Lioe[2], RAJ Spence[1], PG Johnston[1], D McCormick[1]. [1]Dept of Oncology and [2]Dept of Histopathology, Queens University of Belfast, Belfast City Hospital Belfast, BT9 7AB

A monoclonal antibody has recently been isolated in this laboratory that recognises an epitope on a glioma-associated glycoprotein (pMQ1) with characteristics suggestive of an adhesion molecule or receptor. It has a cell surface distribution and immunocytochemistry and flow cytometry have demonstrated that in astrocytic tumour cells the level of expression increases with tumour grade. N-terminal sequencing has shown that pMQ1 is a previously undescribed mammalian protein with significant sequence identity with a microorganism gene product implicated in cell adhesion. The finding that this protein is present in many secondary tumours in brain prompted the current immunohistochemical survey of the expression of pMQ1 in breast tumours.

Immunohistochemical analysis was carried out on archival, paraffin-embedded sections from 124 breast biopsy specimens (114 breast carcinomas, 5 fibroadenomas and 5 cases of fibrocystic disease). The immunostaining was independently assessed by two observers who noted the distribution of staining and scored the intensity of labelling on a scale of 0-3. The results were correlated with the patient's age, tumour size, histological grading, lymph node status and Nottingham Prognostic Index (NPI).

### P176 QUANTITATION OF EGFR and c-*erb*B-2 EXPRESSION IN PREINVASIVE COMPARED TO INVASIVE BREAST CANCER.

*D. Chong, J. Reeves, T. Cooke, [1]W.D. George, [1]E. Mallon, [2]B. Ozanne and P. Stanton, University Department of Surgery, Glasgow Royal Infirmary, [1]University Department of Surgery, Western Infirmary, Glasgow and [2]Beatson Institute for Cancer Research, University of Glasgow.

EGFR and c-erbB-2 are being explored as therapeutic targets in breast cancer. This approach will be enhanced if it is effective in pre-invasive as well as invasive disease. We have found that EGFR is downregulated and c-erbB-2 is overexpressed in 95% of invasive breast cancers, but little is known about the role of these factors in *in situ* disease.

In frozen sections of invasive cancers with evidence of ductal carcinoma *in-situ* (DCIS), EGFR (n=46) and c-*erb*B-2 (n=40) receptor levels were assayed quantitatively using a radiolabelled antibody method. Numbers of receptors were determined by comparison with cell lines of known receptor density and compared in each element of the tumour.

EGFR and c-*erb*B-2 expression each varied by a factor of several thousand. The frequency distributions for expression of both factors were comparable in DCIS and invasive tumours (Mann-Whitney U test, EGFR p=0.41, c-erbB-2 p=0.56). Within each tumour, there was no significant difference in expression of c-erbB-2 or EGFR in the DCIS and invasive components (Wilcoxon Signed Rank Test, EGFR p=0.419, c-*erb*B-2 p=0.343).

These data suggest that alterations in type I growth factor receptors occur before progression of *in-situ* disease to invasive cancer. High levels of c-*erb*B-2 overexpression in both *in-situ* and invasive areas suggest that the c-*erb*B-2 product is a potential therapeutic target for the treatment of breast cancer at an early stage.

### P177 QUANTITATION OF C-*erb*B-2 IN PRIMARY BREAST CANCER ALLOWS IDENTIFICATION OF A FURTHER POOR PROGNOSTIC GROUP.

*D Chong, J. Reeves, T Cooke, [1]W.D George, [1]E Mallon, [2]B Ozanne and P. Stanton, University Departments of Surgery, Glasgow Royal Infirmary, [1]Western Infirmary, Glasgow and [2]Beatson Institute for Cancer Research, University of Glasgow

C-*erb*B-2 amplification or high levels of overexpression are found in about 25% of breast cancers and these patients have a poor outcome. Using a quantitative radioimmunohistochemical method in frozen sections we have previously shown that c-*erb*B-2 is nearly always overexpressed with gene amplification accounting for a population with very high levels of expression.

Here we report on the application of radioimmunohistochemistry to measure the c-*erb*B-2 protein in a larger set of cases (n = 182) with followup exceeding 5 years. Disease specific survival was assessed using Kaplan Meier life table analysis and log rank tests.

We found that 88% of tumours overexpressed c-*erb*B-2 compared to normal breast. 23% had greater than 20 times normal expression, which we have previously shown, indicates amplification at this locus. These cases had a significantly poorer survival than the rest of the overexpressors ($p<0.0001$). 12% of cases had lower than normal c-*erb*B-2 expression and these patients also had a poorer prognosis than the non-amplified overexpressors ($p<0.0001$).

Quantitative estimations of c-*erb*B-2 in frozen sections allow measurements of the protein in nearly all cases whereas paraffin section immunohistochemistry provides subjective data only the highest expressing cases. Tumours with down regulated c-*erb*B-2 have as poor a prognosis as those with gene amplification levels of the protein.

### P178 CHROMOSOME 11q13 AMPLIFICATION IN HIGH GRADE DCIS DETECTED BY CGH.

H. Magee*[1], E. Moore[1], J. Coyne[2], T. Gorey[3], and P.A. Dervan[1]. Dept. of Pathology[1] and Surgery[3], Mater Hospital and University College Dublin, Ireland and Dept. of Pathology[2], Withington Hospital, Manchester, U.K.

For a variety of technical reasons it is rarely possible to study cytogenetic abnormalities in DCIS, using traditional techniques. However, by combining molecular biology and computerised image analysis techniques it is now possible to carry out cytogenetic analyses on formalin-fixed, paraffin-embedded tissue, using Comparative Genomic Hybridisation (CGH). The purpose of this study was to identify the prevalence of chromosomal amplifications and deletions in high grade DCIS, and to look specifically for unique or consistent abnormalities in this preinvasive cancer.

We examined 23 cases of screen-detected high grade DCIS using a combination of tumour microdissection from histology slides and CGH. All cases showed chromosomal abnormalities. A wide variety of amplifications and deletions were spread across the genome. . The most frequent changes were gains of chromosomes 17 (13 of 23), 16p (13 of 23), 20q (9 of 23) and amplifications of 11q13 (22 of 23), 12q 24.1-24.2 (12 of 23), 6p21.3 (11of 23) and 1q31-qter (6 of 23). The most frequent deletions were on 13q 21.3-q33 (7 of 23), 9p21 (4 of 23), and 6q16.1 (4 of 23).

This is the first systematic cytogenetic study of high grade DCIS, carried out on mammographic screen-detected cancers. Our findings indicate that high grade DCIS is, from a cytogenetic viewpoint, an advanced lesion. There was no absolutely consistent finding in every case. However, we found an amplification of 11q13 in 22 of the 23 cases. The significance of this is unknown at present. It is likely that many of our findings are the result of accumulated chromosomal abnormalities, reflecting an unstable genome in established malignancy.

### P179 NUCLEAR EXPRESSION OF THE WILMS' TUMOUR SUPPRESSOR GENE IN DUCTAL CARCINOMA IN SITU OF THE BREAST.

A. Fabre*[1], H. Magee[1], A. McCann[1], G. Keating[1], T. Gorey[3], P. Dervan[2]. [1]Biotechnology Centre, U.C.D[2] Dept. of Pathology, [3]Dept. of Surgery, Mater Hospital, Dublin, Ireland.

The Wilms' tumour suppressor gene (WT1), located on chromosome 11p13, encodes a nuclear transcription factor involved in the regulation of the insulin-like growth factors and transforming growth factors pathways. As both systems have been found to be deregulated in breast tumourigenesis, we undertook a study to determine the nuclear expression of WT1 in various grades of ductal carcinoma in situ (DCIS) of the breast, a pre-invasive malignancy of the terminal duct epithelium.

Using a commercially available antibody to WT1 (WT1 C-19, Santa Cruz Biotechnology, USA), standard avidin-biotin coupled immunocytochemistry with pressure cooker heat pre-treatment, was performed on 37 cases of DCIS of formalin fixed paraffin embedded tissue (FFPE). 51% (19/37) had an adjacent invasive component.

The DCIS was graded[1] and categorised into a high grade (20/37) and a non high grade (intermediate and low, 17/37) groups.

Nuclear staining for WT1 stratified into negative(-), <50% positive (+), >50% positive, indicated that:

1) There was no correlation between nuclear staining phenotype and the presence of an adjacent invasive component ($p=0.7308$, $\chi^2$ for trend)

2) There was a trend for pure DCIS cases with (-)/<50%(+) to be of higher grade.

Of the many technical approaches employed to evaluate the use of WT1 C-19 in FFPE tissue, pressure cooker heat pre-treatment happened to be optimal. However, despite statistical analysis of grade, nuclear staining and the presence of an adjacent invasive component, this study gave little information concerning the relevance of WT1 in early breast cancer by immunocytochemistry.

{1}: Consensus Conference on the classification of Ductal Carcinoma in Situ. 1997. Human Pathology. 28(11):p.1221-1225

### P180 MITOGENIC ACTIONS OF HEREGULIN ISOFORMS AND TGF-α IN OVARIAN CANCER MODELS

L.M.R. Gilmour, K.G. Macleod, W.R. Miller, J.F. Smyth and S.P. Langdon, ICRF Medical Oncology Unit, Western General Hospital, Edinburgh EH4 2XU.

Increased expression of certain members of the c-erbB receptor family (EGF receptor, erbB2, erbB3 and erbB4) has been linked with poor prognosis in ovarian cancer suggesting that these receptors are implicated in the growth and progression of this disease (Simpson BJB et al, Int J Cancer 64: 202-206, 1995). The EGF receptor is activated by TGF-α while erbB3 and erbB4 are activated by the heregulins (HRGs) ; upon activation these receptors dimerise with each other and also erbB2 and initiate intracellular signalling. The HRG family exists as splice variants and includes HRG-α and HRG-β (Holmes WE et al, Science 256: 1205-1210, 1992).

In the present study, we have investigated the effects of HRG-α, HRG-β and TGF-α on the growth of a range of ovarian cancer cell lines. HRG-α ($10^{-9}$M) and HRG-β ($10^{-9}$M) produced growth stimulation in 6 cell lines, inhibition in 1 line and had no effect in 3 cell lines. TGF-α ($10^{-9}$M) produced growth stimulation in 5 cell lines and had no effect in 3 others. The magnitude of growth stimulation was greatest in the order, HRG-β > TGF-α > HRG-α. RT-PCR demonstrated mRNA expression of EGF receptor, erbB2 and erbB3 in 10 / 10 cell lines and erbB4 in 5 / 10 cell lines. mRNA for TGF-α was detected in 10 / 10 cell lines and HRG (either isoform) in 8 / 10 cell lines. Western blot analysis with the antibodies erbB2 (CB11), erbB3 (RTJ2) and erbB4 (Ab2 Neomarkers) showed a range of receptor levels consistent with RT-PCR data. Cell lines which were growth stimulated by the HRGs expressed moderate levels of erbB2 in conjunction with moderate levels of erbB3 and or erbB4. Heterodimerisation was demonstrated by the observation of tyrosine phosphorylation on erbB2 after activation with all 3 of the above ligands in one of these cell lines.

These data demonstrate that both the HRGs and TGF-α are mitogenic in many ovarian cancer models and are co-expressed with their receptors ; they may therefore regulate growth in an autocrine manner.

### P181 MICROSATELLITE ANALYSIS OFCHROMSOME 7 IN OVARIAN CANCER IDENTIFIES 7q31 AS A REGION FREQUENTLY DELETED IN BENIGN DISEASE.

Martin. R.J.L*., Hurlstone. A., Black. D.H., Hickey.I., and Russell. S.E.H. Department of Oncology. [R.M., S.E.H.R.]. School of Biology and Biochemistry. [I.H]. The Queen's University of Belfast. N.Ireland. U.K. Beatson Institute for Cancer Research, Glasgow, Scotland, U.K. [D.B., A.H.].

The inactivation of multiple tumour suppressor genes is known to play an important role in the development of ovarian cancer. One strategy for locating putative tumour suppressor genes is to analysis tumour DNA and matched blood samples for high rates of loss of heterozygosity (LOH). The aim of this study was to carry out LOH analysis of chromosome 7q using twelve microsatellite markers on a panel of sixty-five ovarian tumours. The microsatellites included four novel CA.GT dinucleotide repeat markers from the D7S522-GATA44F09 interval. Overall 48% of the tumours exhibited LOH at one or more of the markers assayed. These losses spanned tumour stage and histological subtype. Deletion of the entire chromosome arm was not observed in any samples. The highest rate of loss (42%) was observed at the marker CFTR on 7q31.3. Twenty-five of the tumours examined were benign. LOH was greatest in these at CFTR (9/19 informative tumours, 47%) and then at GATA44F09 (6/19 i.e. 32%). In the group of 32 malignant tumours, the highest rate of LOH was at D7S522 (11/18, 61%), and then at 724CA (13/25), D7S633 (9/18, 50%), and CFTR (10/23 tumours, 44%). Overall seven tumours were identified with partial deletions in the vicinity of CFTR. These findings provide strong support for the existence of a putative tumour suppressor gene on 7q31. Further more the high level of deletions observed in benign and early stage disease indicates that this inactivational event occurs early in the development of sporadic ovarian carcinomas.

### P182 CHARACTERISATION OF CHROMOSOME 17 IN ELEVEN HUMAN OVARIAN EPITHELIAL CARCINOMA CELL LINES.

*[1]Keilty, G.W., [2]Cranston, A.N., [1]Harkin, D.P., [1]Church, S.W., [1]Fallows, S., [3]Langdon, S.P., [1]Russell, S.E.H., and [2]Hickey, GI. [1]Department of Oncology, [2]School of Biology and Biochemistry, Queen's University of Belfast, UK., [3]ICRF, Western General Hospital, Edinburgh, UK.

Chromosome 17 harbors over two dozen related genes involved in various types of cancer, neurological disorders and birth defects. Of those involved in cancer, many are significant in ovarian disease.

In this study we have characterised chromosome 17 in eleven human ovarian epithelial carcinoma cell lines ; PEO14, PEO23, TO14, PEA1, PEA2, OAW42, OTN14, A2780, PEO1, PEO4 and PEO6. We describe chromosome 17 translocations, p53 mutations and allelic status of the cell lines.

Cytogenetic analysis by FISH with a chromosome 17 painting probe has shown the presence of complex chromosomal rearrangements. The majority of translocations observed involved the telomeric region of chromosome 17.

The status of the tumour suppressor gene p53 has been determined by immunocytochemistry, SSCP and sequencing analysis. Our results indicate that mutation of p53 shows no association with chromosome 17 translocations.

Mutation of p53 is often associated with loss of an entire chromosome 17 homologue. We provide evidence that cell line p53 mutation and loss of an entire copy of chromosome 17 parallels results described in sporadic ovarian epithelial tumours.

### P183 RESTRICTION MAPPING OF A P1, BAC CONTIG COVERING A REGION ON DISTAL CHROMOSOME 17Q SHOWING HIGH LOH IN SPORADIC OVARIAN TUMOURS

*Burrows, J.F.[1], Lunny, C.[1], Petty, E.M.[3], Kalikin, L.M.[3], Hickey, I[2], and Russell, S.E.H.[1]. [1] Department of Oncology, [2] School of Biology and Biochemistry, The Queen's University of Belfast, UK. [3] Department of Internal Medicine, University of Michigan Medical School, Ann Arbor, MI, 48109

The identification of areas showing a high rate of loss of heterozygosity (LOH) between matched control and tumour DNA's has been widely used to map tumour suppressor genes in sporadic cancers. There have been numerous regions of high rates of LOH found on chromosome 17 in sporadic epithelial ovarian tumours. Whole chromosome loss is common, but we have identified tumours with partial losses on distal chromosome 17q. We have observed such losses in benign, borderline and malignant tumours of all histological subtypes. Fine deletion mapping has reduced this area to a region on 17q25 centred around four polymorphic markers which show zero recombination. We have established a BAC and P1 contig of this entire region. This contig spans ~ 200kb and is made up of 3 P1 clones of ~80kb, ~40kb and ~70kb and 1 BAC clone of ~110kb. The largest P1 clone and the BAC clone span the entire region on their own with an overlap of ~5kb. We have now established a Not1, Sal1 restriction map of this region. This has allowed us to order the markers D17S1790—D17S937—D17S939—Afm203wc5. We are now proceeding with the cloning of transcripts from this region.

### P184 INTERACTION OF OESTROGEN AND c-erbB2 SIGNALLING PATHWAYS IN OVARIAN CANCER CELLS.

P. Mullen*, K. Macleod, G.J. Rabiasz, J.F. Smyth and S.P. Langdon, ICRF Medical Oncology Unit, Western General Hospital, Edinburgh EH4 2XU.

Oestrogen has been shown to increase tyrosine phosphorylation of several proteins, including c-erbB2, in human cancer cells under conditions of growth stimulation (Migliaccio et al, 1993, Oncogene, 8, 2183; Matsuda et al, 1993, PNAS, 90, 10803). However, over-expression of c-erbB2 may also confer oestrogen resistance in oestrogen receptor (ER) positive cells (Pietras, et al, 1995, Oncogene, 10, 2435). The interaction of these pathways has not previously been investigated in ovarian cancer.

In the present study, we have investigated the relationship between oestrogen-mediated growth stimulation and c-erbB2 activation in four ovarian cancer cell lines. PE01, PE04, SKOV-3 and PE014 cell lines (ER concentrations of 96, 112, 66 and 0 fmol/mg respectively) were treated with 17β-oestradiol ($E_2$) ($10^{-10}$M) for 5 days. Growth stimulation was observed in both the PE01 and PE04 cell lines but not in the SKOV-3 (ER-positive) or PE014 (ER-negative) lines. Expression of c-erbB2 and its tyrosine phosphorylation were measured by Western blot analysis using the CB11 (Novo Castra) and PY20 (Santa Cruz Biotechnology) monoclonal antibodies respectively. Relative c-erbB2 levels for unstimulated PE01, PE04, SKOV-3 and PE014 cells were 1 : 2 : 8 : 2.5 respectively as measured by densitometry. Upon stimulation with $E_2$, PE01 and PE04 cell lines showed increased tyrosine phosphorylation of c-erbB2 over a 15 min time course, this effect being mediated within 2 min. Expression of c-erbB2 remained unchanged throughout this time period. In contrast, $E_2$ produced no change in either tyrosine phosphorylation or c-erbB2 levels in SKOV-3 or PE014 cell lines.

In conclusion, ER-positive ovarian cancer cells expressing only low-moderate levels of c-erbB2 showed increased tyrosine phosphorylation when growth-stimulated with $E_2$. These data indicate that these signalling pathways are interactive in ovarian cancer.

### P185 FUNCTIONAL ANALYSIS OF NOVEL OVARIAN CANCER SUPPRESSOR REGIONS ON CHROMOSOME 11.

G.C. Sellar*, G.J. Rabiasz, L. Li, S. Al-Yahyahee, E.P. Miller, K.J. Taylor, A. Paige, J.E.V. Watson, D.J. Porteous†, J.F. Smyth, and H. Gabra. ICRF Medical Oncology Unit and †MRC Human Genetics Unit, Western General Hospital, Edinburgh UK.

LOH of an 8.5 Mb region on chromosome 11q has been associated with advanced FIGO stage and poor survival in patients with ovarian cancer. 556.1.5, a somatic cell hybrid, was used to transfer human chromosome 11 by microcell mediated chromosome transfer into OVCAR3 (an ovarian cancer cell line with chromosome 11q rearrangement). Chromosome 11 microcell hybrid clones (MHCs) remained immortalized and exhibited distinct phenotypes. Inhibition of invasiveness in matrigel localized to distal 11q in a 4.5Mb region. Analysis of 16 markers from the minimal region of interest in 87 cancer cell lines did not show any homozygous deletions. A growth suppression phenotype was found to localize outside the distal 11q region.

Candidate genes involved in the invasiveness and growth suppression phenotype pathways are being identified using Differential Display RT-PCR (DDRT-PCR), cDNA-Representational Difference Analysis and PCR-Select. DDRT-PCR has so far identified more than 20 products which are differentially expressed between MHCs of differing phenotypes and the control cell line. Products which relate either to the invasiveness phenotype or to the growth suppression phenotype have been identified. Characterization of these products is in progress. The DDRT-PCR products have been excised from acrylamide, eluted, re-amplified by PCR and subcloned into pGEM-T Easy vector. To determine their suitability for further analyses, including mutation screening by combined SSCPE/ Heteroduplex analysis, sub-cloned products are undergoing a standard screening protocol, comprising sequencing, database homology searching, chromosomal localization and expression profiling.

### P186 HIGH RISK HUMAN PAPILLOMAVIRUSES CAN INDUCE NUMERICAL CHROMOSOME ABNORMALITIES IN LOW-GRADE SQUAMOUS INTRAEPITHELIAL LESIONS,

A.Giannoudis*, S. A. Southern and C. S. Herrington, Department of Pathology, University of Liverpool Royal Liverpool University Hospital, Liverpool L69 3GA.

Human Papillomaviruses have been associated with benign and malignant neoplasms in humans and particularly with cervical cancer. However, viral infection appears to be an early event and additional abnormalities are required for biological transformation. We analysed 125 low grade cervical intraepithelial lesions (condylomata acuminata, flat condylomata, cervical intraepithelial neoplasia (CIN) grade 1) and 15 normal cervices for the presence of HPVs using both *in situ* hybridisation (ISH) and the polymerase chain reaction (PCR). PCR was performed with the L1 consensus primers GP5+/GP6+ followed by hybridisation using probes for HPV types 6, 11, 16, 18, 31, 33, 35, 39, 40, 42, 43, 44, 45, 51, 52, 56, 58, 59, 66 and 68. Interphase cytogenetic analysis using probes for chromosomes 1, 17, and X was also performed to identify numerical chromosomal abnormalities.

Of the 125 lesions, 43 contained low-risk, and 78 high-risk HPVs. Basal tetrasomy with all three chromosomes was identified in 20/78 (26%) lesions, infected with high-risk HPVs (HPV 16, 18, 31, 33, 35, 52, 56, 58, 66). None of the lesions infected with low-risk HPVs and none of 15 normal cervices showed any numerical chromosomal abnormality. Although tetrasomy was observed only in lesions infected with high-risk HPV, these changes were not type-specific implying that additional factors are required. However, these data indicate that induction of chromosomal instability by high risk, but not low-risk, HPV types, may be one mechanism underlying their biological differences.

HPV Chromosome abnormalities

### P187 Papilloma Virus (HPV) DNA sequences and HLA antigen expression in Papillary TCC bladder cancer.

AME Nouri, J Brewer, V Nargund, C Fowler, AMI Paris, RTD Oliver

A recent literature review of more than 700 cases in 15 publications has demonstrated that approximately 1 in 5 papillary bladder cancers express an HPV DNA sequences. As this proportion is similar to the number with normal HLA expression in our previous studies, this study sets out to examine the relationship between HLA expression in a series of bladder cancer patients.

Snap frozen tissue was stained using standard immunoperoxidase staining techniques for identification of HLA antigen expression, while standard pcr with low strngency for HPV viral DNA sequences.

7 of 43 (16%) were positive for a range of HPV (2 HPV 16, 3 EV related and 2 other mucosal subtypes). All 7 were W6/32 positive for HLA monomorphic antigen detected by W6/32 and 6/7 positive for class 1 heavy chain antigen defined by antibody HC10 while of the HPV negative tumours 75% were positive for W6/32 and 67% were positive for HC10.

The numbers tested are too small for definitive conclusion. However as there is increasing evidence from study of papilloma viruses in bovine bladder cancer that these viruses play a hit and run role in tumour initiation, further study of these observations may provide an explanation of the realtively low frequency of HPV infection in these obvious papillary tumours.